# Global Trends and Disparities in Social Isolation

**DOI:** 10.1001/jamanetworkopen.2025.32008

**Published:** 2025-09-15

**Authors:** Thomas E. Fuller-Rowell, Samia Sultana, Ichiro Kawachi

**Affiliations:** 1 Department of Human Development and Family Science, Auburn University, Auburn, Alabama; 2 Department of Social and Behavioral Sciences, Harvard T.H. Chan School of Public Health, Boston, Massachusetts

## Abstract

**Importance:**

Social isolation is consequential for human health and well-being. However, global trends and trends across countries, regions, and socioeconomic strata remain inadequately characterized, limiting targeted policy responses.

**Objectives:**

To quantify global changes in social isolation from 2009 to 2024 across within-country income groups and evaluate cross-country and regional variation in isolation levels and trends.

**Design, Setting, and Participants:**

This cross-sectional study used data from 2009 to 2024 from the Gallup World Poll, a globally representative repeated cross-sectional survey with consistent methodology across more than 150 countries. A random sample of approximately 1000 adults (age ≥15 years) within each country was selected at each annual assessment. Global trends in isolation were examined for the full sample and for top and bottom income groups, defined within each country.

**Main Outcomes and Measures:**

The prevalence of social isolation at each time point within each country was assessed as the proportion of respondents who answered “no” to having relatives or friends available to help in times of trouble. Household income was assessed in local currency and coded into 5 quintiles.

**Results:**

The study findings are based on data from 2 483 935 person-level assessments (mean [SD] age, 41.7 [17.9] years; 53.1% women) across 16 time points and 159 countries. Prepandemic global mean isolation levels were stable. A marked increase in isolation occurred between 2019 and 2020, concurrent with the onset of the COVID-19 pandemic, and was disproportionately seen in lower-income groups (β = 2.6 percentage points [95% CI, 0.9-4.4 percentage points]; *P* = .003; an 11.0% increase). From 2020 to 2024, isolation continued to increase, with steeper increases among higher-income groups (β = 1.9 [95% CI, 0.7-3.1]; *P* < .001; a 12.3% increase). The global prevalence of social isolation increased by 13.4% from 2009 to 2024 (from 19.2% [95% CI, 17.3%-21.6%] to 21.8% [95% CI, 19.4%-24.2%]), with the entire increase occurring after 2019. The disparity in isolation prevalence between high- and low-income groups peaked in 2020: 26.4% (95% CI, 23.6%-29.2%) of lower income groups were isolated vs 15.6% (95% CI, 13.6%-17.7%) of higher income groups. By 2024, global isolation was 2.6 percentage points (95% CI, 0.7-4.5 percentage points) above prepandemic levels and the income disparity was 8.6 percentage points (95% CI, 5.1-12.1 percentage points). A total of 54 countries experienced worsening isolation and widening disparities, while 41 saw improvements.

**Conclusions and Relevance:**

In this cross-sectional study, social isolation was found to have increased globally after the COVID-19 pandemic, with the initial increase disproportionately seen in lower-income populations and subsequent increases broadening across socioeconomic strata. Targeted interventions for vulnerable groups and research examining country-level policies are urgently needed to mitigate high isolation levels and reduce inequities.

## Key Points

**Question:**

How did the global prevalence and trajectories of social isolation change among within-country income groups between 2009 and 2024?

**Findings:**

In this repeated cross-sectional study of 159 countries, the global prevalence of social isolation increased by 13.4% over the 16-year study period (from 19.2 to 21.8), with the entire increase occurring after 2019. The disparity in isolation prevalence between high-income and low-income groups peaked in 2020 at 10.8 percentage points (high-income, 15.6% vs low-income, 26.4%).

**Meaning:**

This study suggests that targeted interventions to address disparities in isolation prevalence and increasing isolation levels are warranted.

## Teaser

This cross-sectional study uses data from over 2 million person-level assessments across 16 time points and 159 countries to examine changes in the global prevalence and trajectories of social isolation among within-country income groups between 2009 and 2024.

## Introduction

Public health and policy organizations worldwide—including the US surgeon general, the Organisation for Economic Co-operation and Development, and the World Health Organization—have warned of a “crisis in social connectedness,” citing its detrimental effects on individuals and communities.^1,2^ A growing literature has established that social isolation is associated with adverse mental and physical health outcomes through both direct and stress-exacerbating pathways,^3,4^ and that isolation increased in several countries during the COVID-19 pandemic.^5,6,7^ However, relatively little is known about global trends in isolation or variability in these trends across countries, world regions, or socioeconomic strata.^8,9,10^

Social isolation is embedded in broader social structures and shaped by macro-level conditions that influence social relations across demographic subgroups.^11^ Although some research suggests that lower-income groups compensate for economic hardships by forming strong social networks,^12,13^ most evidence indicates heightened risks of isolation due to discrimination, structural barriers, and resource limitations.^14,15^ Less-advantaged socioeconomic groups face disproportionate exposure to unfair treatment and exclusion,^16,17^ spatial marginalization or segregation from more advantaged peers,^18,19^ and social networks that are less equipped to provide psychosocial or material support.^20,21^ Furthermore, the widening income inequality observed in many countries in recent decades may have exacerbated these vulnerabilities, compounding their effects on social connectedness.^22,23^ However, global time trends in social isolation across socioeconomic groups remain largely unexplored.

Understanding how social isolation and loneliness have changed over time within and across countries and income groups is crucial for several reasons. Urbanization and the rise of digital lifestyles have transformed the structure of daily life. Technological advances have created new opportunities to combat isolation, such as digital communication tools that facilitate long-distance connections, participation in online communities, and access to virtual support networks.^24,25,26^ Urban environments also offer diverse cultural, social, and professional opportunities that can foster meaningful connections.^27^

On the other hand, these same forces can intensify social isolation. Increasing mobility for work has physically dispersed families and social networks,^28^ while societal norms emphasizing individualism have diminished communal values.^29^ Remote work, increasing amounts of screen time, and the decline of shared community spaces have reduced face-to-face interactions and weakened community ties.^30,31^ Collectively, these shifts have constrained opportunities for in-person engagement, potentially exacerbating experiences of isolation and loneliness.^32,33^

The COVID-19 pandemic further accelerated these trends, bringing renewed attention to the dynamics of social relationships. Although studies have examined pandemic-associated increases in isolation in some countries,^34,35^ there remains a significant gap in understanding longer-term cross-national patterns and differences across socioeconomic groups. In addition, it is unclear whether isolation levels are returning to prepandemic norms. This study seeks to bridge these gaps by examining global isolation trends across 159 countries from 2009 to 2024, with consideration of potential differences between high-income and low-income populations, defined within countries. Our approach allows for characterizing overarching global patterns and comparison of country-specific and region-specific trajectories.

## Methods

Data for this cross-sectional study were obtained from the Gallup World Poll, a repeated cross-sectional survey representing more than 98% of the world’s adult population.^36^ A probability-based nationally representative sample of adults (age, ≥15 years), with a sample size of approximately 1000, were surveyed annually in each country. We analyzed annual data from 159 countries spanning 16 time points from 2009 to 2024. The mean (SD) number of assessments was 14.7 (2.3). The data are widely available and deidentified and thus did not require ethical approval as a secondary analysis of deidentified survey data in accordance with the Common Rule 45 CFR 46.104(d)(4). This study followed the Strengthening the Reporting of Observational Studies in Epidemiology (STROBE) reporting guideline.

### Measures

#### Income Group

Household income was assessed in local currency and coded into 5 quintiles within each country. To maximize sample size and robustness of comparisons, we compared the bottom 2 quintiles (bottom 40%) with the top 2 quintiles (top 40%).^37,38,39^

#### Social Isolation

At each time point, participants were asked, “If you were in trouble, do you have relatives or friends you can count on to help you whenever you need them, or not?” Response options were “yes” or “no.” The proportion of “no” responses—indicating social isolation—was calculated at the country level for each income group. Specifically, the weighted count of socially isolated individuals in each income group was divided by the weighted total number of individuals in the group using Gallup’s final survey weights. Survey weights account for unequal probabilities of selection, nonresponse, and population demographic characteristics using poststratification methods.^36^

Although this measure is based on a single item, it is widely used in large-scale international surveys and has face validity as an indicator of a core element of social isolation—perceived lack of supportive relationships. This aligns with leading conceptualizations of social isolation, which emphasize the absence of available and reliable social ties.^40,41^ Descriptive statistics for each time point are shown in eTable 1 in Supplement 1.

### Statistical Analysis

Hierarchical linear models were estimated in R, version 4.4.3 (R Project for Statistical Computing), using the glmmTMB package.^42,43^ To determine the most appropriate trajectory, we first estimated unweighted maximum-likelihood models that contained different combinations of 3 growth terms: (1) a linear slope (coded 0-15 for 2009-2024), (2) a pandemic step change (0 before 2020, 1 from 2020 onward), and (3) a postpandemic slope adjustment (0 up to 2020, increasing by 1 each subsequent year). Nested structures were compared with likelihood-ratio χ^2^ tests, and nonnested structures with the Akaike Information Criterion and Bayesian Information Criterion. The specification retaining all 3 growth terms fit best. We then re-estimated this model with precision weights equal to the inverse sampling variance (1/SE^2^) to give greater influence to reliable country-year estimates and downweight noisier ones.^44^ An unconstrained 4 × 4 variance-covariance matrix captured random effects for the intercept and each slope, and we extracted country-level best linear unbiased predictions (BLUPs) from the final weighted model to visualize national trajectories.^44^ Robustness was assessed in 2 ways: comparing fixed-effect estimates from the weighted and unweighted fits to ensure weighting did not materially alter results (stability defined as |Δβ|<1 weighted SE) and re-estimating the weighted model with all random-effect covariances fixed to zero (diagonal G-matrix). Both checks confirmed the stability and robustness of the findings (eTables 2 and 3 in Supplement 1). A priori, the significance level was set at α = .05 (2-sided).

## Results

Models of global time trends in social isolation prevalence were based on data from a total of 2 483 935 participants (mean [SD] age, 41.7 [17.9]; 53.1% women and 46.9% men) from 159 countries between 2009 and 2024. The first trend structure considered was a simple linear slope across all 16 time points. It showed a 0.15-unit mean global increase in social isolation per year (95% CI, 0.03-0.26; *P* < .001), suggesting that by 2024, an additional 2.2% of the population in each country reported being socially isolated, compared with 2009 levels. Two alternative trend structures were then examined: an inflection point model allowed for a change in the linear slope after 2020, and a step-change model allowed for a break or shift in the trajectory between 2019 and 2020 (concurrent with the onset of the COVID-19 pandemic). In the full sample (all income groups), both models showed an improvement over the linear slope model, with the larger improvement coming from the step-change model (χ^2^_4_ = 102.00; *P* < .001) than from the slope-change model (χ^2^_4_ = 53.60; *P* < .001). Akaike information criterion and Bayes information criterion values indicated that the step-change model provided a better fit than the inflection point model for the full sample (eTable 4 in Supplement 1).

**Figure 1.**
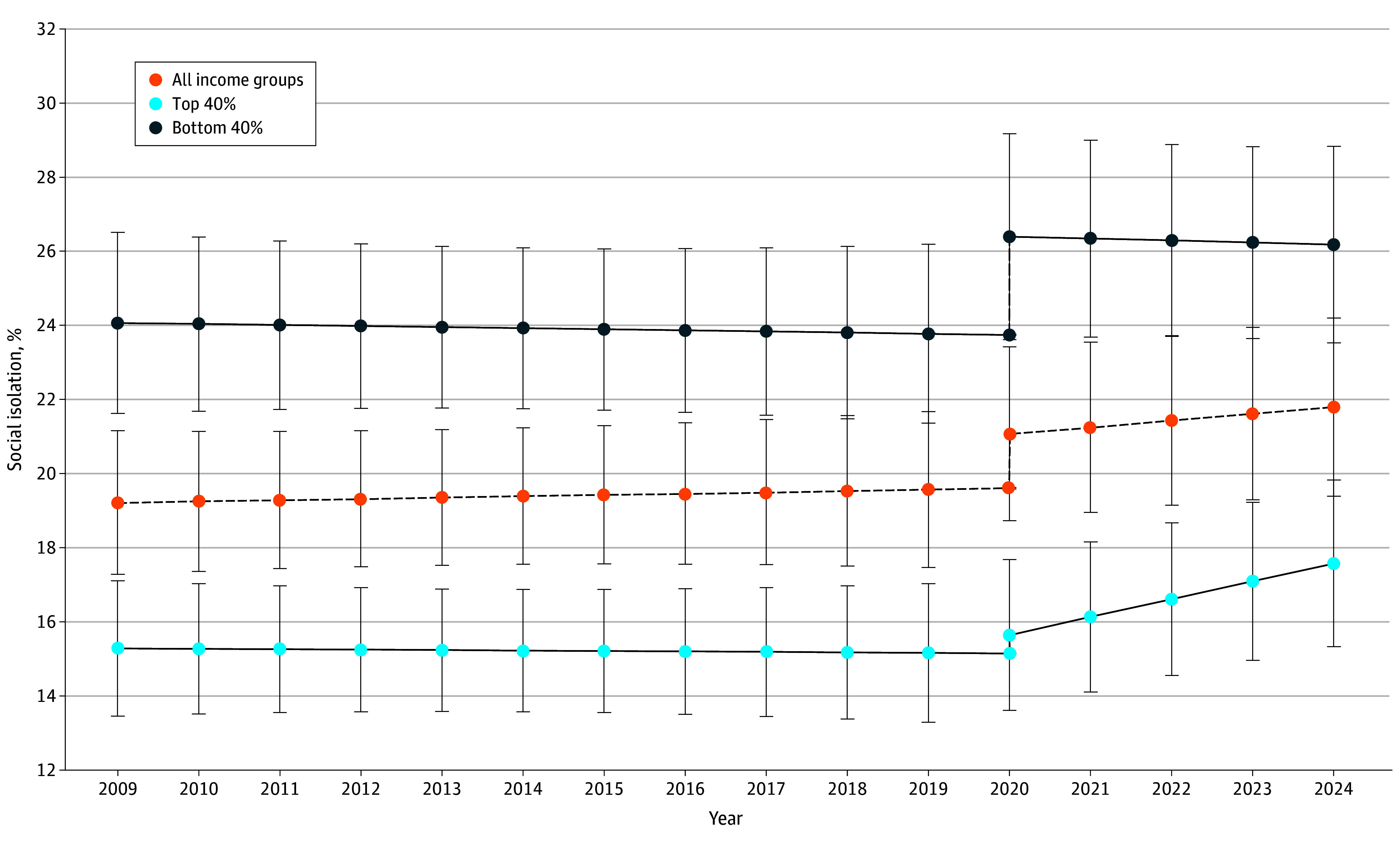
Fitted Global Trend in Social Isolation for the Total Population and by Income Group Estimates are from the final best-fitting model. Errors bars indicate 95% CIs of each point estimate.

Next, we fit a final model that combined the 2 above models, allowing both a step-change and a discontinuity in the slope after 2020. The likelihood ratio test indicated that this model was an improvement over the linear slope model (χ^2^_8_ = 139.2; *P* < .001), the step-change only model (χ^2^_4_ = 37.20; *P* < .001), and the slope-change only model (χ^2^_4_ = 85.60; *P* < .001). A plot of the global trend from the final model is shown in Figure 1, and the results are described below and shown in eTable 5 in Supplement 1.

### Final Model

In 2009, the global mean isolation prevalence was 19.2% (95% CI, 17.3%-21.2%; *P* < .001), and the gap between the top and bottom income groups was 8.8 percentage points (5.7-11.8 percentage points; *P* < .001). In relative terms, the bottom income group had 1.6 times higher levels of isolation than the top income group (24.1% socially isolated [95% CI, 21.6%-26.5%]) vs 15.3% [95% CI, 13.5%-17.1%]). The mean change in isolation from 2009 to 2024 was 2.6 percentage points (95% CI, 0.7-4.5 percentage points), a 13.4% increase from 19.2% (95% CI, 17.3%-21.2%) to 21.8% (95% CI, 19.4%-24.2%), equivalent to 0.2 SD units. By 2024, the income gap in social isolation was 8.6 percentage points (95% CI, 5.1-12.1 percentage points), with 26.2% (95% CI, 23.5%-28.8%) of lower-income individuals reporting isolation, compared with 17.6% (95% CI, 15.3%-19.8%) of higher-income individuals. The magnitude of this difference between high- and low-income groups was 0.7 SD units.

Prior to the COVID-19 pandemic (2009-2019), global isolation remained stable (*β* = 0.4 [95% CI, –1.3 to 2.0]; *P* = .68 for linear slope). However, a disruption occurred between 2019 and 2020, with social isolation levels increasing 1.5 percentage points (95% CI, 0.3-2.7 percentage points; *P* = .01), a 7.7% increase. This increase was driven by lower-income groups (*β* = 2.6 [95% CI, 0.9-4.4]; *P* = .003; an 11.0% increase), with higher-income groups showing no significant change (*β* = 0.5 [95% CI, –0.5 to 1.5]; *P* = .35).

Between 2020 and 2024, global increases in isolation remained stable (linear slope: *β* = 0.72 [95% CI, –0.52 to 1.96]; *P* = .25; a 3.4% increase), with an increase among the higher-income group (linear slope: *β* = 1.9 [95% CI, 0.7-3.1]; *P* = .001; a 12.3% increase), and with relative stability among lower-income groups (linear slope: *β* = −0.2 [95% CI, –1.9 to 1.4]; *P* = .80; an 0.8% increase). The income disparity in social isolation was largest in 2020 (*β* = 10.8 [95% CI, 7.3-14.2]; *P* < .001; a 22.4% increase from 2009), and by 2024 had returned to levels that were 1.9% lower than in 2009 (*β* = 8.8 [95% CI, 5.7-11.8]; *P* < .001). In 2020, 26.4% (95% CI, 23.6%-29.2%) of lower income groups were isolated vs 15.6% (95% CI, 13.6%-17.7%) of higher income groups.

**Figure 2.**
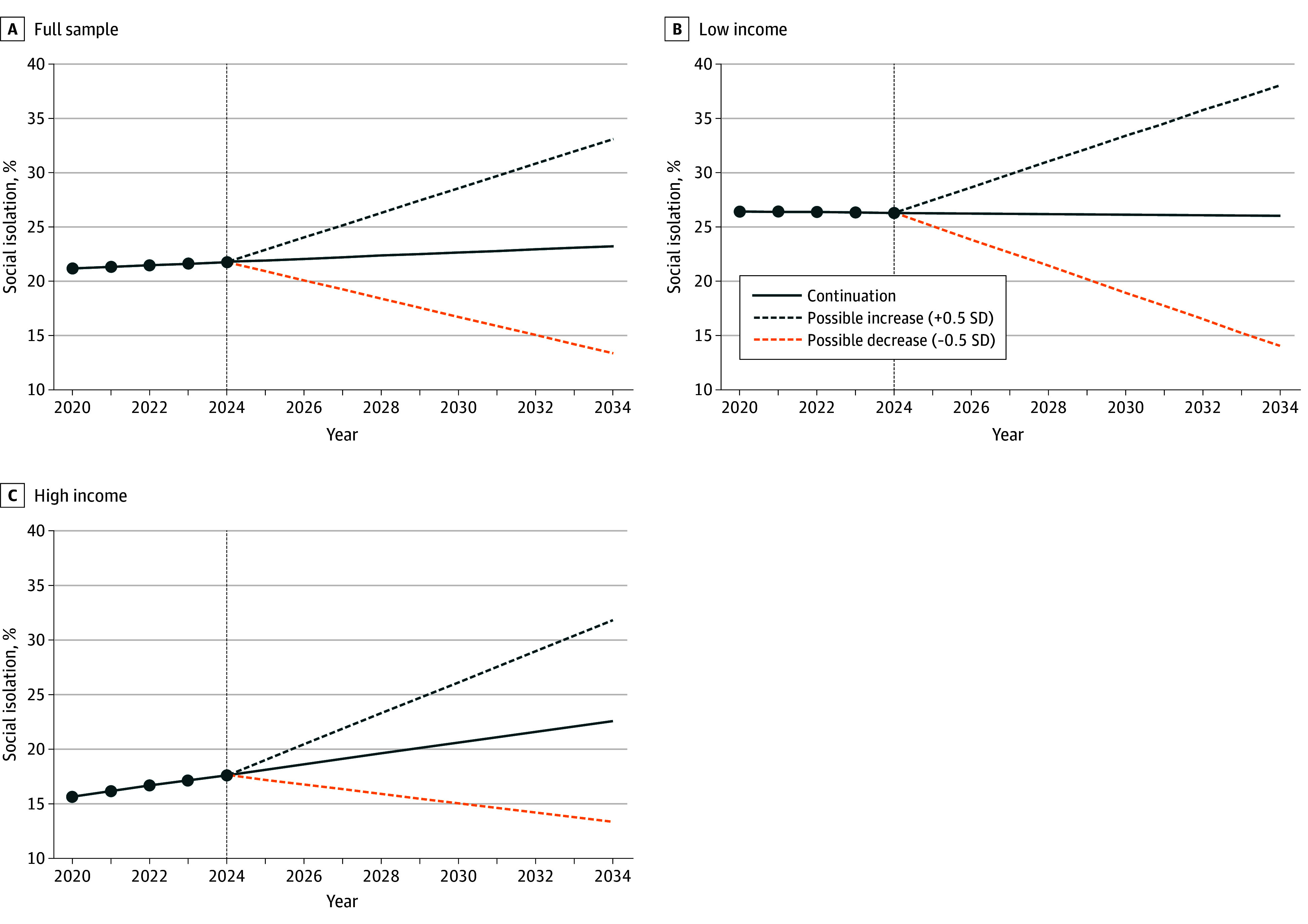
Projections for Social Isolation Trajectories

To stimulate discussion of implications, 10-year global projections for the full sample and for low-income and high-income groups are presented in Figure 2. The trajectories linearly extrapolate the post–COVID19 (2020-2024) slope under 3 scenarios: (1) continuation of the observed trend; (2) slope increases (+0.5 SD); and (3) slope decreases (–0.5 SD). The ±0.5-SD thresholds were derived from the cross-country distribution of 2020-2024 slopes, ensuring that the alternative paths reflect plausible bounds based on observed variability. Projections suggest that global mean levels would reach 23.1% (95% CI, 19.8%-26.5%) by 2034 if they continue the current trajectory, 33.0% (95% CI, 29.6%-36.3%) with worsening, and 13.3% (95% CI, 9.9%-16.7%) with improvement.

### Variability Across Countries and World Regions

**Figure 3.**
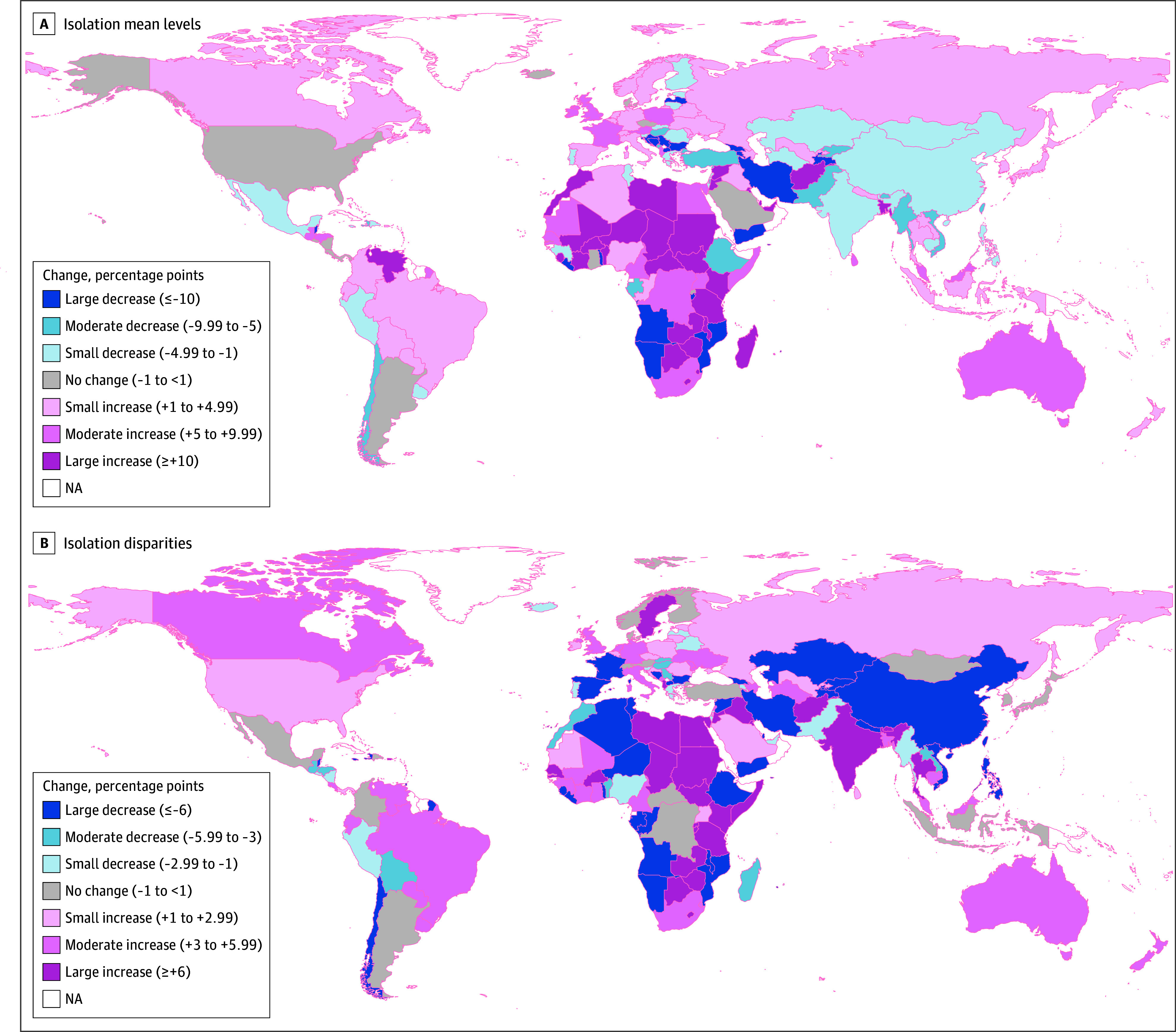
Global Variability With Respect to Change in Isolation Mean Levels and Disparities Units are percentage point magnitudes of change from 2009 to 2024, derived from fitted trajectories. NA indicates not available.

**Figure 4.**
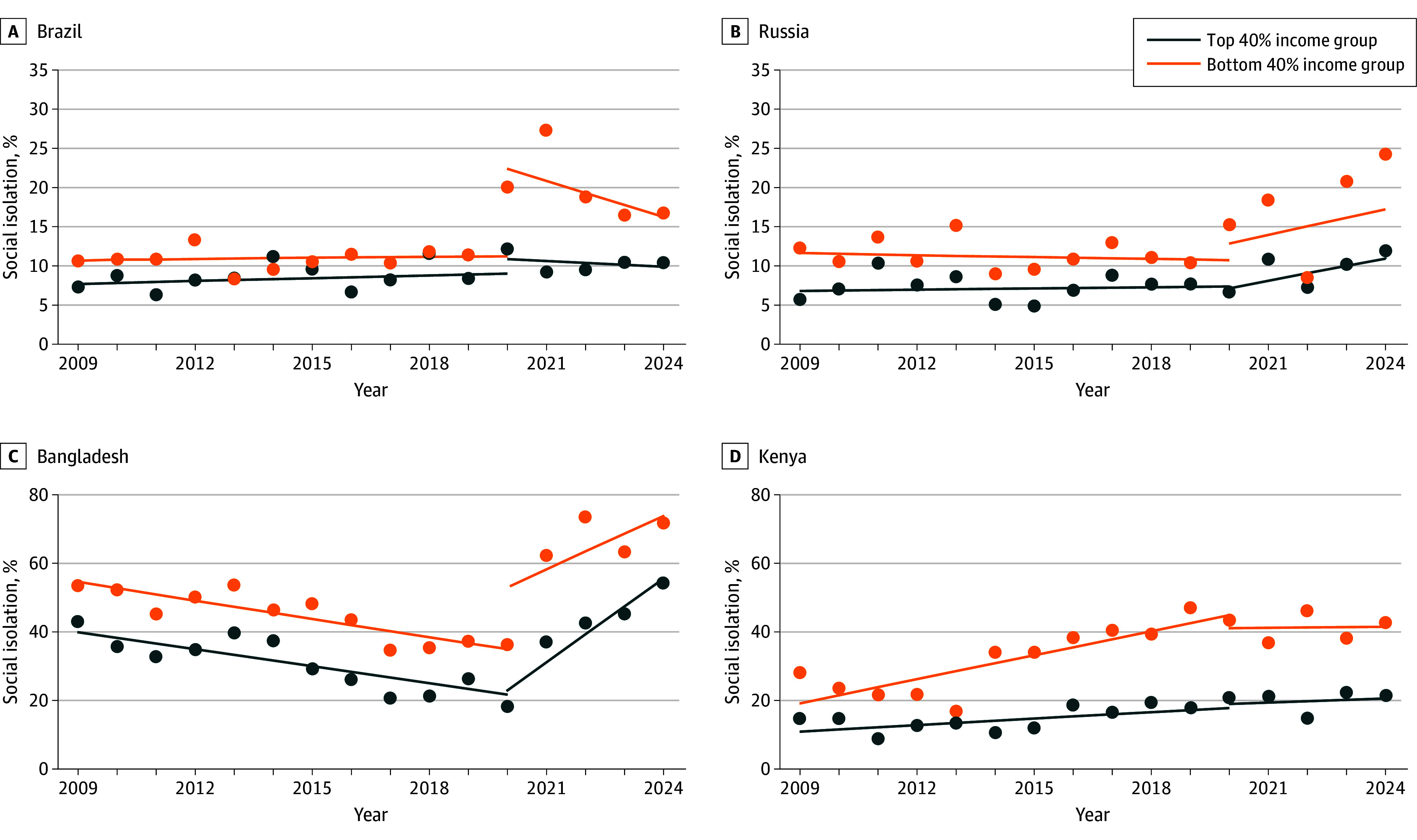
Trajectories of Social Isolation Trends in 4 Countries Fitted trajectories are derived from empirical Bayes estimates of the final best fitting model, with raw data overlaid.

Growth parameters (BLUPs) derived from the final weighted model were used to plot trajectories of isolation by income group for each country (eFigures 1-159 in Supplement 1). Four key values were derived for cross-country comparison of trajectories: change in mean level, 2009-2024; change in disparity, 2009-2024; final status 2024 level; and final status 2024 disparity. Global variability with respect to change in mean levels and disparities are visualized in Figure 3 (with rankings for countries, regions, and countries within regions shown in eTables 6-8 in Supplement 1, and trend types summarized in eTable 9 in Supplement 1). Among the 159 countries analyzed, 87 experienced an increase in social isolation (≥1 unit), 12 remained stable (<1 unit change), and 60 showed a decrease (≥1 unit). With respect to disparities, 75 of 159 showed an increase, 17 had no change, and 67 showed a decrease. A total of 54 countries experienced worsening isolation and widening disparities, while 41 saw improvements. Trajectories of 4 example countries showing increases in both levels and disparities are shown in Figure 4.

Country-specific intercept and slope coefficients (BLUPs) were averaged across countries within each region to produce region-level trajectories (eFigures 160-169 in Supplement 1). A list of world regions with descriptive statistics and rankings for trajectory parameters is shown in shown in eTable 7 in Supplement 1. Between 2009 and 2024, sub-Saharan Africa, Middle East and North Africa, and South Asia had the largest increase in mean levels, while Russia and the Former Soviet Union, Europe, and Southeast Asia had the largest decreases. With respect to changes in disparities, South Asia and Australia and New Zealand showed the largest increases, while the Middle East and North Africa and Southeast Asia showed the largest decreases.

**Figure 5.**
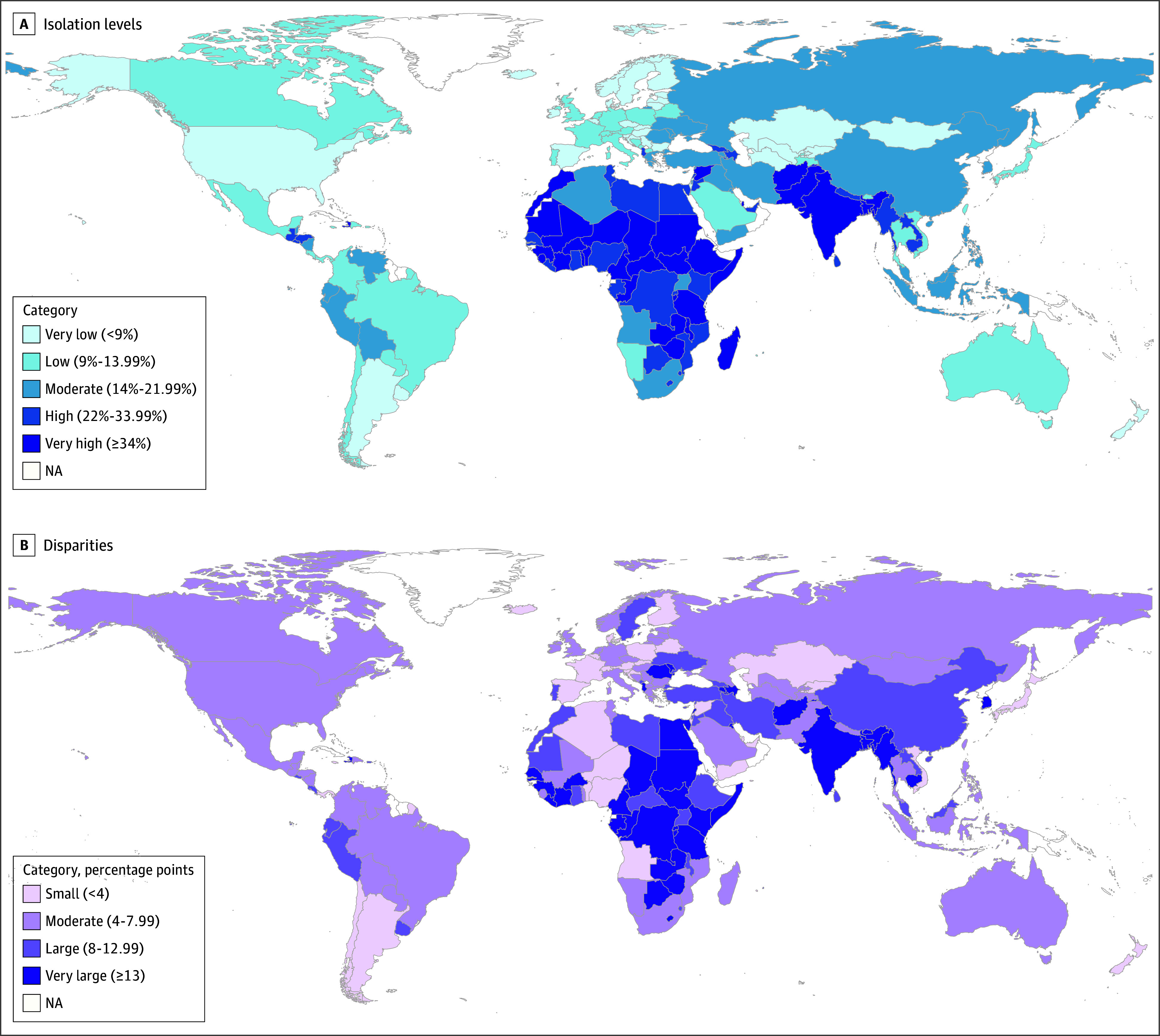
Global Variability in 2024 Isolation Levels and Disparities Units are prevalence estimates (percentage socially isolated) in 2024, derived from fitted trajectories. NA indicates not available.

Cross-country variability in final status 2024 levels and disparities is visualized in Figure 5 (with data and rankings for countries, regions, and countries within regions shown in eTables 10-12 in Supplement 1). South Asia, sub-Saharan Africa, and Middle East and North Africa had the highest mean levels, and North America, Australia and New Zealand, and Europe had the lowest (eTable 11 in Supplement 1). With respect to 2024 disparities, South Asia and sub-Saharan Africa had the highest and Australia and New Zealand and Europe had the lowest.

## Discussion

The results of this study indicate global increases in isolation among both high-income and low-income groups between 2009 and 2024, with substantial variability across countries and world regions. Prior to the COVID-19 pandemic, isolation levels remained stable, but a marked increase, likely due to the COVID-19 pandemic, occurred between 2019 and 2020, primarily seen in lower-income groups. From 2020 to 2024, isolation levels remained elevated and continued to increase for higher-income groups. Overall, the results show that a global increase in isolation occurred after 2019 with no evidence of a return to baseline thereafter. The size of the increase indicated that, on average, an additional 2.6% of the population in each country was socially isolated in 2024, compared with prepandemic levels (2009-2019). Put in the context of variability across countries, this increase is equivalent to one-fifth (22%) of an SD. These findings are consistent with prior research showing an increase in isolation in various country contexts after the pandemic.^5,6,7^ The current study extends this work by demonstrating the global magnitude of the increase and showing that the problem has continued to worsen rather than resolve between 2021 and 2024.

The magnitude of socioeconomic differences in isolation is notable, with 26.2% of lower-income individuals experiencing isolation, compared with 17.6% of higher-income individuals. In the context of variability across countries, this 8.6–percentage-point gap is equivalent to 0.7 SDs. These results extend theoretical^45,46,47^ and empirical^20,31,48,49,50,51,52^ work indicating that groups with more socioeconomic advantage have higher levels of social capital and less isolation.

To our knowledge, this study is among the first to demonstrate the global magnitude of income disparities in isolation and to show how these disparities are evolving around the world. Projection plots suggest that if current trends continue, global isolation levels will continue to increase by approximately 1.5 percentage points over the next decade—further diverging from prepandemic norms. However, the substantial cross-country variability in slopes suggests that global improvement is possible. A modest downward shift in trajectory (−0.5 SD) would put the world on a path to return to prepandemic isolation levels within roughly 3 years. Such a turnaround would likely require coordinated policy responses and collective action across countries.

Our analysis also illustrates the diversity of trajectories that exist across countries around the world. Comparison of world regions suggested that Russia and the Former Soviet Union showed the most overall combined improvement with respect to levels and disparities. Even though Russia and some former Soviet Union countries—such as Azerbaijan, Moldova, and Ukraine—showed increases in both levels and disparities, the overall trend for the Russia and the Former Soviet Union region was among the most favorable, owing to marked improvements in Armenia, Georgia, Kazakhstan, Kyrgyzstan, and Tajikistan (eTable 7 in Supplement 1). The specific reasons for these trends remain to be elucidated but suggest a clear trajectory of social improvement in several former Soviet Union countries.

Sub-Saharan Africa and South Asia showed a trajectory of worsening levels and disparities. Trends in South Asia were accounted for by substantial increases in mean levels and disparities in Afghanistan, Bangladesh, and Sri Lanka, and little improvement in other countries across the region. Sub-Saharan Africa had a diversity of trends across countries but 19 experienced an increase in both levels and disparities (Botswana, Burkina Faso, Cameroon, Chad, Cote d’Ivoire, Eswatini, Kenya, Lesotho, Mali, Mauritania, Senegal, Somalia, South Africa, South Sudan, Sudan, Tanzania, Uganda, Zambia, Zimbabwe), compared with 9 showing improvement (Angola, Ethiopia, Gabon, Gambia, Liberia, Mauritius, Mozambique, Namibia, Togo). South Asia and sub-Saharan Africa also had the highest social isolation levels and disparities at the trajectory end point (2024), with Australia and New Zealand, Europe, and North America showing the lowest end point levels and disparities (eTable 11 in Supplement 1). Although Australia and New Zealand showed the second-largest increase in disparities from 2009 to 2024 (behind only South Asia), this change was associated with the absence of any evident disparity at baseline. As a result, the magnitude of the disparity in 2024 remained the lowest of any world region. By highlighting this diversity in social isolation levels and trends, we aim to raise awareness of cross-national comparisons, stimulate discussion on underlying factors, and encourage policy action to reduce levels and disparities.

### Strengths and Limitations

This study has some strengths. A key strength is the study’s ability to examine social isolation trends across 159 countries over a 16-year period. Although the study’s broad geographic and temporal scope is a strength, it also presents limitations. One key limitation is the reliance on a single-item measure of isolation. Although this measure captures an essential aspect of social support, future research should explore additional dimensions of isolation and related constructs. Another limitation was that socioeconomic status focused on one dimension: income quintiles within each country. Consideration of other measures of socioeconomic status, such as educational level or occupational status, could add to this research.

The scope of our analyses and focus on cross-country comparisons also did not allow for consideration of how the analyses might differ across demographic subgroups (eg, age, gender, immigrant status, disability status, and intersections of demographic categories) or country-level predictors of trends. Future studies will be useful to elucidate demographic group differences and country-level determinants to provide insight into more targeted approaches to addressing social isolation.

## Conclusions

The findings of this repeated cross-sectional study suggest that social isolation has increased in many countries around the world. The findings also indicate that income disparities in isolation are large, with sizable variability across countries and over time, particularly in the years after the onset of the COVID-19 pandemic. The substantial income disparities observed underscore the need for targeted interventions, particularly in countries experiencing increasing isolation trends. Future research should examine country-specific factors, including policy changes, that may help to mitigate social isolation.

## Notes

Supplement 1.
eMethods

eResults
**eFigure 1.** Trends in Social Isolation for Tanzania by Income Group **eFigure 2.** Trends in Social Isolation for Congo (Kinshasa) by Income Group **eFigure 3.** Trends in Social Isolation for Central African Republic by Income Group **eFigure 4.** Trends in Social Isolation for Kenya by Income Group **eFigure 5.** Trends in Social Isolation for Zimbabwe by Income Group **eFigure 6.** Trends in Social Isolation for Botswana by Income Group **eFigure 7.** Trends in Social Isolation for Lesotho by Income Group **eFigure 8.** Trends in Social Isolation for Mali by Income Group **eFigure 9.** Trends in Social Isolation for Côte d’Ivoire by Income Group **eFigure 10.** Trends in Social Isolation for Sudan by Income Group **eFigure 11.** Trends in Social Isolation for Rwanda by Income Group **eFigure 12.** Trends in Social Isolation for Zambia by Income Group **eFigure 13.** Trends in Social Isolation for Liberia by Income Group **eFigure 14.** Trends in Social Isolation for Mauritania by Income Group **eFigure 15.** Trends in Social Isolation for Comoros by Income Group **eFigure 16.** Trends in Social Isolation for Burkina Faso by Income Group **eFigure 17.** Trends in Social Isolation for Sierra Leone by Income Group **eFigure 18.** Trends in Social Isolation for Eswatini by Income Group **eFigure 19.** Trends in Social Isolation for South Sudan by Income Group **eFigure 20.** Trends in Social Isolation for Chad by Income Group **eFigure 21.** Trends in Social Isolation for Malawi by Income Group **eFigure 22.** Trends in Social Isolation for Ghana by Income Group **eFigure 23.** Trends in Social Isolation for Somalia by Income Group **eFigure 24.** Trends in Social Isolation for Burundi by Income Group **eFigure 25.** Trends in Social Isolation for Gambia by Income Group **eFigure 26.** Trends in Social Isolation for Niger by Income Group **eFigure 27.** Trends in Social Isolation for Uganda by Income Group **eFigure 28.** Trends in Social Isolation for Cameroon by Income Group **eFigure 29.** Trends in Social Isolation for Benin by Income Group **eFigure 30.** Trends in Social Isolation for Guinea by Income Group **eFigure 31.** Trends in Social Isolation for Senegal by Income Group **eFigure 32.** Trends in Social Isolation for Nigeria by Income Group **eFigure 33.** Trends in Social Isolation for Congo Brazzaville by Income Group **eFigure 34.** Trends in Social Isolation for Madagascar by Income Group **eFigure 35.** Trends in Social Isolation for South Africa by Income Group **eFigure 36.** Trends in Social Isolation for Angola by Income Group **eFigure 37.** Trends in Social Isolation for Mozambique by Income Group **eFigure 38.** Trends in Social Isolation for Namibia by Income Group **eFigure 39.** Trends in Social Isolation for Gabon by Income Group **eFigure 40.** Trends in Social Isolation for Mauritius by Income Group **eFigure 41.** Trends in Social Isolation for Ethiopia by Income Group **eFigure 42.** Trends in Social Isolation for Togo by Income Group **eFigure 43.** Trends in Social Isolation for Afghanistan by Income Group **eFigure 44.** Trends in Social Isolation for Bangladesh by Income Group **eFigure 45.** Trends in Social Isolation for India by Income Group **eFigure 46.** Trends in Social Isolation for Bhutan by Income Group **eFigure 47.** Trends in Social Isolation for Sri Lanka by Income Group **eFigure 48.** Trends in Social Isolation for Pakistan by Income Group **eFigure 49.** Trends in Social Isolation for Nepal by Income Group **eFigure 50.** Trends in Social Isolation for Haiti by Income Group **eFigure 51.** Trends in Social Isolation for Brazil by Income Group **eFigure 52.** Trends in Social Isolation for Costa Rica by Income Group **eFigure 53.** Trends in Social Isolation for Colombia by Income Group **eFigure 54.** Trends in Social Isolation for Jamaica by Income Group **eFigure 55.** Trends in Social Isolation for Venezuela by Income Group **eFigure 56.** Trends in Social Isolation for Mexico by Income Group **eFigure 57.** Trends in Social Isolation for Argentina by Income Group **eFigure 58.** Trends in Social Isolation for Honduras by Income Group **eFigure 59.** Trends in Social Isolation for Ecuador by Income Group **eFigure 60.** Trends in Social Isolation for Panama by Income Group **eFigure 61.** Trends in Social Isolation for Bolivia by Income Group **eFigure 62.** Trends in Social Isolation for Dominican Republic by Income Group **eFigure 63.** Trends in Social Isolation for Guatamala by Income Group **eFigure 64.** Trends in Social Isolation for Nicaragua by Income Group **eFigure 65.** Trends in Social Isolation for Paraguay by Income Group **eFigure 66.** Trends in Social Isolation for Uruguay by Income Group **eFigure 67.** Trends in Social Isolation for Peru by Income Group **eFigure 68.** Trends in Social Isolation for El Salvador by Income Group **eFigure 69.** Trends in Social Isolation for Chile by Income Group **eFigure 70.** Trends in Social Isolation for Belize by Income Group **eFigure 71.** Trends in Social Isolation for Trinidad and Tobago by Income Group **eFigure 72.** Trends in Social Isolation for Puerto Rico by Income Group **eFigure 73.** Trends in Social Isolation for Jordan by Income Group **eFigure 74.** Trends in Social Isolation for Syria by Income Group **eFigure 75.** Trends in Social Isolation for Lebanon by Income Group **eFigure 76.** Trends in Social Isolation for Iraq by Income Group **eFigure 77.** Trends in Social Isolation for Morocco by Income Group **eFigure 78.** Trends in Social Isolation for Libya by Income Group **eFigure 79.** Trends in Social Isolation for Bahrain by Income Group **eFigure 80.** Trends in Social Isolation for the United Arab Emirates by Income Group **eFigure 81.** Trends in Social Isolation for Egypt by Income Group **eFigure 82.** Trends in Social Isolation for Kuwait by Income Group **eFigure 83.** Trends in Social Isolation for Saudi Arabia by Income Group **eFigure 84.** Trends in Social Isolation for Tunisia by Income Group **eFigure 85.** Trends in Social Isolation for Israel by Income Group **eFigure 86.** Trends in Social Isolation for Qatar by Income Group **eFigure 87.** Trends in Social Isolation for Türkiye by Income Group **eFigure 88.** Trends in Social Isolation for the State of Palestine by Income Group **eFigure 89.** Trends in Social Isolation for Algeria by Income Group **eFigure 90.** Trends in Social Isolation for Iran by Income Group **eFigure 91.** Trends in Social Isolation for Yemen by Income Group **eFigure 92.** Trends in Social Isolation for Canada by Income Group **eFigure 93.** Trends in Social Isolation for the United States of America by Income Group **eFigure 94.** Trends in Social Isolation for New Zealand by Income Group **eFigure 95.** Trends in Social Isolation for Australia by Income Group **eFigure 96.** Trends in Social Isolation for Thailand by Income Group **eFigure 97.** Trends in Social Isolation for Malaysia by Income Group **eFigure 98.** Trends in Social Isolation for Indonesia by Income Group **eFigure 99.** Trends in Social Isolation for Laos by Income Group **eFigure 100.** Trends in Social Isolation for Vietnam by Income Group **eFigure 101.** Trends in Social Isolation for Cambodia by Income Group **eFigure 102.** Trends in Social Isolation for Singapore by Income Group **eFigure 103.** Trends in Social Isolation for Myanmar by Income Group **eFigure 104.** Trends in Social Isolation for the Philippines by Income Group **eFigure 105.** Trends in Social Isolation for Albania by Income Group **eFigure 106.** Trends in Social Isolation for Luxembourg by Income Group **eFigure 107.** Trends in Social Isolation for Austria by Income Group **eFigure 108.** Trends in Social Isolation for Ireland by Income Group **eFigure 109.** Trends in Social Isolation for Germany by Income Group **eFigure 110.** Trends in Social Isolation for Belgium by Income Group **eFigure 111.** Trends in Social Isolation for Denmark by Income Group **eFigure 112.** Trends in Social Isolation for the United Kingdom of Great Britain and Northern Ireland **eFigure 113.** Trends in Social Isolation for Poland by Income Group **eFigure 114.** Trends in Social Isolation for Norway by Income Group **eFigure 115.** Trends in Social Isolation for Greece by Income Group **eFigure 116.** Trends in Social Isolation for Italy by Income Group **eFigure 117.** Trends in Social Isolation for the Netherlands by Income Group **eFigure 118.** Trends in Social Isolation for Sweden by Income Group **eFigure 119.** Trends in Social Isolation for Malta by Income Group **eFigure 120.** Trends in Social Isolation for the Republic of Cyprus by Income Group **eFigure 121.** Trends in Social Isolation for Spain by Income Group **eFigure 122.** Trends in Social Isolation for Iceland by Income Group **eFigure 123.** Trends in Social Isolation for Switzerland by Income Group **eFigure 124.** Trends in Social Isolation for Portugal by Income Group **eFigure 125.** Trends in Social Isolation for the Czech Republic by Income Group **eFigure 126.** Trends in Social Isolation for Slovenia by Income Group **eFigure 127.** Trends in Social Isolation for Estonia by Income Group **eFigure 128.** Trends in Social Isolation for France by Income Group **eFigure 129.** Trends in Social Isolation for Lithuania by Income Group **eFigure 130.** Trends in Social Isolation for North Macedonia by Income Group **eFigure 131.** Trends in Social Isolation for Montenegro by Income Group **eFigure 132.** Trends in Social Isolation for Finland by Income Group **eFigure 133.** Trends in Social Isolation for Slovakia by Income Group **eFigure 134.** Trends in Social Isolation for Croatia by Income Group **eFigure 135.** Trends in Social Isolation for Serbia by Income Group **eFigure 136.** Trends in Social Isolation for Latvia by Income Group **eFigure 137.** Trends in Social Isolation for Romania by Income Group **eFigure 138.** Trends in Social Isolation for Hungary by Income Group **eFigure 139.** Trends in Social Isolation for Bulgaria by Income Group **eFigure 140.** Trends in Social Isolation for Kosovo by Income Group **eFigure 141.** Trends in Social Isolation for Bosnia Herzegovina by Income Group **eFigure 142.** Trends in Social Isolation for Hong Kong by Income Group **eFigure 143.** Trends in Social Isolation for Japan by Income Group **eFigure 144.** Trends in Social Isolation for South Korea by Income Group **eFigure 145.** Trends in Social Isolation for Mongolia by Income Group **eFigure 146.** Trends in Social Isolation for Taiwan by Income Group **eFigure 147.** Trends in Social Isolation for China by Income Group **eFigure 148.** Trends in Social Isolation for Azerbizan by Income Group **eFigure 149.** Trends in Social Isolation for Russia by Income Group **eFigure 150.** Trends in Social Isolation for Uzbekistan by Income Group **eFigure 151.** Trends in Social Isolation for Moldova by Income Group **eFigure 152.** Trends in Social Isolation for Ukraine by Income Group **eFigure 153.** Trends in Social Isolation for Turkmenistan by Income Group **eFigure 154.** Trends in Social Isolation for Belarus by Income Group **eFigure 155.** Trends in Social Isolation for Kyrgyzstan by Income Group **eFigure 156.** Trends in Social Isolation for Kazakhstan by Income Group **eFigure 157.** Trends in Social Isolation for Armenia by Income Group **eFigure 158.** Trends in Social Isolation for Tajikistan by Income Group **eFigure 159.** Trends in Social Isolation for Georgia by Income Group **eFigure 160.** Trends in Social Isolation for Sub-Saharan Africa by Income Group **eFigure 161.** Trends in Social Isolation for South Asia by Income Group **eFigure 162.** Trends in Social Isolation for Latin America and the Caribbean (LAC) by Income Group **eFigure 163.** Trends in Social Isolation for Middle East and North Africa (MENA) by Income Group **eFigure 164.** Trends in Social Isolation for North America by Income Group **eFigure 165.** Trends in Social Isolation for ANZ (Australia and New Zealand) by Income Group **eFigure 166.** Trends in Social Isolation for Southeast Asia by Income Group **eFigure 167.** Trends in Social Isolation for Europe by Income Group **eFigure 168.** Trends in Social Isolation for East Asia by Income Group **eFigure 169.** Trends in Social Isolation for RFSU (Russia and the Former Soviet Union) by Income Group **eTable 1.** Raw Global Descriptive Statistics for Social Isolation at Each Timepoint **eTable 2.** Comparison of Fixed-Effect Estimates From the Weighted and Unweighted Models **eTable 3.** Comparing Fixed-Effect Estimates From the Unrestricted and Diagonal G-Matrix Models **eTable 4.** Comparison of Model Fit Statistics for Alternative Trajectory Structures **eTable 5.** Multilevel Discontinuous Growth Curve Parameter Estimates for Full Sample, Low-Income (Bottom 40%), and High-Income (Top 40%) Group **eTable 6.** Country Rankings on Changes in Social Isolation Trajectories (2009–2024) **eTable 7.** Regional Rankings from Best to Worst for Social Isolation Trajectories (2009-2024) **eTable 8.** Country Rankings Within Each Region on Changes in Social Isolation Trajectories (2009-2024) **eTable 9.** Global Summary of Social Isolation Trends by Trend Type **eTable 10.** Country Rankings for Social Isolation Levels and Disparities in 2024 (Best to Worst) **eTable 11.** Regional Rankings for Social Isolation Levels and Disparities in 2024 (Best to Worst) **eTable 12.** Country Ranking Within Each Region on Social Isolation Trajectories in 2024

Supplement 2.
Data Sharing Statement

## References

[1] Office of the Surgeon General. *Our Epidemic of Loneliness and Isolation: The U.S. Surgeon General’s Advisory on the Healing Effects of Social Connection and Community*. US Dept of Health and Human Services; 2023. Accessed January 13, 2025. https://www.ncbi.nlm.nih.gov/books/NBK595227/37792968

[2] Social isolation and loneliness among older people: advocacy brief. World Health Organization. 2021. Accessed January 13, 2025. https://iris.who.int/handle/10665/343206

[3] Berkman LF, Glass T, Brissette I, Seeman TE. From social integration to health: Durkheim in the new millennium. Soc Sci Med. 2000;51(6):843-857. doi:10.1016/S0277-9536(00)00065-4 10972429

[4] Wang F, Gao Y, Han Z, . A systematic review and meta-analysis of 90 cohort studies of social isolation, loneliness and mortality. Nat Hum Behav. 2023;7(8):1307-1319. doi:10.1038/s41562-023-01617-6 37337095

[5] Hettich N, Entringer TM, Kroeger H, . Impact of the COVID-19 pandemic on depression, anxiety, loneliness, and satisfaction in the German general population: a longitudinal analysis. Soc Psychiatry Psychiatr Epidemiol. 2022;57(12):2481-2490. doi:10.1007/s00127-022-02311-0 35680681 PMC9181932

[6] Kannan VD, Veazie PJ. US trends in social isolation, social engagement, and companionship—nationally and by age, sex, race/ethnicity, family income, and work hours, 2003-2020. SSM Popul Health. 2022;21:101331. doi:10.1016/j.ssmph.2022.101331 36618547 PMC9811250

[7] Sugaya N, Yamamoto T, Suzuki N, Uchiumi C. Loneliness and social isolation factors under the prolonged COVID-19 pandemic in Japan: 2-year longitudinal study. JMIR Public Health Surveill. 2024;10:e51653. doi:10.2196/51653 39250195 PMC11420607

[8] Surkalim DL, Luo M, Eres R, . The prevalence of loneliness across 113 countries: systematic review and meta-analysis. BMJ. 2022;376:e067068. doi:10.1136/bmj-2021-067068 35140066 PMC8826180

[9] Teo RH, Cheng WH, Cheng LJ, Lau Y, Lau ST. Global prevalence of social isolation among community-dwelling older adults: a systematic review and meta-analysis. Arch Gerontol Geriatr. 2023;107:104904. doi:10.1016/j.archger.2022.104904 36563614

[10] Lim MH, Qualter P, Ding D, Holt-Lunstad J, Mikton C, Smith BJ. Advancing loneliness and social isolation as global health challenges: taking three priority actions. Public Health Res Pract. 2023;33(3):e3332320. doi:10.17061/phrp3332320 37699761

[11] Kawachi I, Berkman LF. Social capital, social cohesion, and health. In: Berkman LF, Kawachi I, Glymour MM, eds. Social Epidemiology. 2nd ed. Oxford University Press; 2014:290-319.

[12] Edin K, Lein L. Making Ends Meet: How Single Mothers Survive Welfare and Low-Wage Work. Russell Sage Foundation; 1997.

[13] Small ML. Unanticipated Gains: Origins of Network Inequality in Everyday Life. Oxford University Press; 2009. doi:10.1093/acprof:oso/9780195384352.001.0001

[14] Taylor HO, Cudjoe TKM, Bu F, Lim MH. The state of loneliness and social isolation research: current knowledge and future directions. BMC Public Health. 2023;23(1):1049. doi:10.1186/s12889-023-15967-3 37264355 PMC10233527

[15] Wang Y, Liu M, Yang F, Chen H, Wang Y, Liu J. The associations of socioeconomic status, social activities, and loneliness with depressive symptoms in adults aged 50 years and older across 24 countries: findings from five prospective cohort studies. Lancet Healthy Longev. 2024;5(9):100618. doi:10.1016/j.lanhl.2024.07.001 39208829

[16] Fuller-Rowell TE, Curtis DS, Chae DH, Ryff CD. Longitudinal health consequences of socioeconomic disadvantage: examining perceived discrimination as a mediator. Health Psychol. 2018;37(5):491-500. doi:10.1037/hea0000616 29698020 PMC5926810

[17] Fuller-Rowell TE, Saini EK, El-Sheikh M. Social class discrimination during adolescence as a mediator of socioeconomic disparities in actigraphy-assessed and self-reported sleep. Sleep Med. 2023;108:61-70. doi:10.1016/j.sleep.2023.05.021 37331131 PMC10395515

[18] Kawachi I, Subramanian SV, Kim D. Social capital and health. In: Kawachi I, Subramanian SV, Kim D, eds. Social Capital and Health. Springer; 2008:1-26. doi:10.1007/978-0-387-71311-3_1

[19] van Ham M, Tammaru T, Ubarevičienė R, Janssen H, eds. Urban Socio-Economic Segregation and Income Inequality: A Global Perspective. Springer Nature; 2021. doi:10.1007/978-3-030-64569-4

[20] Matthews KA, Gallo LC, Taylor SE. Are psychosocial factors mediators of socioeconomic status and health connections? a progress report and blueprint for the future. Ann N Y Acad Sci. 2010;1186(1):146-173. doi:10.1111/j.1749-6632.2009.05332.x 20201872

[21] Uphoff EP, Pickett KE, Cabieses B, Small N, Wright J. A systematic review of the relationships between social capital and socioeconomic inequalities in health: a contribution to understanding the psychosocial pathway of health inequalities. Int J Equity Health. 2013;12(1):54. doi:10.1186/1475-9276-12-54 23870068 PMC3726325

[22] Chetty R, Jackson MO, Kuchler T, . Social capital II: determinants of economic connectedness. Nature. 2022;608(7921):122-134. doi:10.1038/s41586-022-04997-3 35915343 PMC9352593

[23] Watson T. Inequality and the measurement of residential segregation by income in American neighborhoods. Rev Income Wealth. 2009;55(3):820-844. doi:10.1111/j.1475-4991.2009.00346.x

[24] Newman K, Wang AH, Wang AZY, Hanna D. The role of internet-based digital tools in reducing social isolation and addressing support needs among informal caregivers: a scoping review. BMC Public Health. 2019;19(1):1495. doi:10.1186/s12889-019-7837-3 31706294 PMC6842183

[25] Sen K, Prybutok G, Prybutok V. The use of digital technology for social wellbeing reduces social isolation in older adults: a systematic review. SSM Popul Health. 2021;17:101020. doi:10.1016/j.ssmph.2021.101020 35024424 PMC8733322

[26] Peng S, Roth AR. Social isolation and loneliness before and during the COVID-19 pandemic: a longitudinal study of U.S. adults older than 50. J Gerontol B Psychol Sci Soc Sci. 2022;77(7):e185-e190. doi:10.1093/geronb/gbab068 33870414 PMC8083229

[27] Bollenbach L, Niermann C, Schmitz J, Kanning M. Social participation in the city: exploring the moderating effect of walkability on the associations between active mobility, neighborhood perceptions, and social activities in urban adults. BMC Public Health. 2023;23(1):2450. doi:10.1186/s12889-023-17366-0 38062419 PMC10701942

[28] Kibria N. Globalization and the family: introduction to the special issue of *International Journal of Sociology of the Family*. Int J Sociol Fam. 2006;32(2):137-139.

[29] Santos HC, Varnum MEW, Grossmann I. Global increases in individualism. Psychol Sci. 2017;28(9):1228-1239. doi:10.1177/0956797617700622 28703638

[30] Leonardi PM, Parker SH, Shen R. How remote work changes the world of work. Annual Review of Organizational Psychology and Organizational Behavior. 2024;11:193-219. doi:10.1146/annurev-orgpsych-091922-015852

[31] Putnam RD. Bowling alone: America’s declining social capital. In: LeGates RT, Stout F, eds. The City Reader. 6th ed. Routledge; 2015:1-9.

[32] Oakman J, Kinsman N, Stuckey R, Graham M, Weale V. A rapid review of mental and physical health effects of working at home: how do we optimise health? BMC Public Health. 2020;20(1):1825. doi:10.1186/s12889-020-09875-z 33256652 PMC7703513

[33] Van Zoonen W, Sivunen AE. The impact of remote work and mediated communication frequency on isolation and psychological distress. Eur J Work Organ Psychol. 2022;31(4):610-621. doi:10.1080/1359432X.2021.2002299

[34] Ernst M, Niederer D, Werner AM, . Loneliness before and during the COVID-19 pandemic: a systematic review with meta-analysis. Am Psychol. 2022;77(5):660-677. doi:10.1037/amp0001005 35533109 PMC9768682

[35] Su Y, Rao W, Li M, Caron G, D’Arcy C, Meng X. Prevalence of loneliness and social isolation among older adults during the COVID-19 pandemic: a systematic review and meta-analysis. Int Psychogeriatr. 2023;35(5):229-241. doi:10.1017/S1041610222000199 35357280

[36] Worldwide research: methodology and codebook. Gallup Inc. 2024. Accessed April 1, 2025. https://www.unicef.org/innocenti/media/3346/file/Gallup-World-Poll-Methodology-2023.pdf

[37] Deaton A. Income, health, and well-being around the world: evidence from the Gallup World Poll. J Econ Perspect. 2008;22(2):53-72. doi:10.1257/jep.22.2.53 19436768 PMC2680297

[38] Lomas T. Exploring associations between income and wellbeing: new global insights from the Gallup World Poll. J Posit Psychol. 2024;19(4):629-646. doi:10.1080/17439760.2023.2248963

[39] Powdthavee N, Burkhauser RV, De Neve JE. Top incomes and human well-being: evidence from the Gallup World Poll. J Econ Psychol. 2017;62:246-257. doi:10.1016/j.joep.2017.07.006

[40] Berkman LF. Social support, social networks, social cohesion and health. Soc Work Health Care. 2000;31(2):3-14. doi:10.1300/J010v31n02_02 11081851

[41] Taylor SE. Social support: a review. In: Friedman HS, ed. The Oxford Handbook of Health Psychology. Oxford University Press; 2012:190-214. doi:10.1093/oxfordhb/9780195342819.013.0009

[42] Lumley T. Complex Surveys: A Guide to Analysis Using R. John Wiley & Sons; 2011.

[43] Brooks M, Bolker B, Kristensen K, . glmmTMB: Generalized linear mixed models using Template Model Builder. April 2, 2025. Accessed July 7, 2025. https://cran.r-project.org/web/packages/glmmTMB/index.html

[44] Raudenbush SW, Bryk AS. Hierarchical Linear Models: Applications and Data Analysis Methods. 2nd ed. Sage Publications; 2002.

[45] Berkman LF, Kawachi I, Glymour MM. Social Epidemiology. Oxford University Press; 2014.

[46] Bourdieu P. The forms of capital. In: Richardson JG, ed. Handbook of Theory and Research for the Sociology of Education. Greenwood; 1986:241-258.

[47] Coleman JS. Social capital in the creation of human capital. Am J Sociol. 1988;94(1):S95-S120. doi:10.1086/228943

[48] Beller J. Social inequalities in loneliness: disentangling the contributions of education, income, and occupation. SAGE Open. 2024;14(3). doi:10.1177/21582440241281408

[49] Huxhold O, Fiori KL, Windsor TD. The dynamic interplay of social network characteristics, subjective well-being, and health: the costs and benefits of socio-emotional selectivity. Psychol Aging. 2013;28(1):3-16. doi:10.1037/a0030170 23066804

[50] Kawachi I, Kennedy BP, Glass R. Social capital and self-rated health: a contextual analysis. Am J Public Health. 1999;89(8):1187-1193. doi:10.2105/AJPH.89.8.1187 10432904 PMC1508687

[51] Murayama H, Fujiwara Y, Kawachi I. Social capital and health: a review of prospective multilevel studies. J Epidemiol. 2012;22(3):179-187. doi:10.2188/jea.JE20110128 22447212 PMC3798618

[52] Pinquart M, Sorensen S. Influences on loneliness in older adults: a meta-analysis. Basic Appl Soc Psych. 2001;23(4):245-266. doi:10.1207/S15324834BASP2304_2

