# Error in Key Points

**DOI:** 10.1001/jamanetworkopen.2025.42520

**Published:** 2025-10-15

In the Original Investigation titled “Global Trends and Disparities in Social Isolation,”^1^ published September 15, 2025, the prevalence values for the high- and low-income groups were inadvertently transposed in the Key Points. This article has been corrected.
