# Supplementary material for: Global Trends and Disparities in Social Isolation
Source: JAMA Netw Open. 2025 Sep 15;8(9):e2532008. doi: 10.1001/jamanetworkopen.2025.32008 (PMC12439063; doi:10.1001/jamanetworkopen.2025.32008)
Supplement: Supplement 1. — eMethods eResults eFigure 1. Trends in Social Isolation for Tanzania by Income Group eFigure 2. Trends in Social Isolation for Congo (Kinshasa) by Income Group eFigure 3. Trends in Social Isolation for Central African Republic by Income Group eFigure 4. Trends in Social Isolation for Kenya by Income Group eFigure 5. Trends in Social Isolation for Zimbabwe by Income Group eFigure 6. Trends in Social Isolation for Botswana by Income Group eFigure 7. Trends in Social Isolation for Lesotho by Income Group eFigure 8. Trends in Social Isolation for Mali by Income Group eFigure 9. Trends in Social Isolation for Côte d’Ivoire by Income Group eFigure 10. Trends in Social Isolation for Sudan by Income Group eFigure 11. Trends in Social Isolation for Rwanda by Income Group eFigure 12. Trends in Social Isolation for Zambia by Income Group eFigure 13. Trends in Social Isolation for Liberia by Income Group eFigure 14. Trends in Social Isolation for Mauritania by Income Group eFigure 15. Trends in Social Isolation for Comoros by Income Group eFigure 16. Trends in Social Isolation for Burkina Faso by Income Group eFigure 17. Trends in Social Isolation for Sierra Leone by Income Group eFigure 18. Trends in Social Isolation for Eswatini by Income Group eFigure 19. Trends in Social Isolation for South Sudan by Income Group eFigure 20. Trends in Social Isolation for Chad by Income Group eFigure 21. Trends in Social Isolation for Malawi by Income Group eFigure 22. Trends in Social Isolation for Ghana by Income Group eFigure 23. Trends in Social Isolation for Somalia by Income Group eFigure 24. Trends in Social Isolation for Burundi by Income Group eFigure 25. Trends in Social Isolation for Gambia by Income Group eFigure 26. Trends in Social Isolation for Niger by Income Group eFigure 27. Trends in Social Isolation for Uganda by Income Group eFigure 28. Trends in Social Isolation for Cameroon by Income Group eFigure 29. Trends in Social Isolation for Benin by Income Group eFigure 30. Tre [file jamanetwopen-e2532008-s001.pdf]

Supplementary Online Content

Fuller-Rowell TE, Sultana S, Kawachi I. Global trends and disparities in social isolation. *JAMA Netw Open.* 2025;8(9):e2532008. doi:10.1001/jamanetworkopen.2025.32008

eMethods

eResults

eFigure 1. Trends in Social Isolation for Tanzania by Income Group

eFigure 2. Trends in Social Isolation for Congo (Kinshasa) by Income Group

eFigure 3. Trends in Social Isolation for Central African Republic by Income Group

eFigure 4. Trends in Social Isolation for Kenya by Income Group

eFigure 5. Trends in Social Isolation for Zimbabwe by Income Group

eFigure 6. Trends in Social Isolation for Botswana by Income Group

eFigure 7. Trends in Social Isolation for Lesotho by Income Group

eFigure 8. Trends in Social Isolation for Mali by Income Group

eFigure 9. Trends in Social Isolation for Côte d'Ivoire by Income Group

eFigure 10. Trends in Social Isolation for Sudan by Income Group

eFigure 11. Trends in Social Isolation for Rwanda by Income Group

eFigure 12. Trends in Social Isolation for Zambia by Income Group

eFigure 13. Trends in Social Isolation for Liberia by Income Group

eFigure 14. Trends in Social Isolation for Mauritania by Income Group

eFigure 15. Trends in Social Isolation for Comoros by Income Group

eFigure 16. Trends in Social Isolation for Burkina Faso by Income Group

eFigure 17. Trends in Social Isolation for Sierra Leone by Income Group

eFigure 18. Trends in Social Isolation for Eswatini by Income Group

eFigure 19. Trends in Social Isolation for South Sudan by Income Group

eFigure 20. Trends in Social Isolation for Chad by Income Group

eFigure 21. Trends in Social Isolation for Malawi by Income Group

© 2025 Fuller-Rowell TE et al. *JAMA Network Open.*

eFigure 22. Trends in Social Isolation for Ghana by Income Group

eFigure 23. Trends in Social Isolation for Somalia by Income Group

eFigure 24. Trends in Social Isolation for Burundi by Income Group

eFigure 25. Trends in Social Isolation for Gambia by Income Group

eFigure 26. Trends in Social Isolation for Niger by Income Group

eFigure 27. Trends in Social Isolation for Uganda by Income Group

eFigure 28. Trends in Social Isolation for Cameroon by Income Group

eFigure 29. Trends in Social Isolation for Benin by Income Group

eFigure 30. Trends in Social Isolation for Guinea by Income Group

eFigure 31. Trends in Social Isolation for Senegal by Income Group

eFigure 32. Trends in Social Isolation for Nigeria by Income Group

eFigure 33. Trends in Social Isolation for Congo Brazzaville by Income Group

eFigure 34. Trends in Social Isolation for Madagascar by Income Group

eFigure 35. Trends in Social Isolation for South Africa by Income Group

eFigure 36. Trends in Social Isolation for Angola by Income Group

eFigure 37. Trends in Social Isolation for Mozambique by Income Group

eFigure 38. Trends in Social Isolation for Namibia by Income Group

eFigure 39. Trends in Social Isolation for Gabon by Income Group

eFigure 40. Trends in Social Isolation for Mauritius by Income Group

eFigure 41. Trends in Social Isolation for Ethiopia by Income Group

eFigure 42. Trends in Social Isolation for Togo by Income Group

eFigure 43. Trends in Social Isolation for Afghanistan by Income Group

eFigure 44. Trends in Social Isolation for Bangladesh by Income Group

eFigure 45. Trends in Social Isolation for India by Income Group

eFigure 46. Trends in Social Isolation for Bhutan by Income Group

eFigure 47. Trends in Social Isolation for Sri Lanka by Income Group

eFigure 48. Trends in Social Isolation for Pakistan by Income Group

eFigure 49. Trends in Social Isolation for Nepal by Income Group

© 2025 Fuller-Rowell TE et al. *JAMA Network Open.*

eFigure 50. Trends in Social Isolation for Haiti by Income Group

eFigure 51. Trends in Social Isolation for Brazil by Income Group

eFigure 52. Trends in Social Isolation for Costa Rica by Income Group

eFigure 53. Trends in Social Isolation for Colombia by Income Group

eFigure 54. Trends in Social Isolation for Jamaica by Income Group

eFigure 55. Trends in Social Isolation for Venezuela by Income Group

eFigure 56. Trends in Social Isolation for Mexico by Income Group

eFigure 57. Trends in Social Isolation for Argentina by Income Group

eFigure 58. Trends in Social Isolation for Honduras by Income Group

eFigure 59. Trends in Social Isolation for Ecuador by Income Group

eFigure 60. Trends in Social Isolation for Panama by Income Group

eFigure 61. Trends in Social Isolation for Bolivia by Income Group

eFigure 62. Trends in Social Isolation for Dominican Republic by Income Group

eFigure 63. Trends in Social Isolation for Guatamala by Income Group

eFigure 64. Trends in Social Isolation for Nicaragua by Income Group

eFigure 65. Trends in Social Isolation for Paraguay by Income Group

eFigure 66. Trends in Social Isolation for Uruguay by Income Group

eFigure 67. Trends in Social Isolation for Peru by Income Group

eFigure 68. Trends in Social Isolation for El Salvador by Income Group

eFigure 69. Trends in Social Isolation for Chile by Income Group

eFigure 70. Trends in Social Isolation for Belize by Income Group

eFigure 71. Trends in Social Isolation for Trinidad and Tobago by Income Group

eFigure 72. Trends in Social Isolation for Puerto Rico by Income Group

eFigure 73. Trends in Social Isolation for Jordan by Income Group

eFigure 74. Trends in Social Isolation for Syria by Income Group

eFigure 75. Trends in Social Isolation for Lebanon by Income Group

eFigure 76. Trends in Social Isolation for Iraq by Income Group

eFigure 77. Trends in Social Isolation for Morocco by Income Group

© 2025 Fuller-Rowell TE et al. *JAMA Network Open.*

eFigure 78. Trends in Social Isolation for Libya by Income Group

eFigure 79. Trends in Social Isolation for Bahrain by Income Group

eFigure 80. Trends in Social Isolation for the United Arab Emirates by Income Group

eFigure 81. Trends in Social Isolation for Egypt by Income Group

eFigure 82. Trends in Social Isolation for Kuwait by Income Group

eFigure 83. Trends in Social Isolation for Saudi Arabia by Income Group

eFigure 84. Trends in Social Isolation for Tunisia by Income Group

eFigure 85. Trends in Social Isolation for Israel by Income Group

eFigure 86. Trends in Social Isolation for Qatar by Income Group

eFigure 87. Trends in Social Isolation for Türkiye by Income Group

eFigure 88. Trends in Social Isolation for the State of Palestine by Income Group

eFigure 89. Trends in Social Isolation for Algeria by Income Group

eFigure 90. Trends in Social Isolation for Iran by Income Group

eFigure 91. Trends in Social Isolation for Yemen by Income Group

eFigure 92. Trends in Social Isolation for Canada by Income Group

eFigure 93. Trends in Social Isolation for the United States of America by Income Group

eFigure 94. Trends in Social Isolation for New Zealand by Income Group

eFigure 95. Trends in Social Isolation for Australia by Income Group

eFigure 96. Trends in Social Isolation for Thailand by Income Group

eFigure 97. Trends in Social Isolation for Malaysia by Income Group

eFigure 98. Trends in Social Isolation for Indonesia by Income Group

eFigure 99. Trends in Social Isolation for Laos by Income Group

eFigure 100. Trends in Social Isolation for Vietnam by Income Group

eFigure 101. Trends in Social Isolation for Cambodia by Income Group

eFigure 102. Trends in Social Isolation for Singapore by Income Group

eFigure 103. Trends in Social Isolation for Myanmar by Income Group

eFigure 104. Trends in Social Isolation for the Philippines by Income Group

eFigure 105. Trends in Social Isolation for Albania by Income Group

© 2025 Fuller-Rowell TE et al. *JAMA Network Open.*

eFigure 106. Trends in Social Isolation for Luxembourg by Income Group

eFigure 107. Trends in Social Isolation for Austria by Income Group

eFigure 108. Trends in Social Isolation for Ireland by Income Group

eFigure 109. Trends in Social Isolation for Germany by Income Group

eFigure 110. Trends in Social Isolation for Belgium by Income Group

eFigure 111. Trends in Social Isolation for Denmark by Income Group

eFigure 112. Trends in Social Isolation for the United Kingdom of Great Britain and Northern Ireland

eFigure 113. Trends in Social Isolation for Poland by Income Group

eFigure 114. Trends in Social Isolation for Norway by Income Group

eFigure 115. Trends in Social Isolation for Greece by Income Group

eFigure 116. Trends in Social Isolation for Italy by Income Group

eFigure 117. Trends in Social Isolation for the Netherlands by Income Group

eFigure 118. Trends in Social Isolation for Sweden by Income Group

eFigure 119. Trends in Social Isolation for Malta by Income Group

eFigure 120. Trends in Social Isolation for the Republic of Cyprus by Income Group

eFigure 121. Trends in Social Isolation for Spain by Income Group

eFigure 122. Trends in Social Isolation for Iceland by Income Group

eFigure 123. Trends in Social Isolation for Switzerland by Income Group

eFigure 124. Trends in Social Isolation for Portugal by Income Group

eFigure 125. Trends in Social Isolation for the Czech Republic by Income Group

eFigure 126. Trends in Social Isolation for Slovenia by Income Group

eFigure 127. Trends in Social Isolation for Estonia by Income Group

eFigure 128. Trends in Social Isolation for France by Income Group

eFigure 129. Trends in Social Isolation for Lithuania by Income Group

eFigure 130. Trends in Social Isolation for North Macedonia by Income Group

eFigure 131. Trends in Social Isolation for Montenegro by Income Group

eFigure 132. Trends in Social Isolation for Finland by Income Group

eFigure 133. Trends in Social Isolation for Slovakia by Income Group

© 2025 Fuller-Rowell TE et al. *JAMA Network Open.*

eFigure 134. Trends in Social Isolation for Croatia by Income Group

eFigure 135. Trends in Social Isolation for Serbia by Income Group

eFigure 136. Trends in Social Isolation for Latvia by Income Group

eFigure 137. Trends in Social Isolation for Romania by Income Group

eFigure 138. Trends in Social Isolation for Hungary by Income Group

eFigure 139. Trends in Social Isolation for Bulgaria by Income Group

eFigure 140. Trends in Social Isolation for Kosovo by Income Group

eFigure 141. Trends in Social Isolation for Bosnia Herzegovina by Income Group

eFigure 142. Trends in Social Isolation for Hong Kong by Income Group

eFigure 143. Trends in Social Isolation for Japan by Income Group

eFigure 144. Trends in Social Isolation for South Korea by Income Group

eFigure 145. Trends in Social Isolation for Mongolia by Income Group

eFigure 146. Trends in Social Isolation for Taiwan by Income Group

eFigure 147. Trends in Social Isolation for China by Income Group

eFigure 148. Trends in Social Isolation for Azerbizan by Income Group

eFigure 149. Trends in Social Isolation for Russia by Income Group

eFigure 150. Trends in Social Isolation for Uzbekistan by Income Group

eFigure 151. Trends in Social Isolation for Moldova by Income Group

eFigure 152. Trends in Social Isolation for Ukraine by Income Group

eFigure 153. Trends in Social Isolation for Turkmenistan by Income Group

eFigure 154. Trends in Social Isolation for Belarus by Income Group

eFigure 155. Trends in Social Isolation for Kyrgyzstan by Income Group

eFigure 156. Trends in Social Isolation for Kazakhstan by Income Group

eFigure 157. Trends in Social Isolation for Armenia by Income Group

eFigure 158. Trends in Social Isolation for Tajikistan by Income Group

eFigure 159. Trends in Social Isolation for Georgia by Income Group

eFigure 160. Trends in Social Isolation for Sub-Saharan Africa by Income Group

eFigure 161. Trends in Social Isolation for South Asia by Income Group

eFigure 162. Trends in Social Isolation for Latin America and the Caribbean (LAC) by Income Group

eFigure 163. Trends in Social Isolation for Middle East and North Africa (MENA) by Income Group

eFigure 164. Trends in Social Isolation for North America by Income Group

eFigure 165. Trends in Social Isolation for ANZ (Australia and New Zealand) by Income Group

eFigure 166. Trends in Social Isolation for Southeast Asia by Income Group

eFigure 167. Trends in Social Isolation for Europe by Income Group

eFigure 168. Trends in Social Isolation for East Asia by Income Group

eFigure 169. Trends in Social Isolation for RFSU (Russia and the Former Soviet Union) by Income Group

eTable 1. Raw Global Descriptive Statistics for Social Isolation at Each Timepoint

eTable 2. Comparison of Fixed-Effect Estimates From the Weighted and Unweighted Models

eTable 3. Comparing Fixed-Effect Estimates From the Unrestricted and Diagonal G-Matrix Models

eTable 4. Comparison of Model Fit Statistics for Alternative Trajectory Structures

eTable 5. Multilevel Discontinuous Growth Curve Parameter Estimates for Full Sample, Low-Income (Bottom 40%), and High-Income (Top 40%) Group

eTable 6. Country Rankings on Changes in Social Isolation Trajectories (2009–2024)

eTable 7. Regional Rankings from Best to Worst for Social Isolation Trajectories (2009-2024)

eTable 8. Country Rankings Within Each Region on Changes in Social Isolation Trajectories (2009-2024)

eTable 9. Global Summary of Social Isolation Trends by Trend Type

eTable 10. Country Rankings for Social Isolation Levels and Disparities in 2024 (Best to Worst)

eTable 11. Regional Rankings for Social Isolation Levels and Disparities in 2024 (Best to Worst)

eTable 12. Country Ranking Within Each Region on Social Isolation Trajectories in 2024

© 2025 Fuller-Rowell TE et al. *JAMA Network Open.*

This supplementary material has been provided by the authors to give readers additional information about their work.

© 2025 Fuller-Rowell TE et al. *JAMA Network Open.*

eMethods

Earlier timepoints (2005–2008) were excluded because they did not have income assessments and contained a smaller number of countries. Eight of the 167 countries in the database were excluded because they did not have more than one time-point of data or did not have income assessments.

Analysis Strategy: Equations

Three growth parameters, shown in the equations below were considered to characterize the time trend:

Level 1: $Y_{jk} = \beta_{00k} + \beta_{10k}$*Linear $\text{Slope}_{jk} + \beta_{20k}$*$\text{Step-Change}_{jk} + \beta_{30k}$*Slope $\text{Change}_{jk} + e_{jk}$

Level 2: $\beta_{00k} = \gamma_{00} + u_{00k}$

$\beta_{10k} = \gamma_{10} + u_{10k}$

$\beta_{20k} = \gamma_{20} + u_{20k}$

$\beta_{30k} = \gamma_{30} + u_{30k}$

Random effects were estimated for the intercept (u_{00k}) and all slope parameters ($u_{10k} – u_{30k}$).

© 2025 Fuller-Rowell TE et al. *JAMA Network Open.*

eResults

In an "empty model" with no predictors, the global mean level of social isolation across all countries and time points was 19.20 (95% CI: 17.28–21.16, $p < .001$). The intraclass correlation coefficient indicated that 90% of the variance in social isolation existed between countries, and 10% was within countries over time.

Detailed Description of Results from the Final Best Fitting Model

In 2009, the global mean level was 19.22 (17.28–21.16, $p < .001$), and the gap between the top and bottom income groups was 8.78 (5.73–11.83, $p < .001$). In relative terms, the bottom income group had 1.57 times higher levels of isolation than the top income group (24.07% socially isolated [21.63–26.51]) vs. 15.29% [13.46–17.11]). The mean change in social isolation from 2009 to 2024 was 2.58 units (0.69–4.47), a 13.42% increase from 19.22% (17.28–21.16) to 21.80% (19.40–24.20) socially isolated, equivalent to .22 *SD* units. By 2024, the income gap in social isolation was 8.61 percentage points (5.13–12.08), with 26.19% (23.54–28.83) of lower-income individuals reporting isolation, compared to 17.58% (15.33–19.83) of higher-income individuals. The magnitude of this difference between high- and low-income groups was .72 SD units.

Prior to the COVID-19 pandemic (2009–2019), global social isolation levels remained stable (linear slope: B = 0.35, 95% CI: -1.33–2.04, $p = 0.681$, a 1.82% increase). However, a significant disruption occurred between 2019 and 2020, with social isolation levels increasing sharply (step-change: $B = 1.50$ [0.30–2.70], $p = 0.014$, 7.66% increase, equivalent to .13 SD units). This increase was driven by lower-income groups (B = 2.62 [0.86–4.38], P = .003, 11.01% increase, equivalent to .22 SD units), with higher-income groups showing relative stability (step-change: $B = 0.48$ [-0.52–1.48], $p = .347$, 3.17% increase).

© 2025 Fuller-Rowell TE et al. *JAMA Network Open.*

Between 2020 and 2024, global increases in isolation remained stable (linear slope: B = 0.72 [-0.52–1.96], P = .252, 3.42% increase), with the increase among the higher-income group (linear slope: B = 1.93 [0.72–3.14]; P = .001, 12.33% increase), and with relative stability among lower-income groups (linear slope: B = -0.21 [-1.86–1.43], P = .799, 0.80% decrease). These trends meant that the income disparity in social isolation was largest in 2020 (B = 10.75 [7.30–14.20]; P < .001, a 22.43% increase from 2009), and by 2024 were 1.93% lower than in 2009 (B = 8.78 [5.73–11.83]; P < .001). This was due to larger increases in isolation for the top income group between 2020 and 2024.

A small positive correlation was observed between absolute change in mean levels and disparities ($r = 0.30$, $p < .001$). However, the correlation between relative change in mean levels and disparities was not significant ($r = -0.19$, $p = .809$).

Country and region trajectories can be grouped into the categories: (1) improving trends, defined as a decrease (≥ 1 unit) in mean levels and a decrease (≥ 1 unit) in disparities; (2) partly improving trends, defined as a decrease in mean levels and no clear change (< 1 unit) in disparities, or a decrease in disparities and no clear change in mean levels; (3) problematic trends, defined as an increase in mean levels and increase in disparities; (4) partly problematic trends, defined as an increase in mean levels and no clear change in disparities, or an increase in disparities and no clear change in mean levels; (5) consistent trend, defined as no clear change in mean levels or disparities; and (6) opposite trends, defined as an increase in disparities and a decrease in mean levels, or an increase in mean levels and a decrease in disparities. Countries within each category are shown in eTable 9.

2024 Levels and Disparities

© 2025 Fuller-Rowell TE et al. *JAMA Network Open.*

Rankings were generated for the trajectory end point (2024) levels, disparities, and combined score of both for each region and country (eTable 10 and eTable 11). With respect to world regions, North America (NAM), and Australia and New Zealand (ANZ) had the lowest mean levels (8.98% and 9.00%, respectively), and Australia and New Zealand (ANZ) and Europe had smallest disparities (3.66% and 5.47%), whereas South Asia and SSA had the highest mean levels (39.95% and 35.74%) and largest disparities (13.86% and 13.0%). With respect to countries, Iceland and Denmark ranked the highest, with very low mean levels of isolation (1.3% and 4.4%, respectively) and disparities smaller than 1%. Central African Republic and Afghanistan ranked the lowest with very high levels of isolation (79.53% and 71.87%), and very high disparities (8.84% and 19.22%).

© 2025 Fuller-Rowell TE et al. *JAMA Network Open.*

eFigure 1. Trends in Social Isolation for Tanzania by Income Group. Fitted trajectories are derived from empirical Bayes estimates of the final best fitting model, with raw data overlaid.

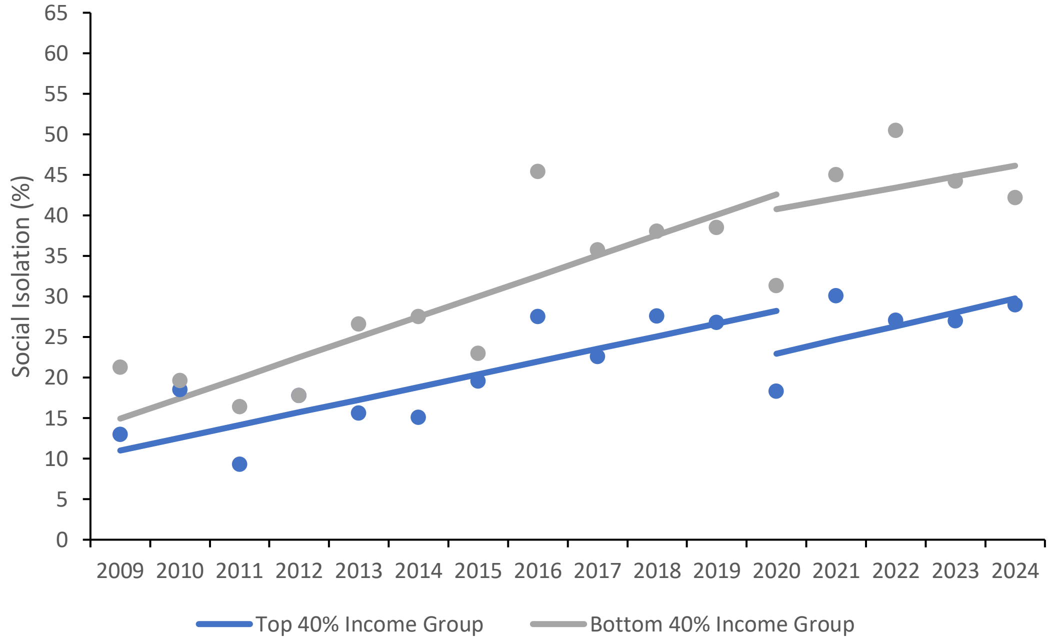

© 2025 Fuller-Rowell TE et al. *JAMA Network Open.*

eFigure 2. Trends in Social Isolation for Congo (Kinshasa) by Income Group. Fitted trajectories are derived from empirical Bayes estimates of the final best fitting model, with raw data overlaid.

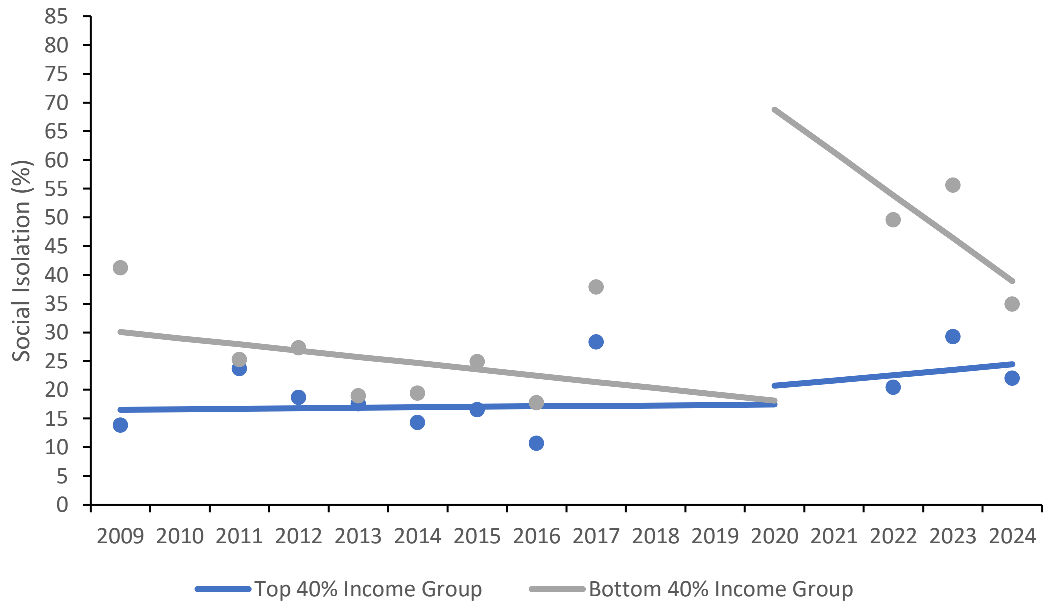

© 2025 Fuller-Rowell TE et al. *JAMA Network Open.*

eFigure 3. Trends in Social Isolation for Central African Republic by Income Group. Fitted trajectories are derived from empirical Bayes estimates of the final best fitting model, with raw data overlaid.

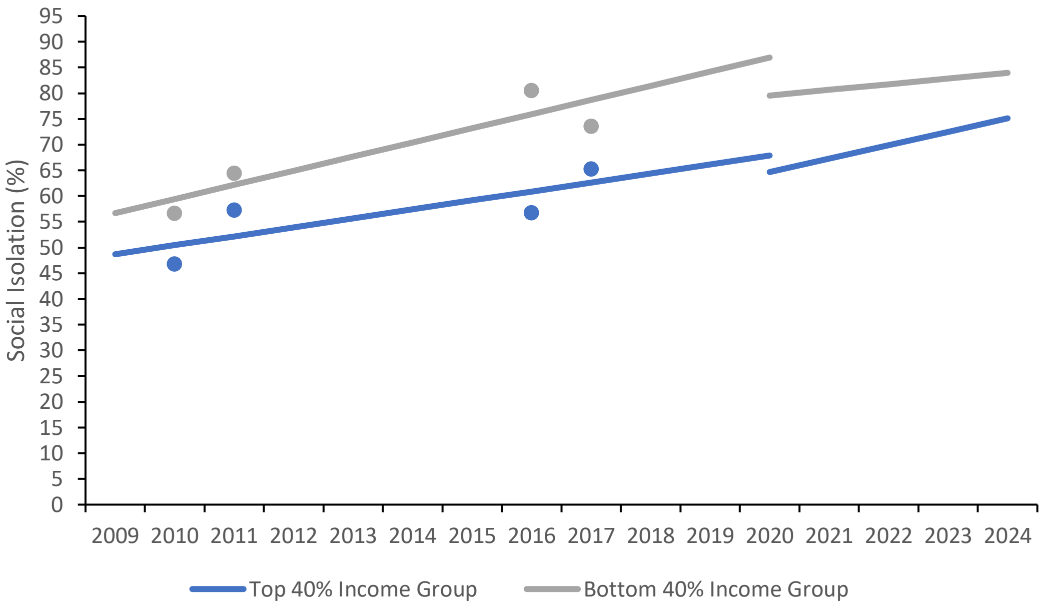

© 2025 Fuller-Rowell TE et al. *JAMA Network Open.*

eFigure 4. Trends in Social Isolation for Kenya by Income Group. Fitted trajectories are derived from empirical Bayes estimates of the final best fitting model, with raw data overlaid.

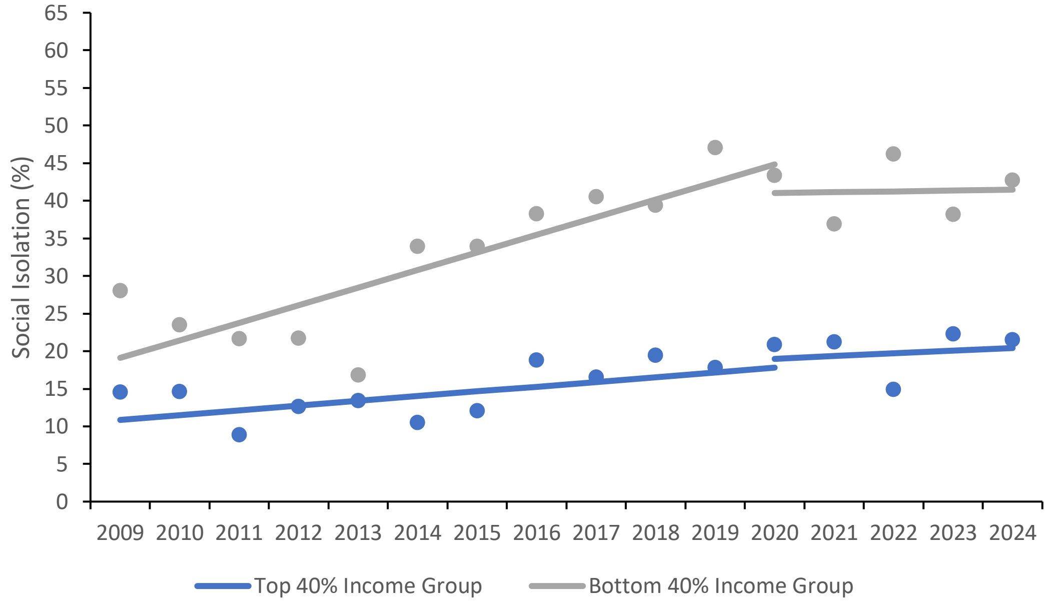

© 2025 Fuller-Rowell TE et al. *JAMA Network Open.*

eFigure 5. Trends in Social Isolation for Zimbabwe by Income Group. Fitted trajectories are derived from empirical Bayes estimates of the final best fitting model, with raw data overlaid.

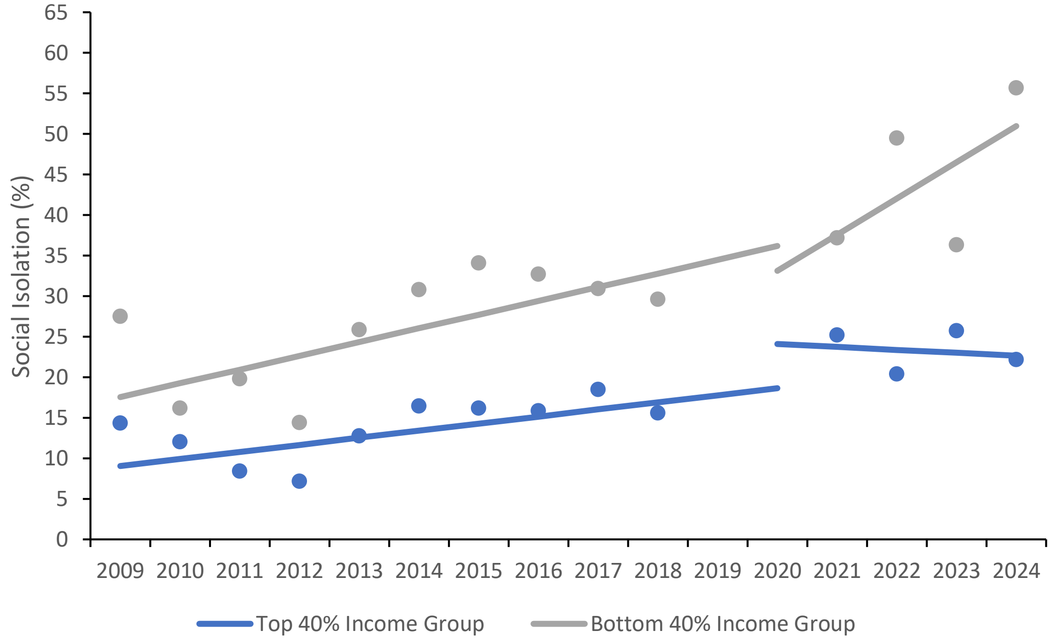

© 2025 Fuller-Rowell TE et al. *JAMA Network Open.*

eFigure 6. Trends in Social Isolation for Botswana by Income Group. Fitted trajectories are derived from empirical Bayes estimates of the final best fitting model, with raw data overlaid.

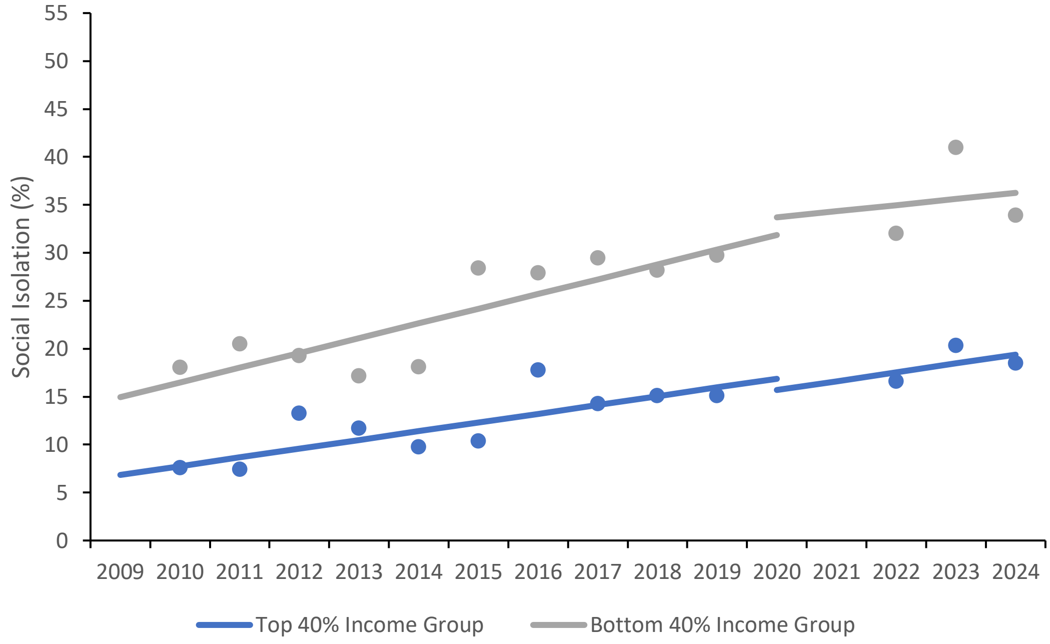

© 2025 Fuller-Rowell TE et al. *JAMA Network Open.*

eFigure 7. Trends in Social Isolation for Lesotho by Income Group. Fitted trajectories are derived from empirical Bayes estimates of the final best fitting model, with raw data overlaid.

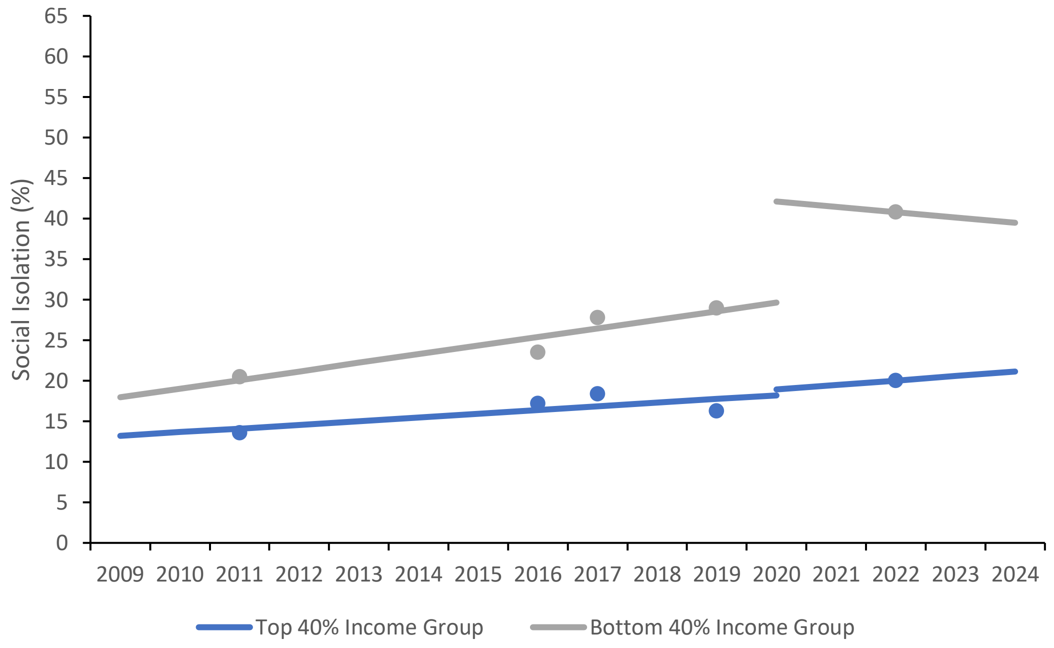

© 2025 Fuller-Rowell TE et al. *JAMA Network Open.*

eFigure 8. Trends in Social Isolation for Mali by Income Group. Fitted trajectories are derived from empirical Bayes estimates of the final best fitting model, with raw data overlaid

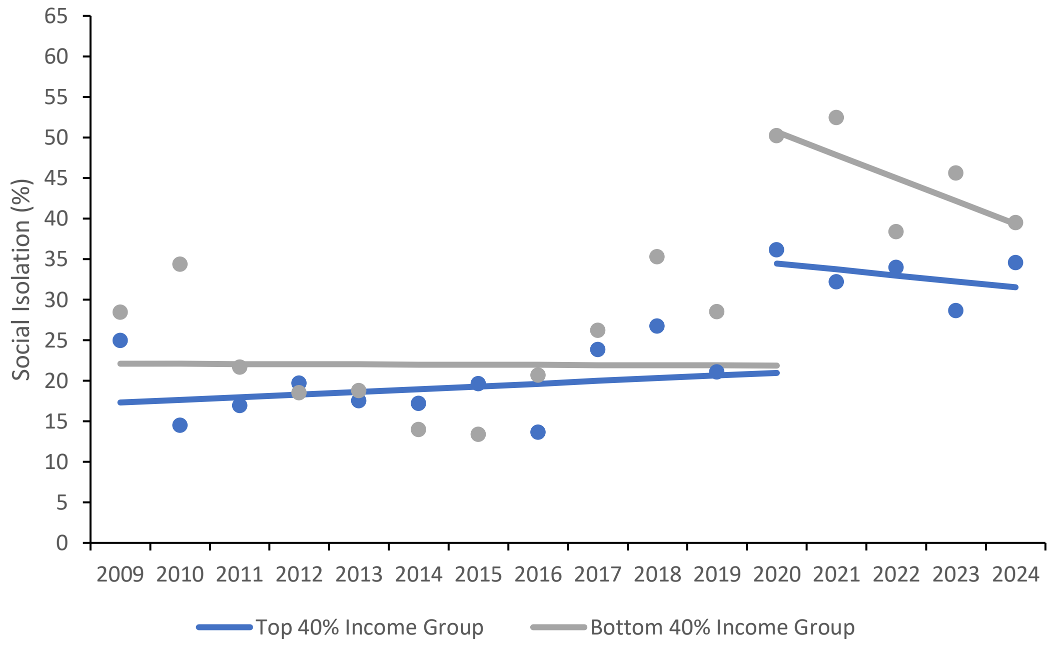

© 2025 Fuller-Rowell TE et al. *JAMA Network Open.*

eFigure 9. Trends in Social Isolation for Côte d'Ivoire by Income Group. Fitted trajectories are derived from empirical Bayes estimates estimates of the final best fitting model, with raw data overlaid

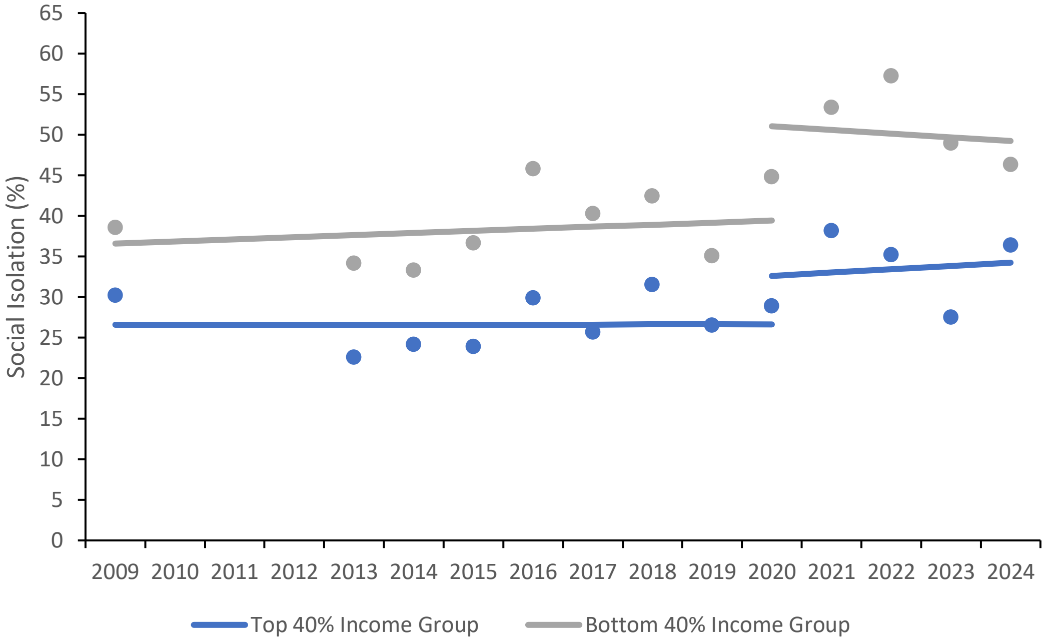

© 2025 Fuller-Rowell TE et al. *JAMA Network Open.*

eFigure 10. Trends in Social Isolation for Sudan by Income Group. Fitted trajectories are derived from empirical Bayes estimates of the final best fitting model, with raw data overlaid

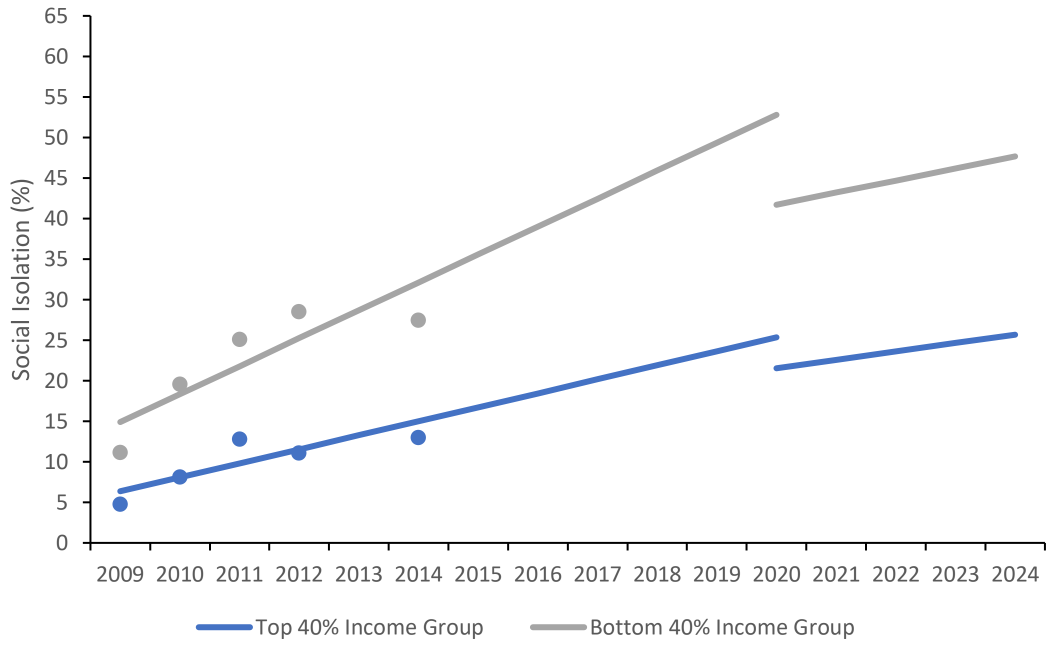

© 2025 Fuller-Rowell TE et al. *JAMA Network Open.*

eFigure 11. Trends in Social Isolation for Rwanda by Income Group. Fitted trajectories are derived from empirical Bayes estimates of the final best fitting model, with raw data overlaid

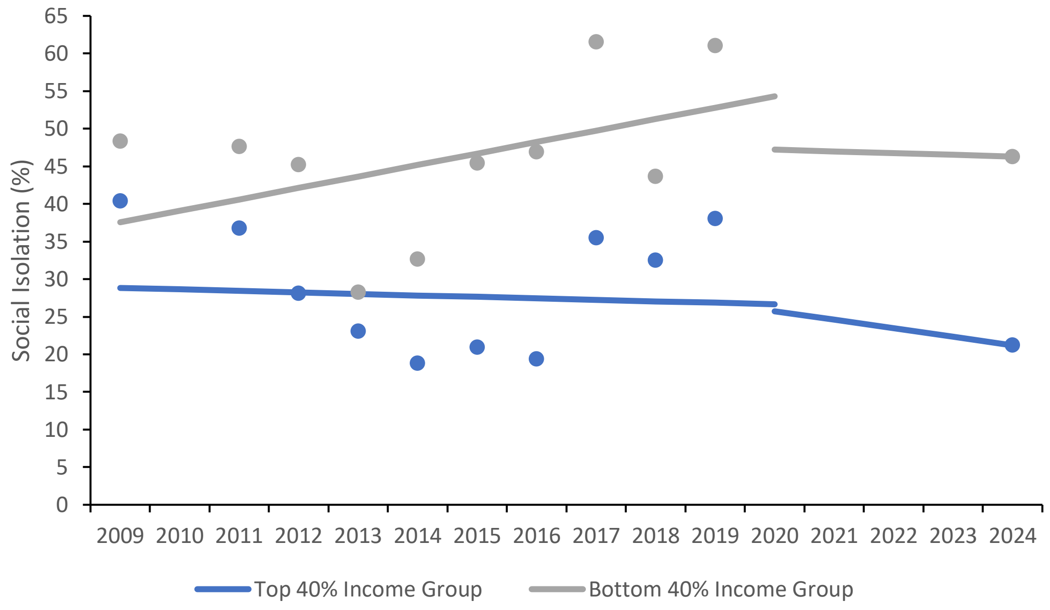

© 2025 Fuller-Rowell TE et al. *JAMA Network Open.*

eFigure 12. Trends in Social Isolation for Zambia by Income Group. Fitted trajectories are derived from empirical Bayes estimates of the final best fitting model, with raw data overlaid

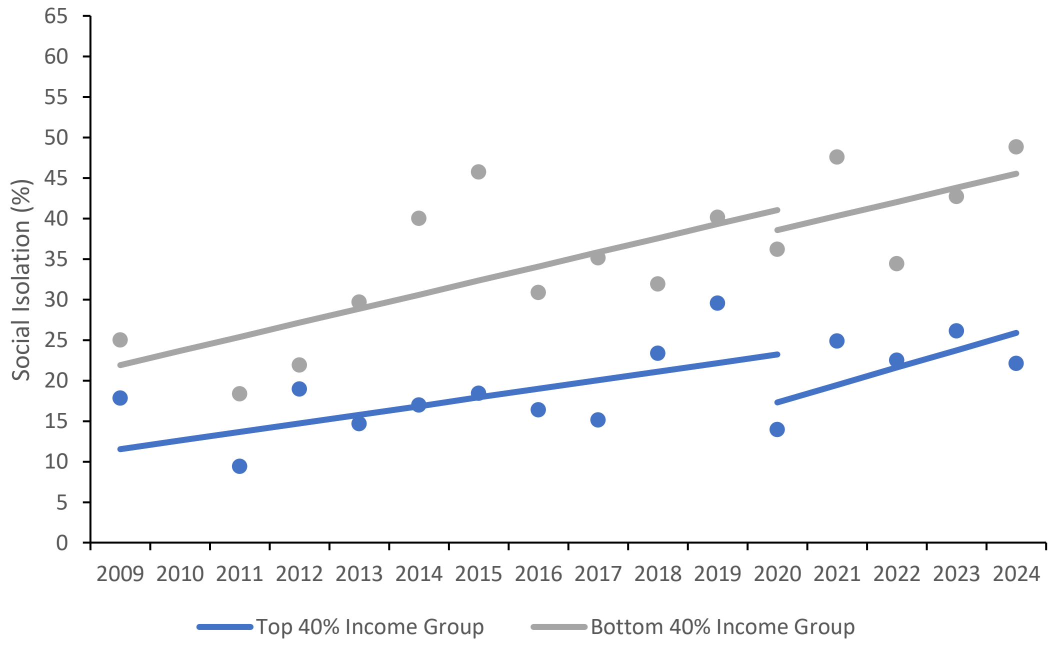

© 2025 Fuller-Rowell TE et al. *JAMA Network Open.*

eFigure 13. Trends in Social Isolation for Liberia by Income Group. Fitted trajectories are derived from empirical Bayes estimates of the final best fitting model, with raw data overlaid.

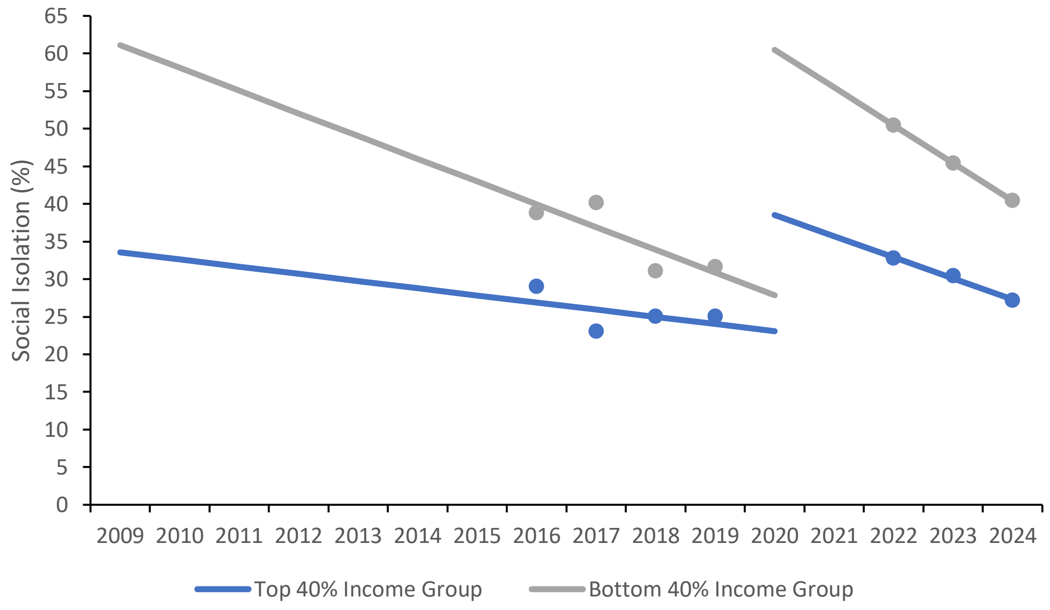

© 2025 Fuller-Rowell TE et al. *JAMA Network Open.*

eFigure 14. Trends in Social Isolation for Mauritania by Income Group. Fitted trajectories are derived from empirical Bayes estimates of the final best fitting model, with raw data overlaid

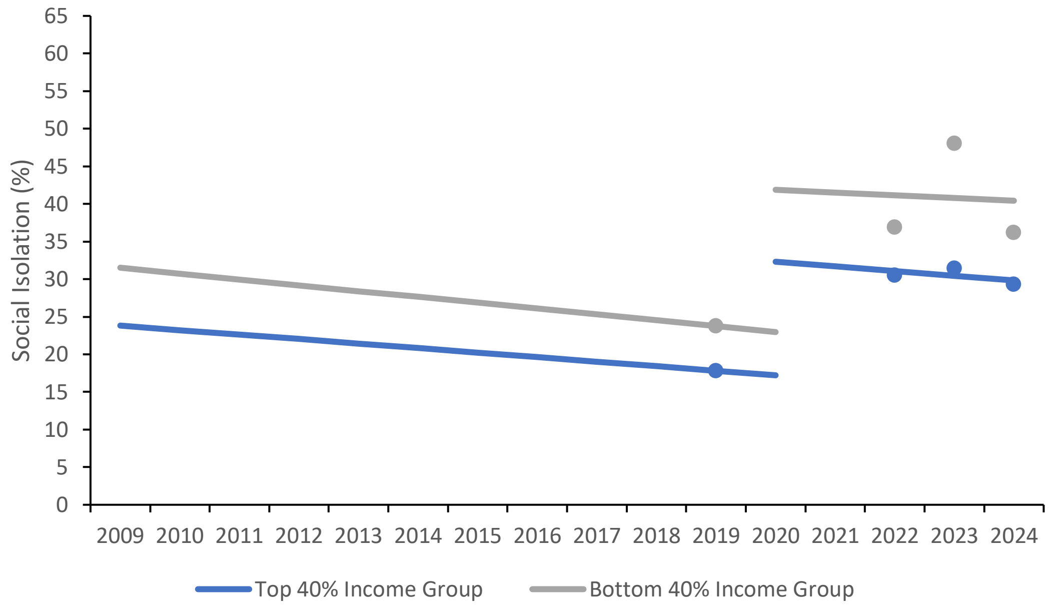

© 2025 Fuller-Rowell TE et al. *JAMA Network Open.*

eFigure 15. Trends in Social Isolation for Comoros by Income Group. Fitted trajectories are derived from empirical Bayes estimates of the final best fitting model, with raw data overlaid

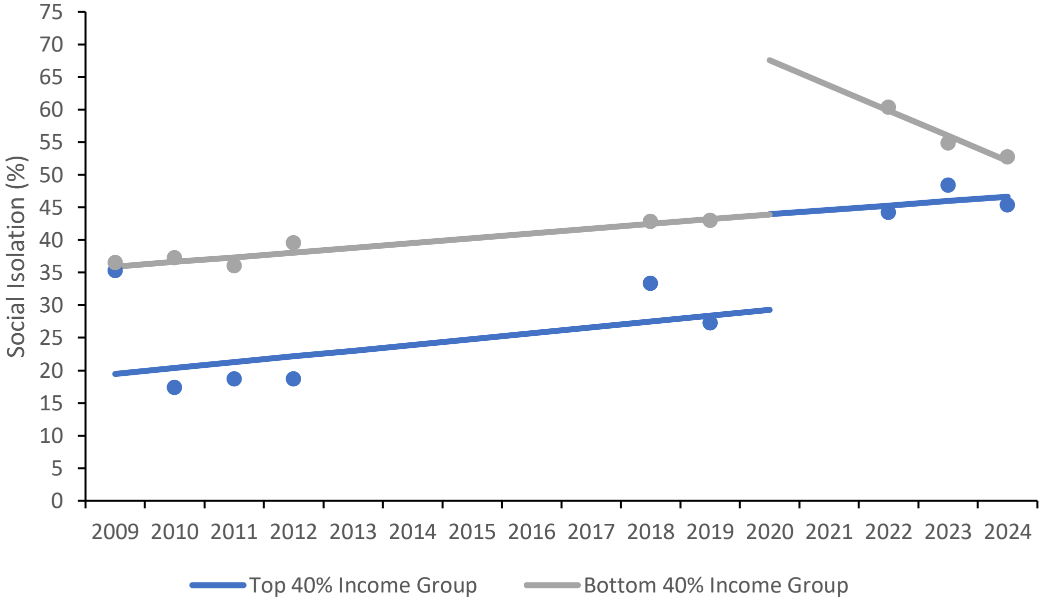

© 2025 Fuller-Rowell TE et al. *JAMA Network Open.*

eFigure 16. Trends in Social Isolation for Burkina Faso by Income Group. Fitted trajectories are derived from empirical Bayes estimates of the final best fitting model, with raw data overlaid.

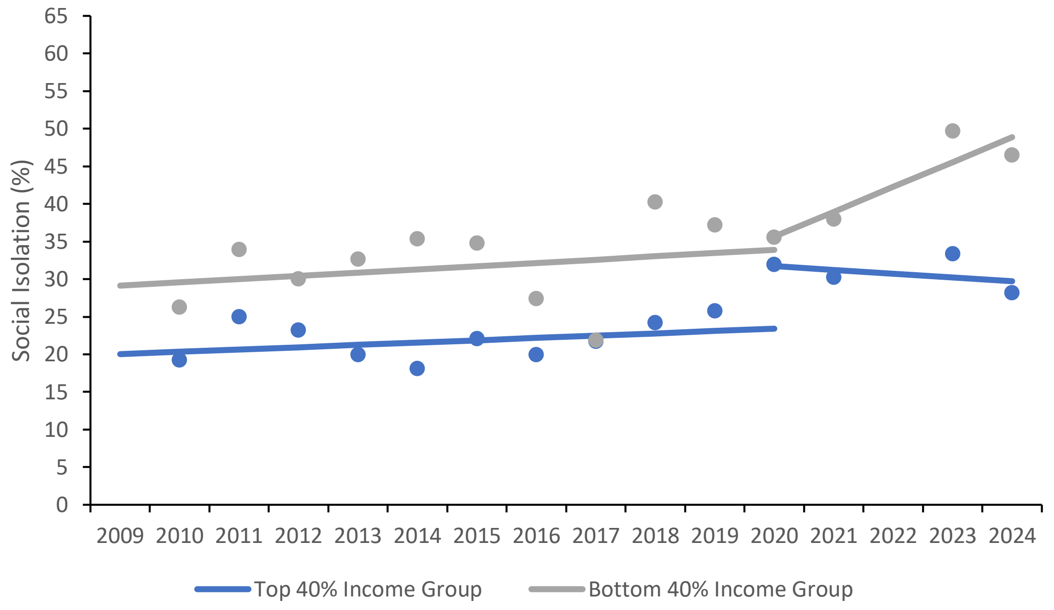

© 2025 Fuller-Rowell TE et al. *JAMA Network Open.*

eFigure 17. Trends in Social Isolation for Sierra Leone by Income Group. Fitted trajectories are derived from empirical Bayes estimates of the final best fitting model, with raw data overlaid.

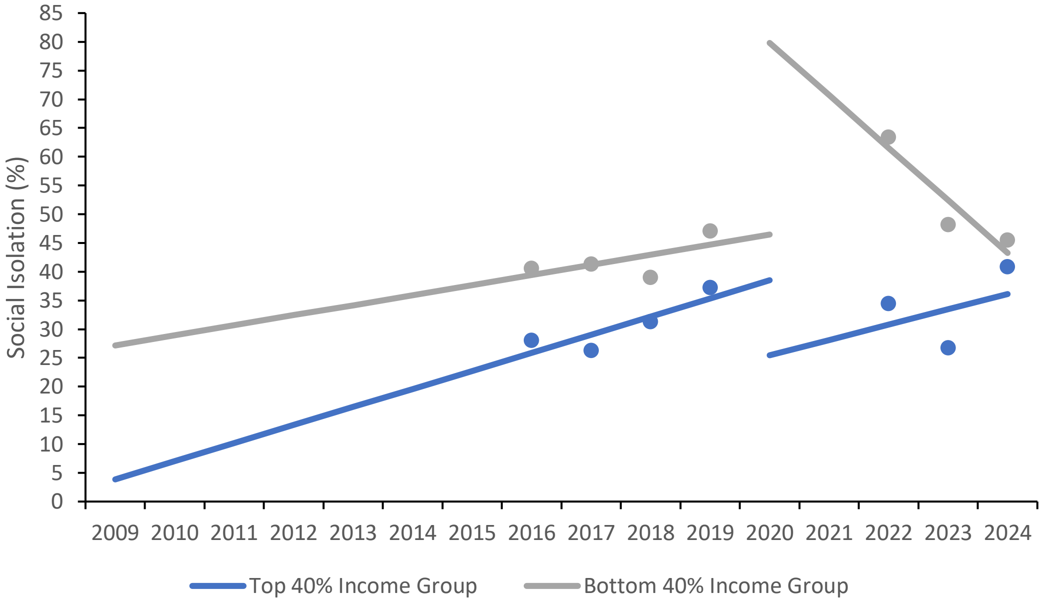

© 2025 Fuller-Rowell TE et al. *JAMA Network Open.*

eFigure 18. Trends in Social Isolation for Eswatini by Income Group. Fitted trajectories are derived from empirical Bayes estimates of the final best fitting model, with raw data overlaid.

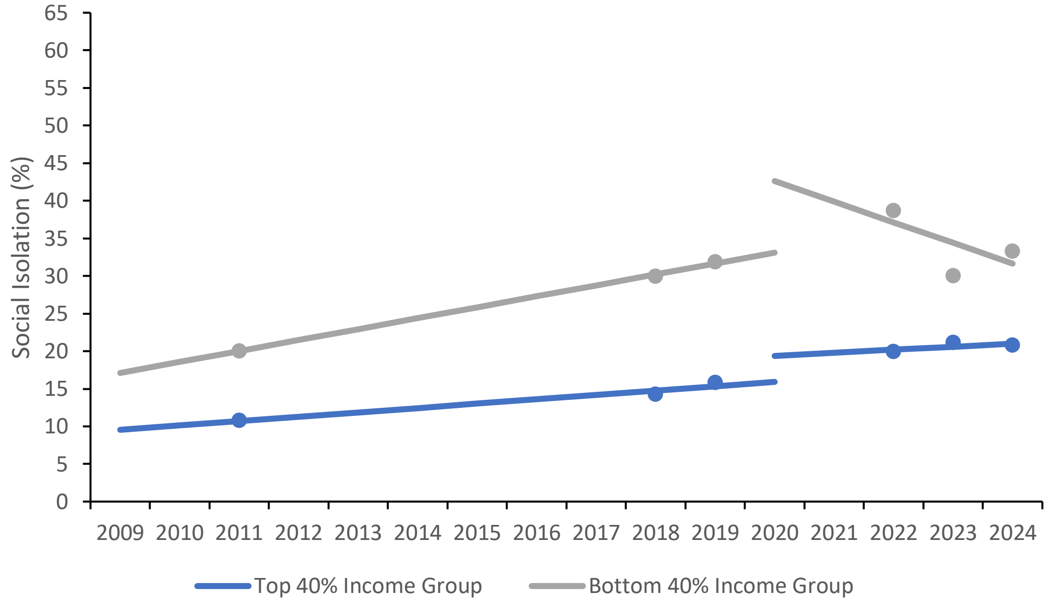

© 2025 Fuller-Rowell TE et al. *JAMA Network Open.*

eFigure 19. Trends in Social Isolation for South Sudan by Income Group. Fitted trajectories are derived from empirical Bayes estimates of the final best fitting model, with raw data overlaid.

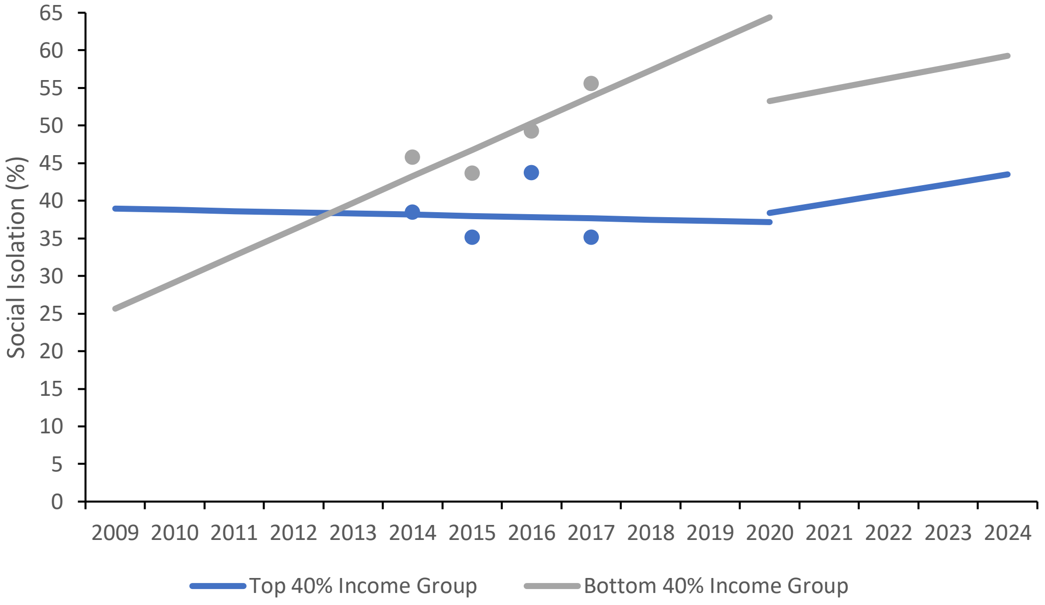

© 2025 Fuller-Rowell TE et al. *JAMA Network Open.*

eFigure 20. Trends in Social Isolation for Chad by Income Group. Fitted trajectories are derived from empirical Bayes estimates of the final best fitting model, with raw data overlaid.

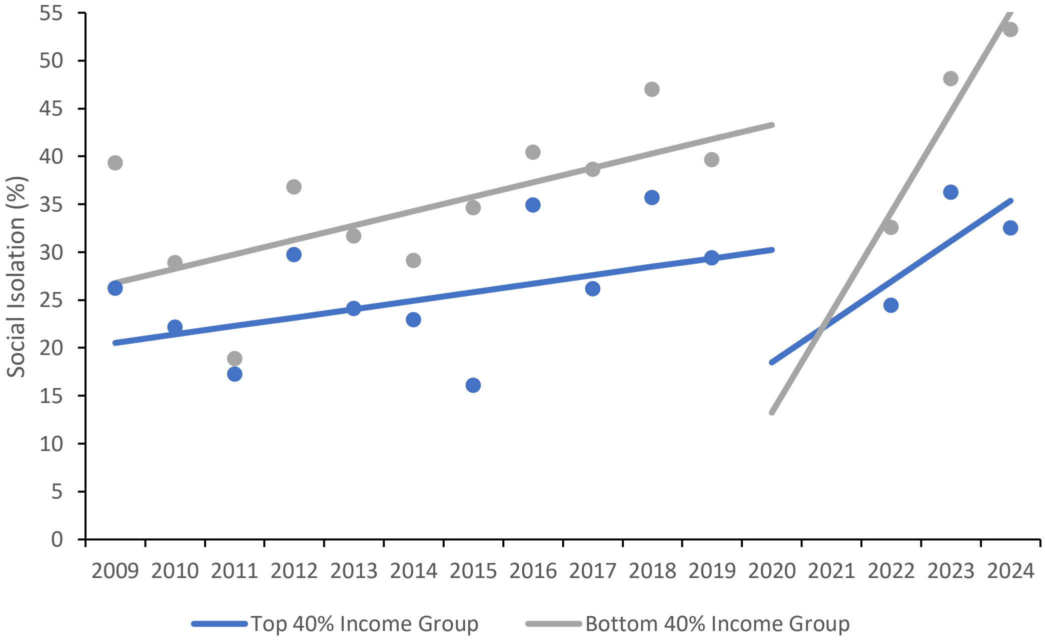

© 2025 Fuller-Rowell TE et al. *JAMA Network Open.*

eFigure 21. Trends in Social Isolation for Malawi by Income Group. Fitted trajectories are derived from empirical Bayes estimates of the final best fitting model, with raw data overlaid.

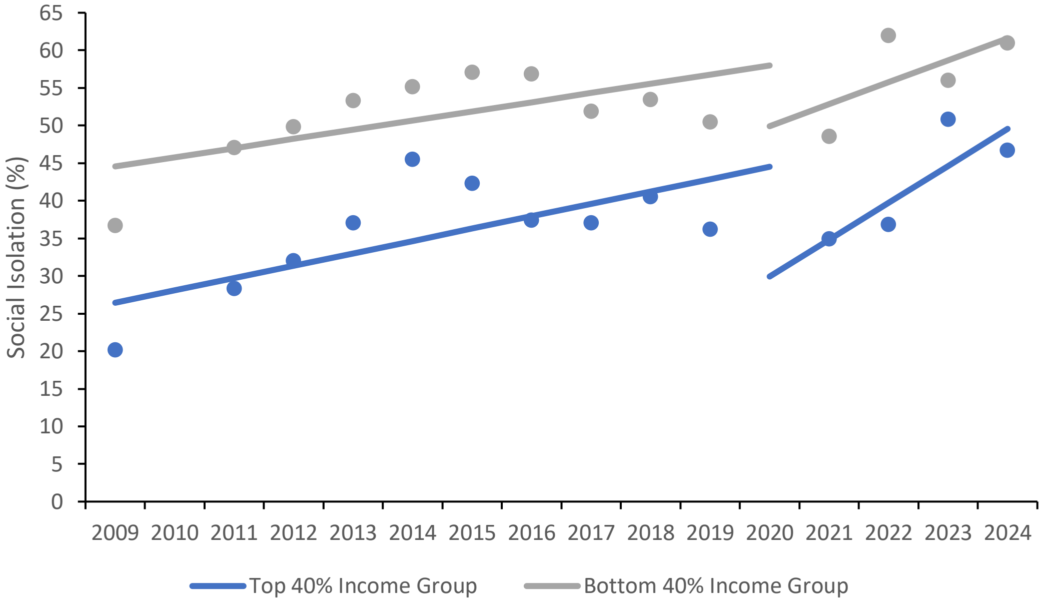

© 2025 Fuller-Rowell TE et al. *JAMA Network Open.*

eFigure 22. Trends in Social Isolation for Ghana by Income Group. Fitted trajectories are derived from empirical Bayes estimates of the final best fitting model, with raw data overlaid.

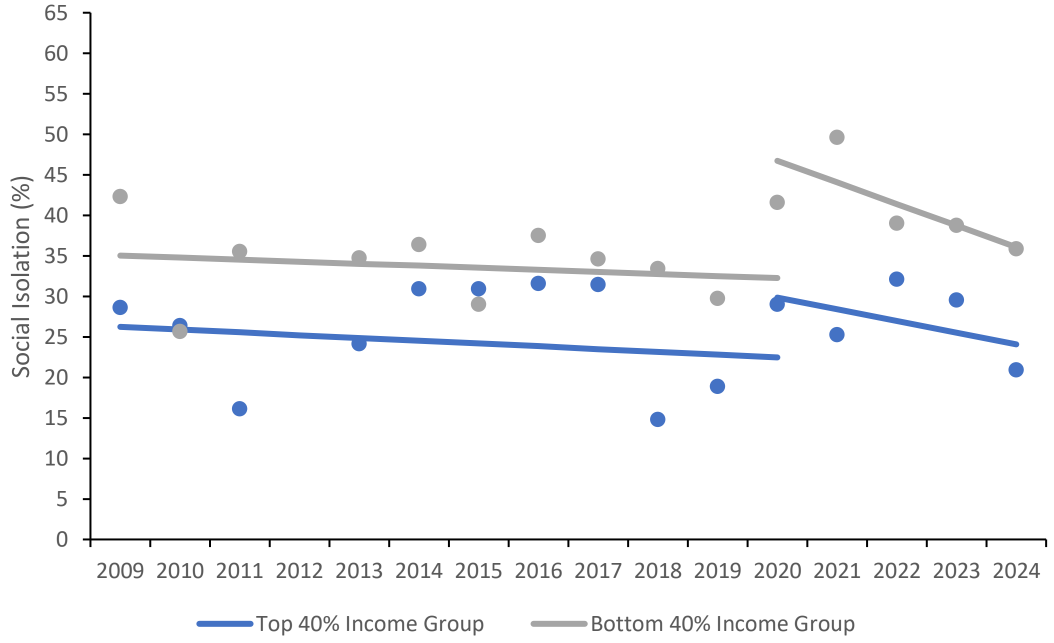

© 2025 Fuller-Rowell TE et al. *JAMA Network Open.*

eFigure 23. Trends in Social Isolation for Somalia by Income Group. Fitted trajectories are derived from empirical Bayes estimates of the final best fitting model, with raw data overlaid

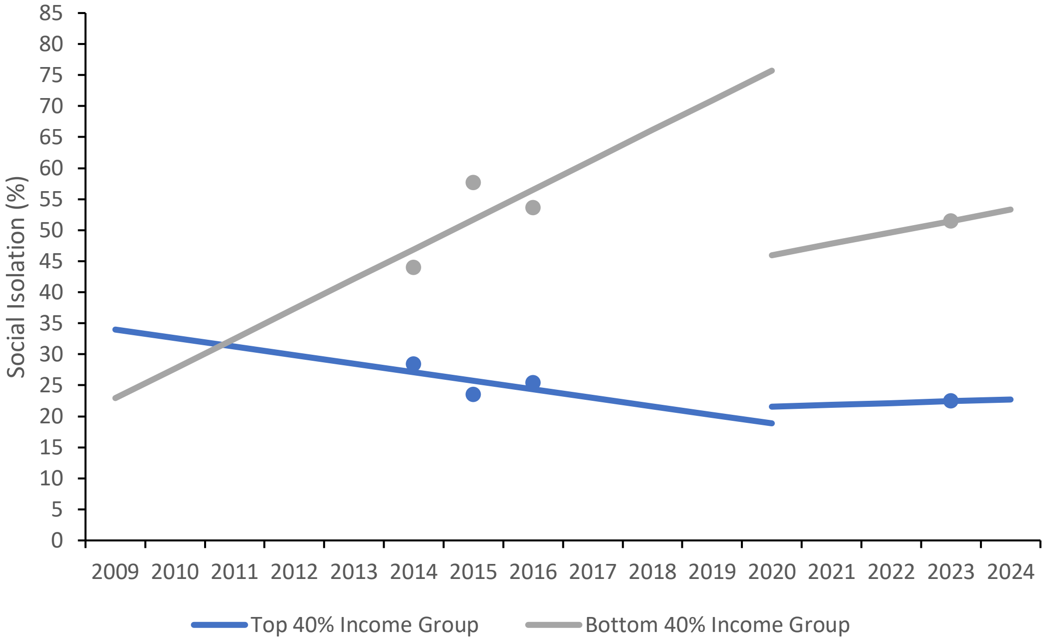

© 2025 Fuller-Rowell TE et al. *JAMA Network Open.*

eFigure 24. Trends in Social Isolation for Burundi by Income Group. Fitted trajectories are derived from empirical Bayes estimates of the final best fitting model, with raw data overlaid.

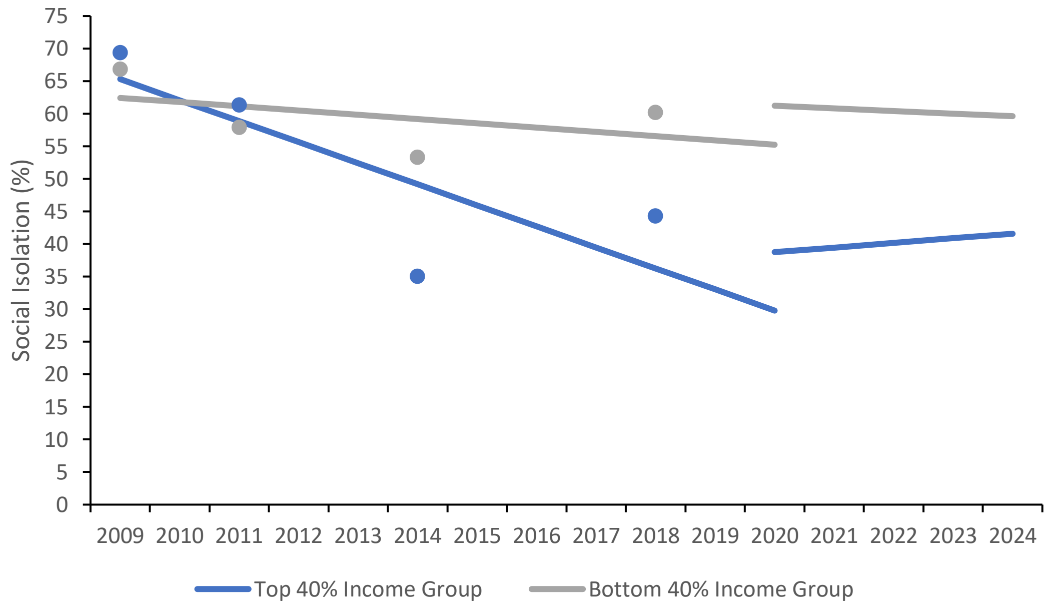

© 2025 Fuller-Rowell TE et al. *JAMA Network Open.*

eFigure 25. Trends in Social Isolation for Gambia by Income Group. Fitted trajectories are derived from empirical Bayes estimates of the final best fitting model, with raw data overlaid.

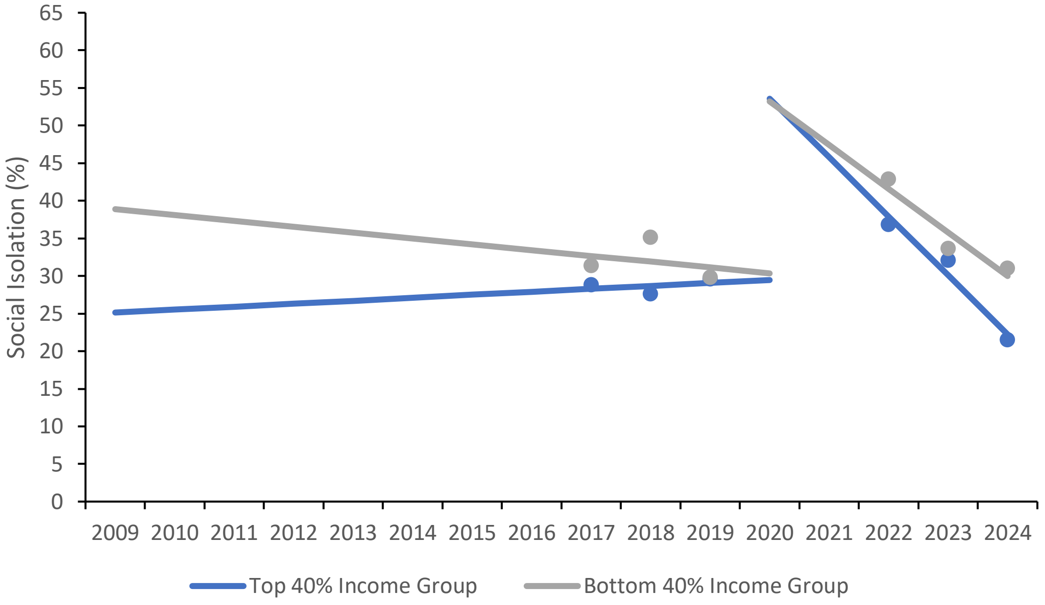

© 2025 Fuller-Rowell TE et al. *JAMA Network Open.*

eFigure 26. Trends in Social Isolation for Niger by Income Group. Fitted trajectories are derived from empirical Bayes estimates of the final best fitting model, with raw data overlaid.

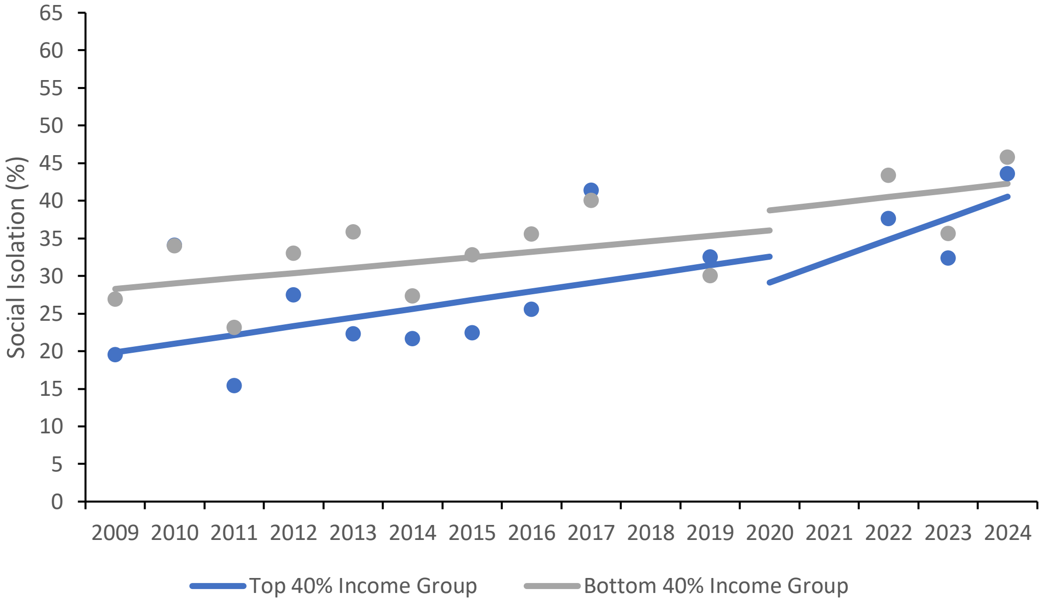

© 2025 Fuller-Rowell TE et al. *JAMA Network Open.*

eFigure 27. Trends in Social Isolation for Uganda by Income Group. Fitted trajectories are derived from empirical Bayes estimates of the final best fitting model, with raw data overlaid.

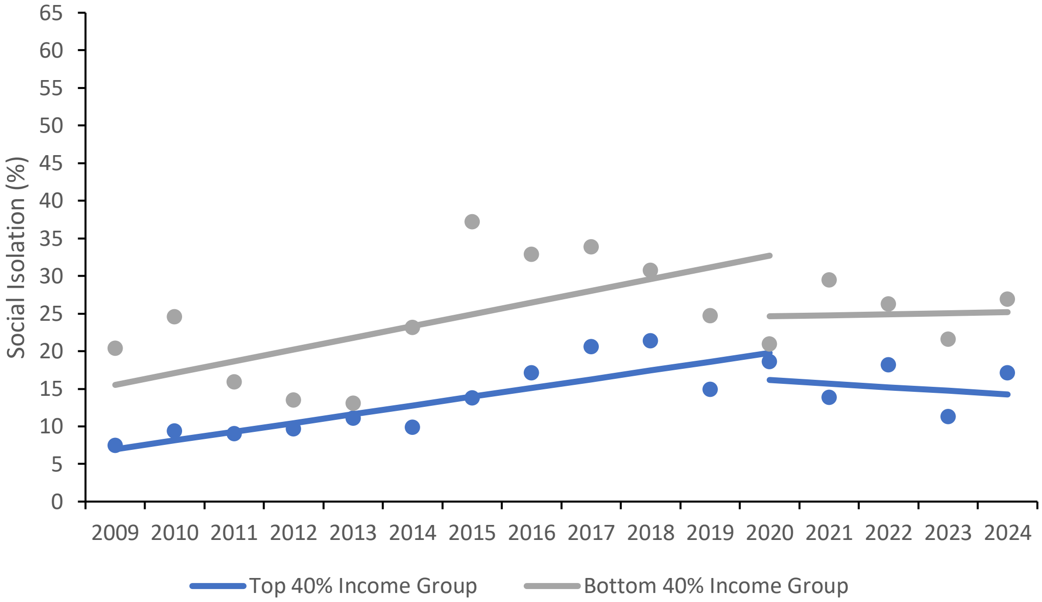

© 2025 Fuller-Rowell TE et al. *JAMA Network Open.*

eFigure 28. Trends in Social Isolation for Cameroon by Income Group. Fitted trajectories are derived from empirical Bayes estimates of the final best fitting model, with raw data overlaid.

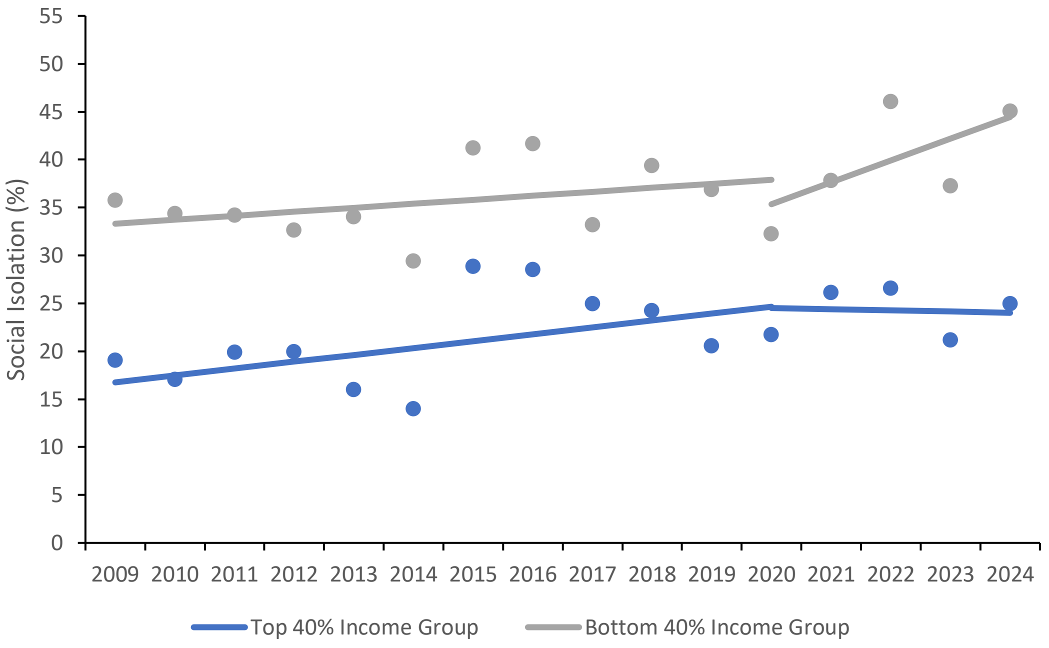

© 2025 Fuller-Rowell TE et al. *JAMA Network Open.*

eFigure 29. Trends in Social Isolation for Benin by Income Group. Fitted trajectories are derived from empirical Bayes estimates of the final best fitting model, with raw data overlaid.

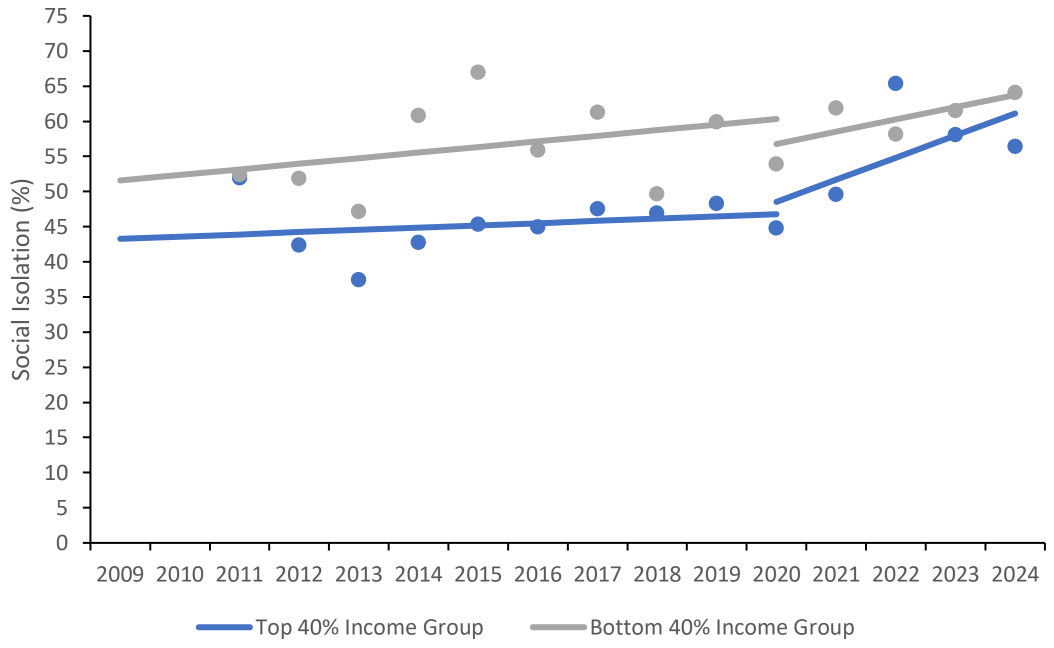

© 2025 Fuller-Rowell TE et al. *JAMA Network Open.*

eFigure 30. Trends in Social Isolation for Guinea by Income Group. Fitted trajectories are derived from empirical Bayes estimates of the final best fitting model, with raw data overlaid

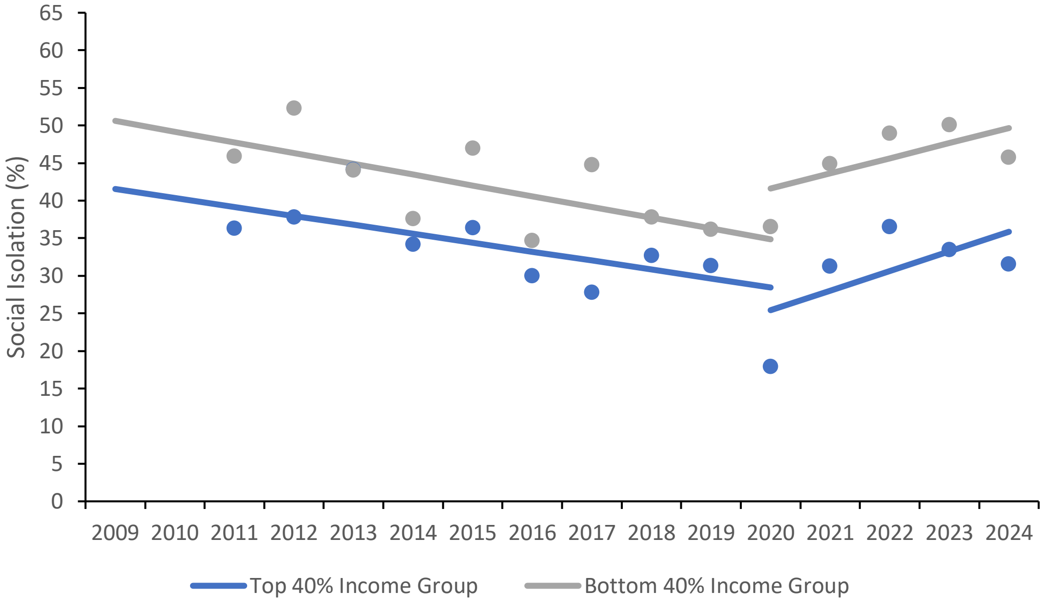

© 2025 Fuller-Rowell TE et al. *JAMA Network Open.*

eFigure 31. Trends in Social Isolation for Senegal by Income Group. Fitted trajectories are derived from empirical Bayes estimates of the final best fitting model, with raw data overlaid.

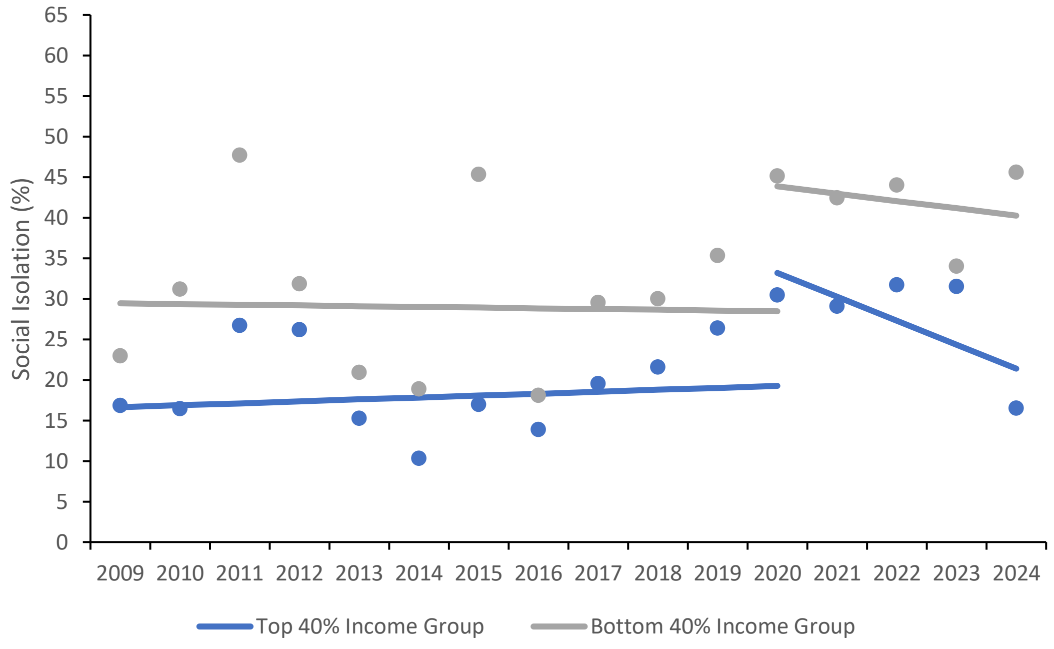

© 2025 Fuller-Rowell TE et al. *JAMA Network Open.*

eFigure 32. Trends in Social Isolation for Nigeria by Income Group. Fitted trajectories are derived from empirical Bayes estimates of the final best fitting model, with raw data overlaid.

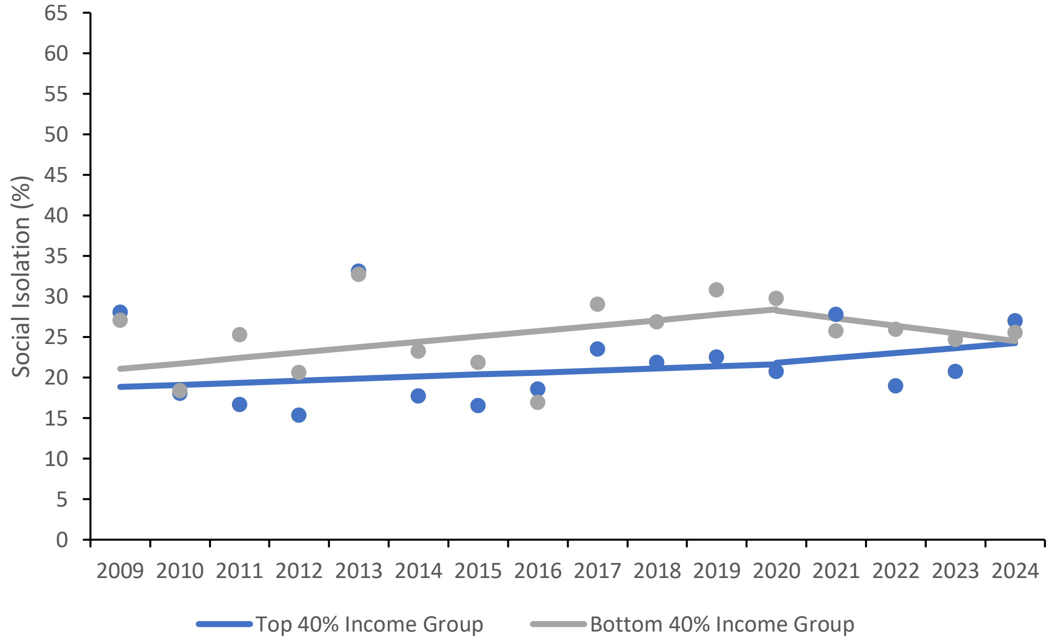

© 2025 Fuller-Rowell TE et al. *JAMA Network Open.*

eFigure 33. Trends in Social Isolation for Congo Brazzaville by Income Group. Fitted trajectories are derived from empirical Bayes estimates of the final best fitting model, with raw data overlaid.

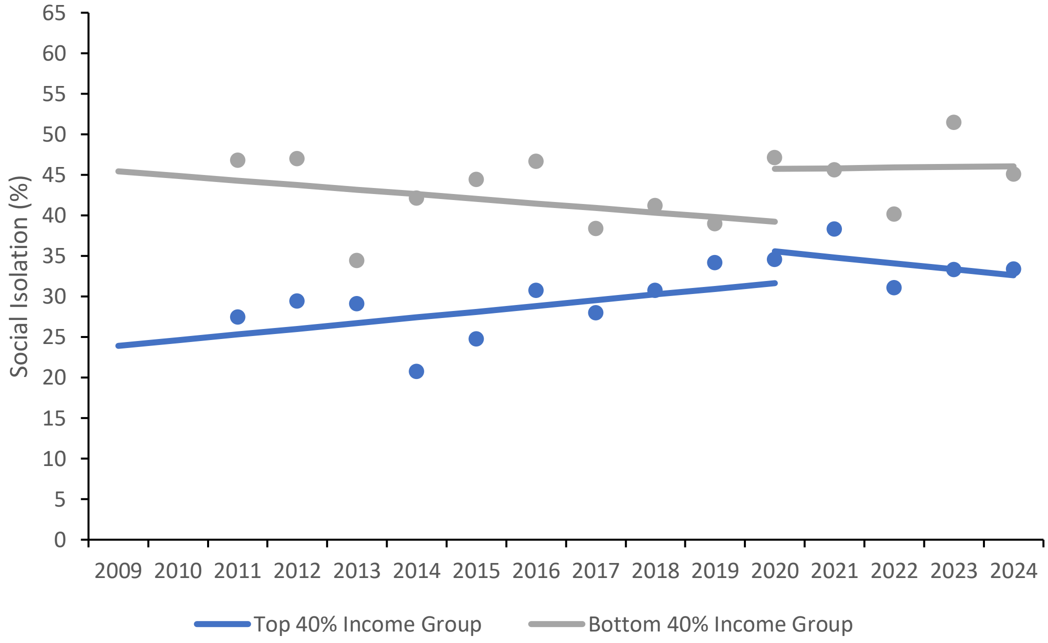

© 2025 Fuller-Rowell TE et al. *JAMA Network Open.*

eFigure 34. Trends in Social Isolation for Madagascar by Income Group. Fitted trajectories are derived from empirical Bayes estimates of the final best fitting model, with raw data overlaid.

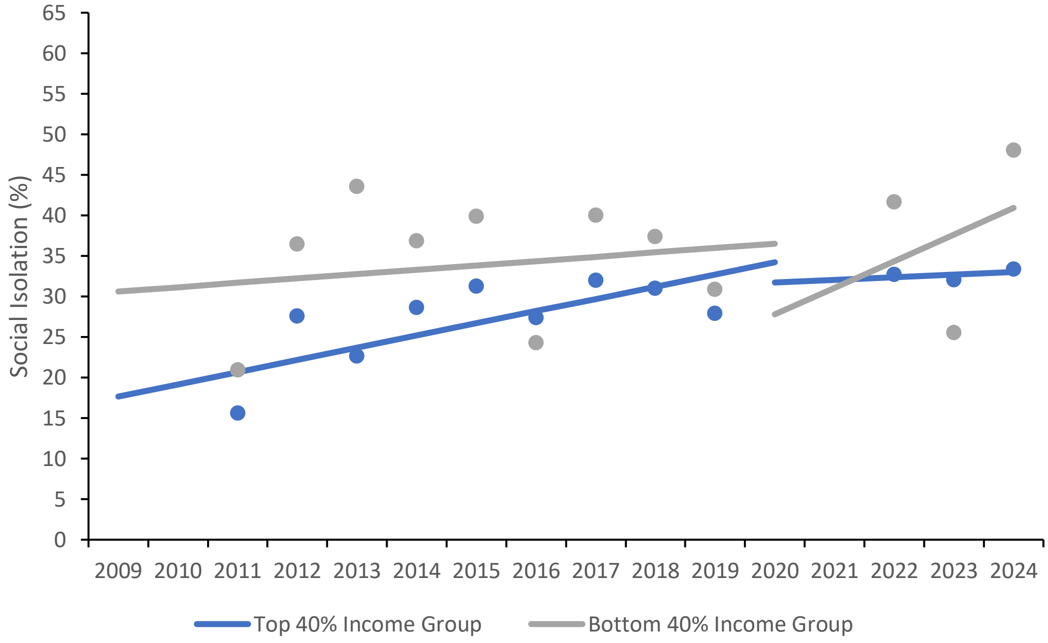

© 2025 Fuller-Rowell TE et al. *JAMA Network Open.*

eFigure 35. Trends in Social Isolation for South Africa by Income Group. Fitted trajectories are derived from empirical Bayes estimates of the final best fitting model, with raw data overlaid.

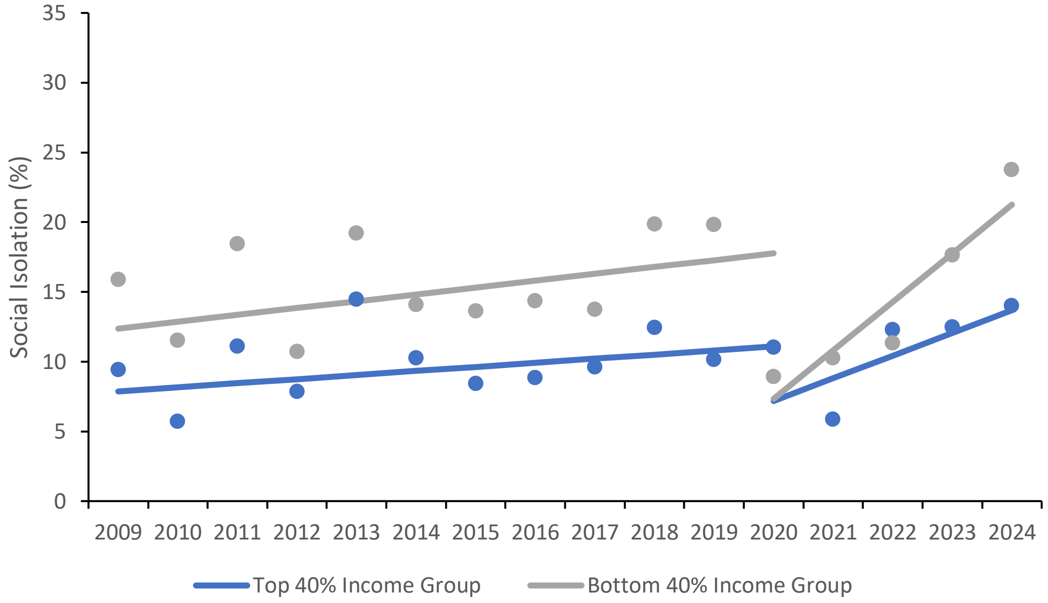

© 2025 Fuller-Rowell TE et al. *JAMA Network Open.*

eFigure 36. Trends in Social Isolation for Angola by Income Group. Fitted trajectories are derived from empirical Bayes estimates of the final best fitting model, with raw data overlaid.

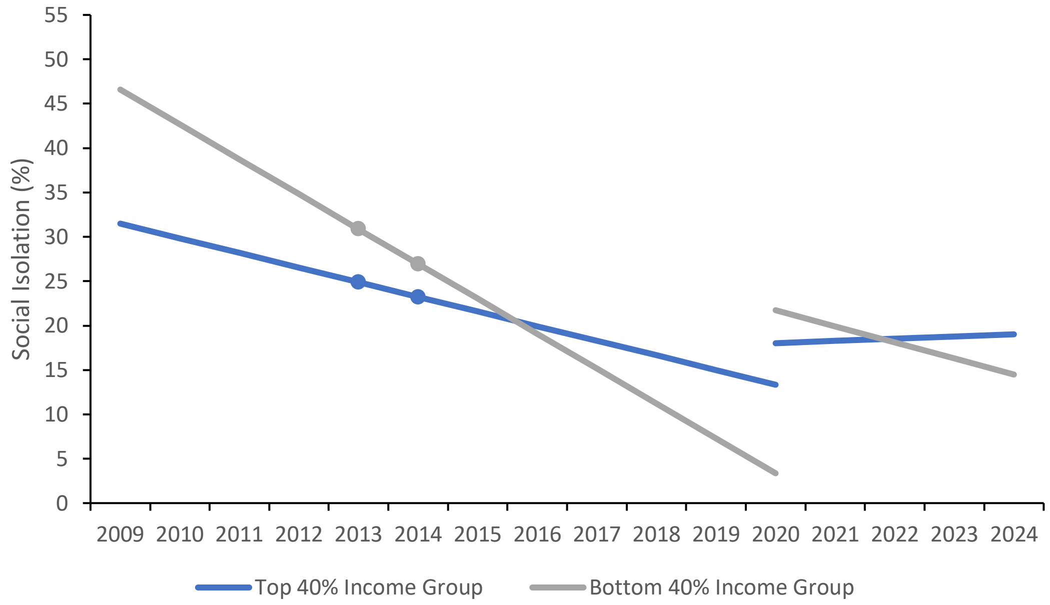

© 2025 Fuller-Rowell TE et al. *JAMA Network Open.*

eFigure 37. Trends in Social Isolation for Mozambique by Income Group. Fitted trajectories are derived from empirical Bayes estimates of the final best fitting model, with raw data overlaid.

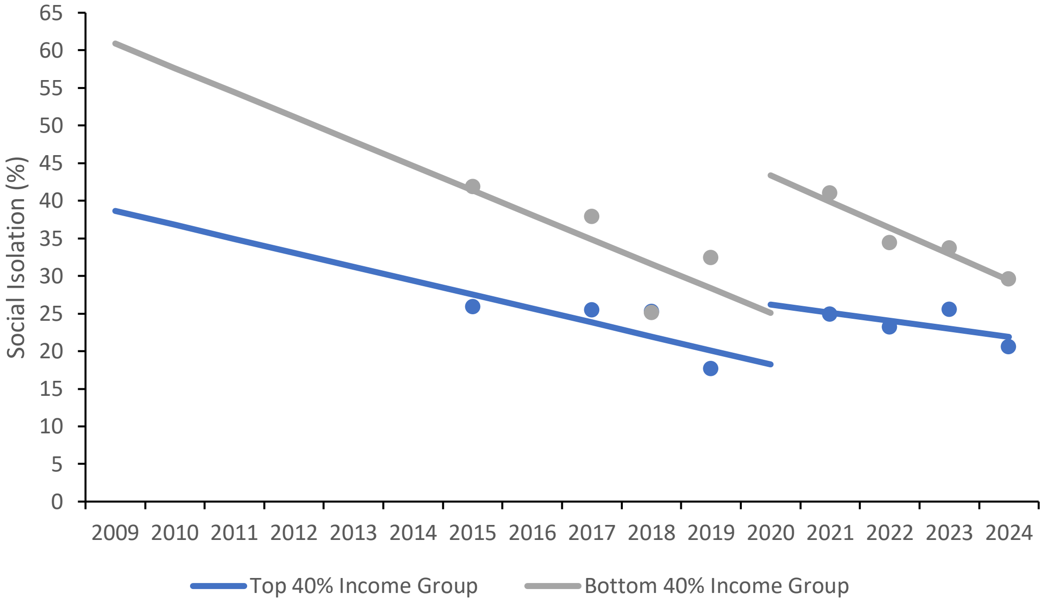

© 2025 Fuller-Rowell TE et al. *JAMA Network Open.*

eFigure 38. Trends in Social Isolation for Namibia by Income Group. Fitted trajectories are derived from empirical Bayes estimates of the final best fitting model, with raw data overlaid.

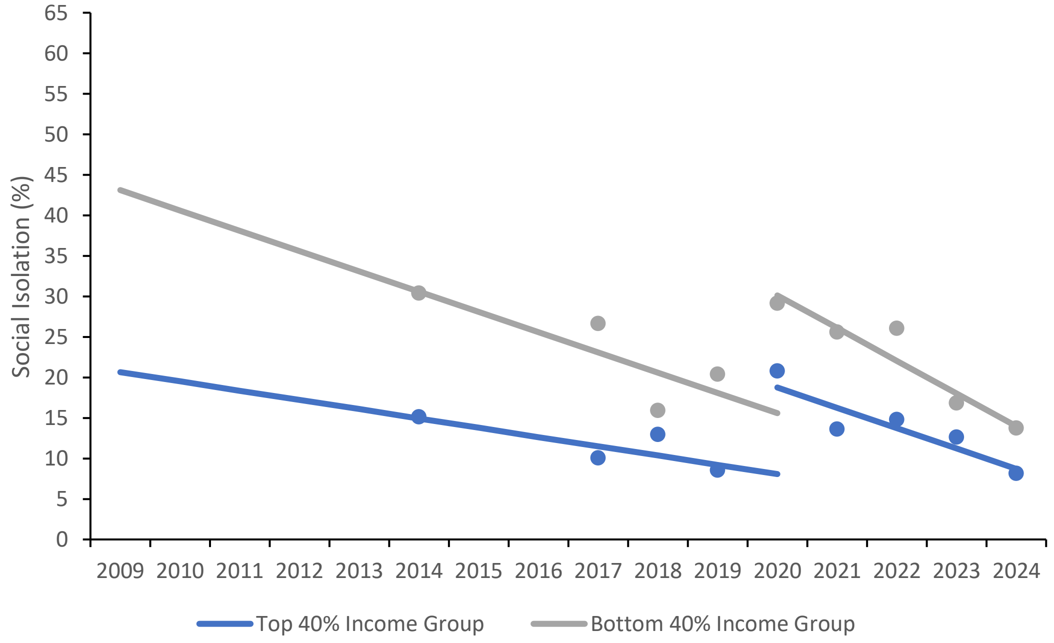

© 2025 Fuller-Rowell TE et al. *JAMA Network Open.*

eFigure 39. Trends in Social Isolation for Gabon by Income Group. Fitted trajectories are derived from empirical Bayes estimates of the final best fitting model, with raw data overlaid.

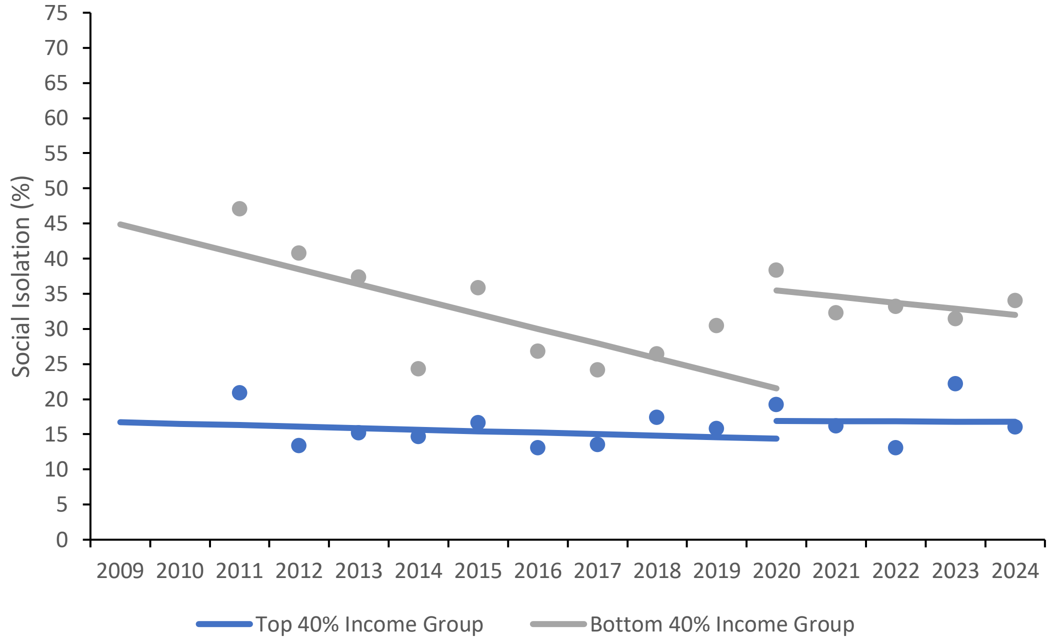

eFigure 40. Trends in Social Isolation for Mauritius by Income Group. Fitted trajectories are derived from empirical Bayes estimates of the final best fitting model, with raw data overlaid.

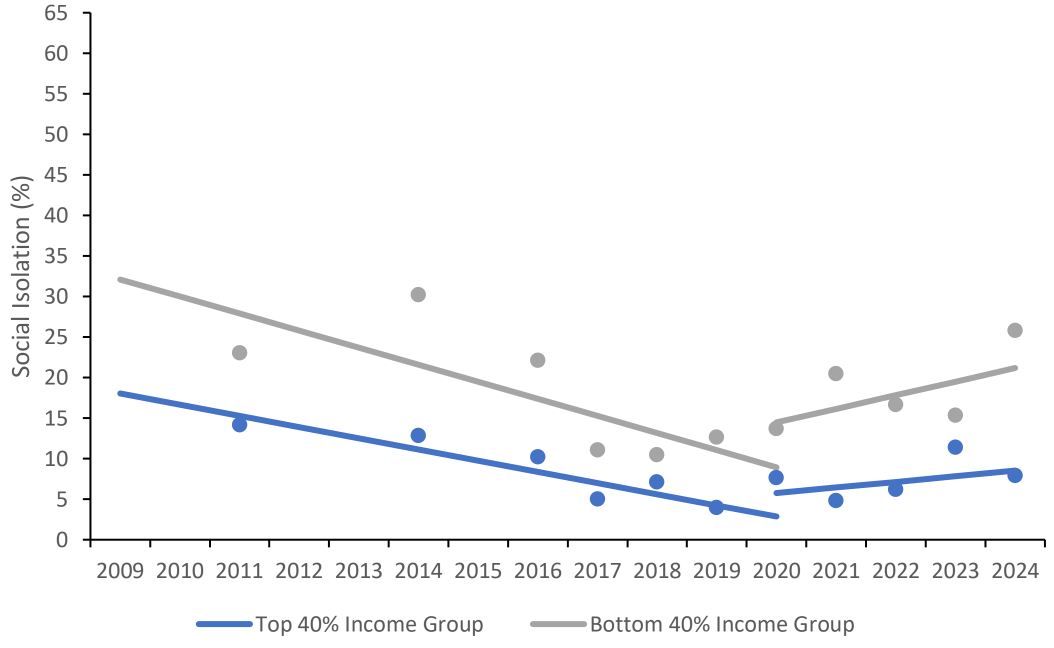

© 2025 Fuller-Rowell TE et al. *JAMA Network Open.*

eFigure 41. Trends in Social Isolation for Ethiopia by Income Group. Fitted trajectories are derived from empirical Bayes estimates of the final best fitting model, with raw data overlaid.

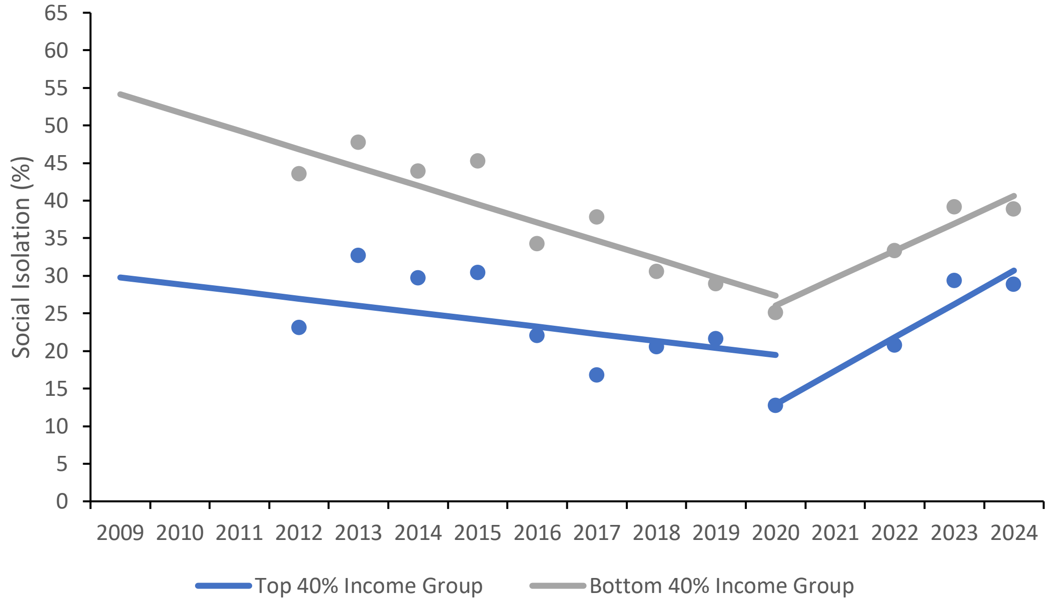

© 2025 Fuller-Rowell TE et al. *JAMA Network Open.*

eFigure 42. Trends in Social Isolation for Togo by Income Group. Fitted trajectories are derived from empirical Bayes estimates of the final best fitting model, with raw data overlaid.

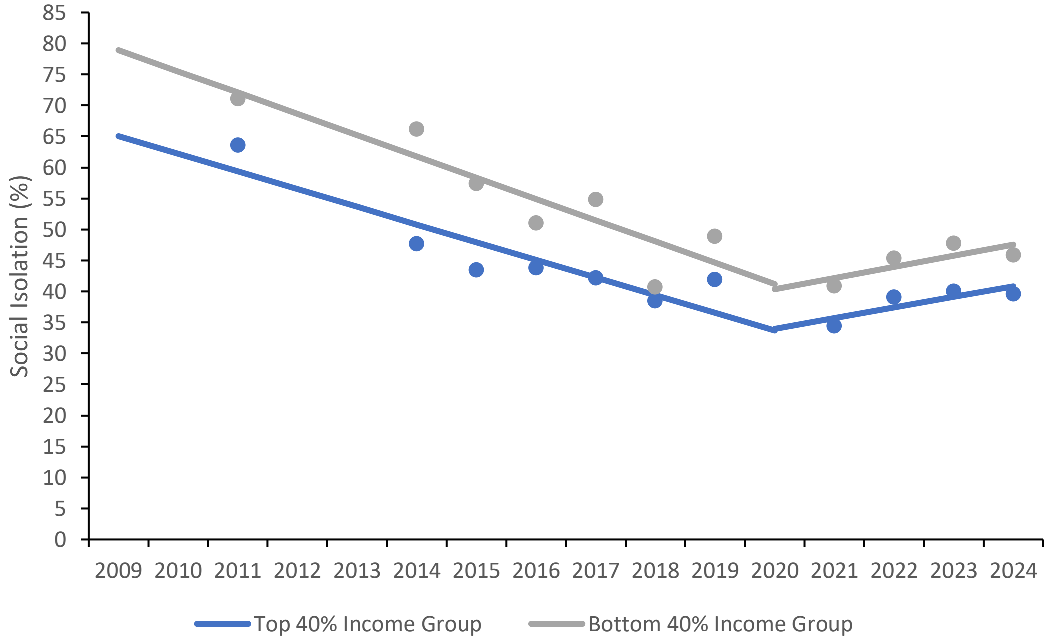

© 2025 Fuller-Rowell TE et al. *JAMA Network Open.*

eFigure 43. Trends in Social Isolation for Afghanistan by Income Group. Fitted trajectories are derived from empirical Bayes estimates of the final best fitting model, with raw data overlaid.

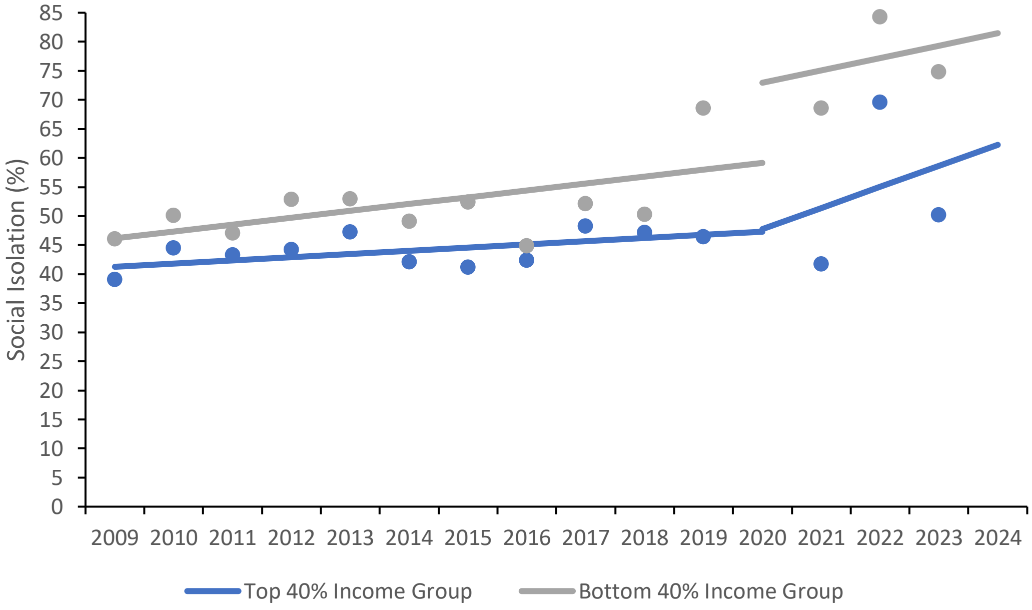

© 2025 Fuller-Rowell TE et al. *JAMA Network Open.*

eFigure 44. Trends in Social Isolation for Bangladesh by Income Group. Fitted trajectories are derived from empirical Bayes estimates of the final best fitting model, with raw data overlaid.

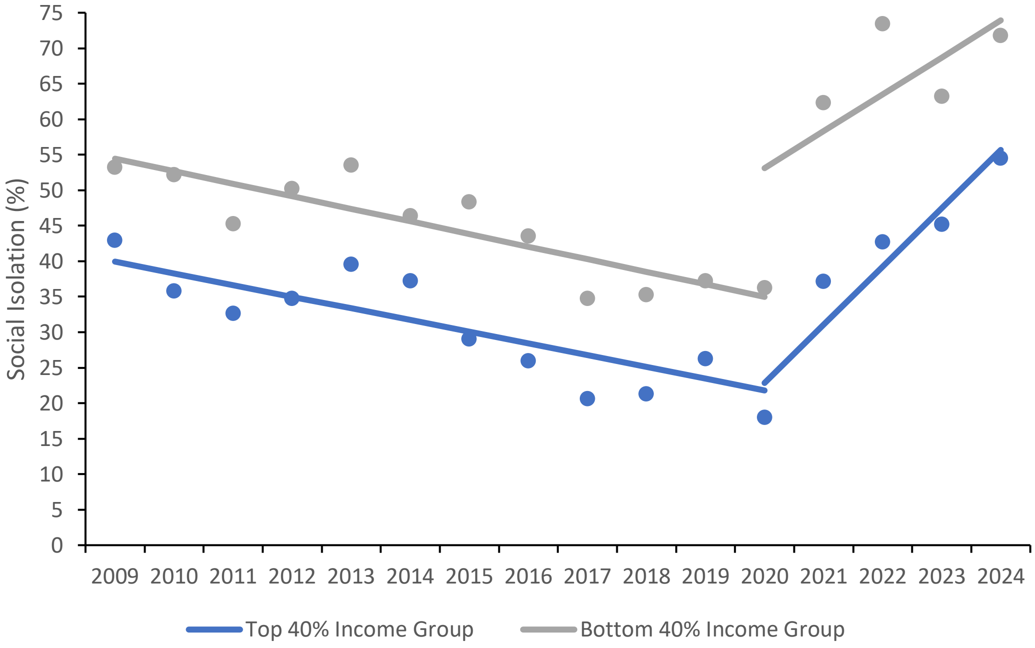

© 2025 Fuller-Rowell TE et al. *JAMA Network Open.*

eFigure 45. Trends in Social Isolation for India by Income Group. Fitted trajectories are derived from empirical Bayes estimates of the final best fitting model, with raw data overlaid.

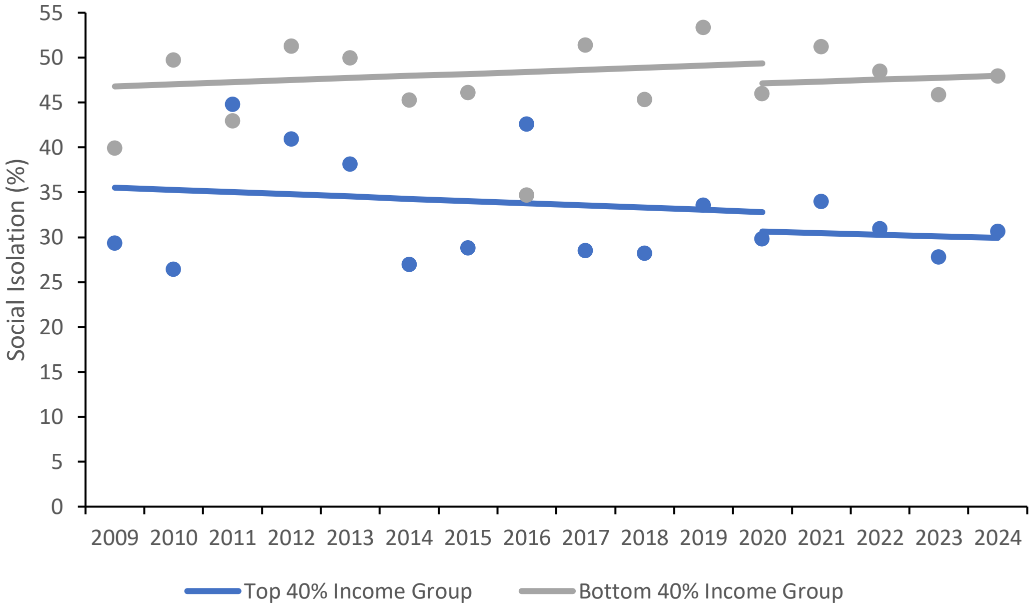

© 2025 Fuller-Rowell TE et al. *JAMA Network Open.*

eFigure 46. Trends in Social Isolation for Bhutan by Income Group. Fitted trajectories are derived from empirical Bayes estimates of the final best fitting model, with raw data overlaid.

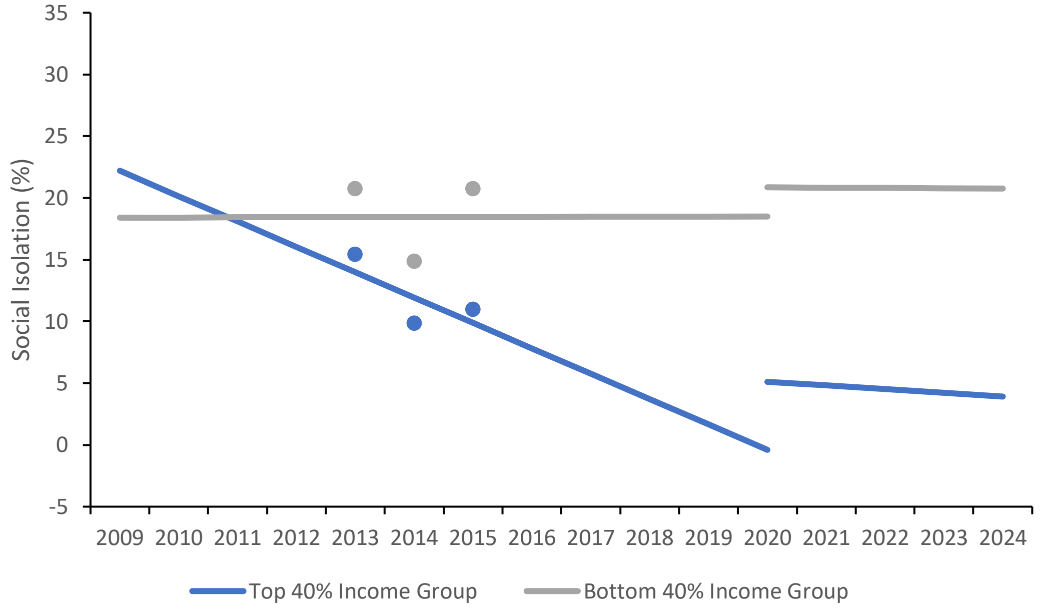

© 2025 Fuller-Rowell TE et al. *JAMA Network Open.*

eFigure 47. Trends in Social Isolation for Sri Lanka by Income Group. Fitted trajectories are derived from empirical Bayes estimates of the final best fitting model, with raw data overlaid.

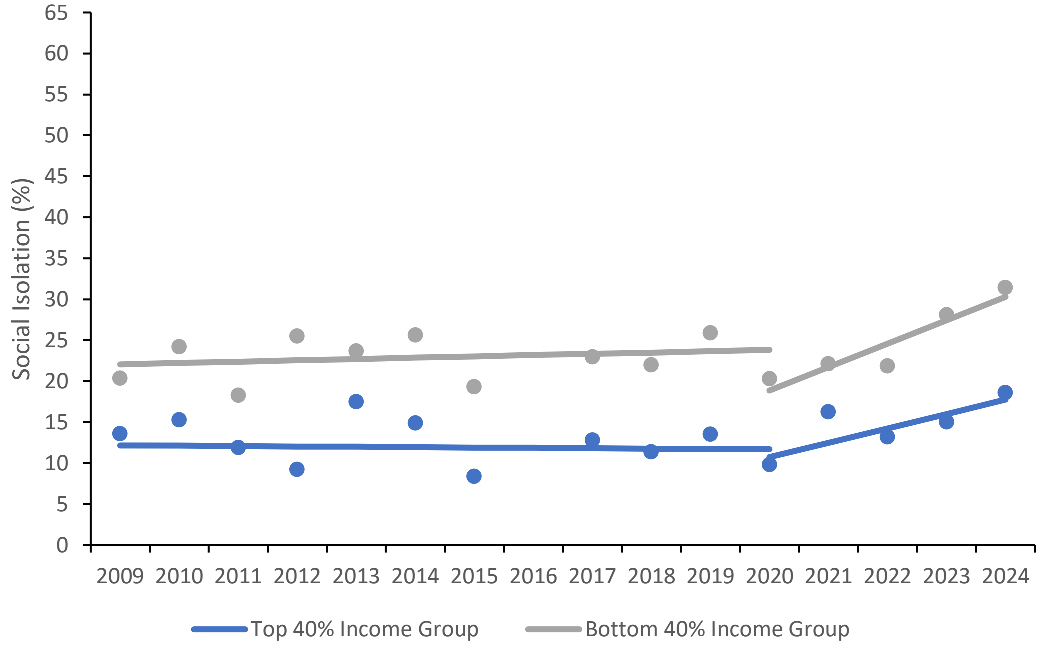

© 2025 Fuller-Rowell TE et al. *JAMA Network Open.*

eFigure 48. Trends in Social Isolation for Pakistan by Income Group. Fitted trajectories are derived from empirical Bayes estimates of the final best fitting model, with raw data overlaid.

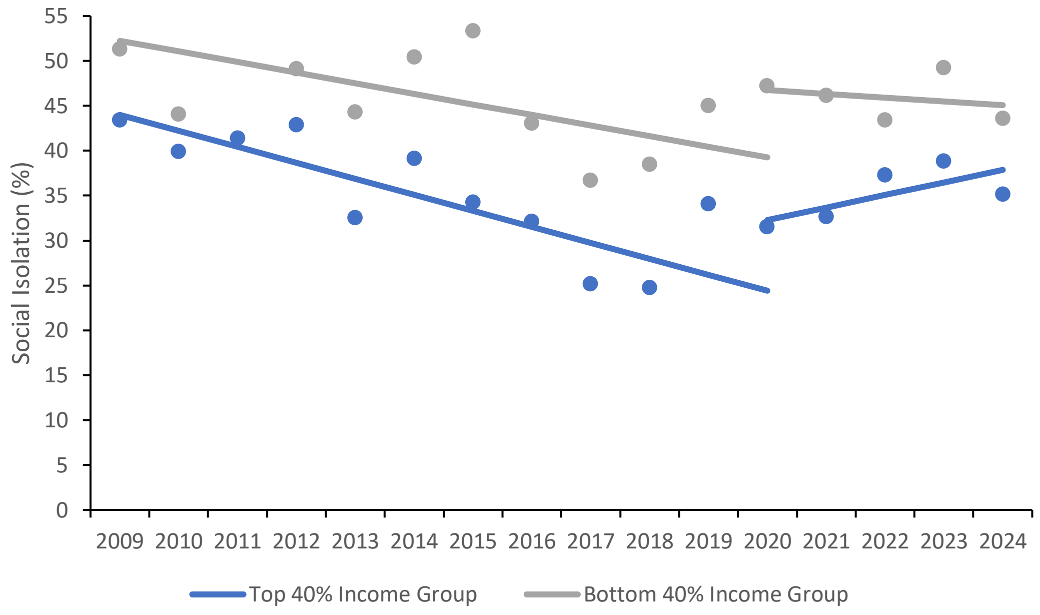

© 2025 Fuller-Rowell TE et al. *JAMA Network Open.*

eFigure 49. Trends in Social Isolation for Nepal by Income Group. Fitted trajectories are derived from empirical Bayes estimates of the final best fitting model, with raw data overlaid.

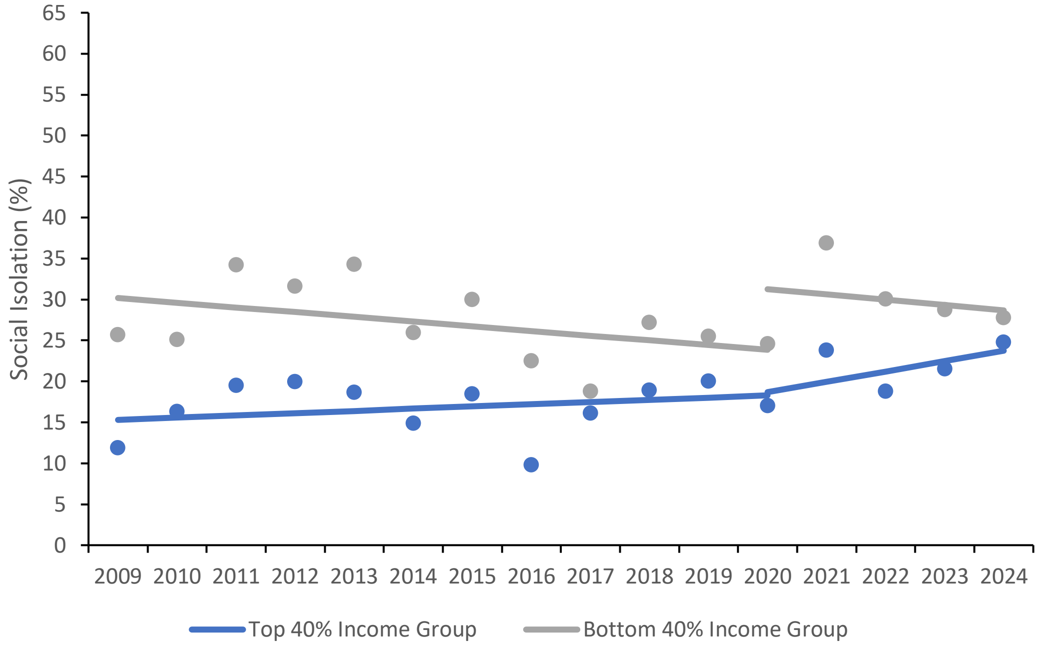

© 2025 Fuller-Rowell TE et al. *JAMA Network Open.*

eFigure 50. Trends in Social Isolation for Haiti by Income Group. Fitted trajectories are derived from empirical Bayes estimates of the final best fitting model, with raw data overlaid.

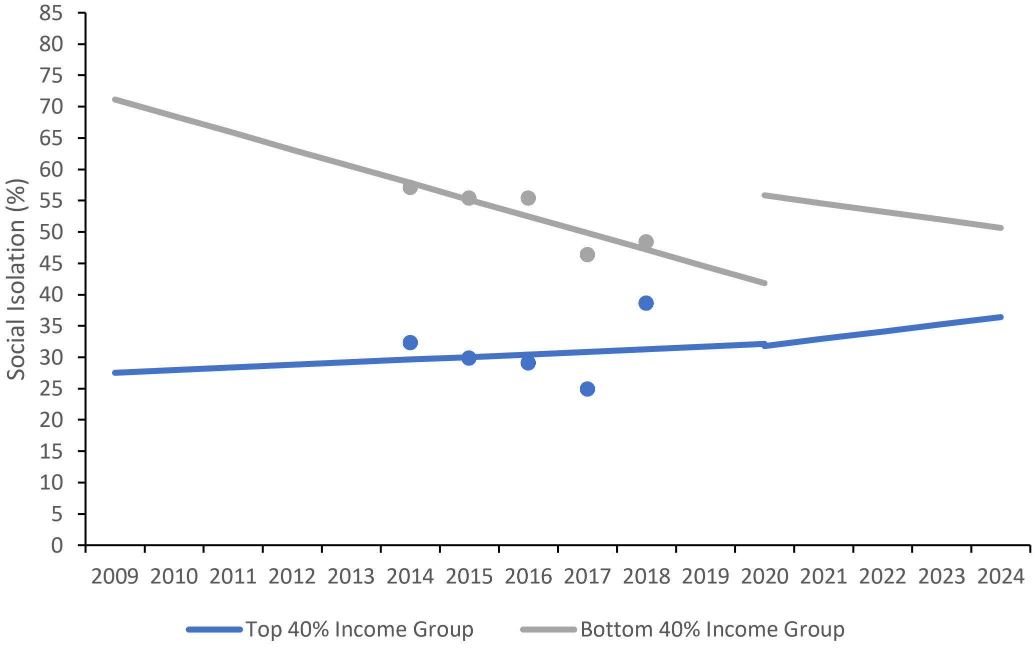

© 2025 Fuller-Rowell TE et al. *JAMA Network Open.*

eFigure 51. Trends in Social Isolation for Brazil by Income Group. Fitted trajectories are derived from empirical Bayes estimates of the final best fitting model, with raw data overlaid.

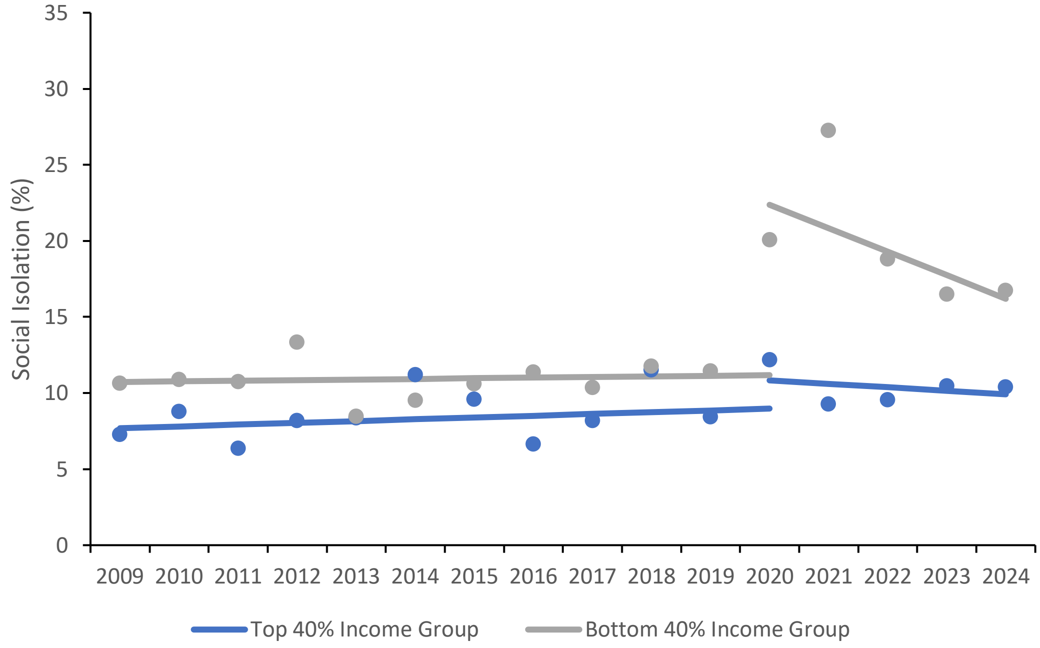

© 2025 Fuller-Rowell TE et al. *JAMA Network Open.*

eFigure 52. Trends in Social Isolation for Costa Rica by Income Group. Fitted trajectories are derived from empirical Bayes estimates of the final best fitting model, with raw data overlaid.

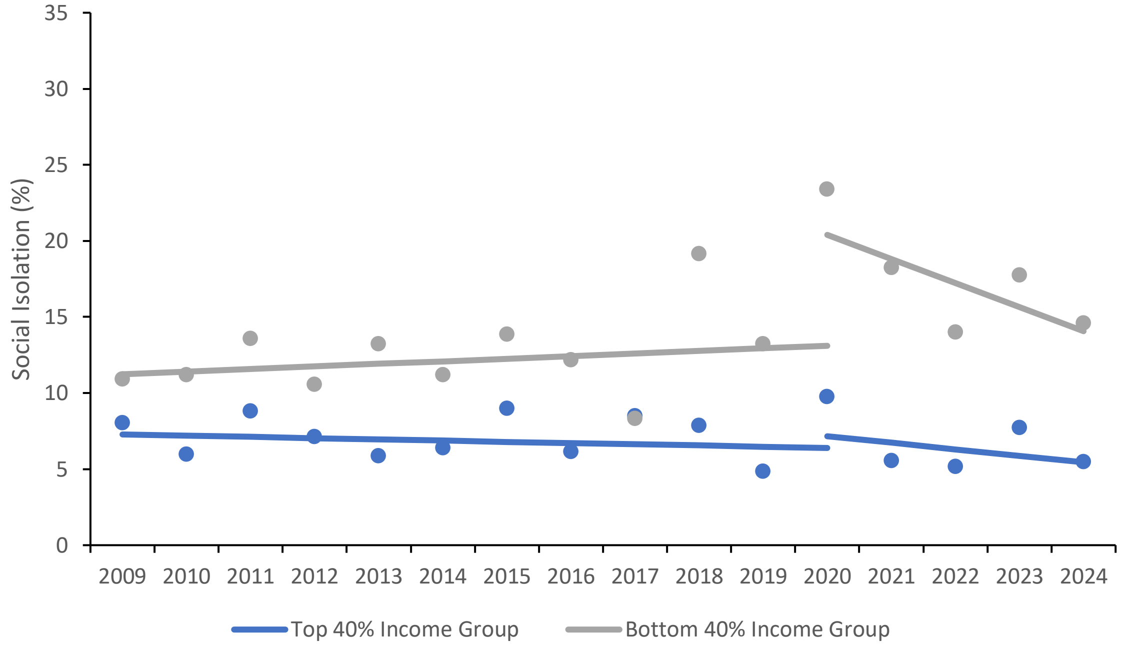

© 2025 Fuller-Rowell TE et al. *JAMA Network Open.*

eFigure 53. Trends in Social Isolation for Colombia by Income Group. Fitted trajectories are derived from empirical Bayes estimates of the final best fitting model, with raw data overlaid.

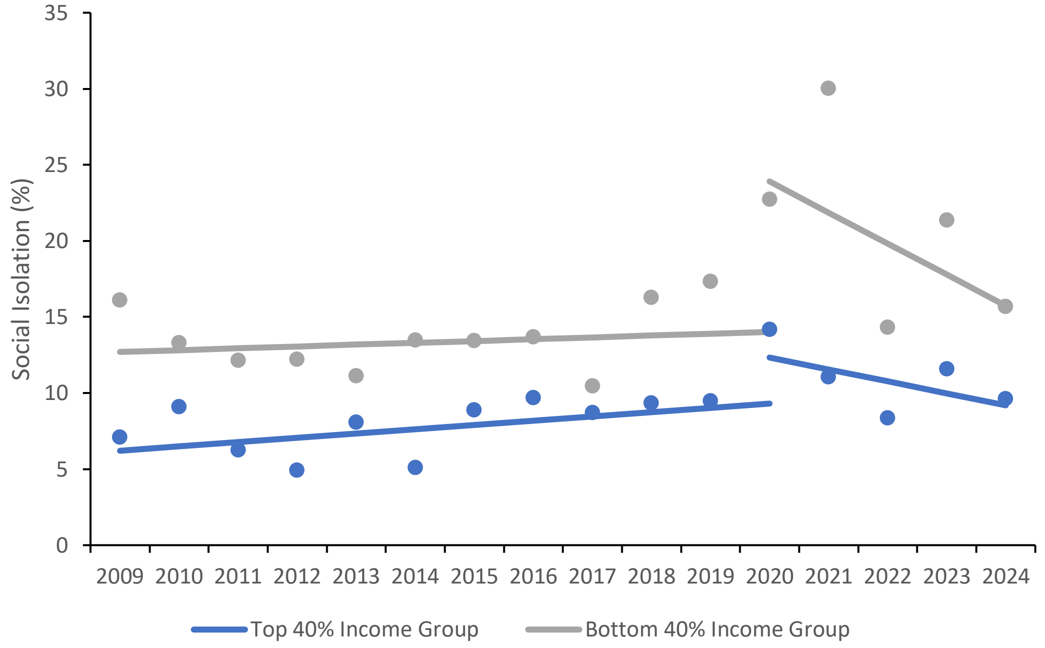

© 2025 Fuller-Rowell TE et al. *JAMA Network Open.*

eFigure 54. Trends in Social Isolation for Jamaica by Income Group. Fitted trajectories are derived from empirical Bayes estimates of the final best fitting model, with raw data overlaid.

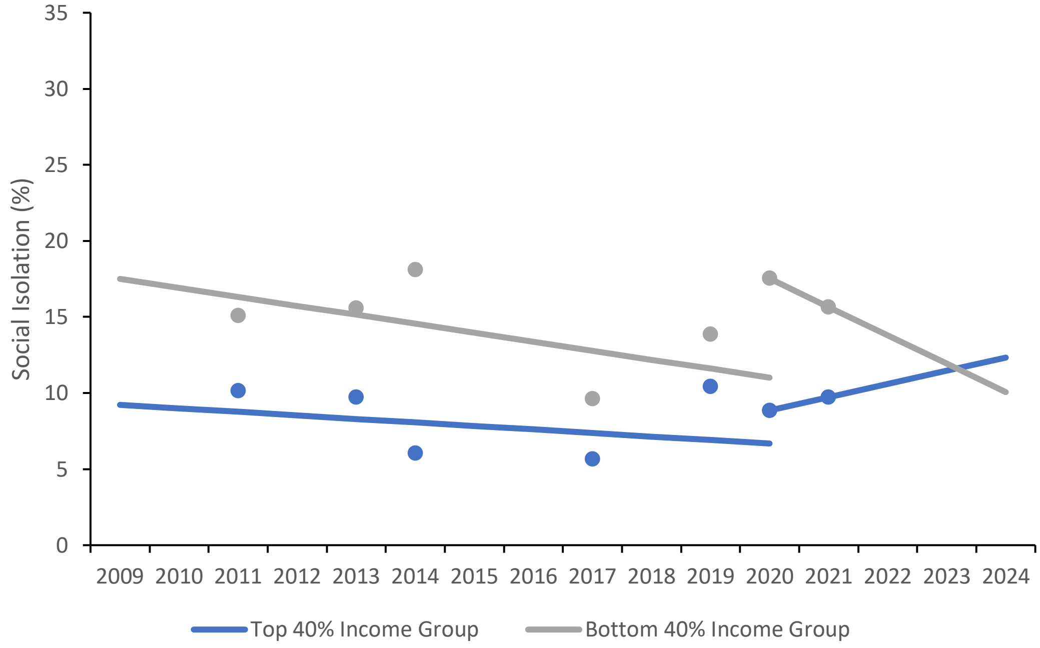

© 2025 Fuller-Rowell TE et al. *JAMA Network Open.*

eFigure 55. Trends in Social Isolation for Venezuela by Income Group. Fitted trajectories are derived from empirical Bayes estimates of the final best fitting model, with raw data overlaid.

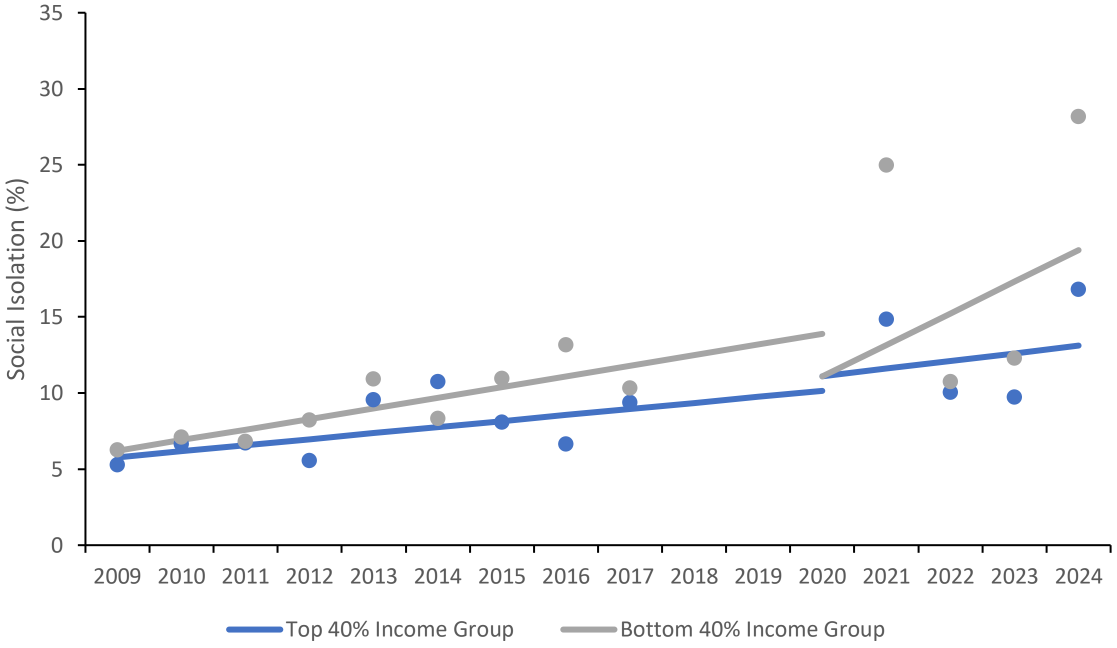

eFigure 56. Trends in Social Isolation for Mexico by Income Group. Fitted trajectories are derived from empirical Bayes estimates of the final best fitting model, with raw data overlaid.

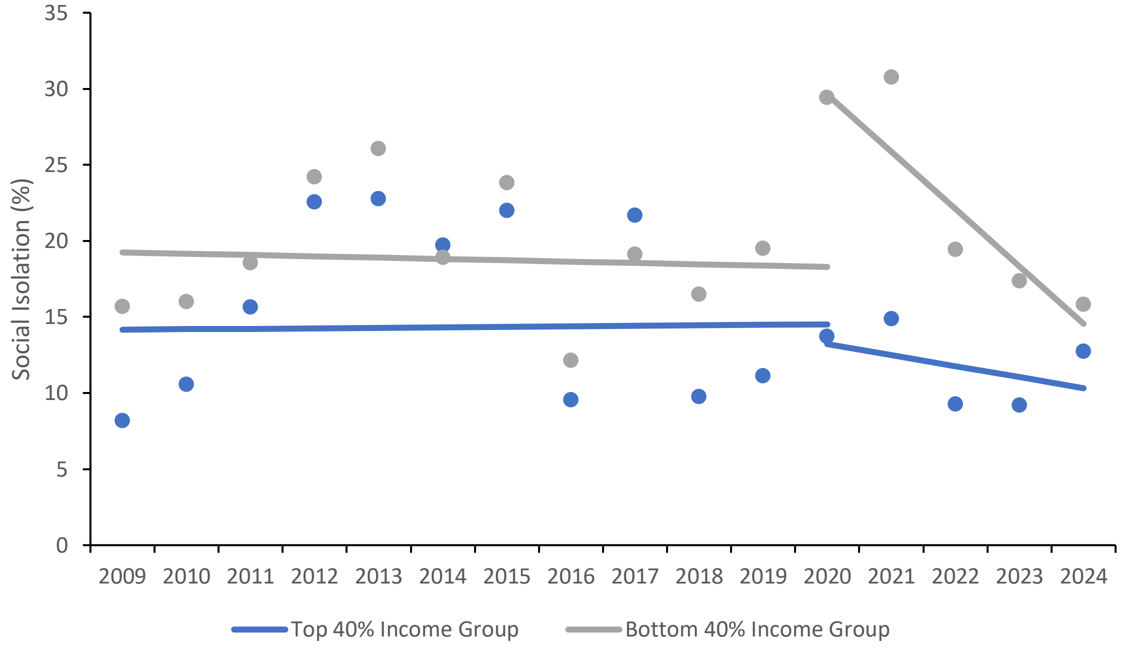

© 2025 Fuller-Rowell TE et al. *JAMA Network Open.*

eFigure 57. Trends in Social Isolation for Argentina by Income Group. Fitted trajectories are derived from empirical Bayes estimates of the final best fitting model, with raw data overlaid.

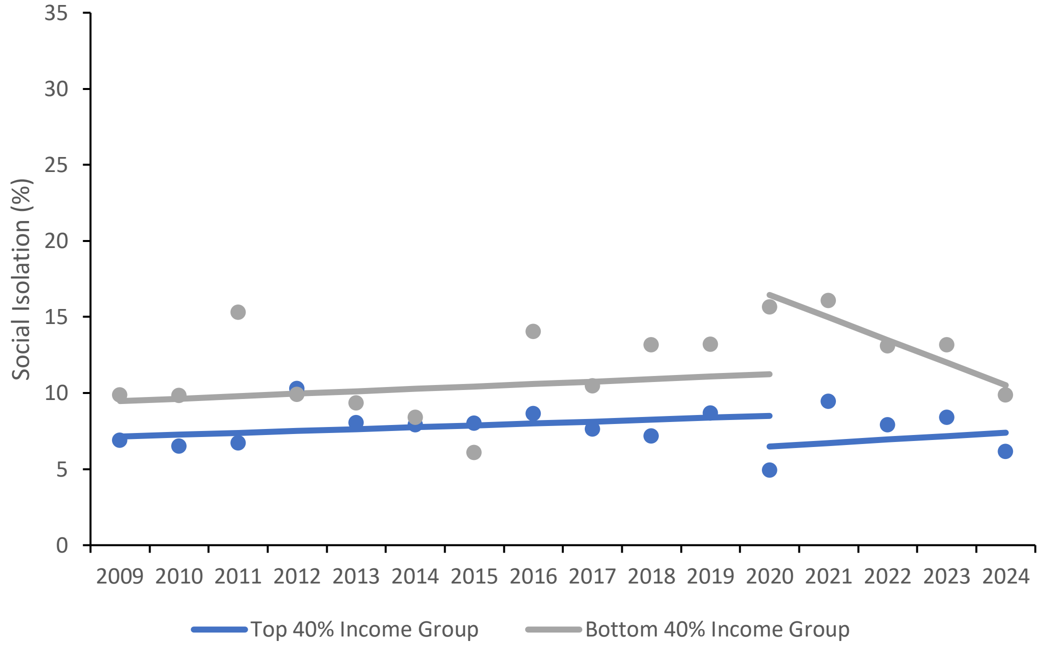

© 2025 Fuller-Rowell TE et al. *JAMA Network Open.*

eFigure 58. Trends in Social Isolation for Honduras by Income Group. Fitted trajectories are derived from empirical Bayes estimates of the final best fitting model, with raw data overlaid.

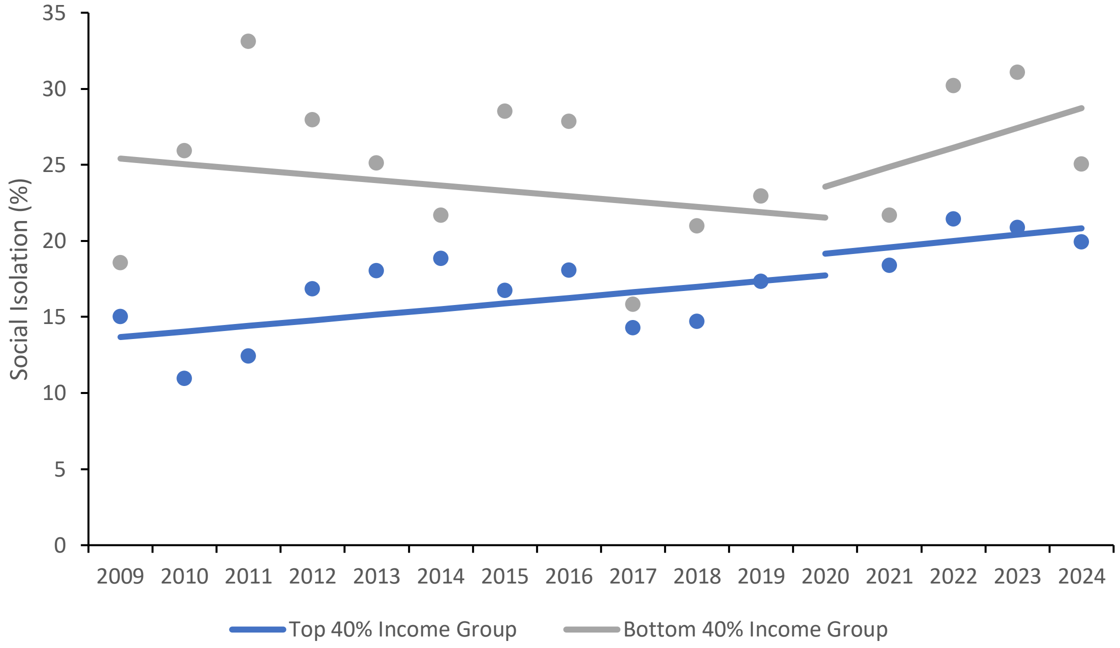

© 2025 Fuller-Rowell TE et al. *JAMA Network Open.*

eFigure 59. Trends in Social Isolation for Ecuador by Income Group. Fitted trajectories are derived from empirical Bayes estimates of the final best fitting model, with raw data overlaid.

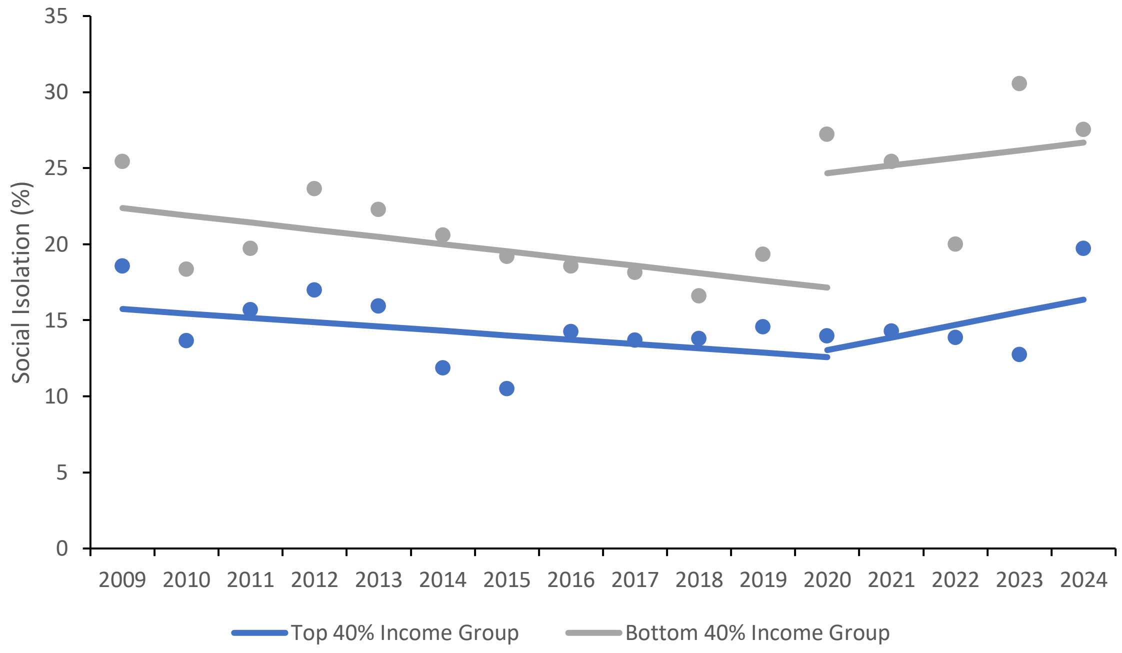

© 2025 Fuller-Rowell TE et al. *JAMA Network Open.*

eFigure 60. Trends in Social Isolation for Panama by Income Group. Fitted trajectories are derived from empirical Bayes estimates of the final best fitting model, with raw data overlaid.

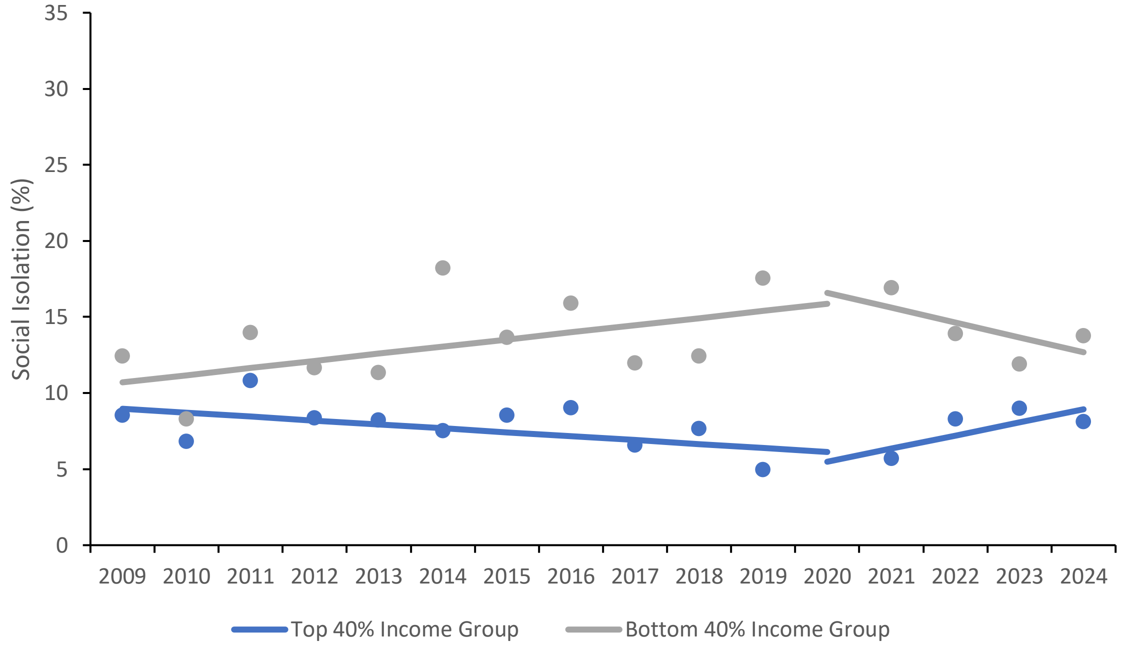

© 2025 Fuller-Rowell TE et al. *JAMA Network Open.*

eFigure 61. Trends in Social Isolation for Bolivia by Income Group. Fitted trajectories are derived from empirical Bayes estimates of the final best fitting model, with raw data overlaid.

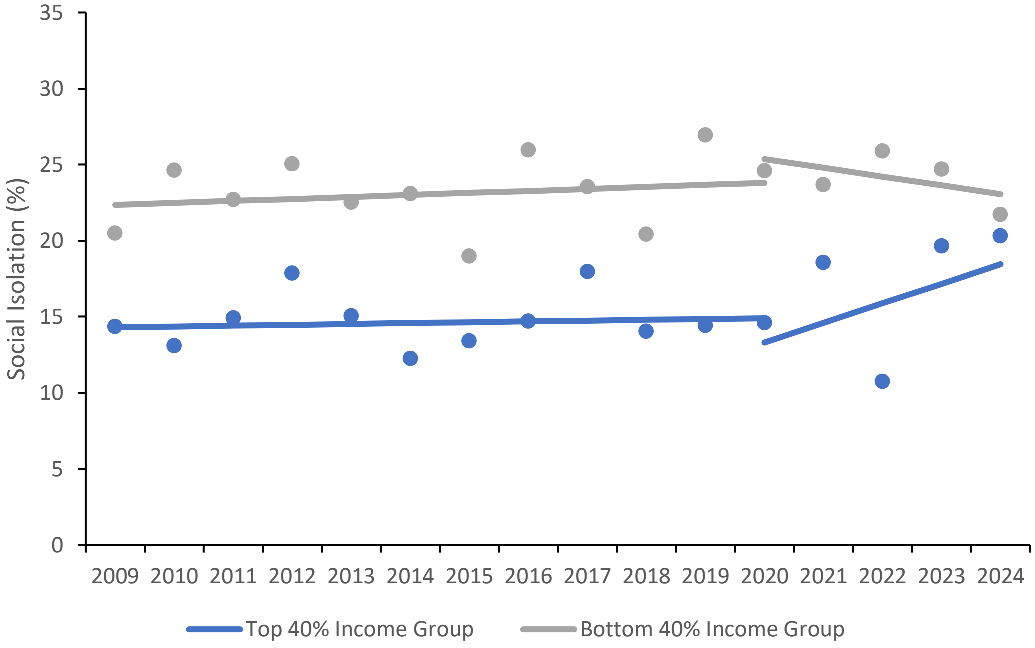

© 2025 Fuller-Rowell TE et al. *JAMA Network Open.*

eFigure 62. Trends in Social Isolation for Dominican Republic by Income Group. Fitted trajectories are derived from empirical Bayes estimates of the final best fitting model, with raw data overlaid.

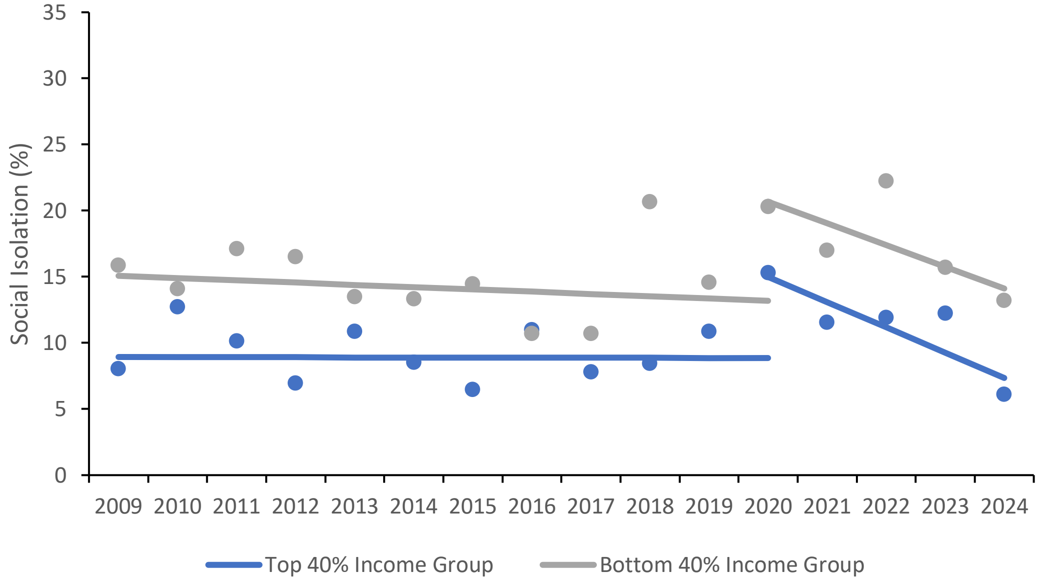

© 2025 Fuller-Rowell TE et al. *JAMA Network Open.*

eFigure 63. Trends in Social Isolation for Guatamala by Income Group. Fitted trajectories are derived from empirical Bayes estimates of the final best fitting model, with raw data overlaid.

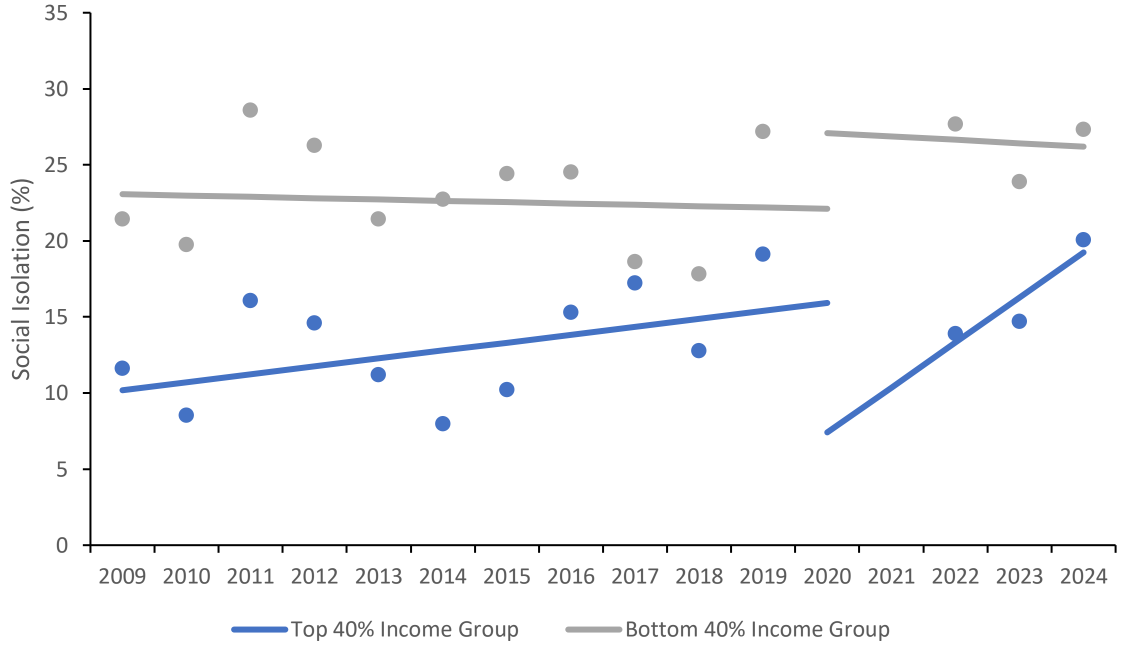

© 2025 Fuller-Rowell TE et al. *JAMA Network Open.*

eFigure 64. Trends in Social Isolation for Nicaragua by Income Group. Fitted trajectories are derived from empirical Bayes estimates of the final best fitting model, with raw data overlaid.

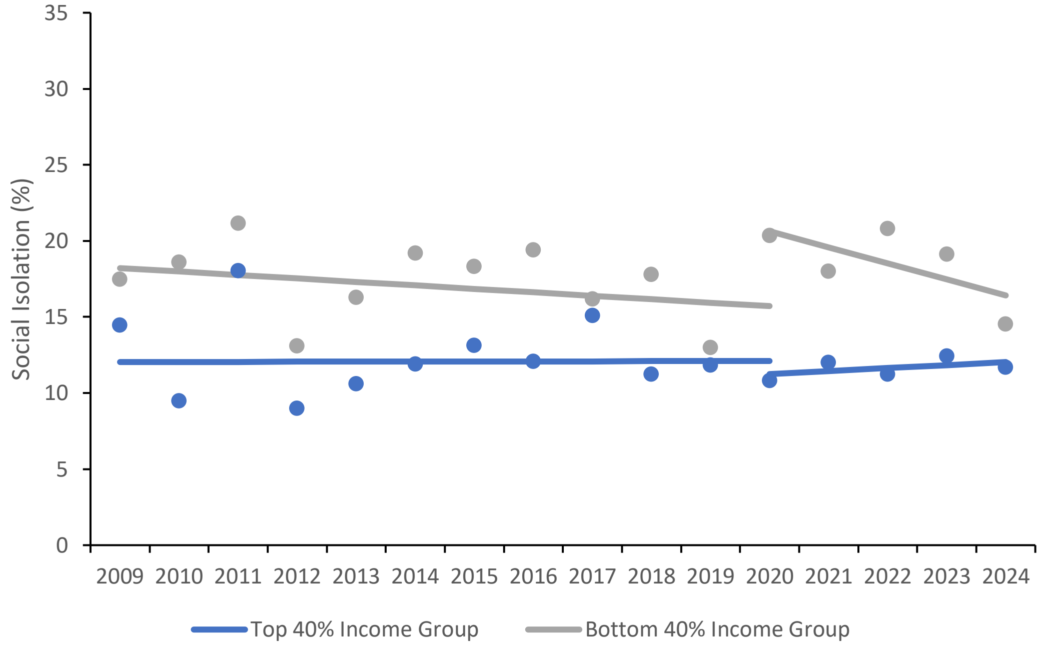

© 2025 Fuller-Rowell TE et al. *JAMA Network Open.*

eFigure 65. Trends in Social Isolation for Paraguay by Income Group. Fitted trajectories are derived from empirical Bayes estimates of the final best fitting model, with raw data overlaid.

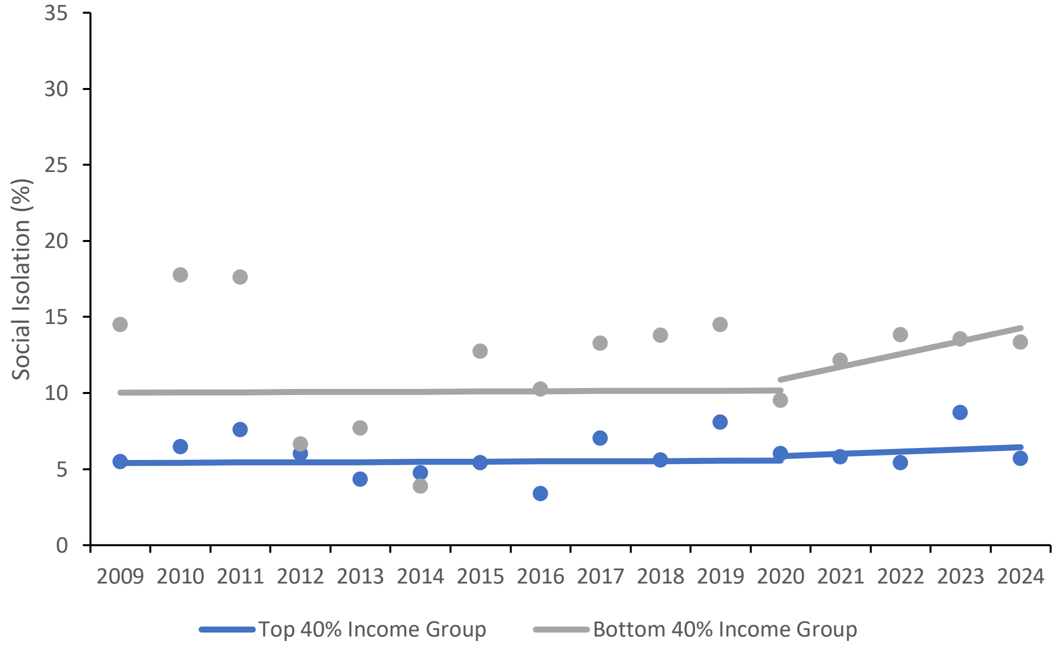

© 2025 Fuller-Rowell TE et al. *JAMA Network Open.*

eFigure 66. Trends in Social Isolation for Uruguay by Income Group**.** Fitted trajectories are derived from empirical Bayes estimates of the final best fitting model, with raw data overlaid.

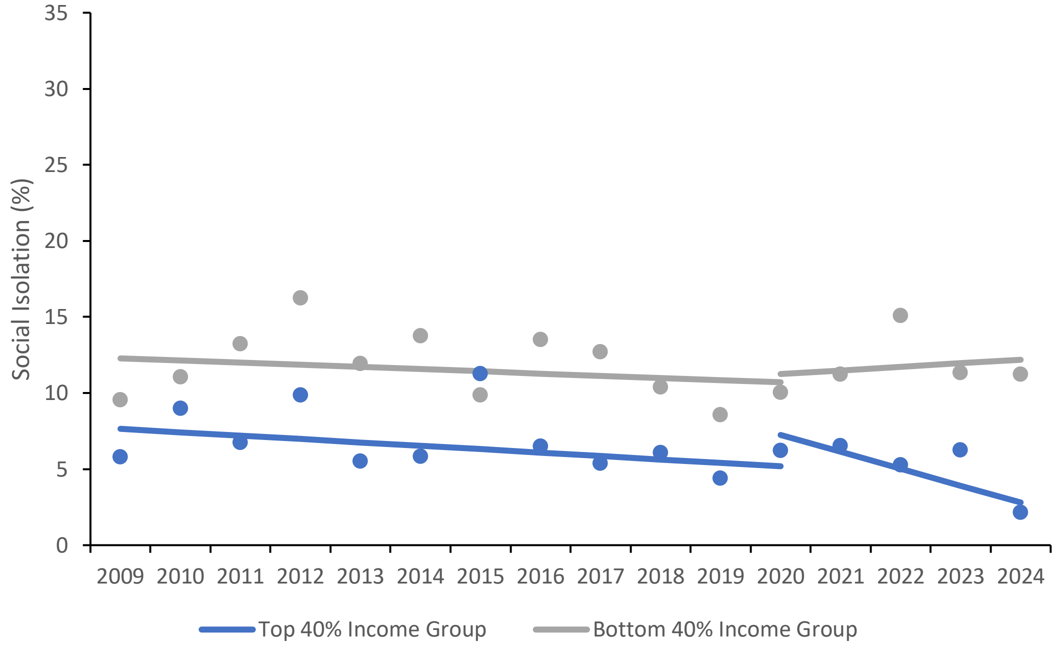

© 2025 Fuller-Rowell TE et al. *JAMA Network Open.*

eFigure 67. Trends in Social Isolation for Peru by Income Group. Fitted trajectories are derived from empirical Bayes estimates of the final best fitting model, with raw data overlaid.

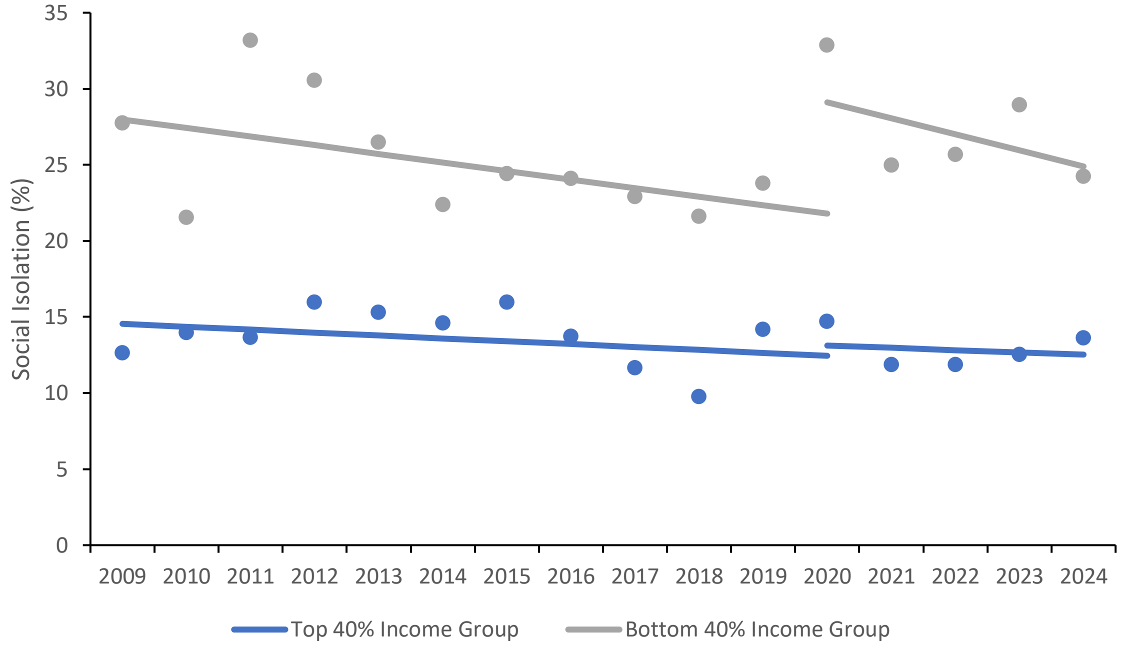

© 2025 Fuller-Rowell TE et al. *JAMA Network Open.*

eFigure 68. Trends in Social Isolation for El Salvador by Income Group. Fitted trajectories are derived from empirical Bayes estimates of the final best fitting model, with raw data overlaid.

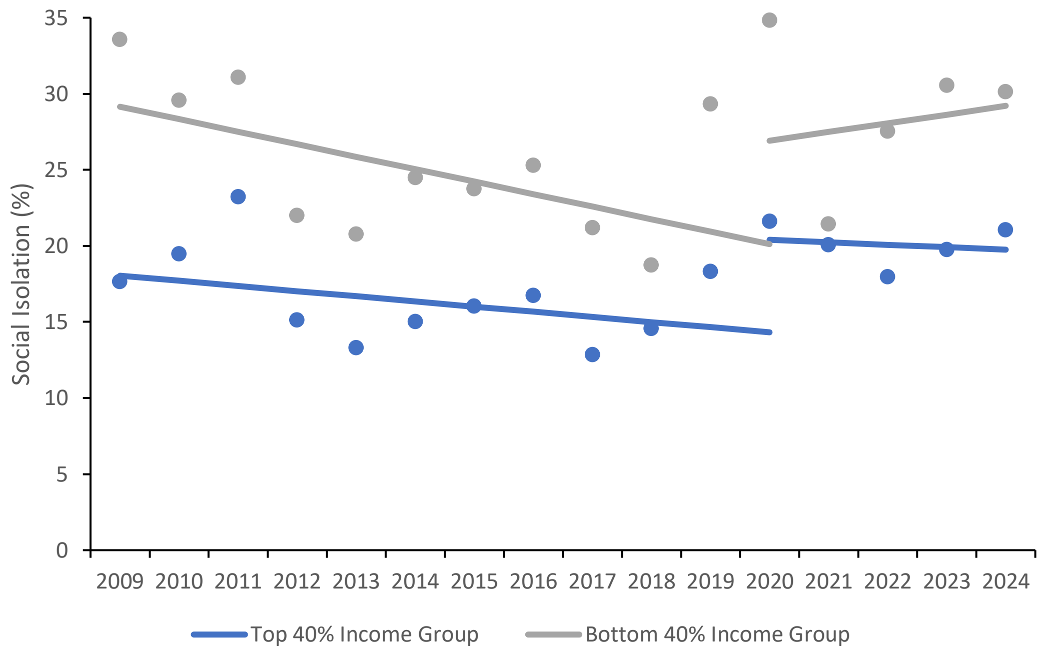

© 2025 Fuller-Rowell TE et al. *JAMA Network Open.*

eFigure 69. Trends in Social Isolation for Chile by Income Group. Fitted trajectories are derived from empirical Bayes estimates of the final best fitting model, with raw data overlaid.

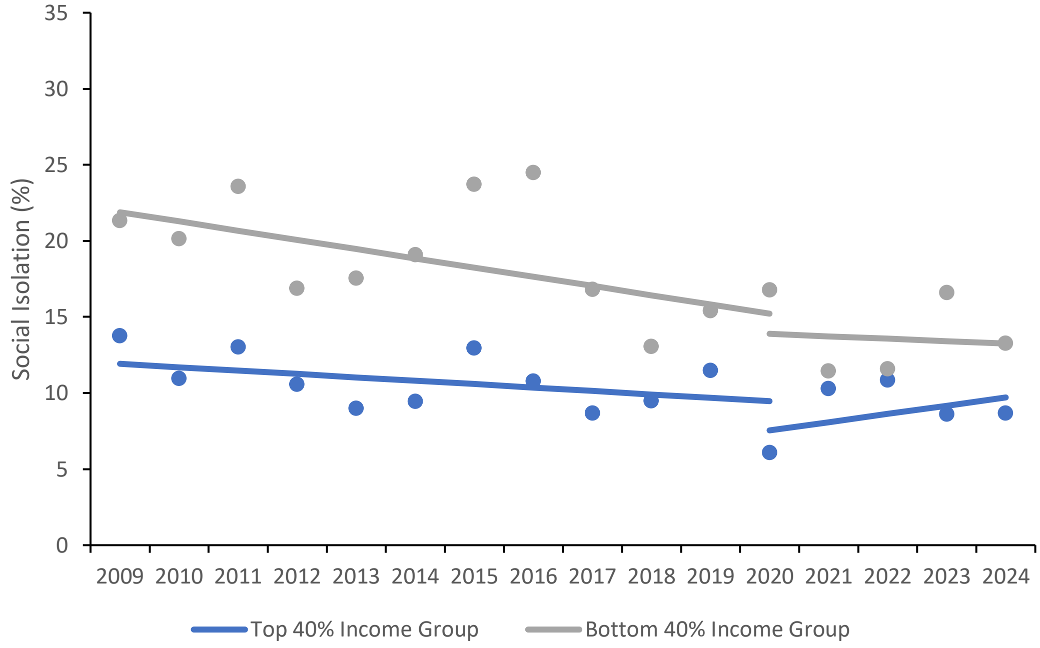

© 2025 Fuller-Rowell TE et al. *JAMA Network Open.*

eFigure 70. Trends in Social Isolation for Belize by Income Group. Fitted trajectories are derived from empirical Bayes estimates of the final best fitting model, with raw data overlaid.

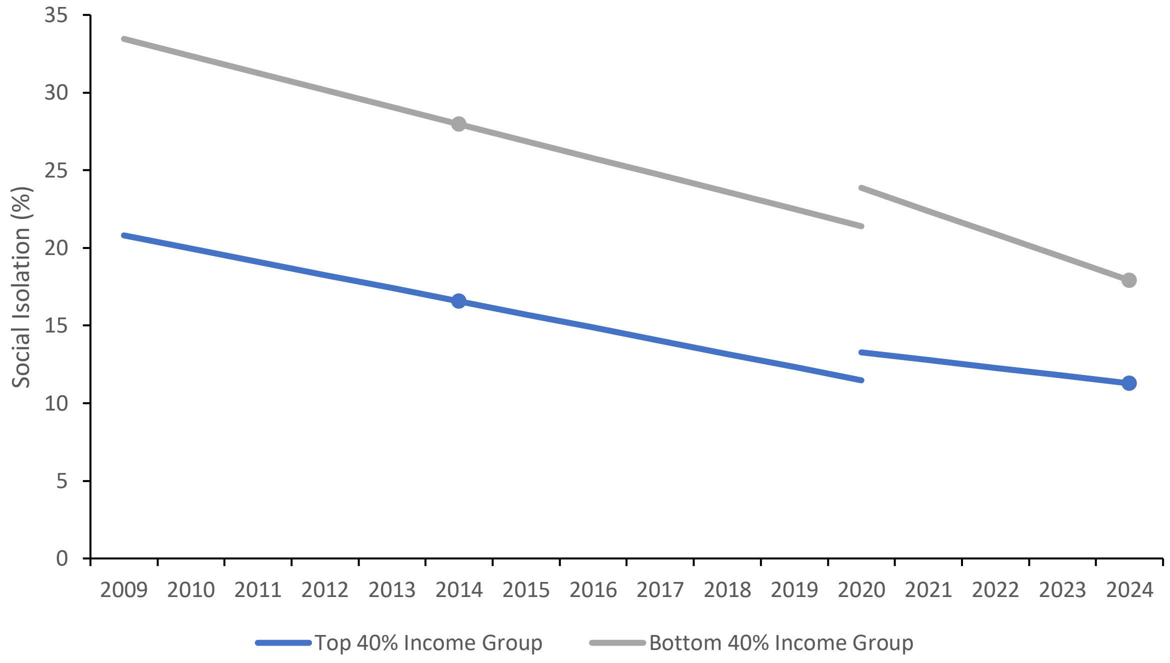

© 2025 Fuller-Rowell TE et al. *JAMA Network Open.*

eFigure 71. Trends in Social Isolation for Trinidad and Tobago by Income Group. Fitted trajectories are derived from empirical Bayes estimates of the final best fitting model, with raw data overlaid.

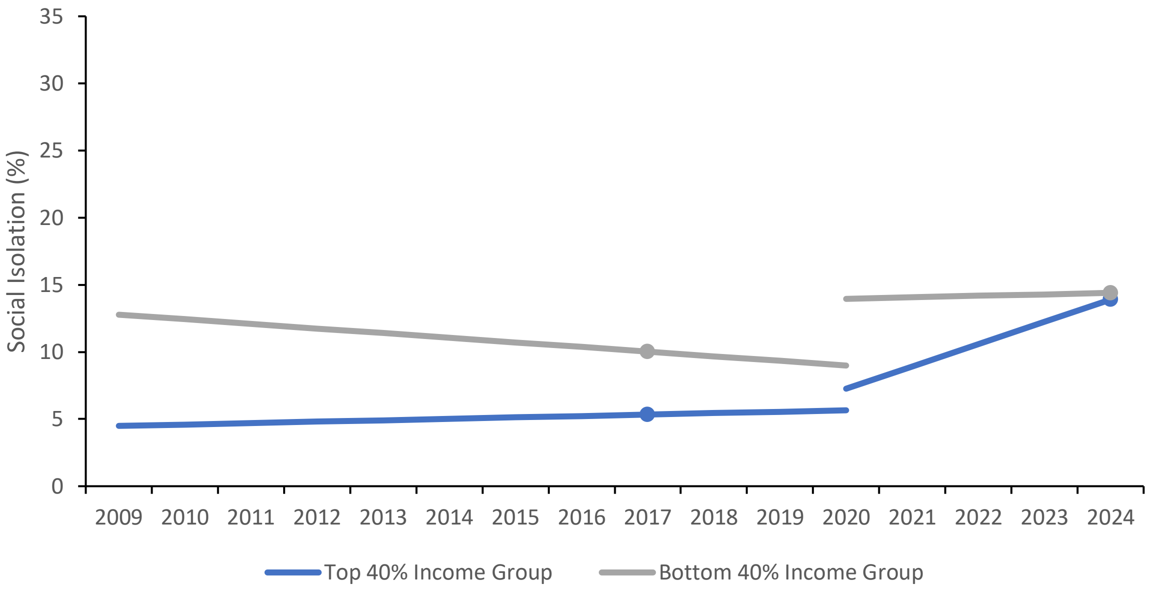

© 2025 Fuller-Rowell TE et al. *JAMA Network Open.*

eFigure 72. Trends in Social Isolation for Puerto Rico by Income Group. Fitted trajectories are derived from empirical Bayes estimates of the final best fitting model, with raw data overlaid.

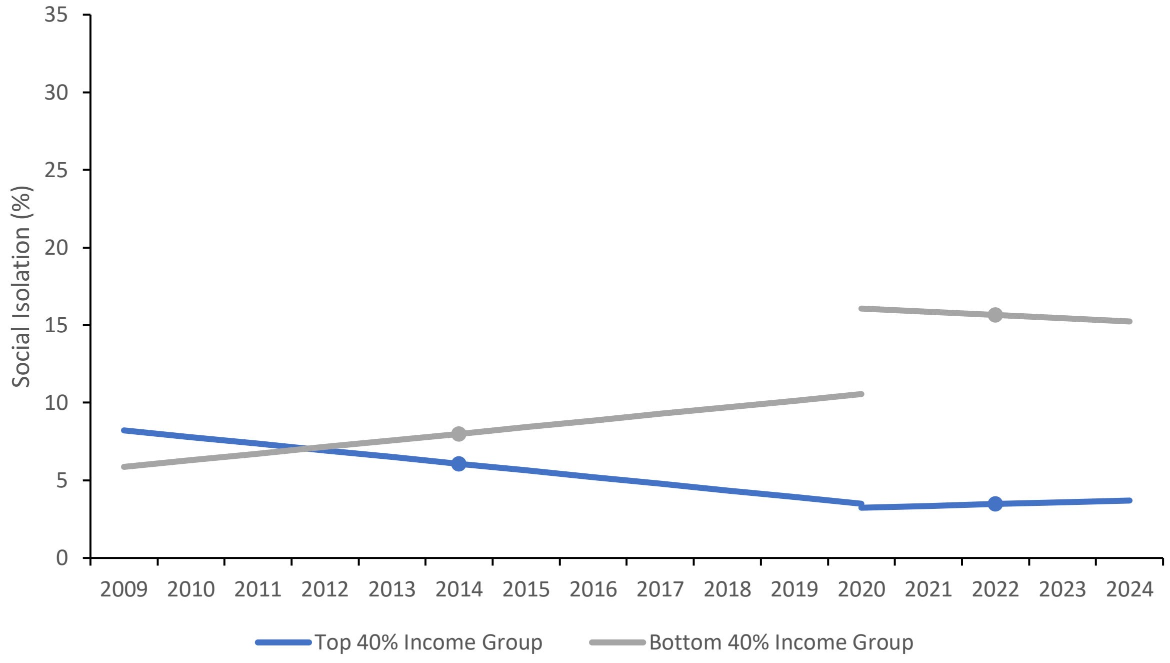

© 2025 Fuller-Rowell TE et al. *JAMA Network Open.*

eFigure 73. Trends in Social Isolation for Jordan by Income Group. Fitted trajectories are derived from empirical Bayes estimates of the final best fitting model, with raw data overlaid.

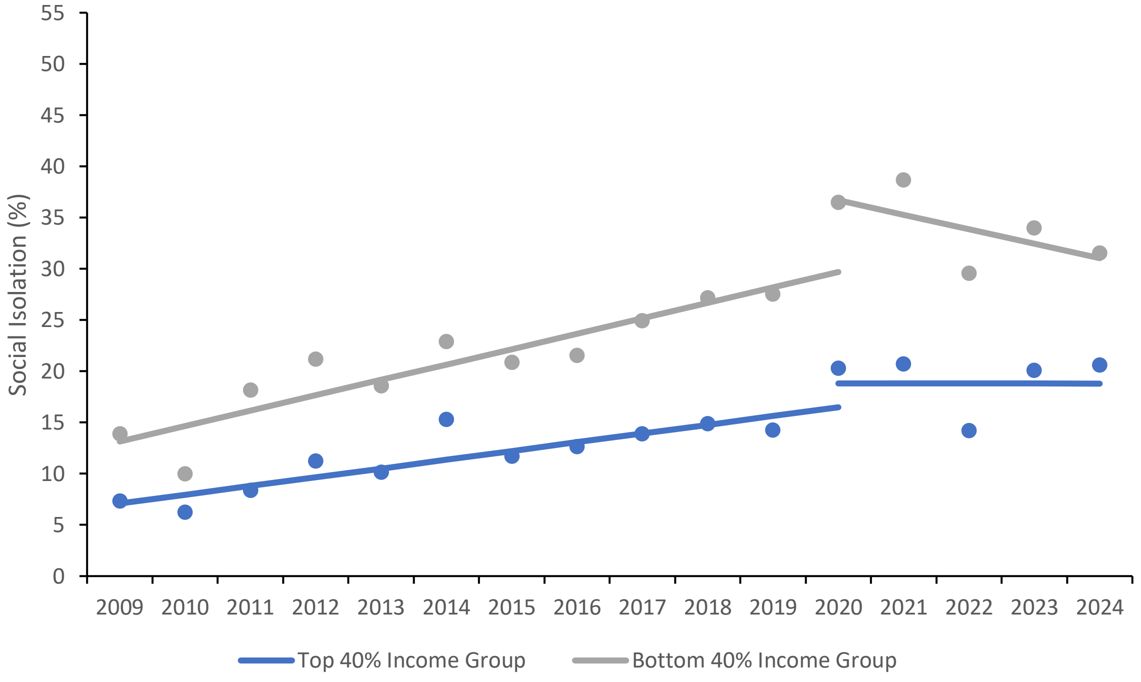

© 2025 Fuller-Rowell TE et al. *JAMA Network Open.*

eFigure 74. Trends in Social Isolation for Syria by Income Group. Fitted trajectories are derived from empirical Bayes estimates of the final best fitting model, with raw data overlaid.

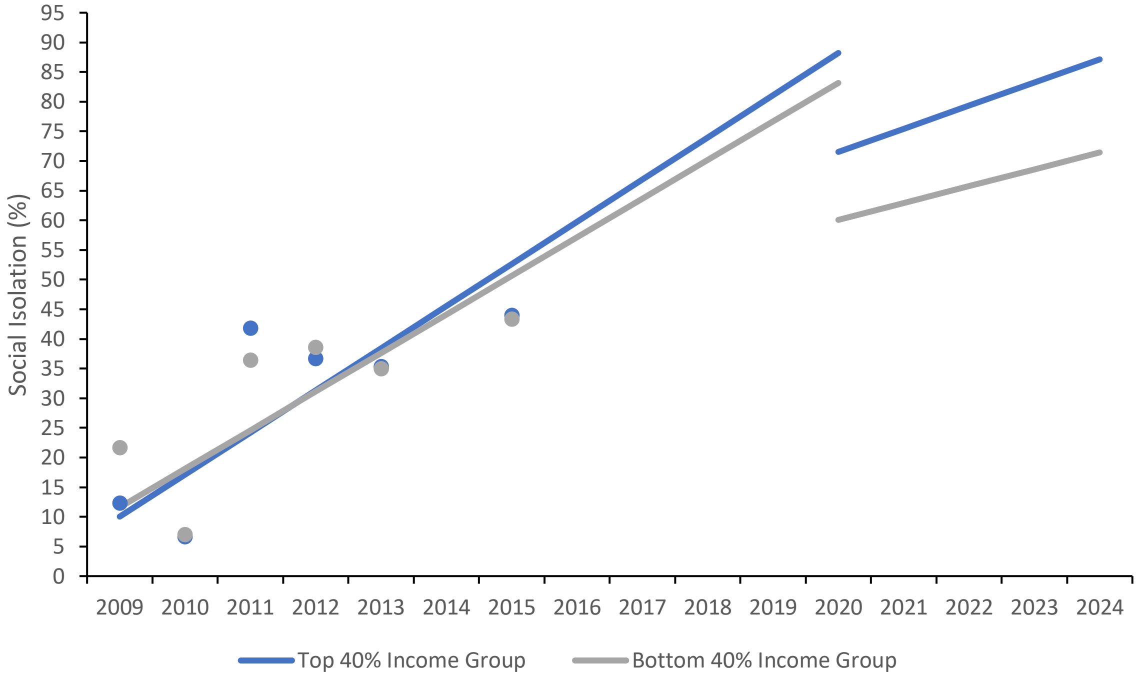

© 2025 Fuller-Rowell TE et al. *JAMA Network Open.*

eFigure 75. Trends in Social Isolation for Lebanon by Income Group. Fitted trajectories are derived from empirical Bayes estimates of the final best fitting model, with raw data overlaid.

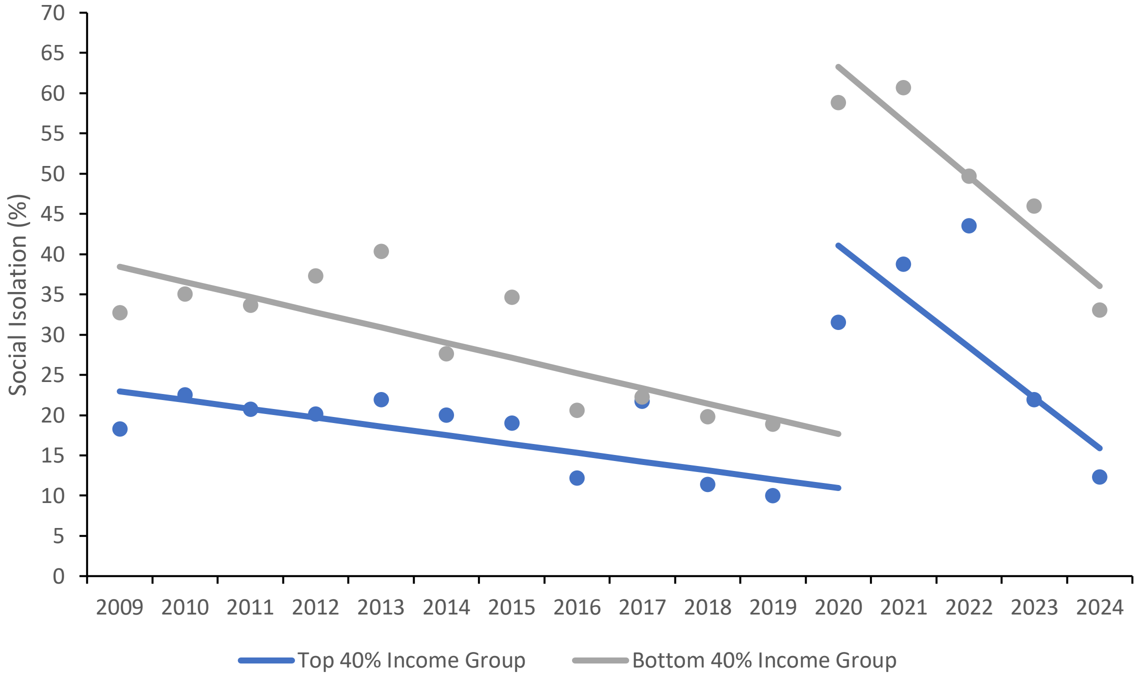

© 2025 Fuller-Rowell TE et al. *JAMA Network Open.*

eFigure 76. Trends in Social Isolation for Iraq by Income Group. Fitted trajectories are derived from empirical Bayes estimates of the final best fitting model, with raw data overlaid.

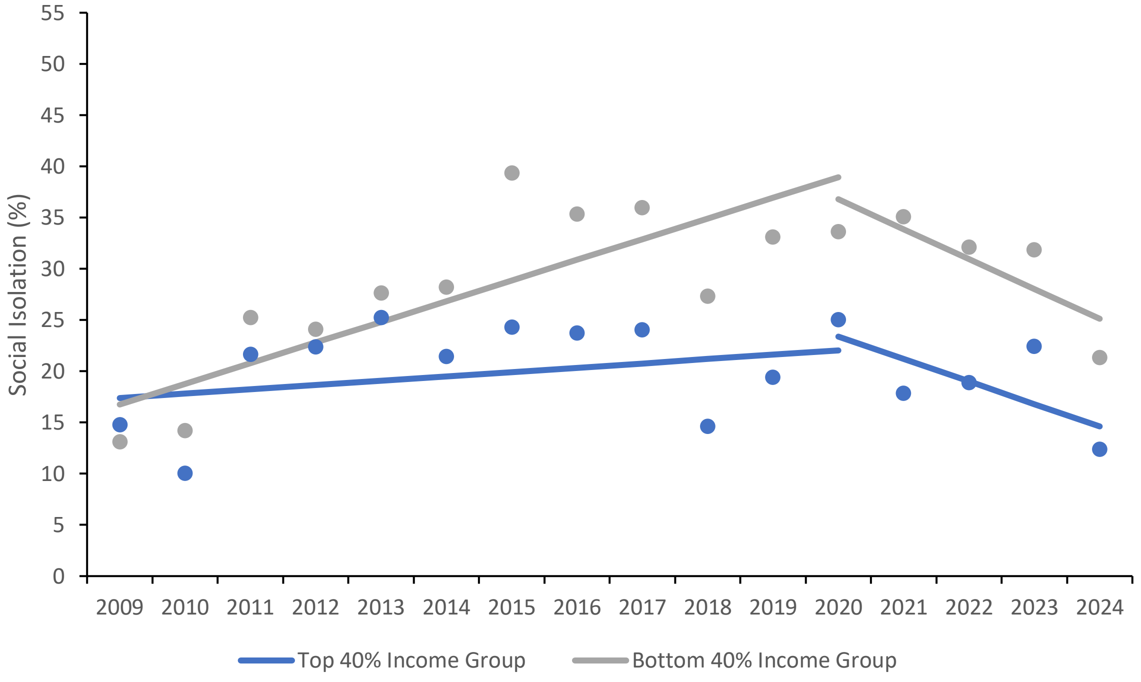

© 2025 Fuller-Rowell TE et al. *JAMA Network Open.*

eFigure 77. Trends in Social Isolation for Morocco by Income Group. Fitted trajectories are derived from empirical Bayes estimates of the final best fitting model, with raw data overlaid.

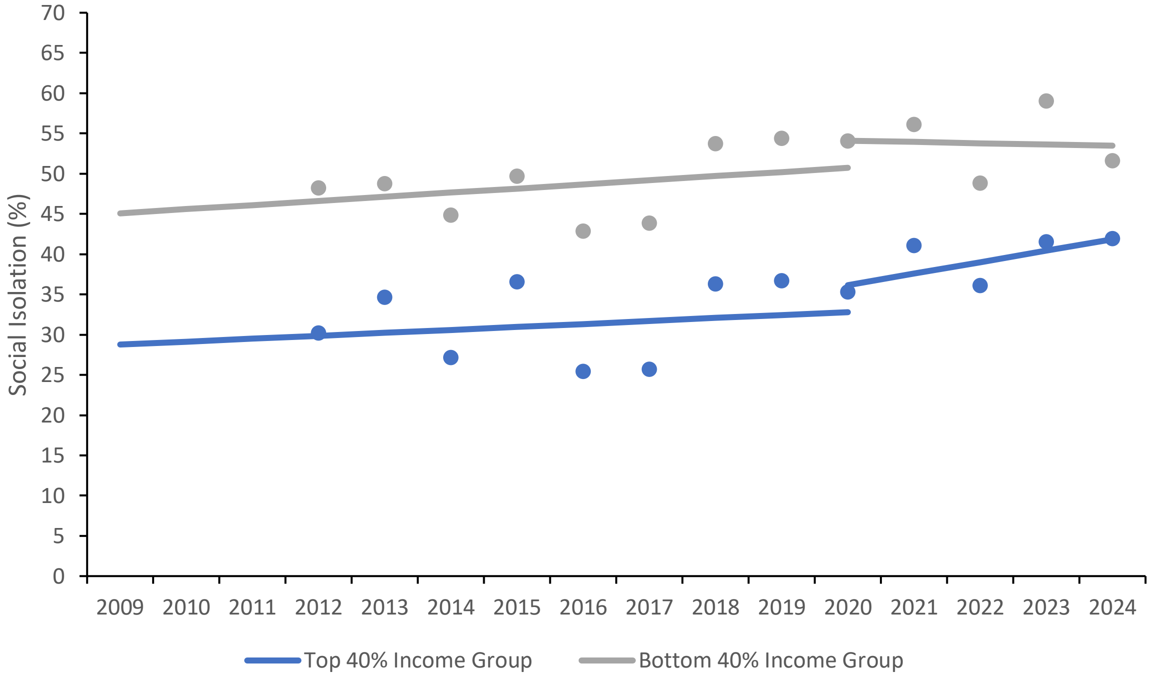

© 2025 Fuller-Rowell TE et al. *JAMA Network Open.*

eFigure 78. Trends in Social Isolation for Libya by Income Group. Fitted trajectories are derived from empirical Bayes estimates of the final best fitting model, with raw data overlaid.

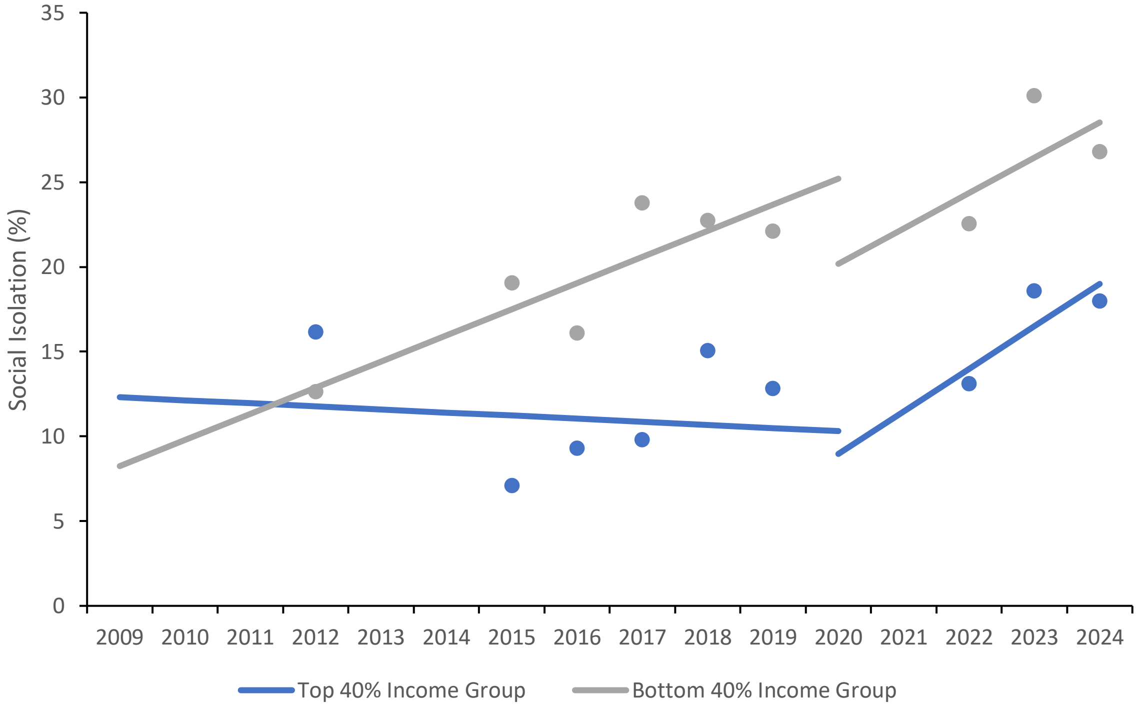

© 2025 Fuller-Rowell TE et al. *JAMA Network Open.*

eFigure 79. Trends in Social Isolation for Bahrain by Income Group. Fitted trajectories are derived from empirical Bayes estimates of the final best fitting model, with raw data overlaid.

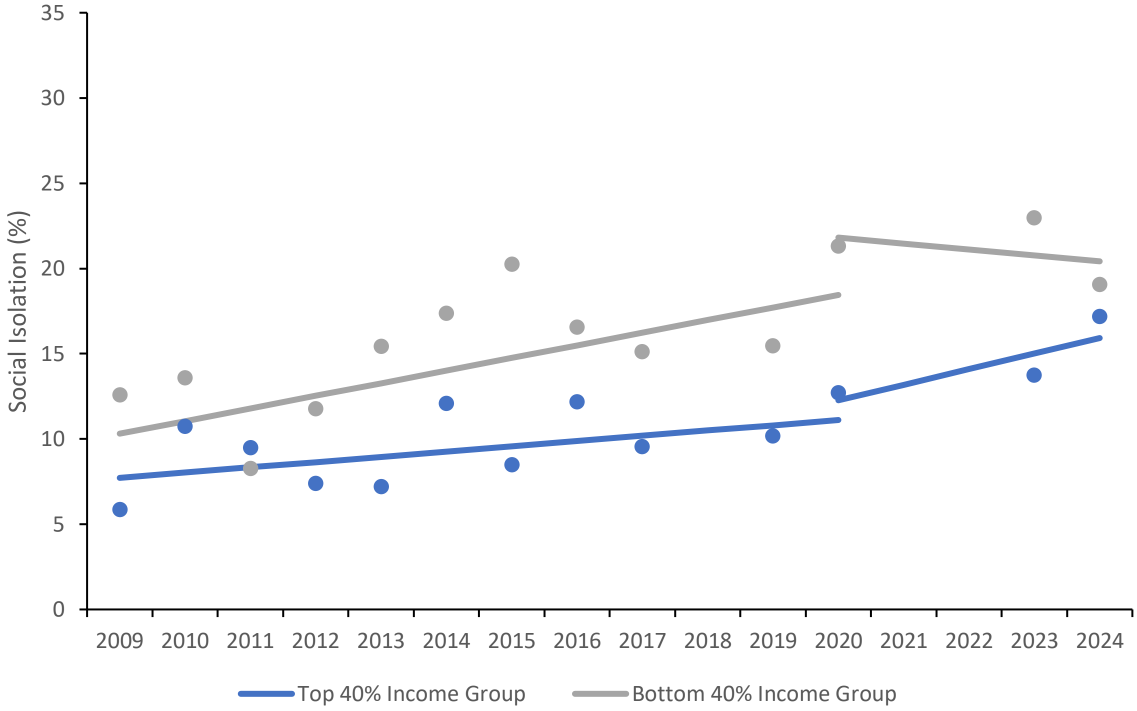

© 2025 Fuller-Rowell TE et al. *JAMA Network Open.*

eFigure 80. Trends in Social Isolation for the United Arab Emirates by Income Group. Fitted trajectories are derived from empirical Bayes estimates of the final best fitting model, with raw data overlaid.

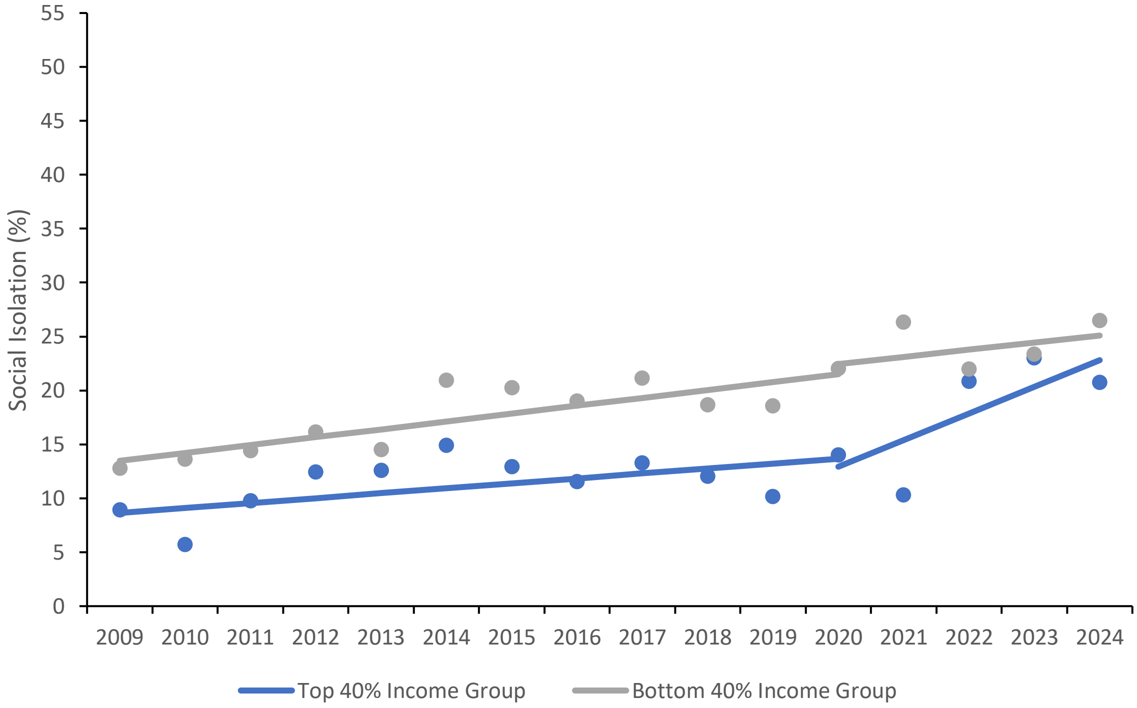

© 2025 Fuller-Rowell TE et al. *JAMA Network Open.*

eFigure 81. Trends in Social Isolation for Egypt by Income Group. Fitted trajectories are derived from empirical Bayes estimates of the final best fitting model, with raw data overlaid.

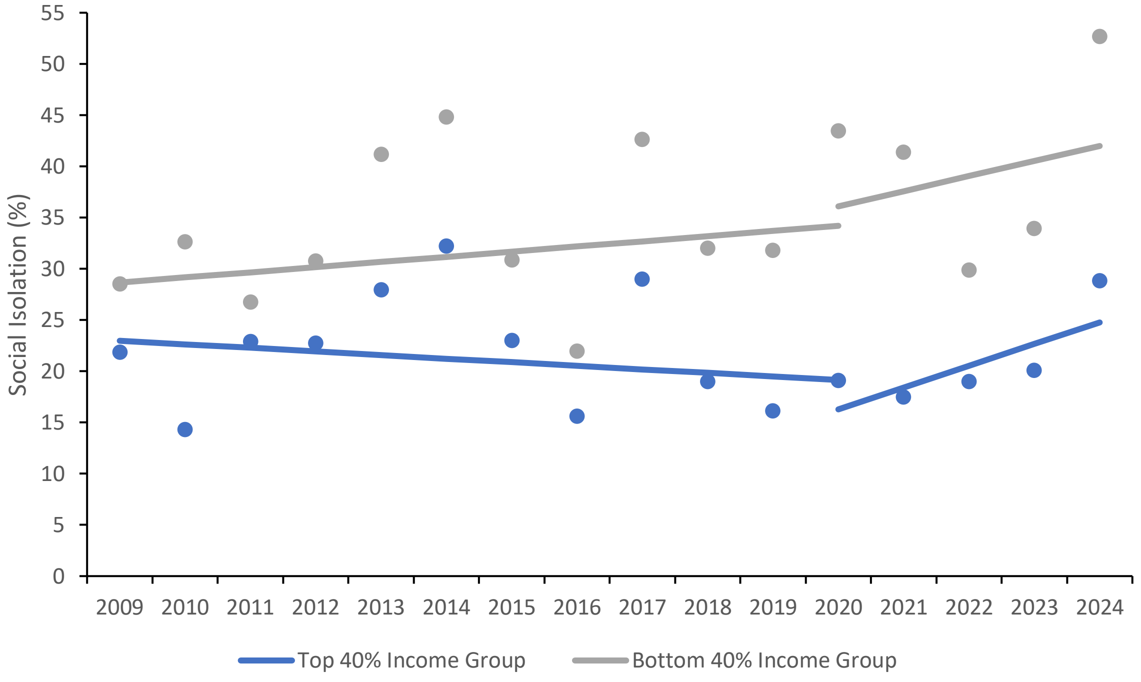

© 2025 Fuller-Rowell TE et al. *JAMA Network Open.*

eFigure 82. Trends in Social Isolation for Kuwait by Income Group. Fitted trajectories are derived from empirical Bayes estimates of the final best fitting model, with raw data overlaid.

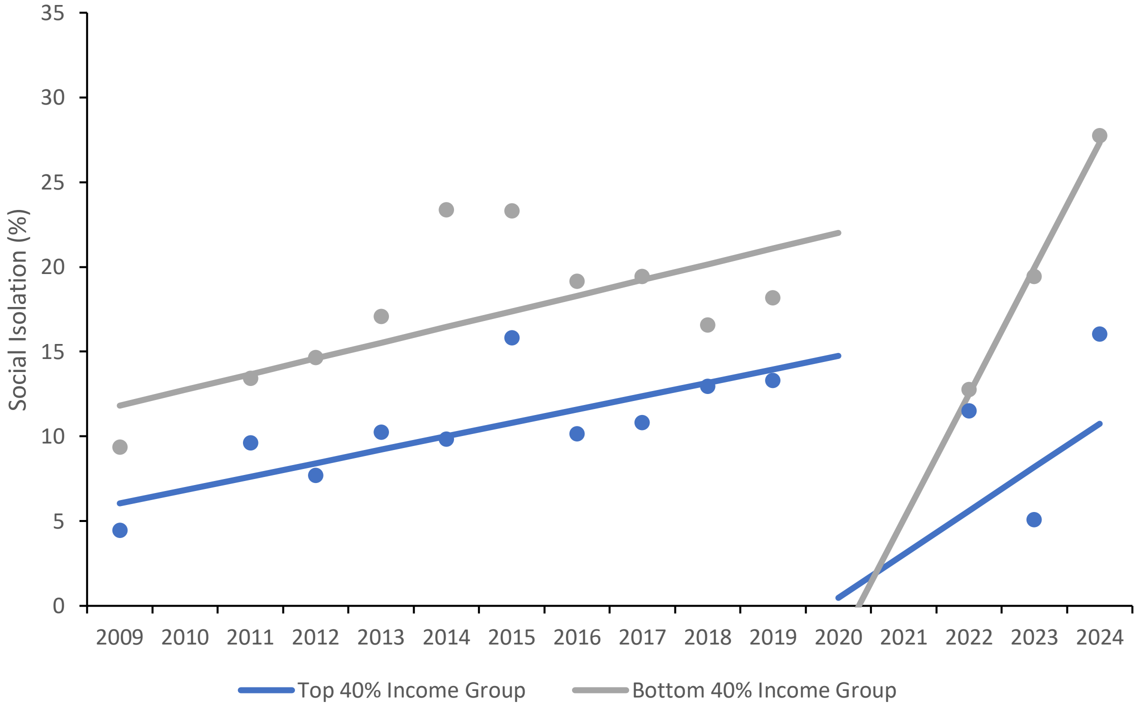

© 2025 Fuller-Rowell TE et al. *JAMA Network Open.*

eFigure 83. Trends in Social Isolation for Saudi Arabia by Income Group. Fitted trajectories are derived from empirical Bayes estimates of the final best fitting model, with raw data overlaid.

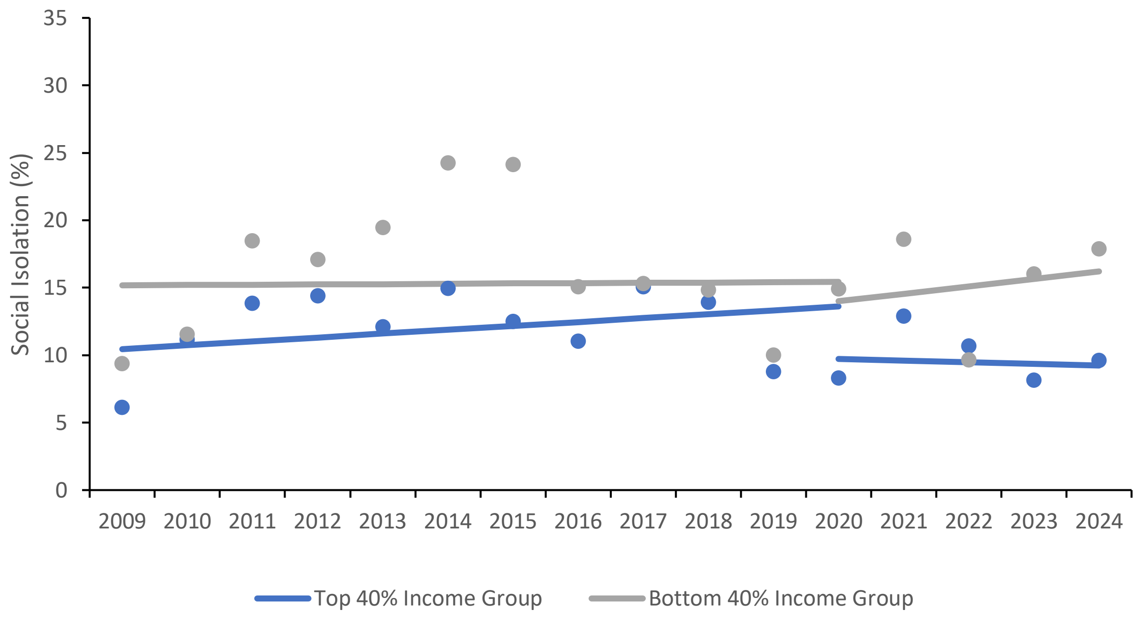

© 2025 Fuller-Rowell TE et al. *JAMA Network Open.*

eFigure 84. Trends in Social Isolation for Tunisia by Income Group. Fitted trajectories are derived from empirical Bayes estimates of the final best fitting model, with raw data overlaid.

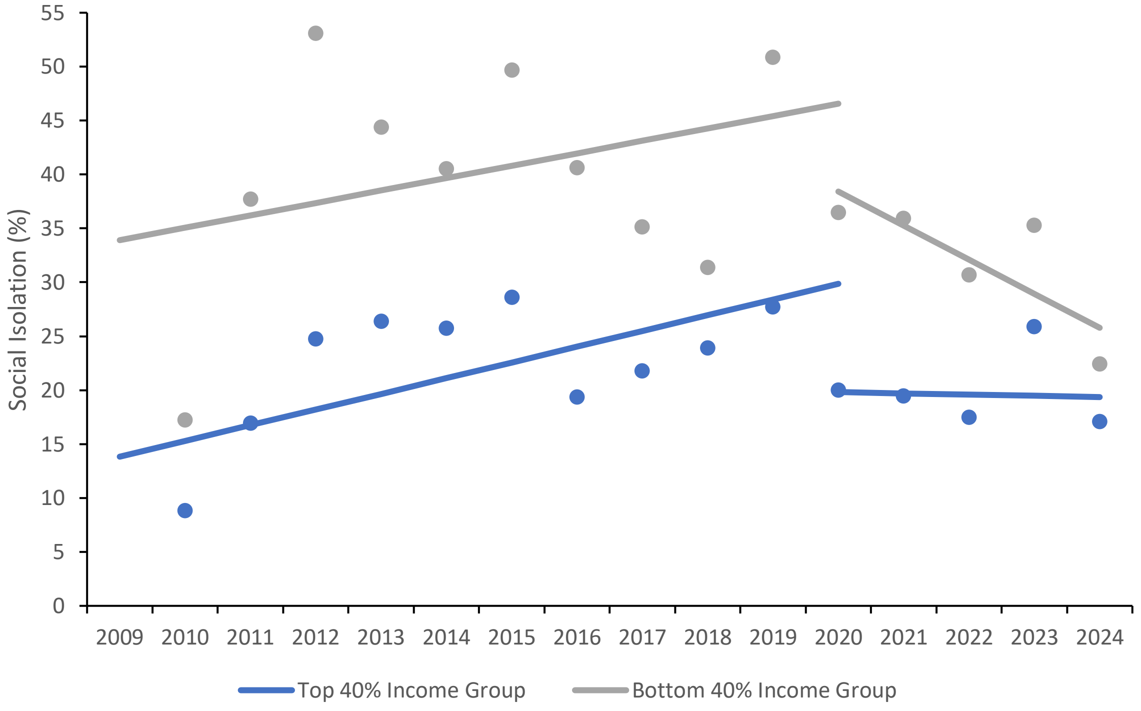

© 2025 Fuller-Rowell TE et al. *JAMA Network Open.*

eFigure 85. Trends in Social Isolation for Israel by Income Group. Fitted trajectories are derived from empirical Bayes estimates of the final best fitting model, with raw data overlaid.

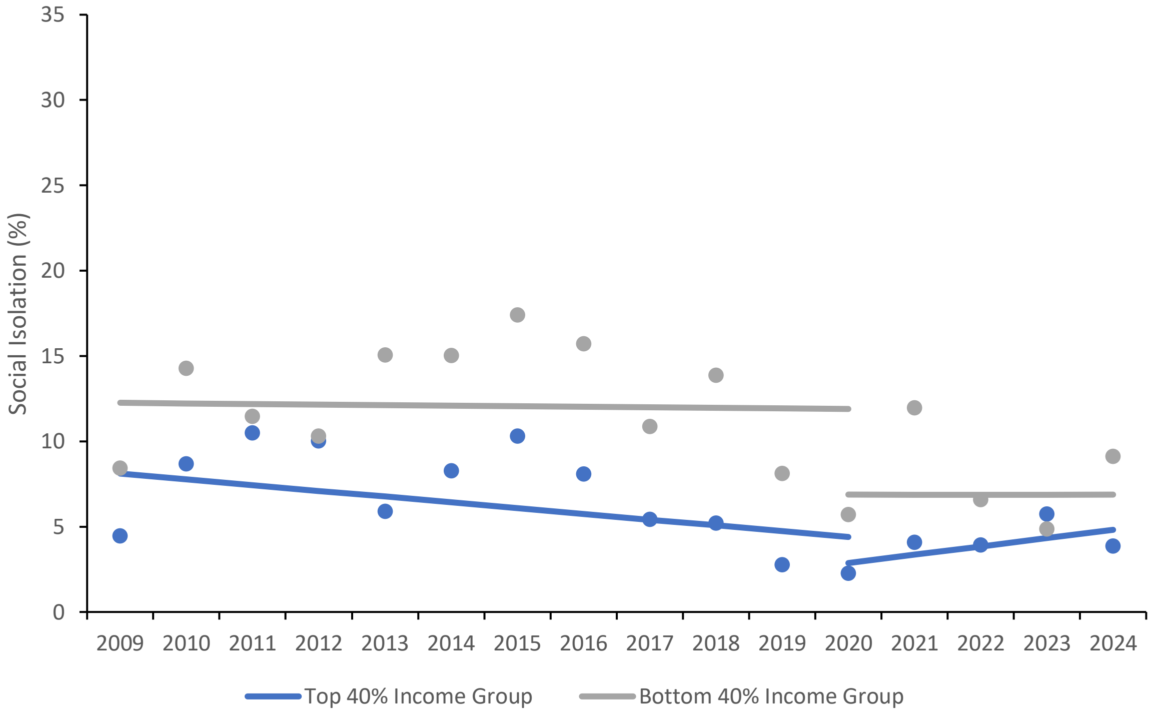

© 2025 Fuller-Rowell TE et al. *JAMA Network Open.*

eFigure 86. Trends in Social Isolation for Qatar by Income Group. Fitted trajectories are derived from empirical Bayes estimates of the final best fitting model, with raw data overlaid.

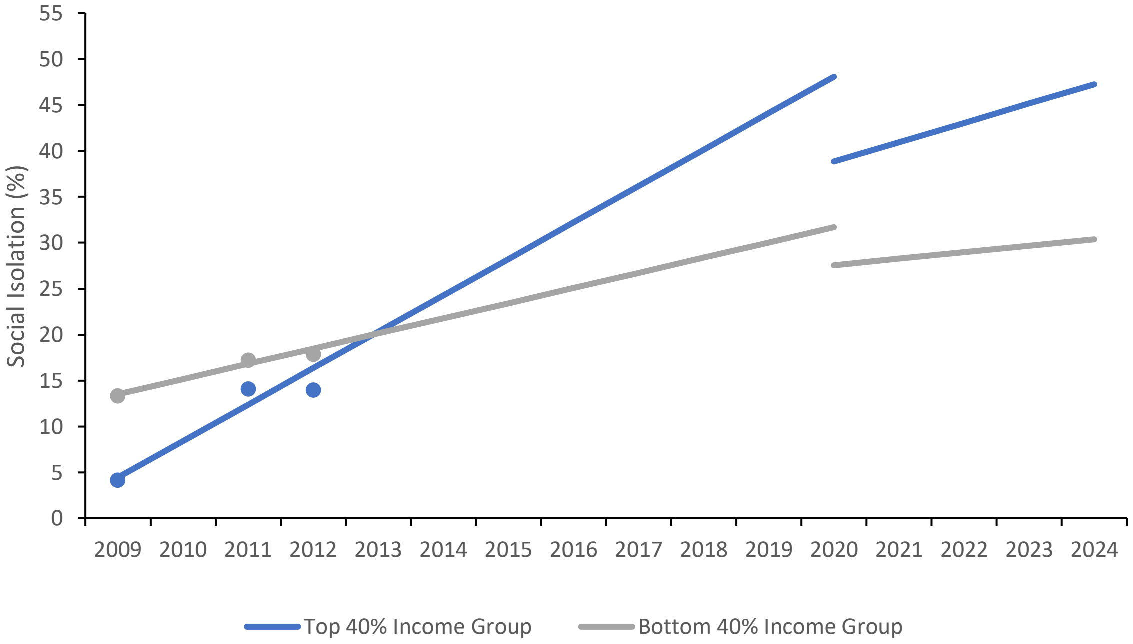

© 2025 Fuller-Rowell TE et al. *JAMA Network Open.*

eFigure 87. Trends in Social Isolation for Türkiye by Income Group. Fitted trajectories are derived from empirical Bayes estimates of the final best fitting model, with raw data overlaid.

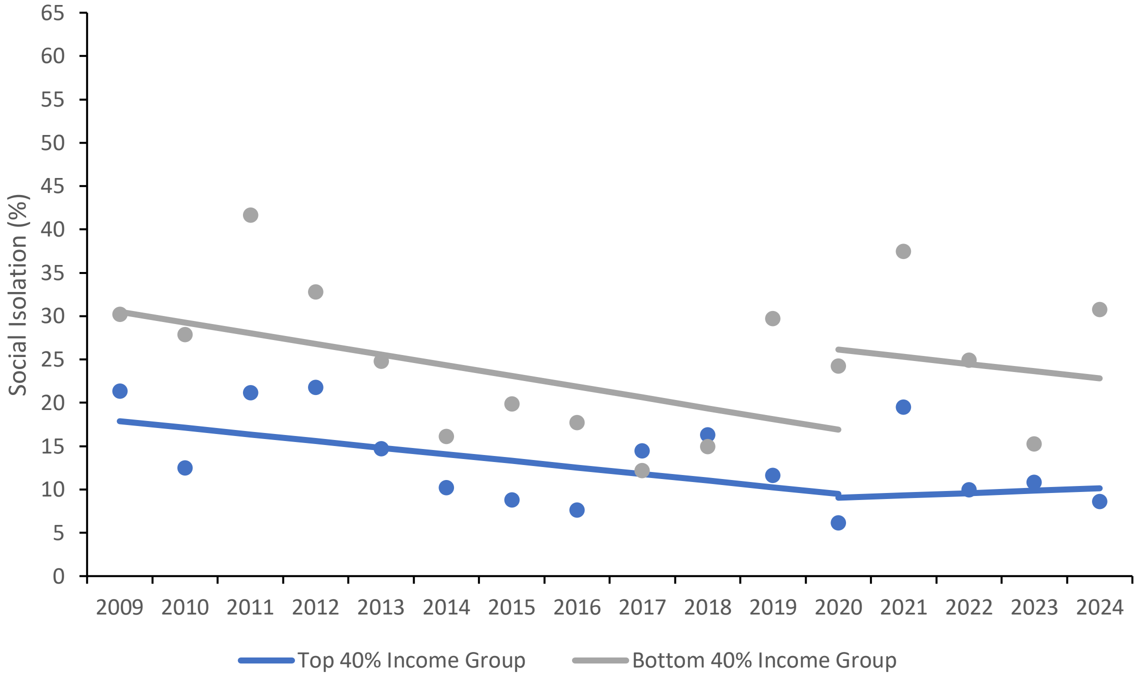

© 2025 Fuller-Rowell TE et al. *JAMA Network Open.*

eFigure 88. Trends in Social Isolation for the State of Palestine by Income Group. Fitted trajectories are derived from empirical Bayes estimates of the final best fitting model, with raw data overlaid.

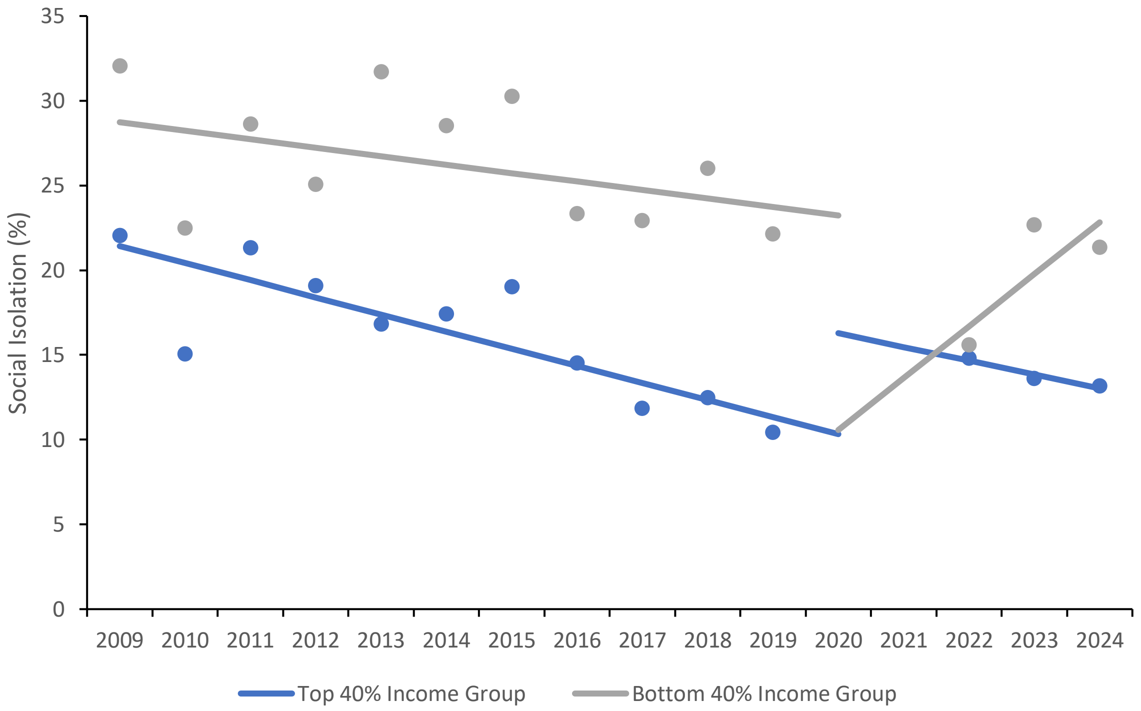

© 2025 Fuller-Rowell TE et al. *JAMA Network Open.*

eFigure 89. Trends in Social Isolation for Algeria by Income Group. Fitted trajectories are derived from empirical Bayes estimates of the final best fitting model, with raw data overlaid.

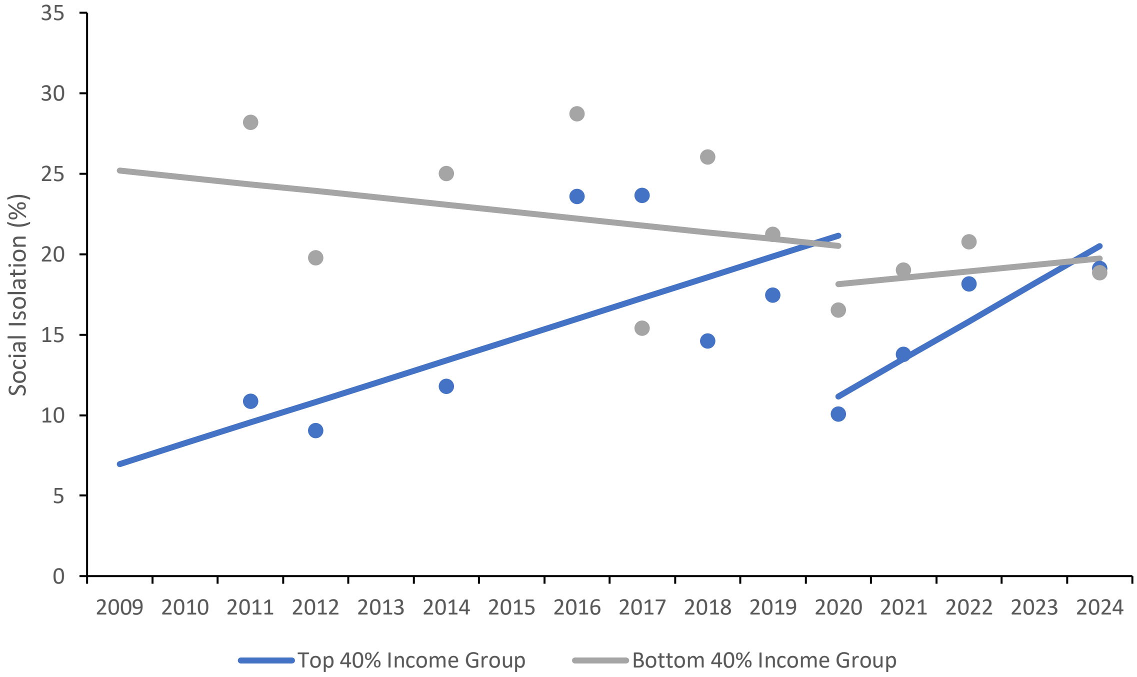

© 2025 Fuller-Rowell TE et al. *JAMA Network Open.*

eFigure 90. Trends in Social Isolation for Iran by Income Group. Fitted trajectories are derived from empirical Bayes estimates of the final best fitting model, with raw data overlaid.

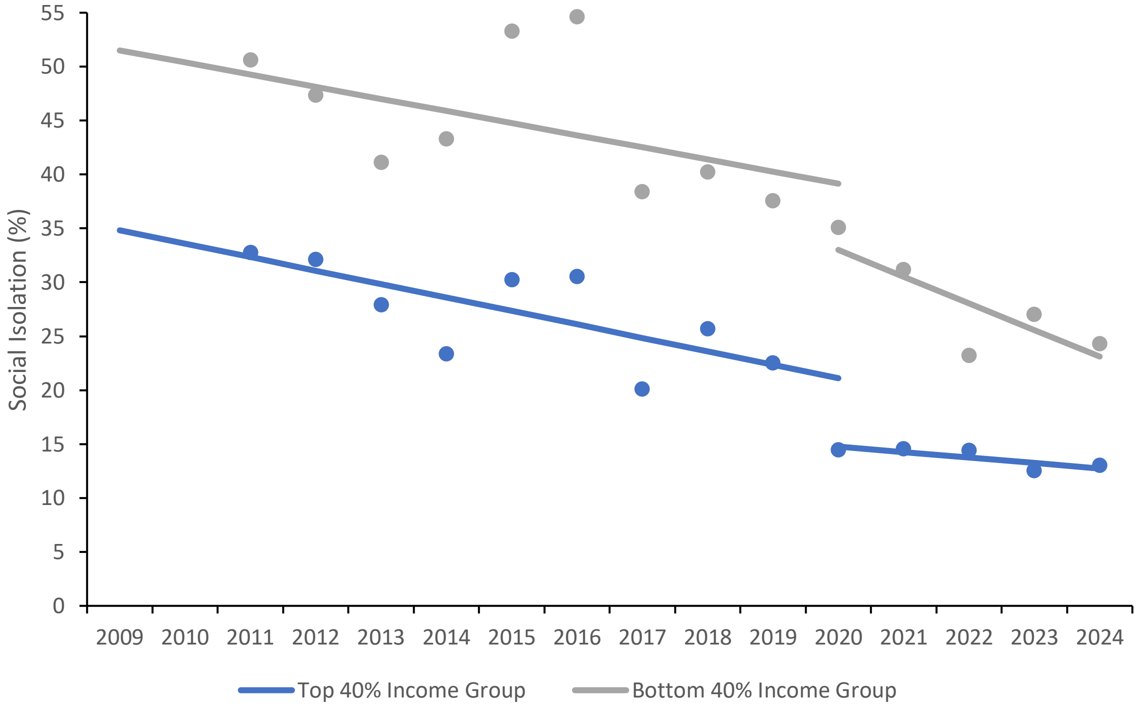

© 2025 Fuller-Rowell TE et al. *JAMA Network Open.*

eFigure 91. Trends in Social Isolation for Yemen by Income Group. Fitted trajectories are derived from empirical Bayes estimates of the final best fitting model, with raw data overlaid.

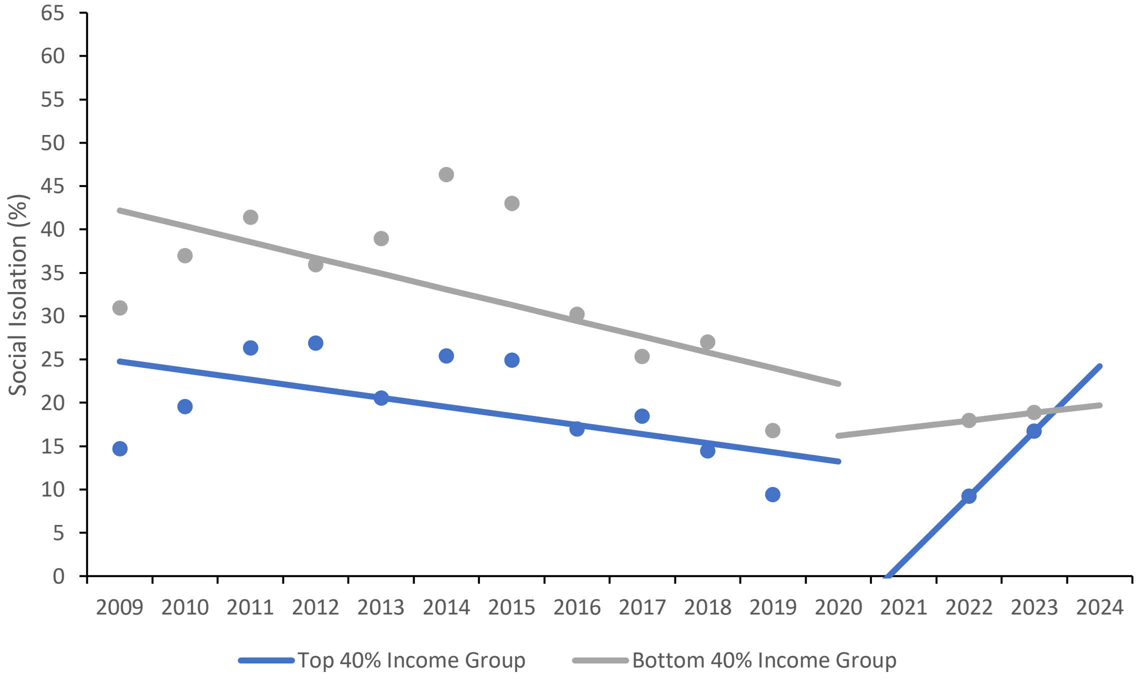

© 2025 Fuller-Rowell TE et al. *JAMA Network Open.*

eFigure 92. Trends in Social Isolation for Canada by Income Group. Fitted trajectories are derived from empirical Bayes estimates of the final best fitting model, with raw data overlaid.

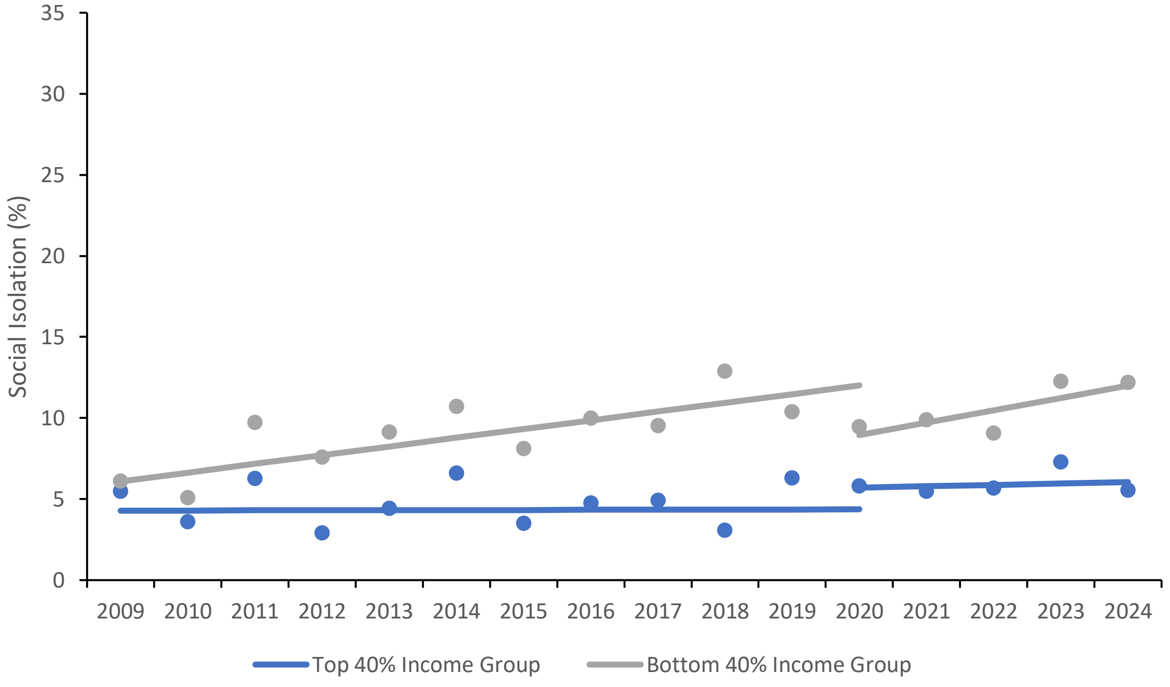

© 2025 Fuller-Rowell TE et al. *JAMA Network Open.*

eFigure 93. Trends in Social Isolation for the United States of America by Income Group. Fitted trajectories are derived from empirical Bayes estimates of the final best fitting model, with raw data overlaid.

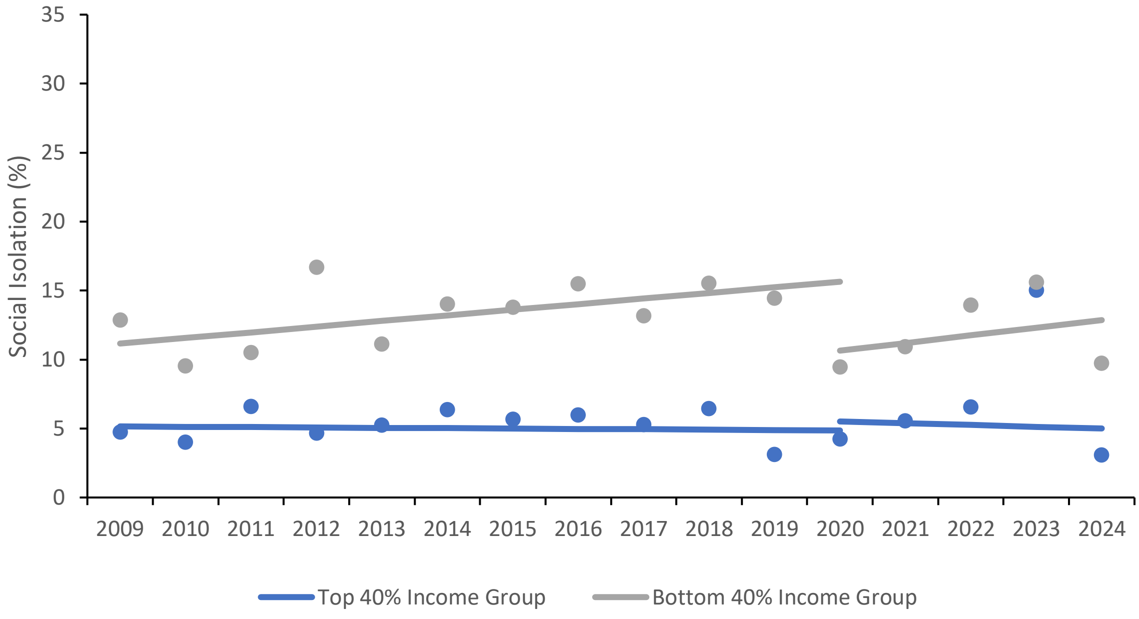

© 2025 Fuller-Rowell TE et al. *JAMA Network Open.*

eFigure 94. Trends in Social Isolation for New Zealand by Income Group. Fitted trajectories are derived from empirical Bayes estimates of the final best fitting model, with raw data overlaid.

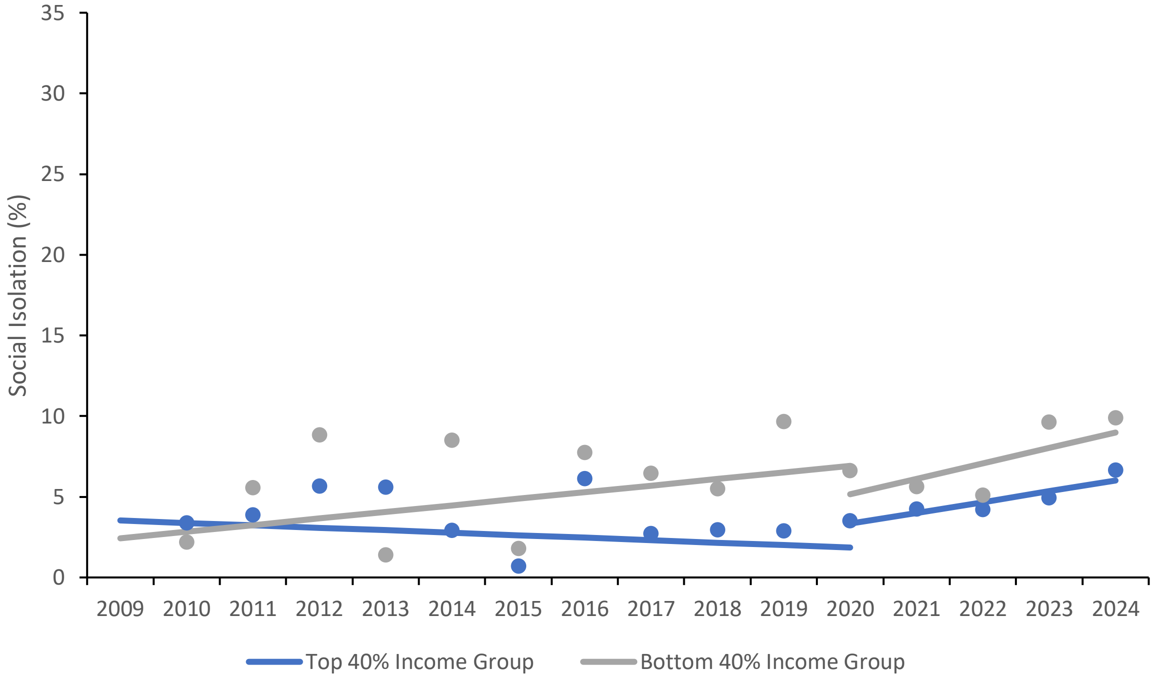

© 2025 Fuller-Rowell TE et al. *JAMA Network Open.*

eFigure 95. Trends in Social Isolation for Australia by Income Group. Fitted trajectories are derived from empirical Bayes estimates of the final best fitting model, with raw data overlaid.

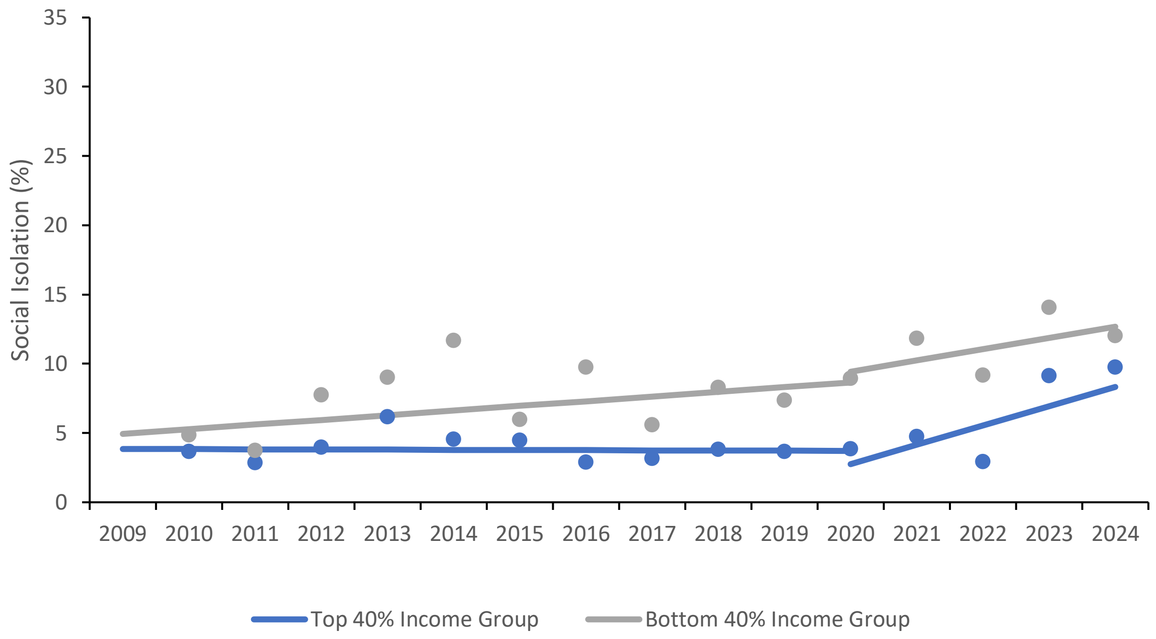

© 2025 Fuller-Rowell TE et al. *JAMA Network Open.*

eFigure 96. Trends in Social Isolation for Thailand by Income Group. Fitted trajectories are derived from empirical Bayes estimates of the final best fitting model, with raw data overlaid.

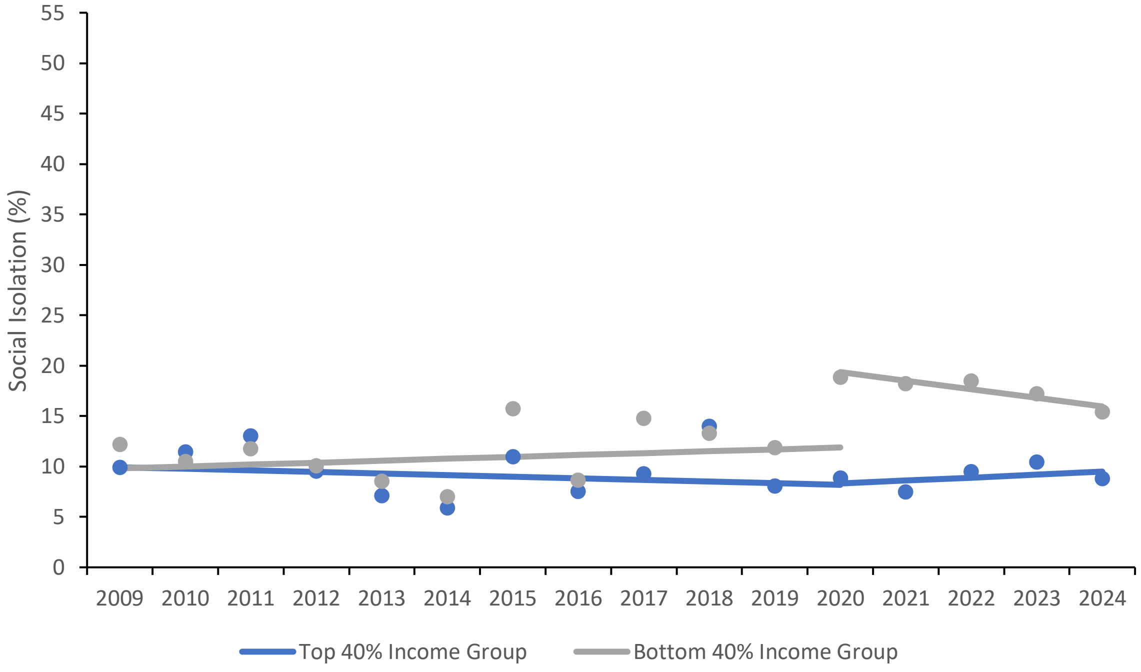

© 2025 Fuller-Rowell TE et al. *JAMA Network Open.*

eFigure 97. Trends in Social Isolation for Malaysia by Income Group. Fitted trajectories are derived from empirical Bayes estimates of the final best fitting model, with raw data overlaid.

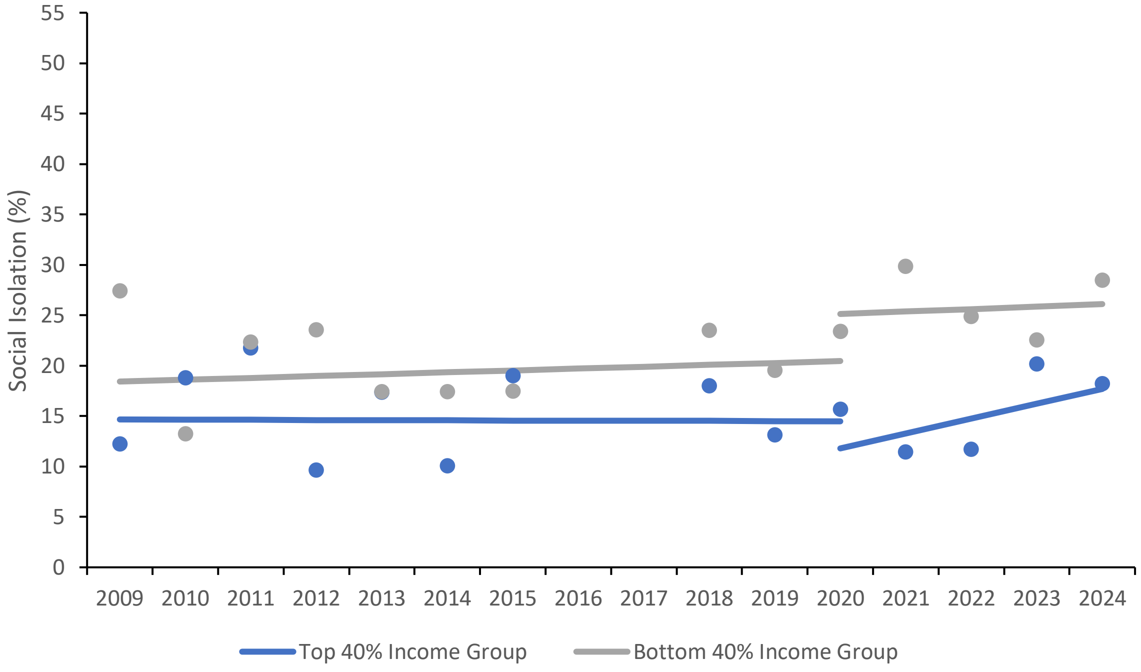

© 2025 Fuller-Rowell TE et al. *JAMA Network Open.*

eFigure 98. Trends in Social Isolation for Indonesia by Income Group. Fitted trajectories are derived from empirical Bayes estimates of the final best fitting model, with raw data overlaid.

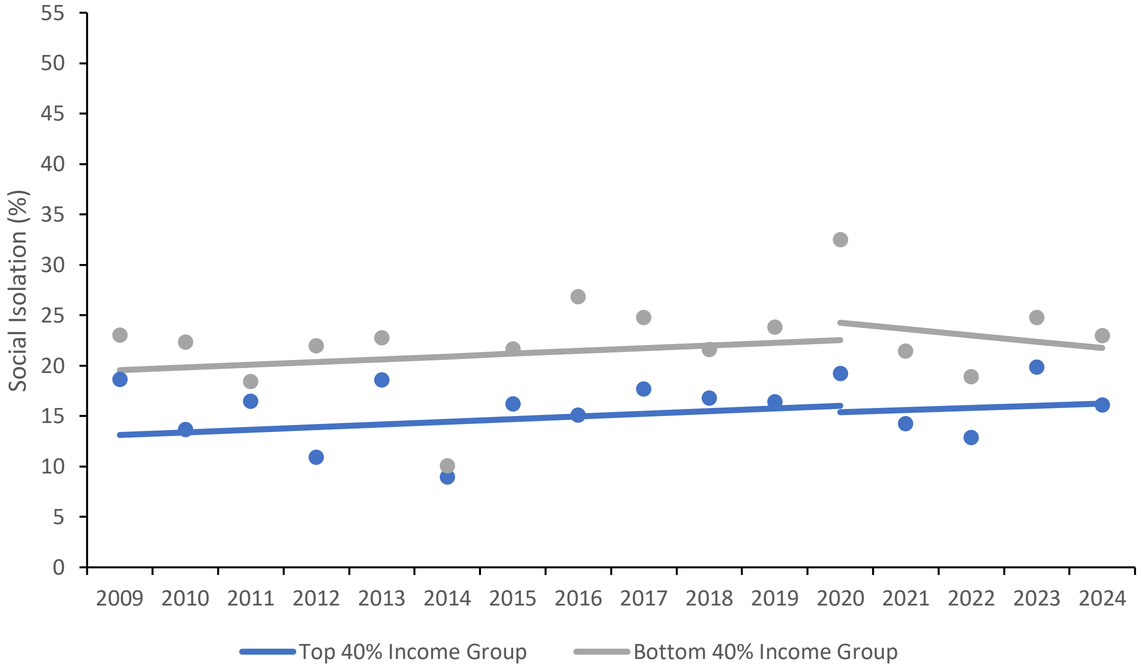

© 2025 Fuller-Rowell TE et al. *JAMA Network Open.*

eFigure 99. Trends in Social Isolation for Laos by Income Group. Fitted trajectories are derived from empirical Bayes estimates of the final best fitting model, with raw data overlaid.

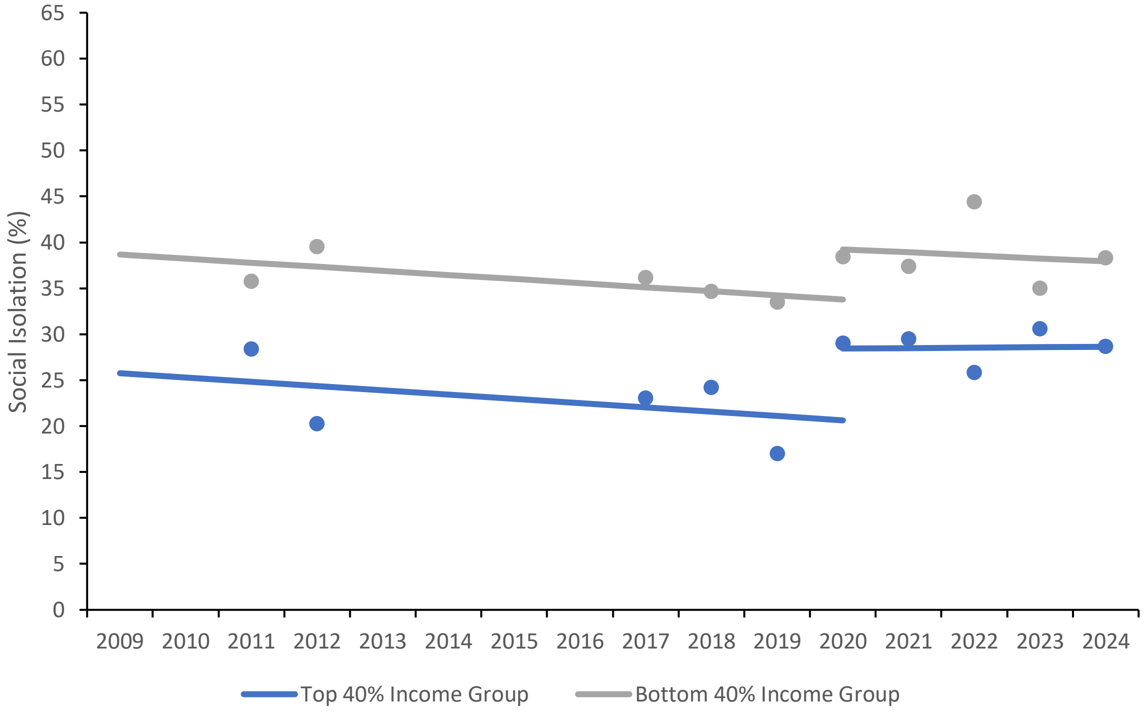

© 2025 Fuller-Rowell TE et al. *JAMA Network Open.*

eFigure 100. Trends in Social Isolation for Vietnam by Income Group. Fitted trajectories are derived from empirical Bayes estimates of the final best fitting model, with raw data overlaid.

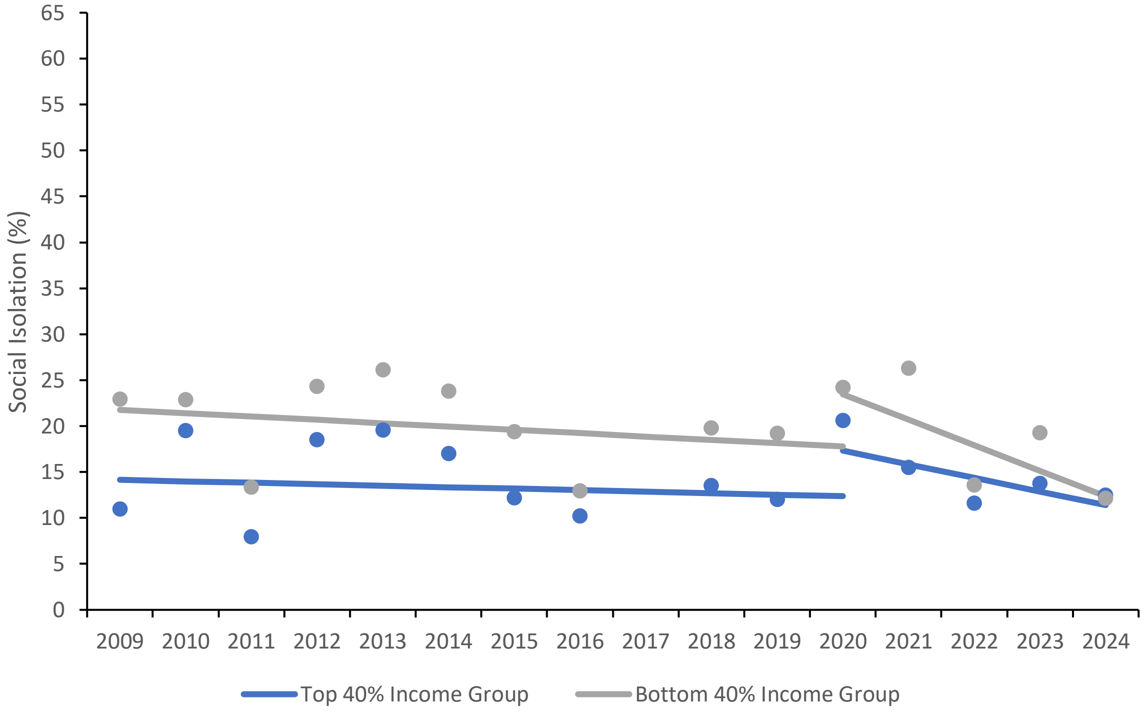

© 2025 Fuller-Rowell TE et al. *JAMA Network Open.*

eFigure 101. Trends in Social Isolation for Cambodia by Income Group. Fitted trajectories are derived from empirical Bayes estimates of the final best fitting model, with raw data overlaid.

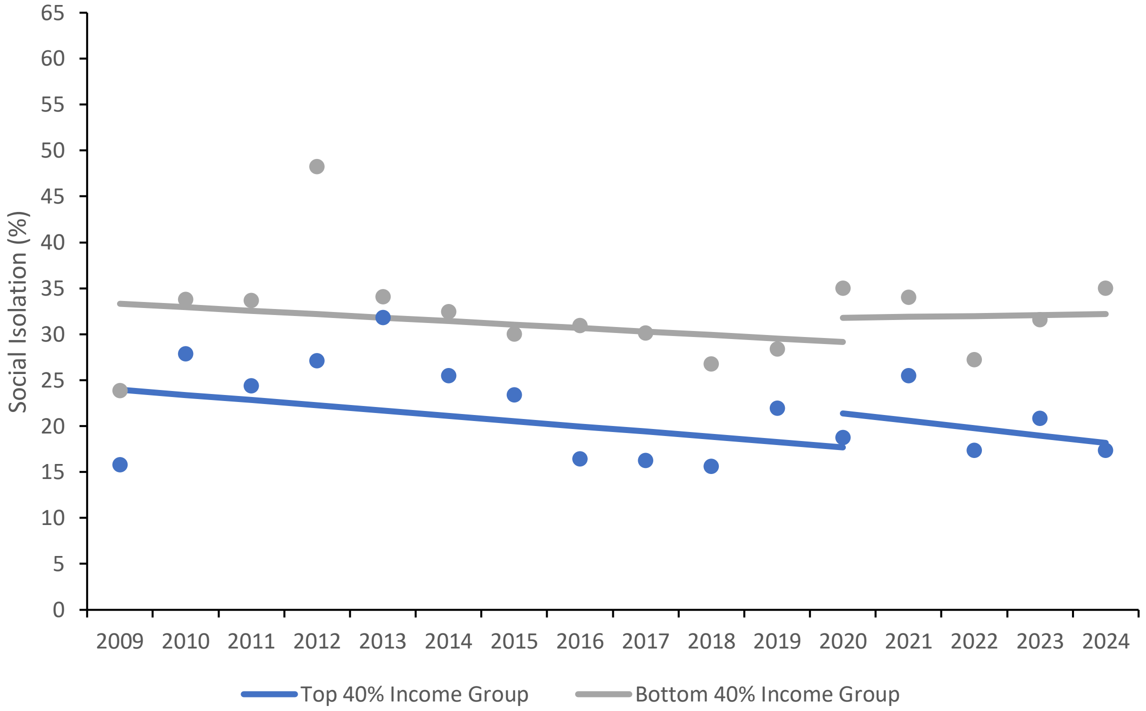

© 2025 Fuller-Rowell TE et al. *JAMA Network Open.*

eFigure 102. Trends in Social Isolation for Singapore by Income Group. Fitted trajectories are derived from empirical Bayes estimates of the final best fitting model, with raw data overlaid

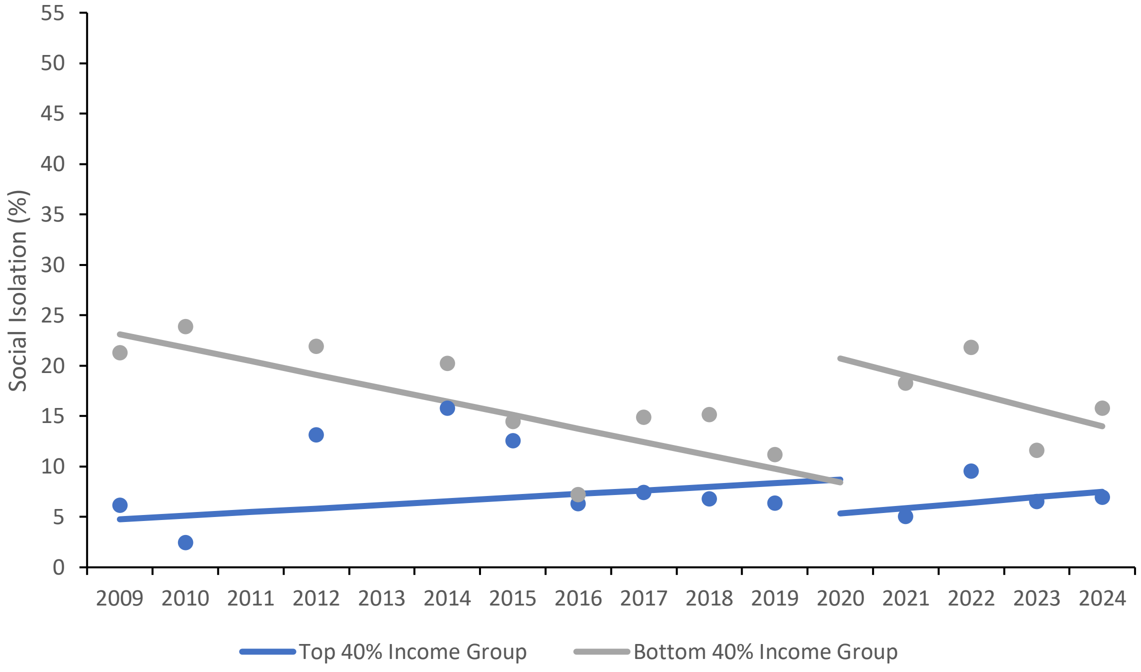

© 2025 Fuller-Rowell TE et al. *JAMA Network Open.*

eFigure 103. Trends in Social Isolation for Myanmar by Income Group. Fitted trajectories are derived from empirical Bayes estimates of the final best fitting model, with raw data overlaid.

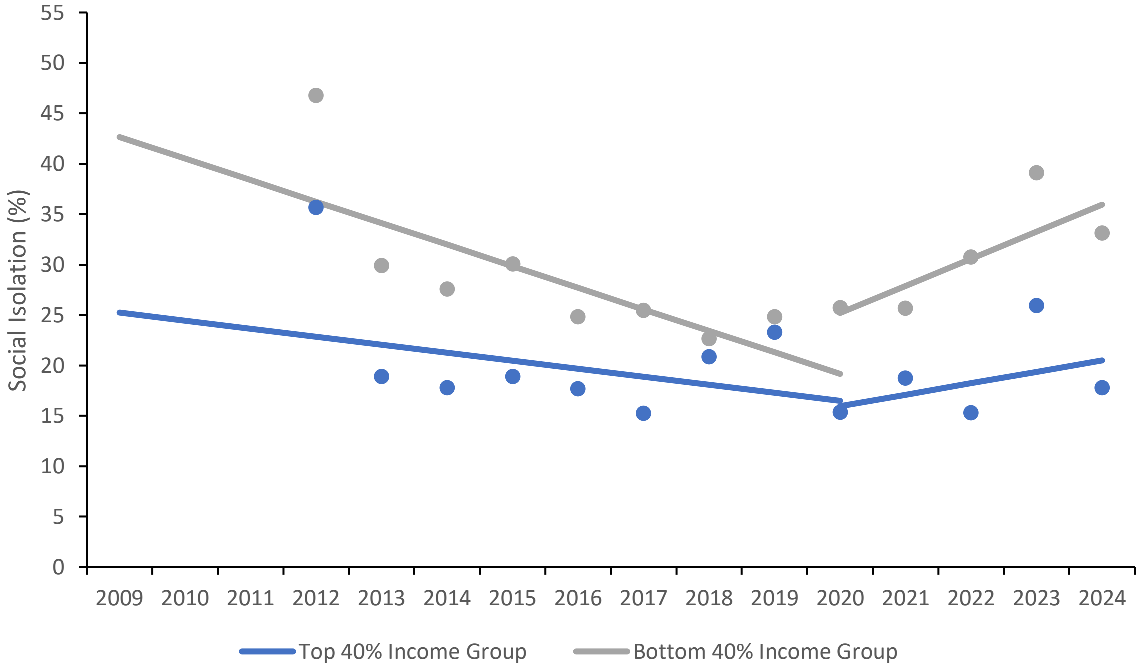

© 2025 Fuller-Rowell TE et al. *JAMA Network Open.*

eFigure 104. Trends in Social Isolation for the Philippines by Income Group. Fitted trajectories are derived from empirical Bayes estimates of the final best fitting model, with raw data overlaid.

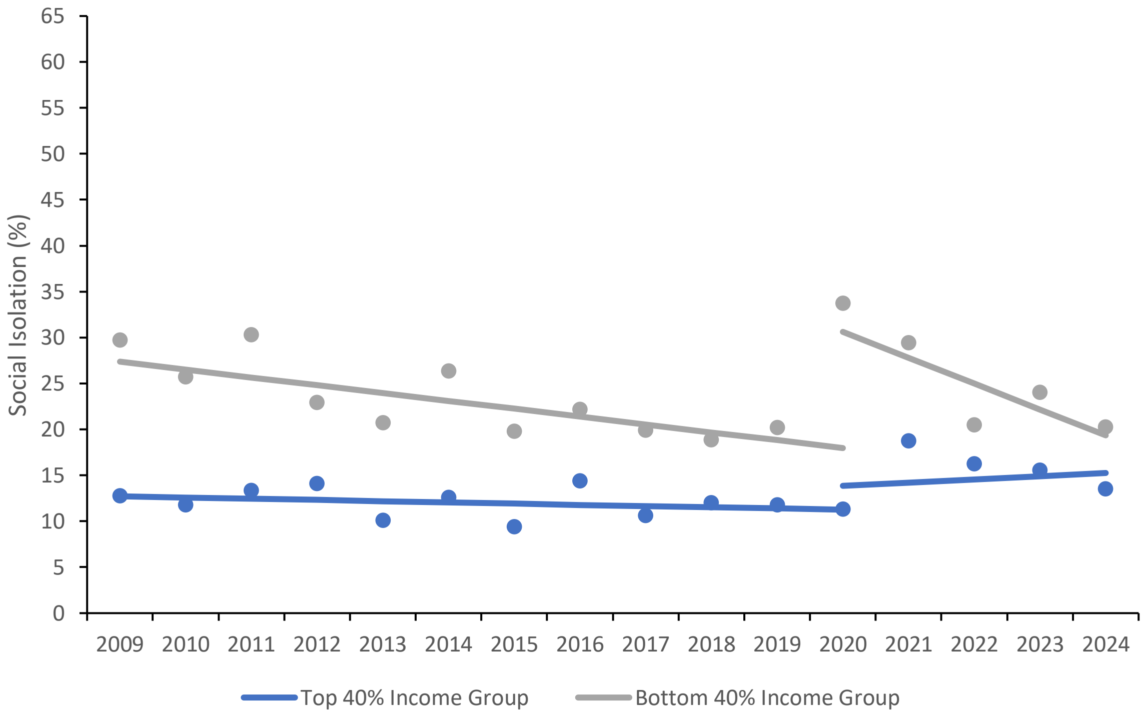

© 2025 Fuller-Rowell TE et al. *JAMA Network Open.*

eFigure 105. Trends in Social Isolation for Albania by Income Group. Fitted trajectories are derived from empirical Bayes estimates of the final best fitting model, with raw data overlaid.

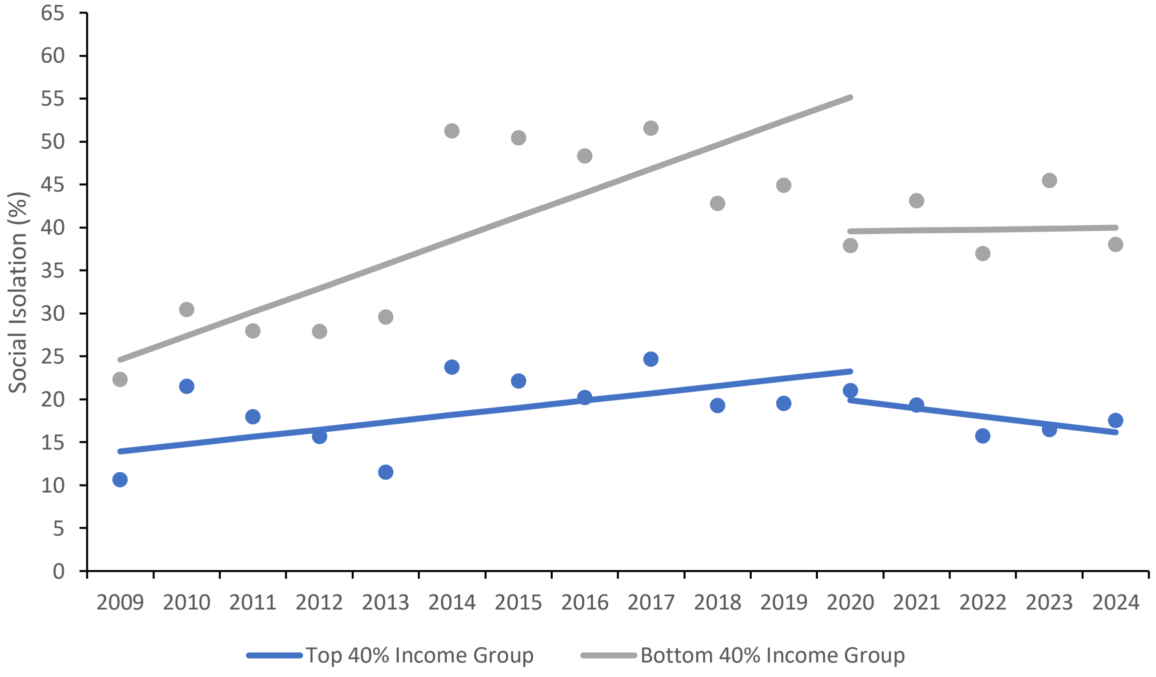

© 2025 Fuller-Rowell TE et al. *JAMA Network Open.*

eFigure 106. Trends in Social Isolation for Luxembourg by Income Group. Fitted trajectories are derived from empirical Bayes estimates of the final best fitting model, with raw data overlaid.

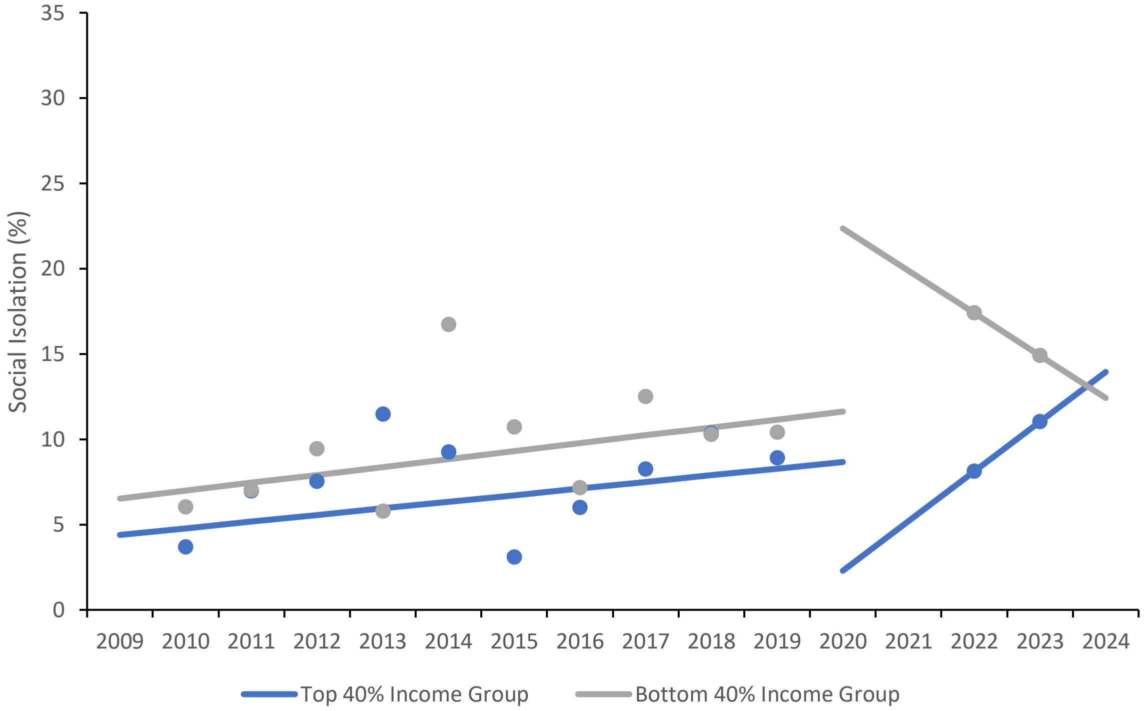

© 2025 Fuller-Rowell TE et al. *JAMA Network Open.*

eFigure 107. Trends in Social Isolation for Austria by Income Group. Fitted trajectories are derived from empirical Bayes estimates of the final best fitting model, with raw data overlaid.

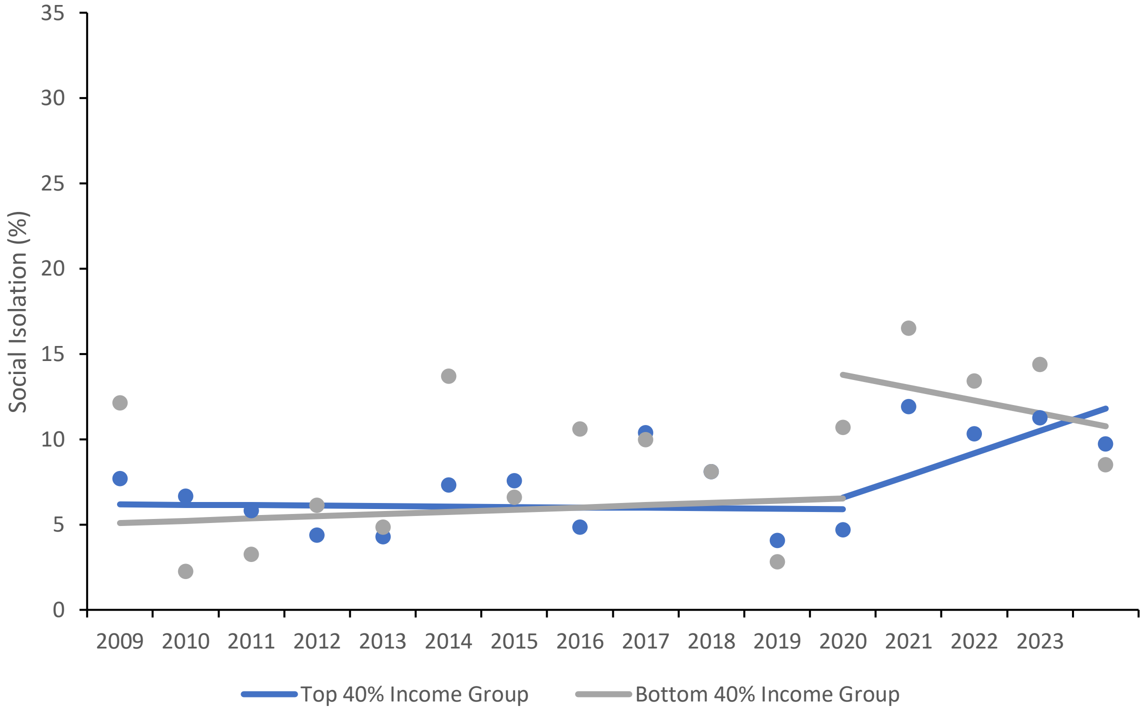

© 2025 Fuller-Rowell TE et al. *JAMA Network Open.*

eFigure 108. Trends in Social Isolation for Ireland by Income Group. Fitted trajectories are derived from empirical Bayes estimates of the final best fitting model, with raw data overlaid.

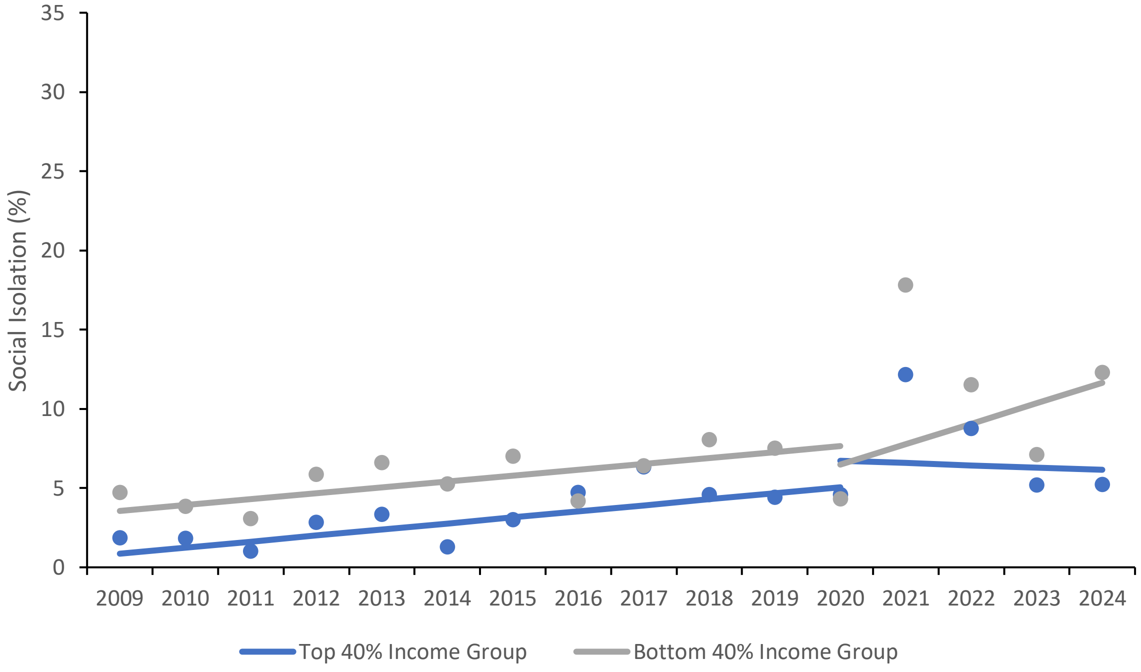

© 2025 Fuller-Rowell TE et al. *JAMA Network Open.*

eFigure 109. Trends in Social Isolation for Germany by Income Group. Fitted trajectories are derived from empirical Bayes estimates of the final best fitting model, with raw data overlaid.

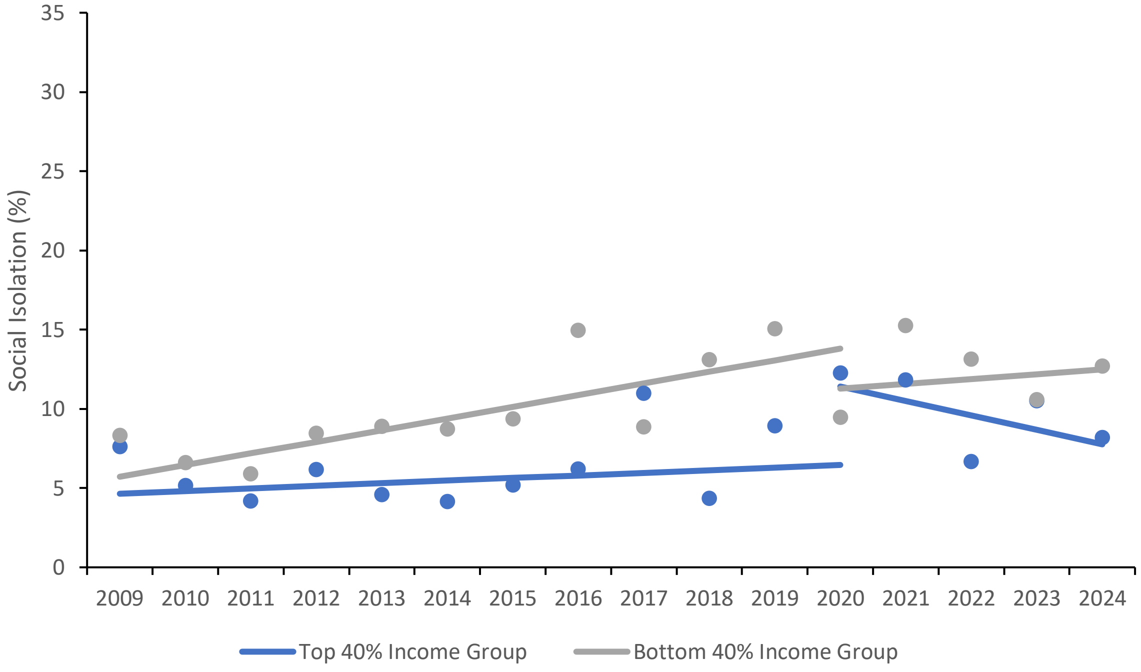

© 2025 Fuller-Rowell TE et al. *JAMA Network Open.*

eFigure 110. Trends in Social Isolation for Belgium by Income Group. Fitted trajectories are derived from empirical Bayes estimates of the final best fitting model, with raw data overlaid.

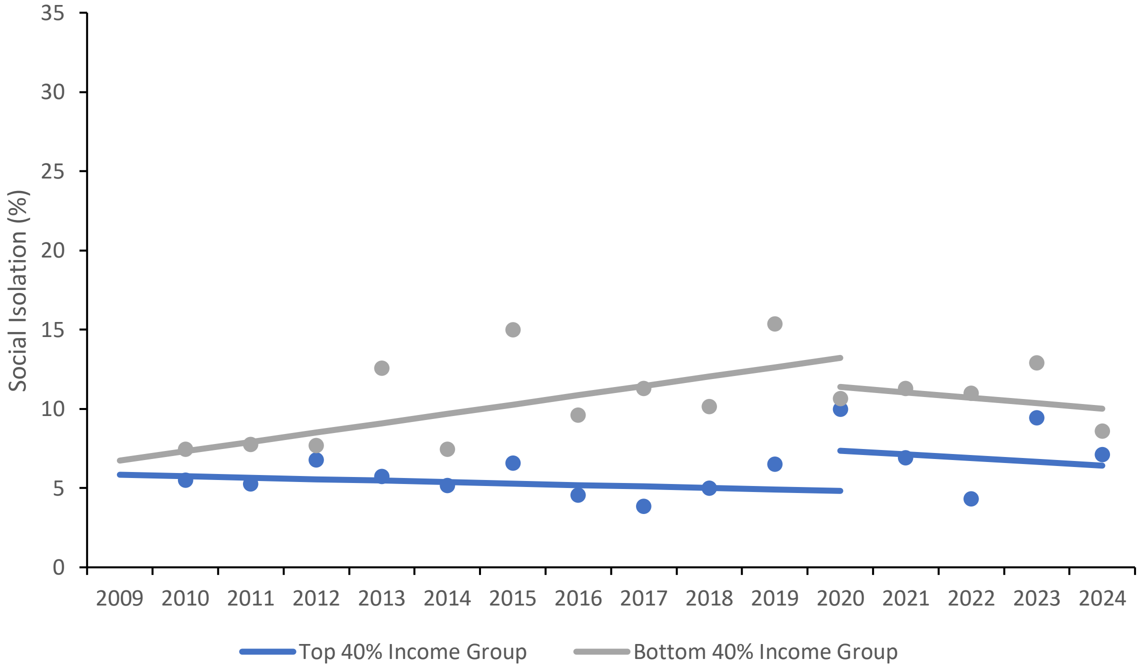

© 2025 Fuller-Rowell TE et al. *JAMA Network Open.*

eFigure 111. Trends in Social Isolation for Denmark by Income Group. Fitted trajectories are derived from empirical Bayes estimates of the final best fitting model, with raw data overlaid.

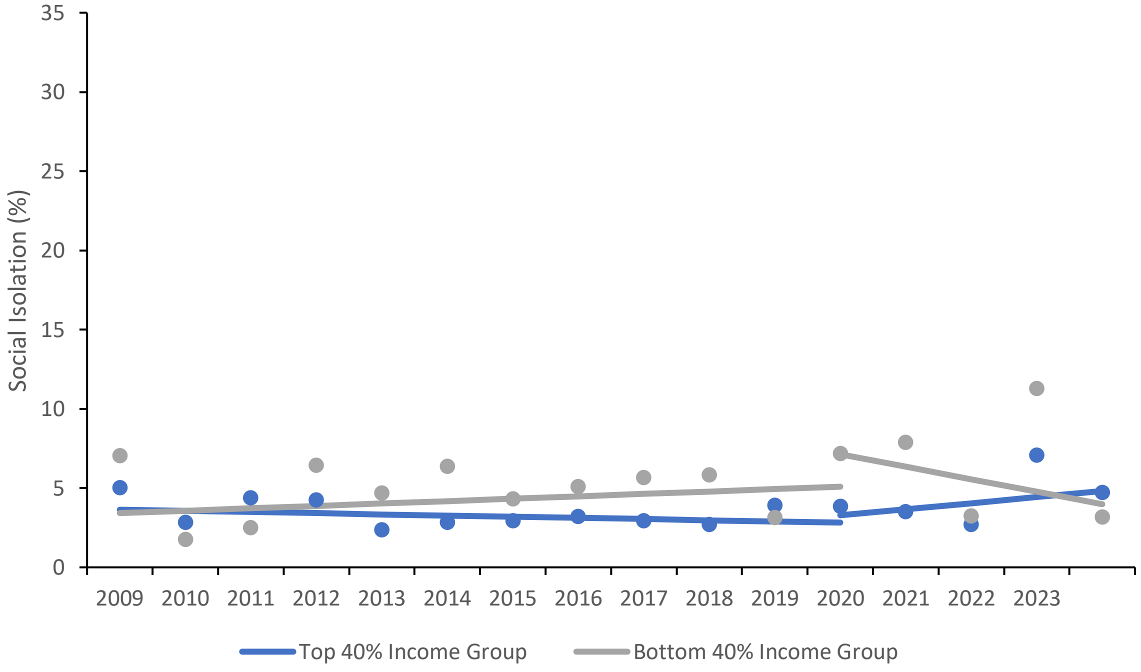

© 2025 Fuller-Rowell TE et al. *JAMA Network Open.*

eFigure 112. Trends in Social Isolation for the United Kingdom of Great Britain and Northern Ireland by Income Group. Fitted trajectories are derived from empirical Bayes estimates of the final best fitting model, with raw data overlaid.

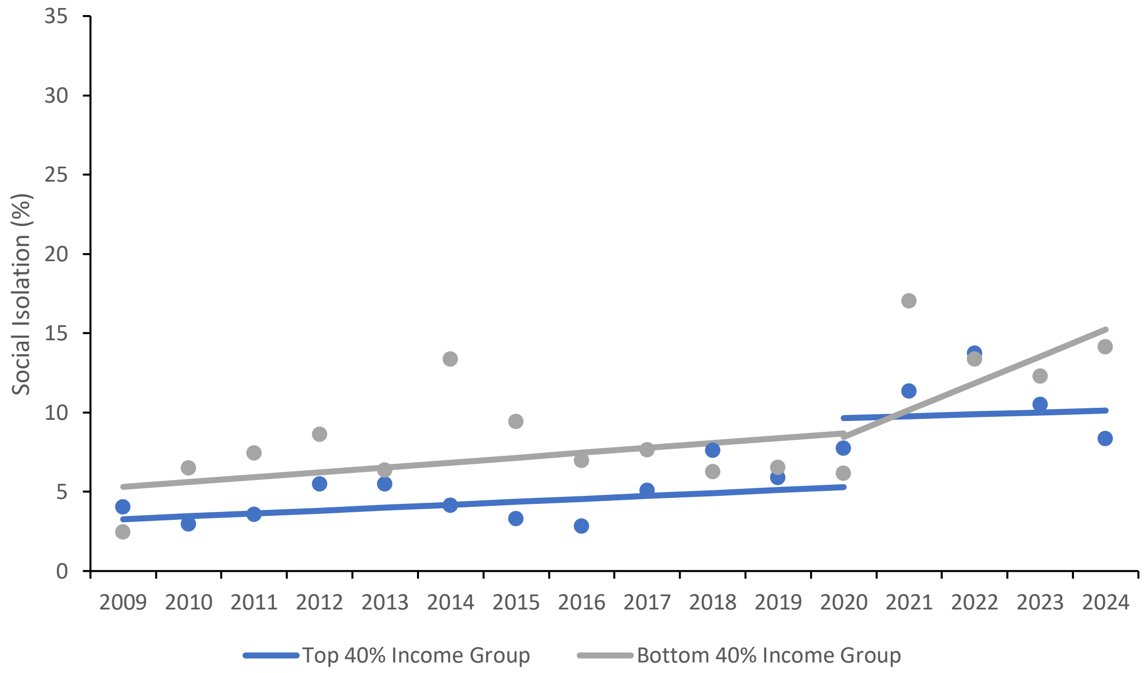

© 2025 Fuller-Rowell TE et al. *JAMA Network Open.*

eFigure 113. Trends in Social Isolation for Poland by Income Group. Fitted trajectories are derived from empirical Bayes estimates of the final best fitting model, with raw data overlaid.

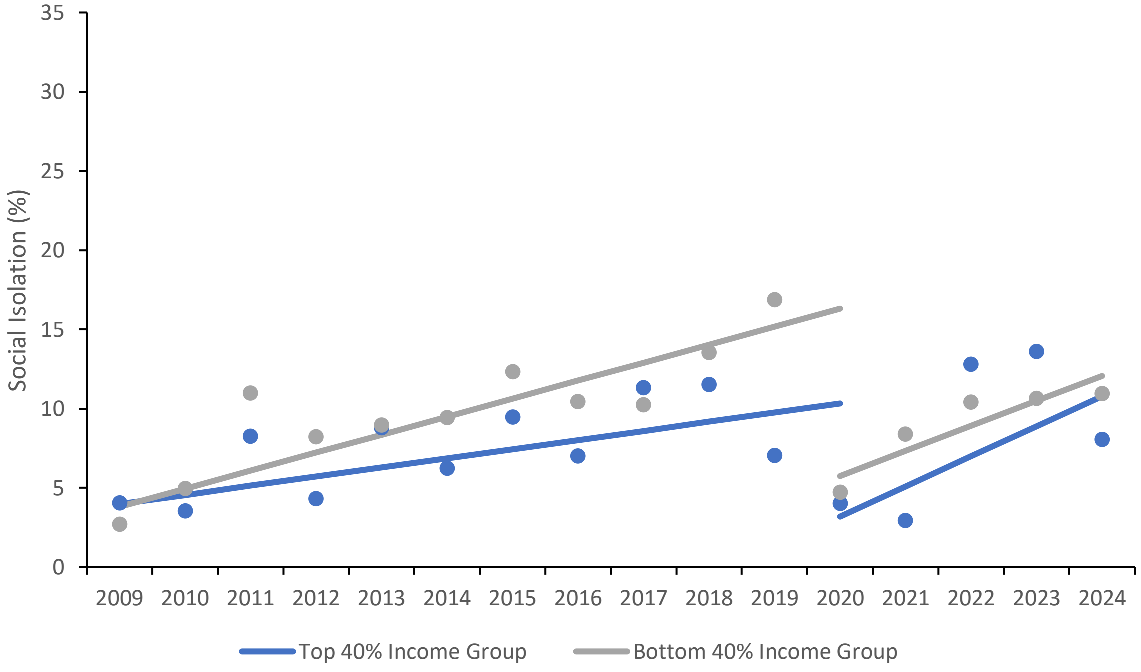

© 2025 Fuller-Rowell TE et al. *JAMA Network Open.*

eFigure 114. Trends in Social Isolation for Norway by Income Group. Fitted trajectories are derived from empirical Bayes estimates of the final best fitting model, with raw data overlaid.

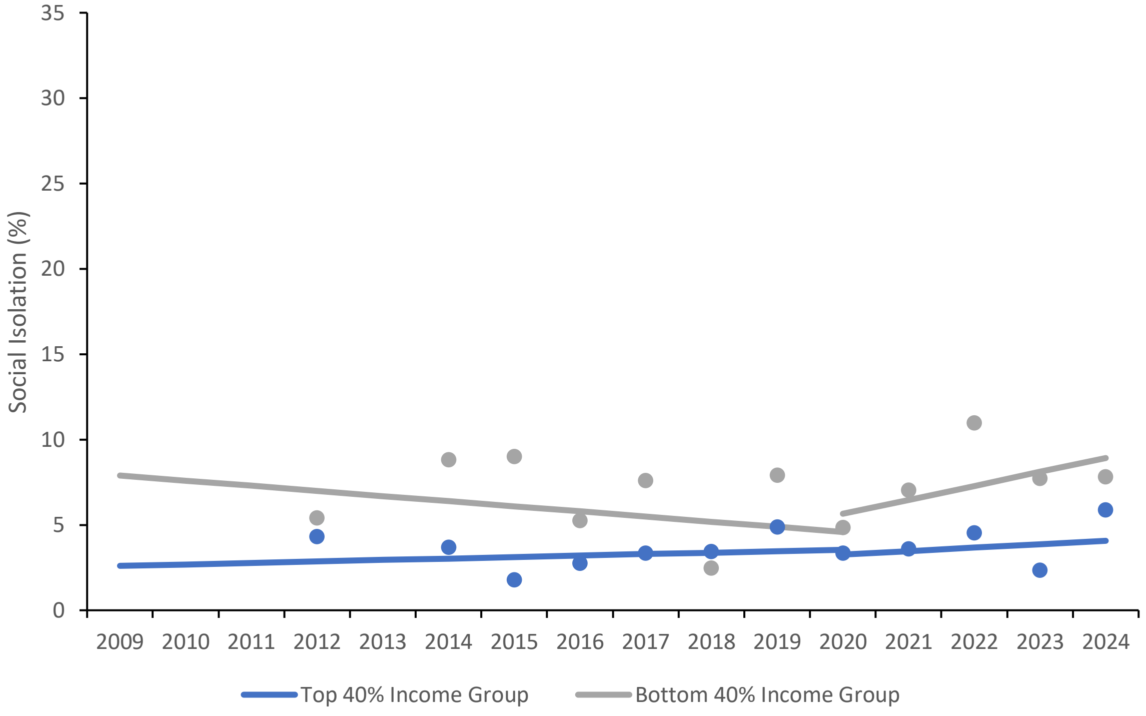

© 2025 Fuller-Rowell TE et al. *JAMA Network Open.*

eFigure 115. Trends in Social Isolation for Greece by Income Group. Fitted trajectories are derived from empirical Bayes estimates of the final best fitting model, with raw data overlaid.

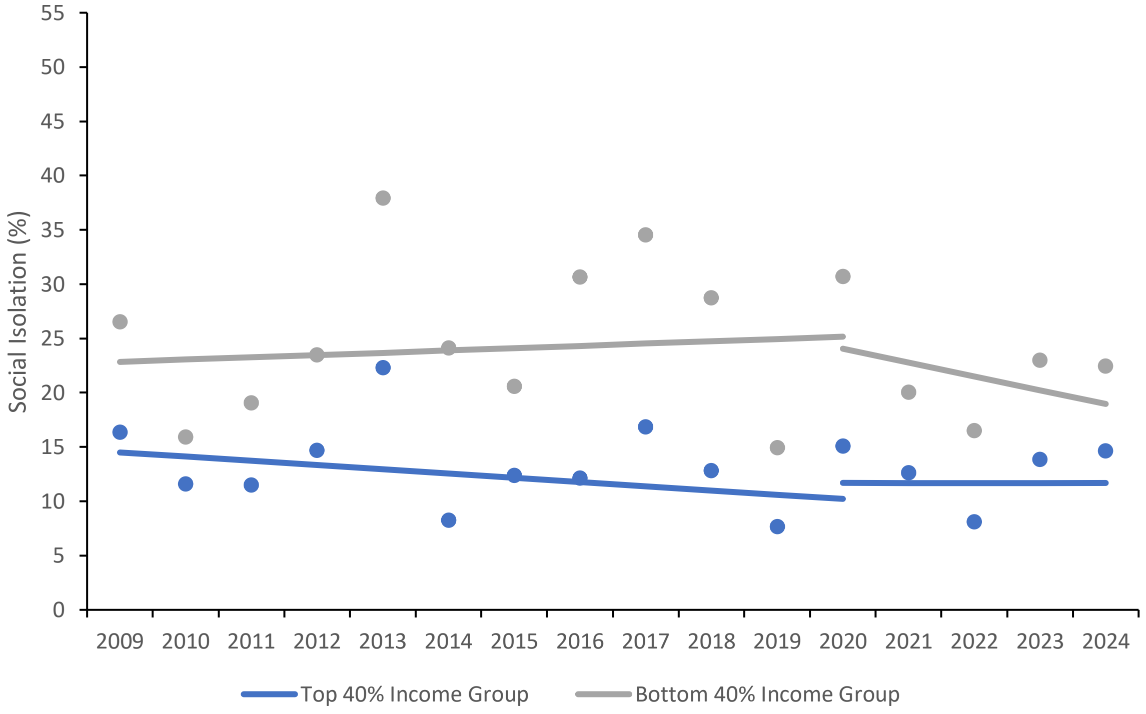

© 2025 Fuller-Rowell TE et al. *JAMA Network Open.*

eFigure 116. Trends in Social Isolation for Italy by Income Group. Fitted trajectories are derived from empirical Bayes estimates of the final best fitting model, with raw data overlaid.

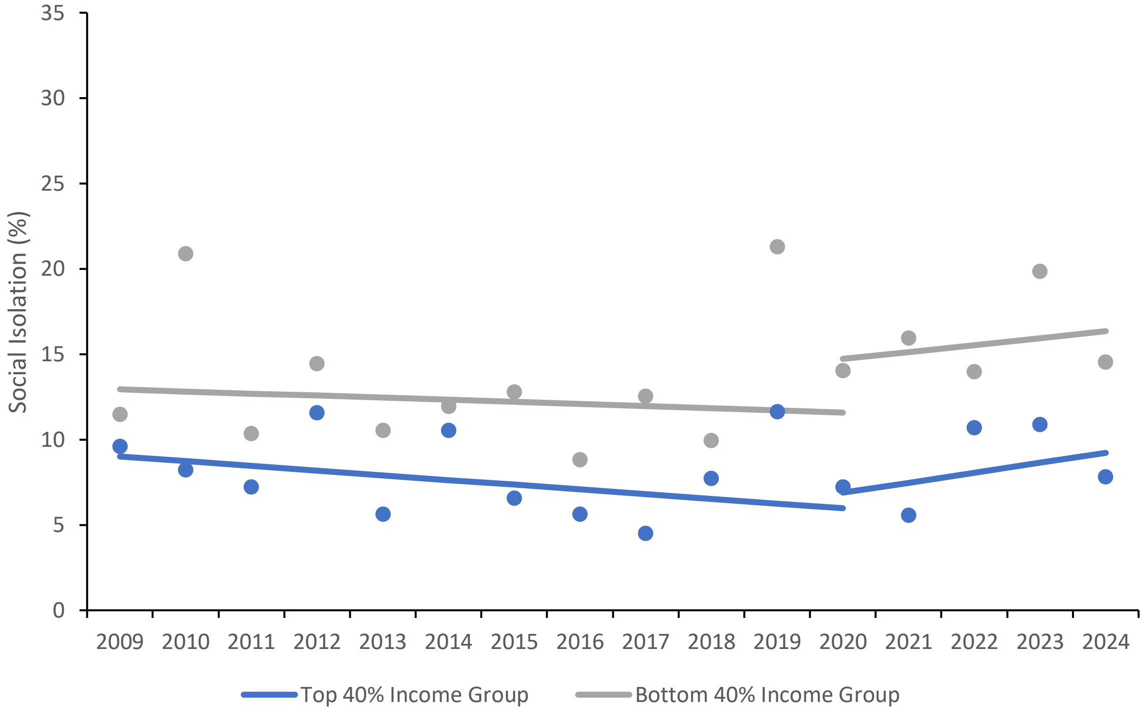

© 2025 Fuller-Rowell TE et al. *JAMA Network Open.*

eFigure 117. Trends in Social Isolation for the Netherlands by Income Group. Fitted trajectories are derived from empirical Bayes estimates of the final best fitting model, with raw data overlaid.

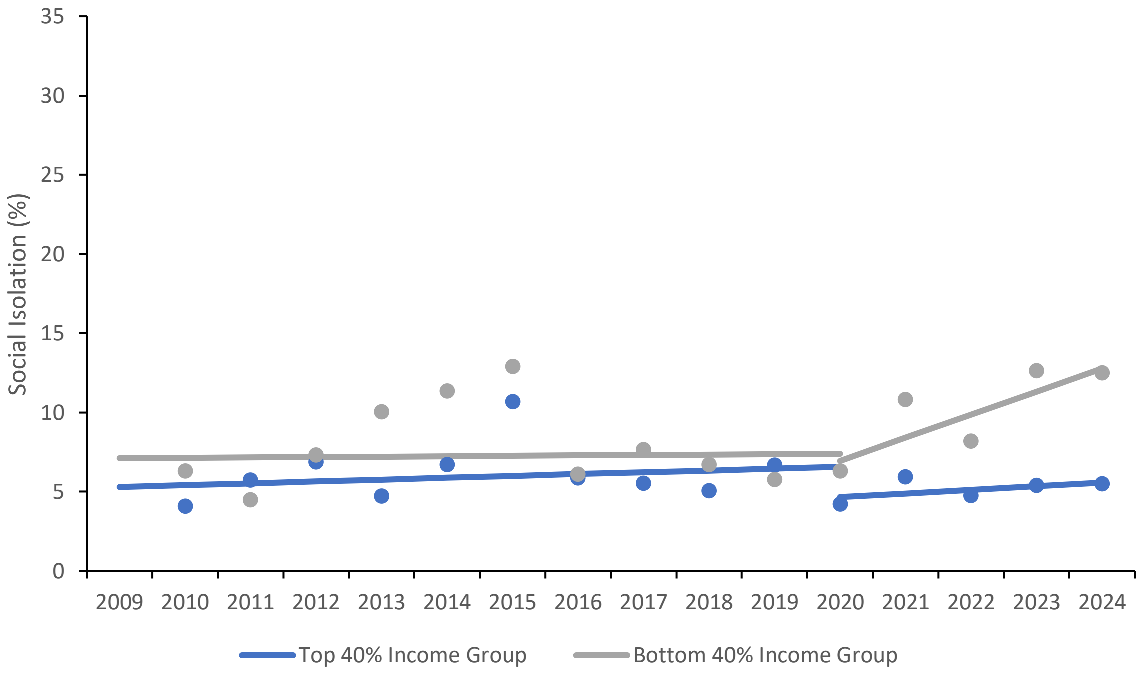

© 2025 Fuller-Rowell TE et al. *JAMA Network Open.*

eFigure 118. Trends in Social Isolation for Sweden by Income Group. Fitted trajectories are derived from empirical Bayes estimates of the final best fitting model, with raw data overlaid.

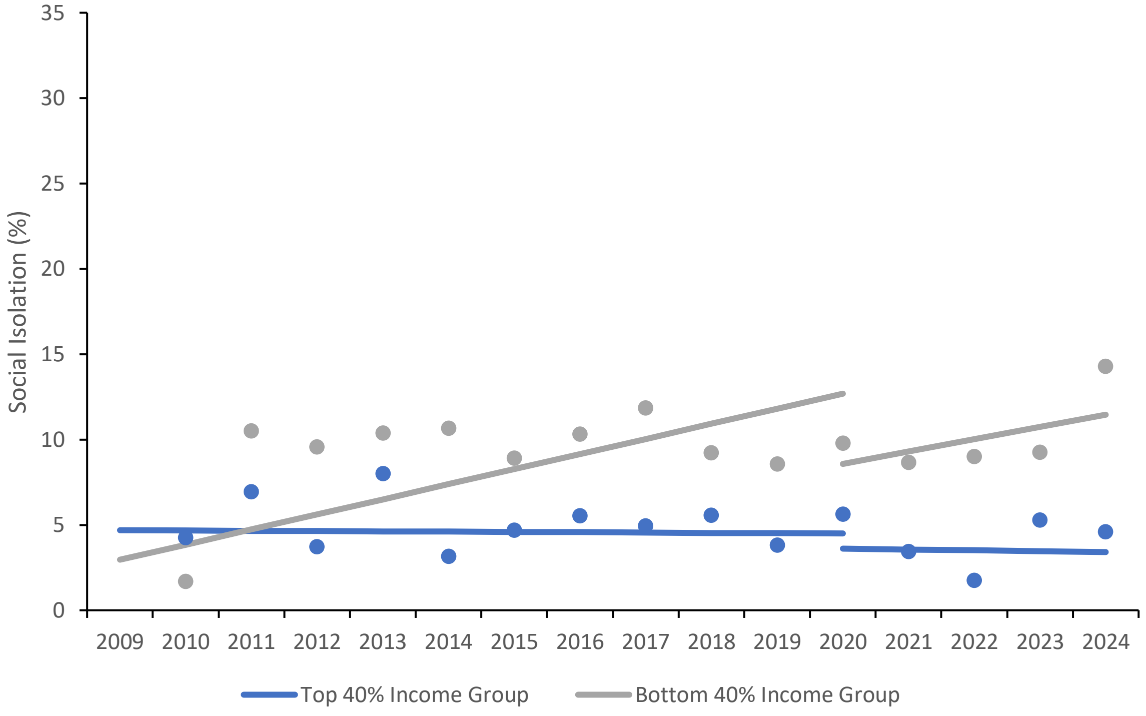

© 2025 Fuller-Rowell TE et al. *JAMA Network Open.*

eFigure 119. Trends in Social Isolation for Malta by Income Group. Fitted trajectories are derived from empirical Bayes estimates of the final best fitting model, with raw data overlaid.

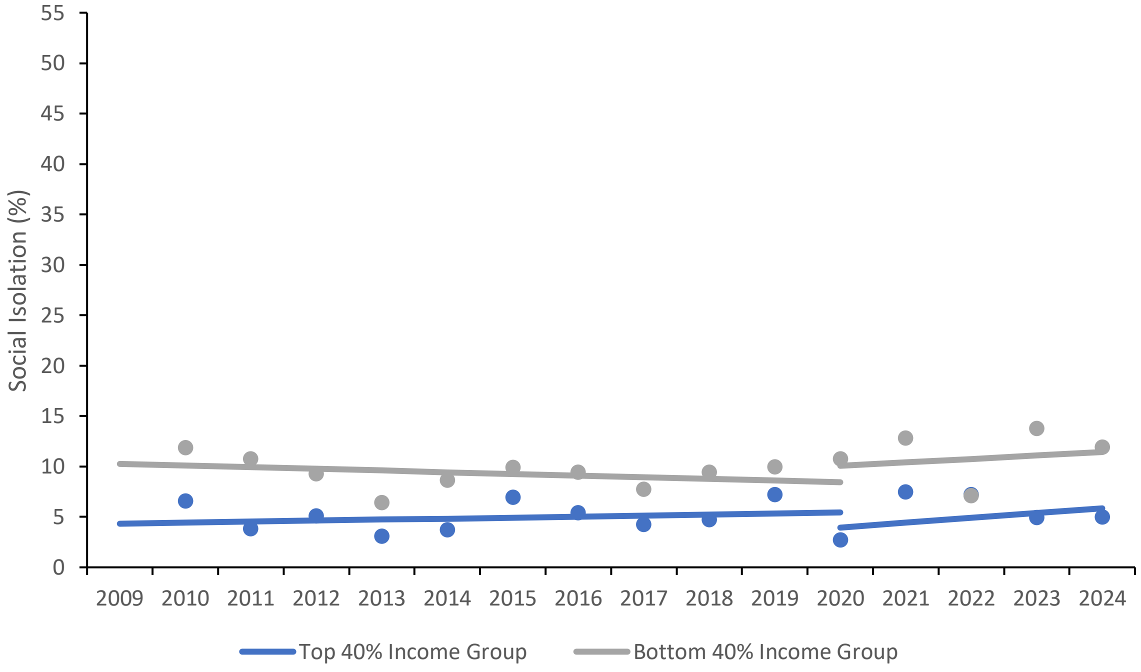

© 2025 Fuller-Rowell TE et al. *JAMA Network Open.*

eFigure 120. Trends in Social Isolation for the Republic of Cyprus by Income Group. Fitted trajectories are derived from empirical Bayes estimates of the final best fitting model, with raw data overlaid.

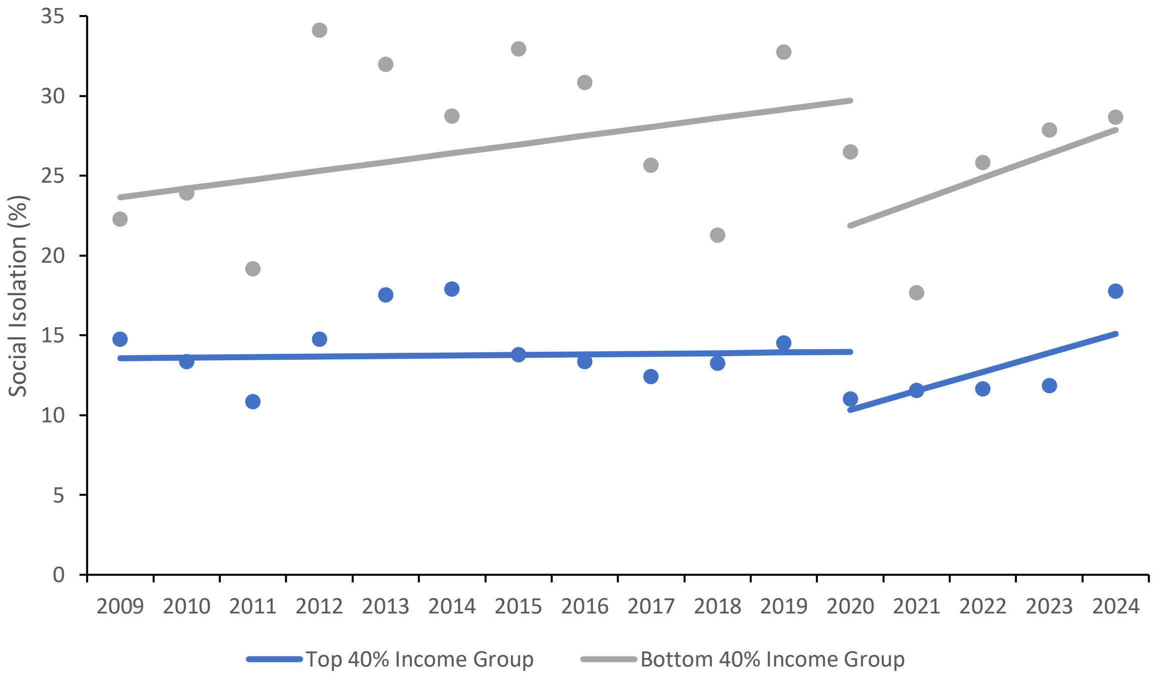

© 2025 Fuller-Rowell TE et al. *JAMA Network Open.*

eFigure 121. Trends in Social Isolation for Spain by Income Group. Fitted trajectories are derived from empirical Bayes estimates of the final best fitting model, with raw data overlaid.

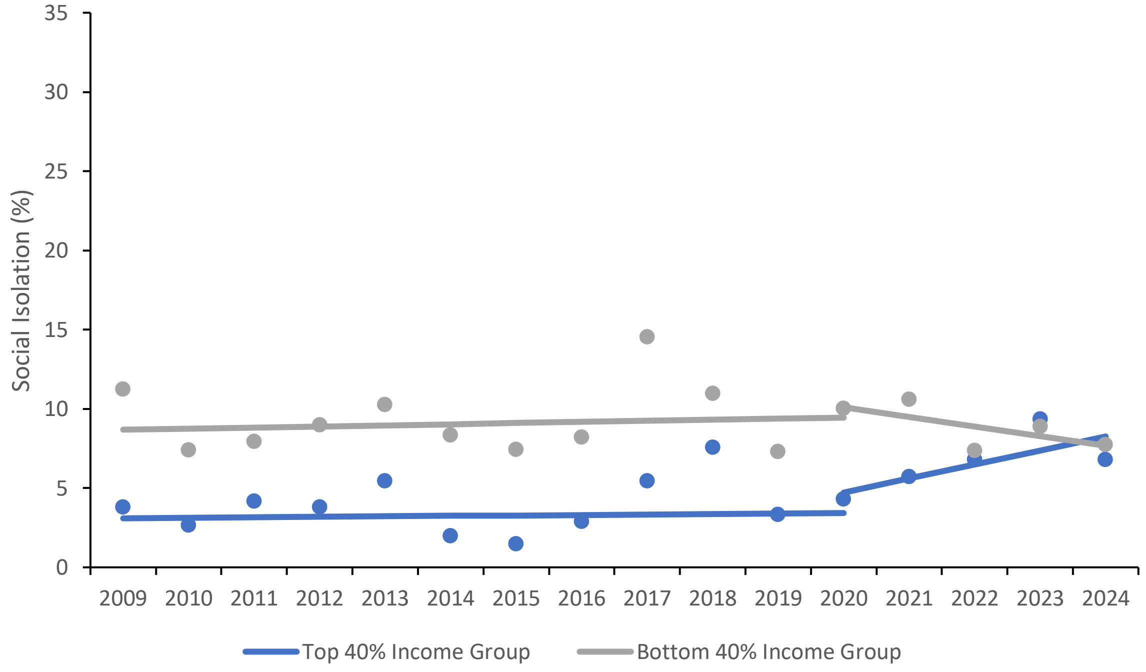

© 2025 Fuller-Rowell TE et al. *JAMA Network Open.*

eFigure 122. Trends in Social Isolation for Iceland by Income Group. Fitted trajectories are derived from empirical Bayes estimates of the final best fitting model, with raw data overlaid.

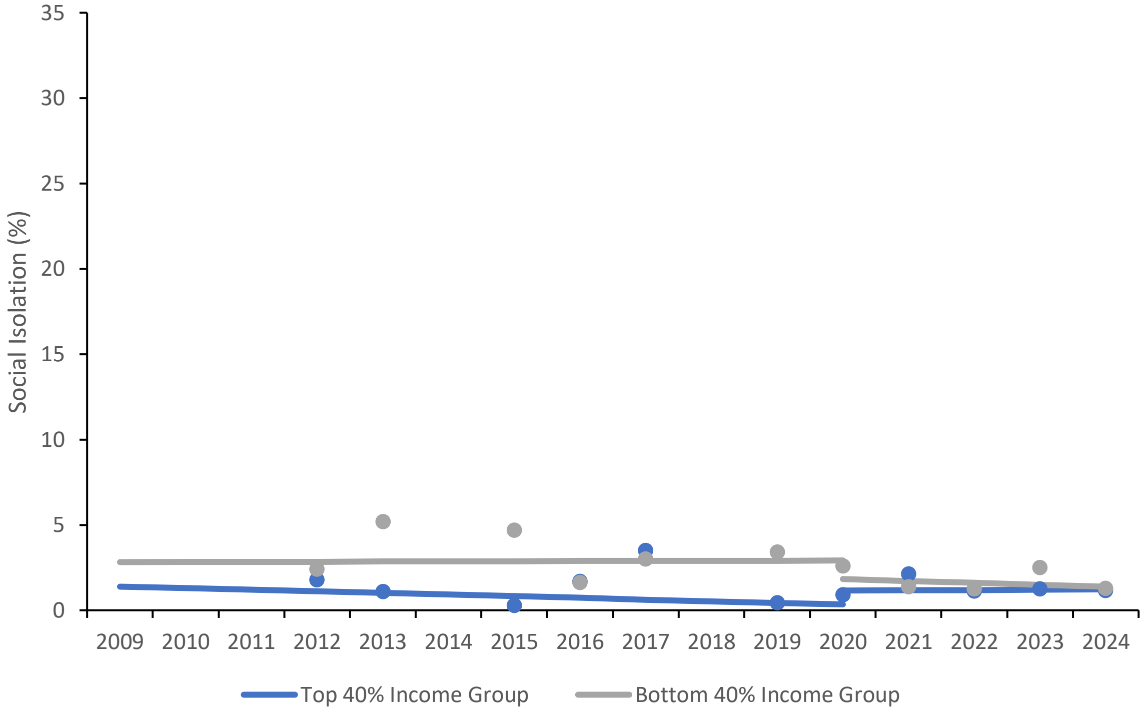

© 2025 Fuller-Rowell TE et al. *JAMA Network Open.*

eFigure 123. Trends in Social Isolation for Switzerland by Income Group. Fitted trajectories are derived from empirical Bayes estimates of the final best fitting model, with raw data overlaid.

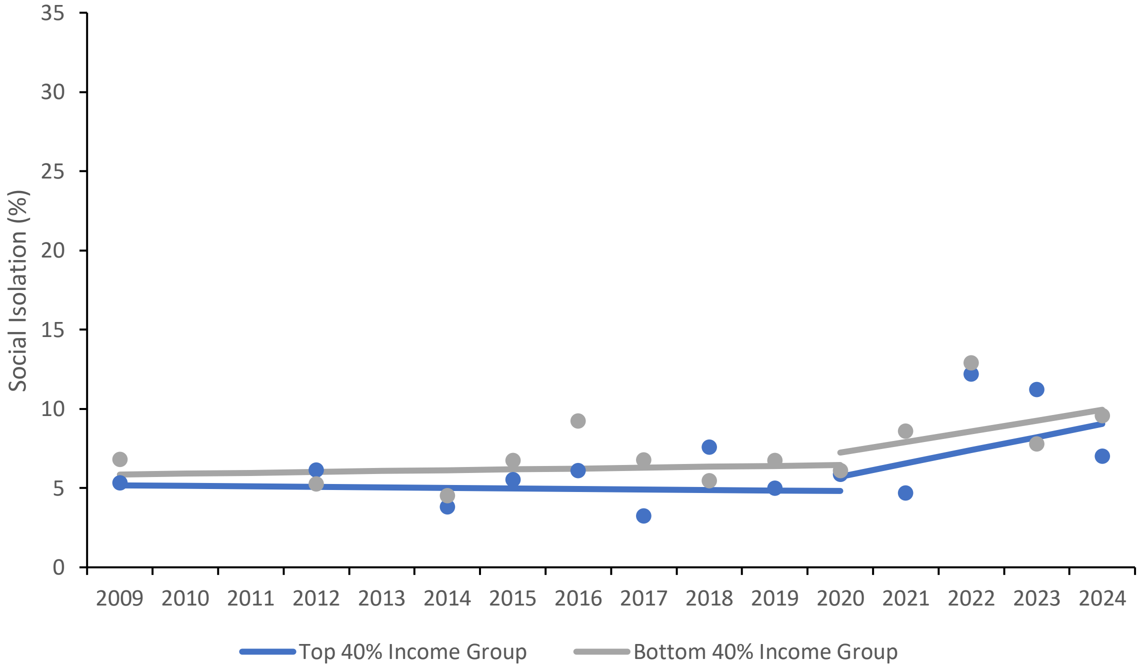

© 2025 Fuller-Rowell TE et al. *JAMA Network Open.*

eFigure 124. Trends in Social Isolation for Portugal by Income Group. Fitted trajectories are derived from empirical Bayes estimates of the final best fitting model, with raw data overlaid.

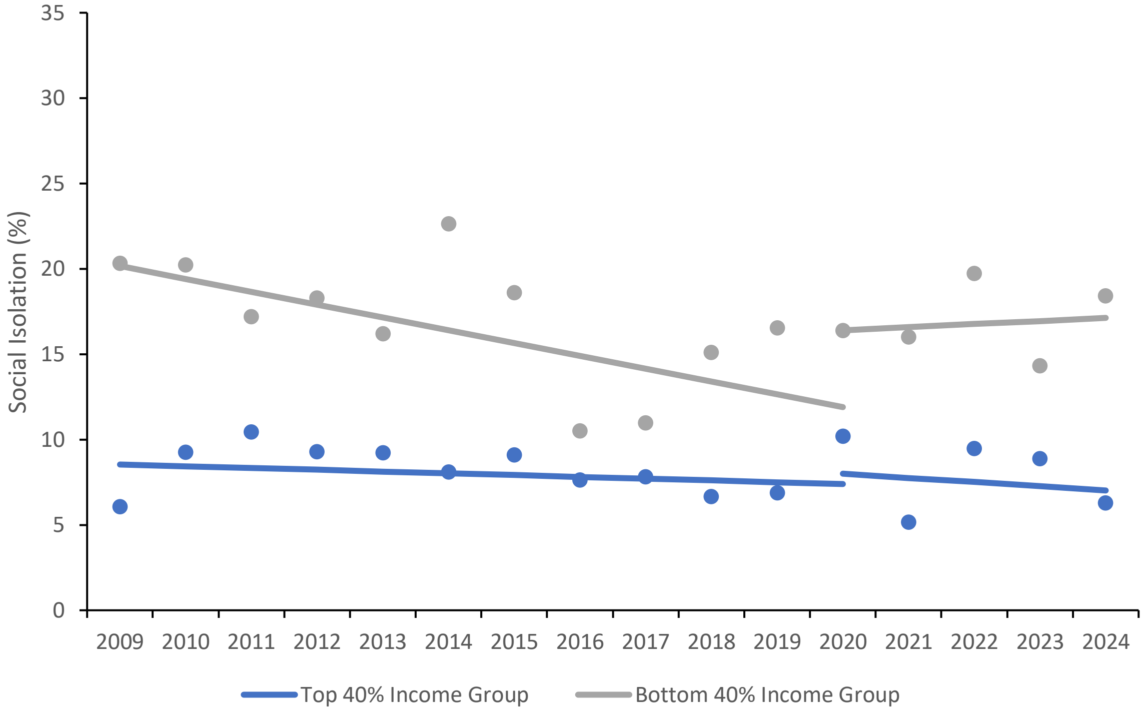

© 2025 Fuller-Rowell TE et al. *JAMA Network Open.*

eFigure 125. Trends in Social Isolation for the Czech Republic by Income Group. Fitted trajectories are derived from empirical Bayes estimates of the final best fitting model, with raw data overlaid.

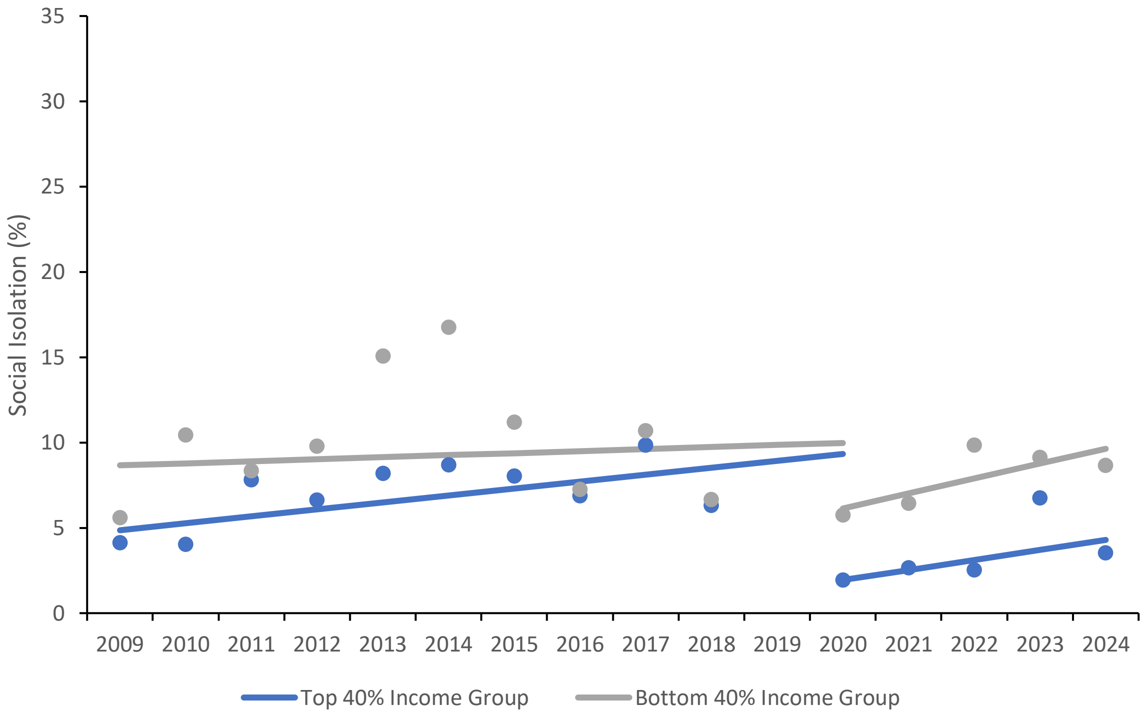

© 2025 Fuller-Rowell TE et al. *JAMA Network Open.*

eFigure 126. Trends in Social Isolation for Slovenia by Income Group. Fitted trajectories are derived from empirical Bayes estimates of the final best fitting model, with raw data overlaid.

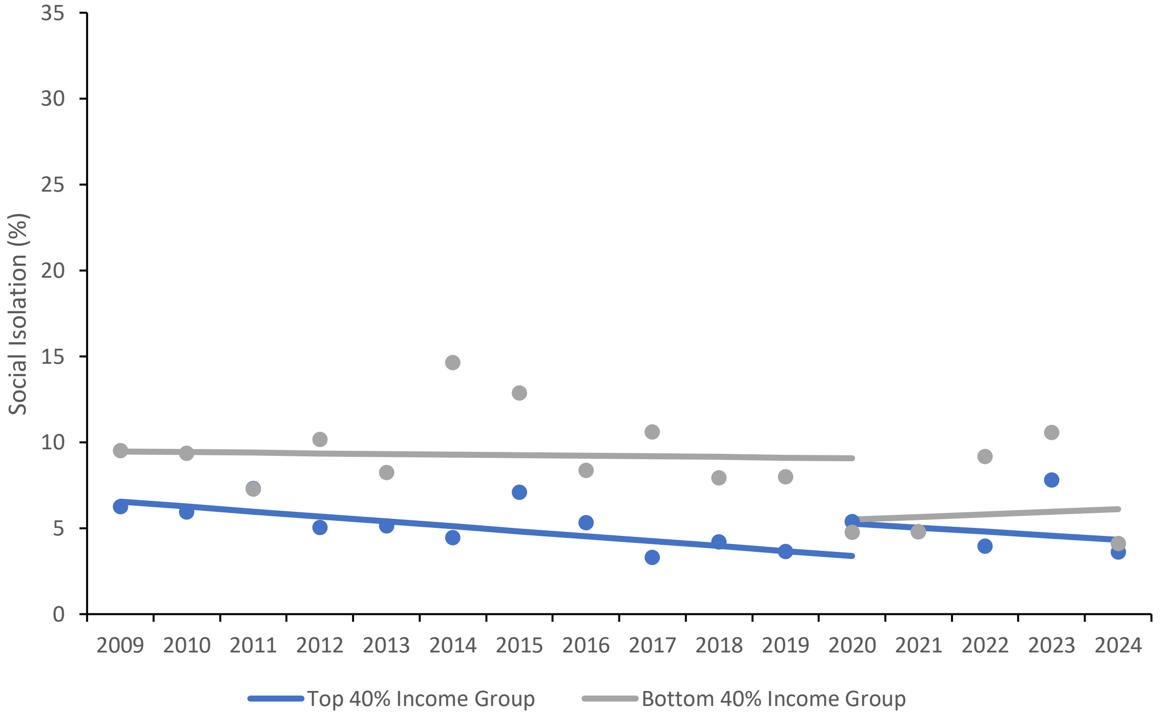

© 2025 Fuller-Rowell TE et al. *JAMA Network Open.*

eFigure 127. Trends in Social Isolation for Estonia by Income Group. Fitted trajectories are derived from empirical Bayes estimates of the final best fitting model, with raw data overlaid.

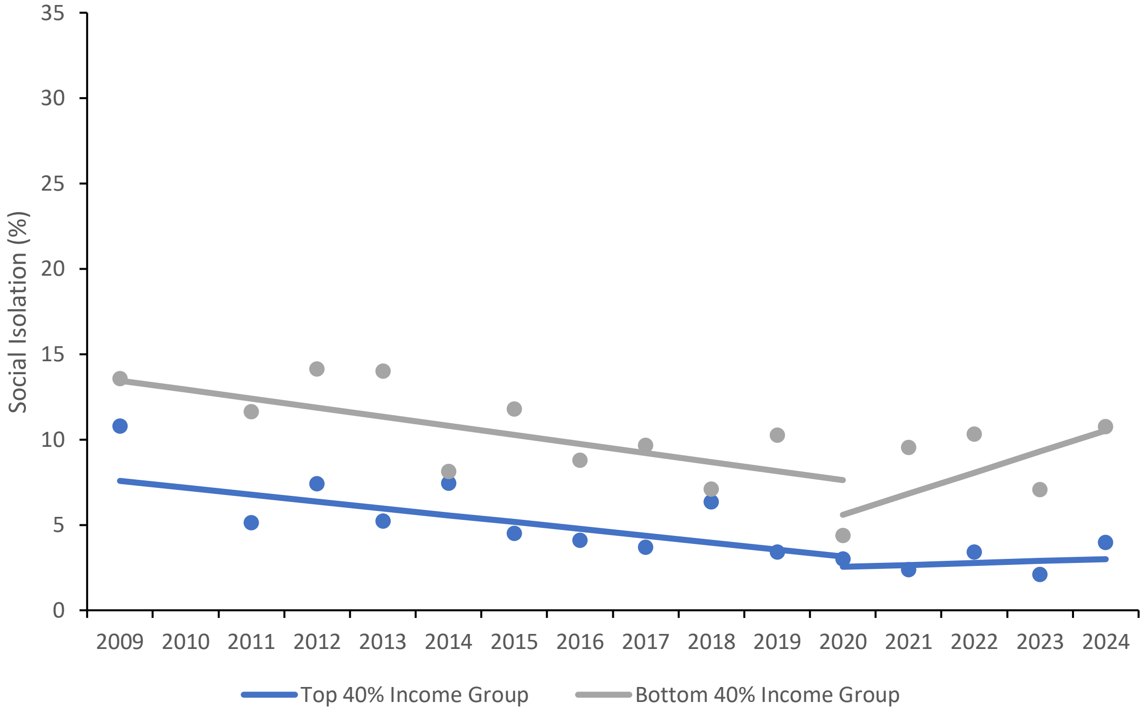

© 2025 Fuller-Rowell TE et al. *JAMA Network Open.*

eFigure 128. Trends in Social Isolation for France by Income Group. Fitted trajectories are derived from empirical Bayes estimates of the final best fitting model, with raw data overlaid.

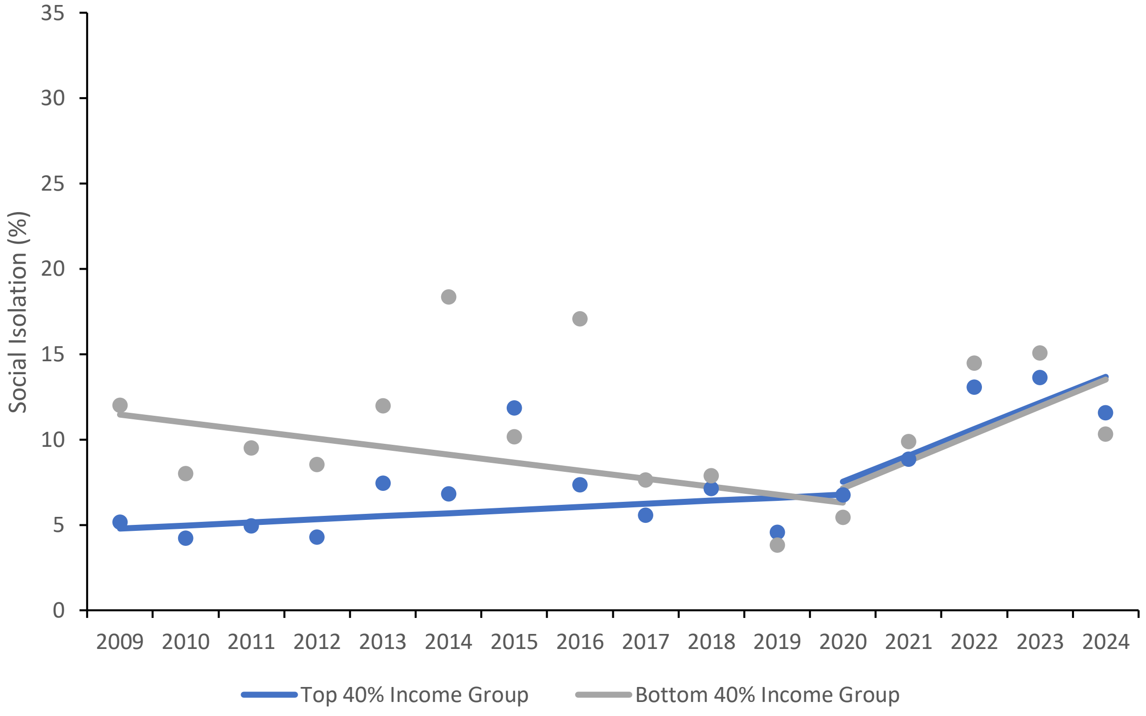

© 2025 Fuller-Rowell TE et al. *JAMA Network Open.*

eFigure 129. Trends in Social Isolation for Lithuania by Income Group. Fitted trajectories are derived from empirical Bayes estimates of the final best fitting model, with raw data overlaid.

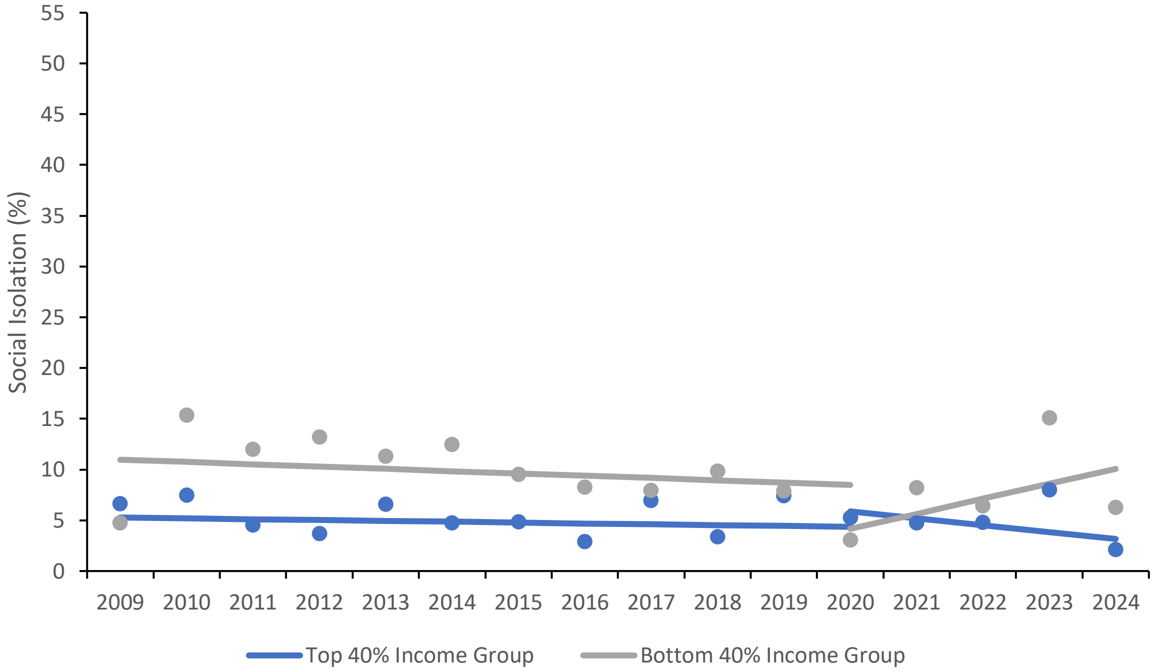

© 2025 Fuller-Rowell TE et al. *JAMA Network Open.*

eFigure 130. Trends in Social Isolation for North Macedonia by Income Group. Fitted trajectories are derived from empirical Bayes estimates of the final best fitting model, with raw data overlaid.

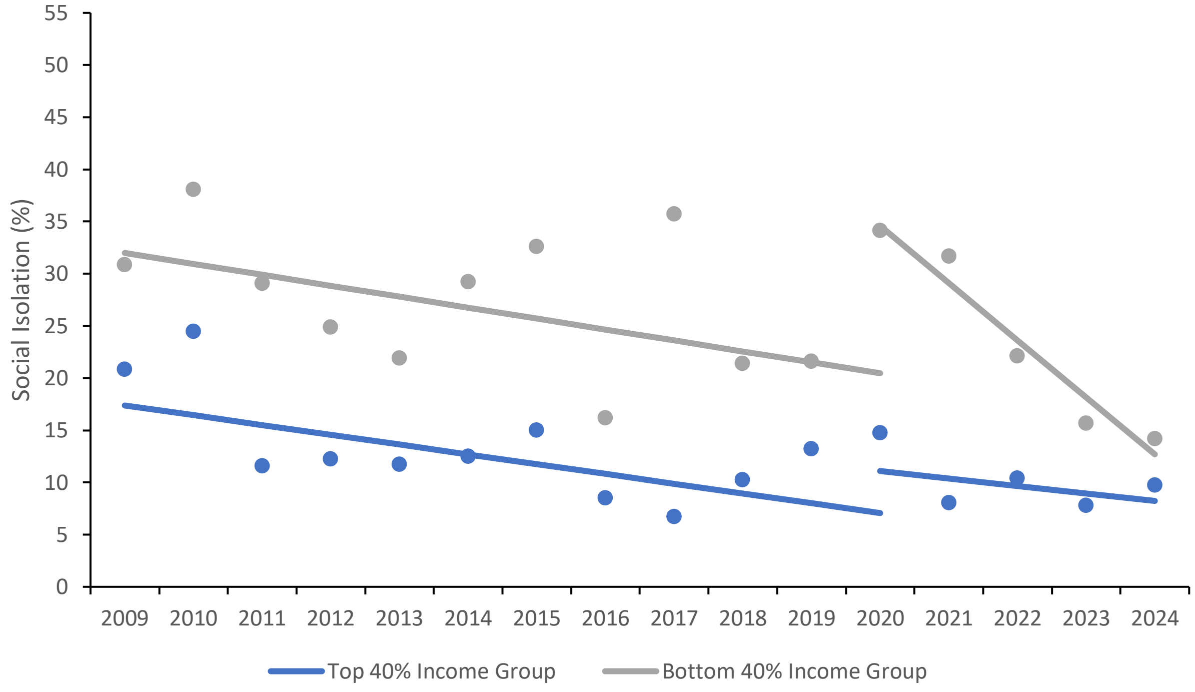

© 2025 Fuller-Rowell TE et al. *JAMA Network Open.*

eFigure 131. Trends in Social Isolation for Montenegro by Income Group. Fitted trajectories are derived from empirical Bayes estimates of the final best fitting model, with raw data overlaid.

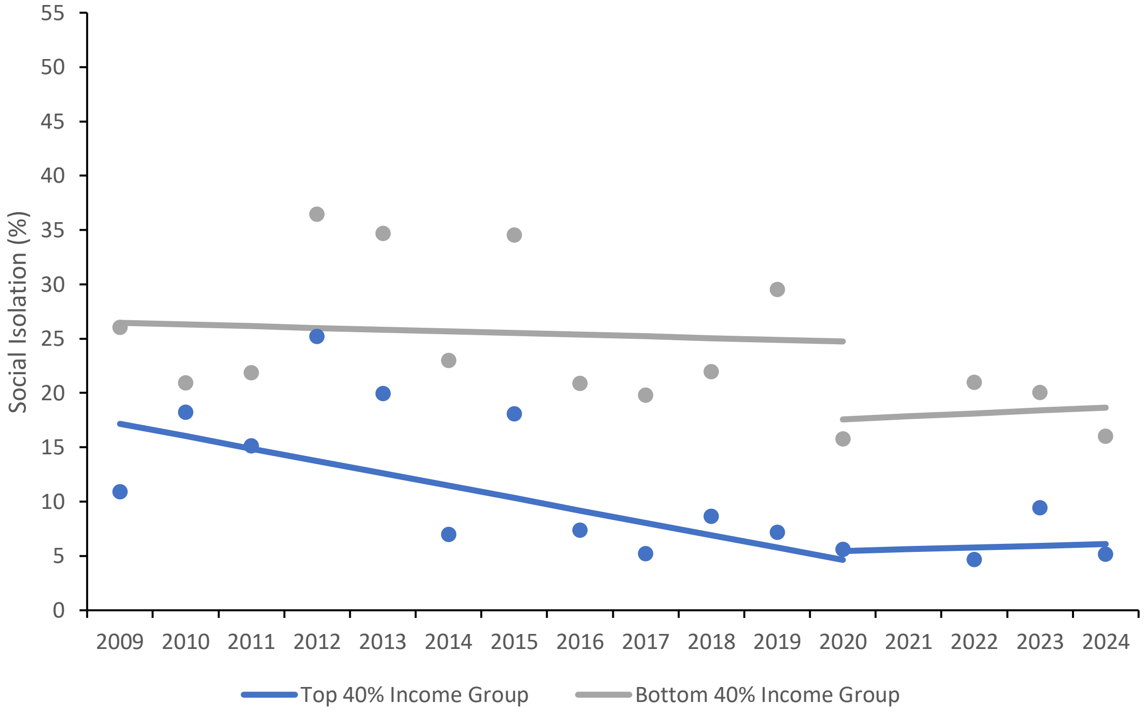

© 2025 Fuller-Rowell TE et al. *JAMA Network Open.*

eFigure 132. Trends in Social Isolation for Finland by Income Group. Fitted trajectories are derived from empirical Bayes estimates of the final best fitting model, with raw data overlaid.

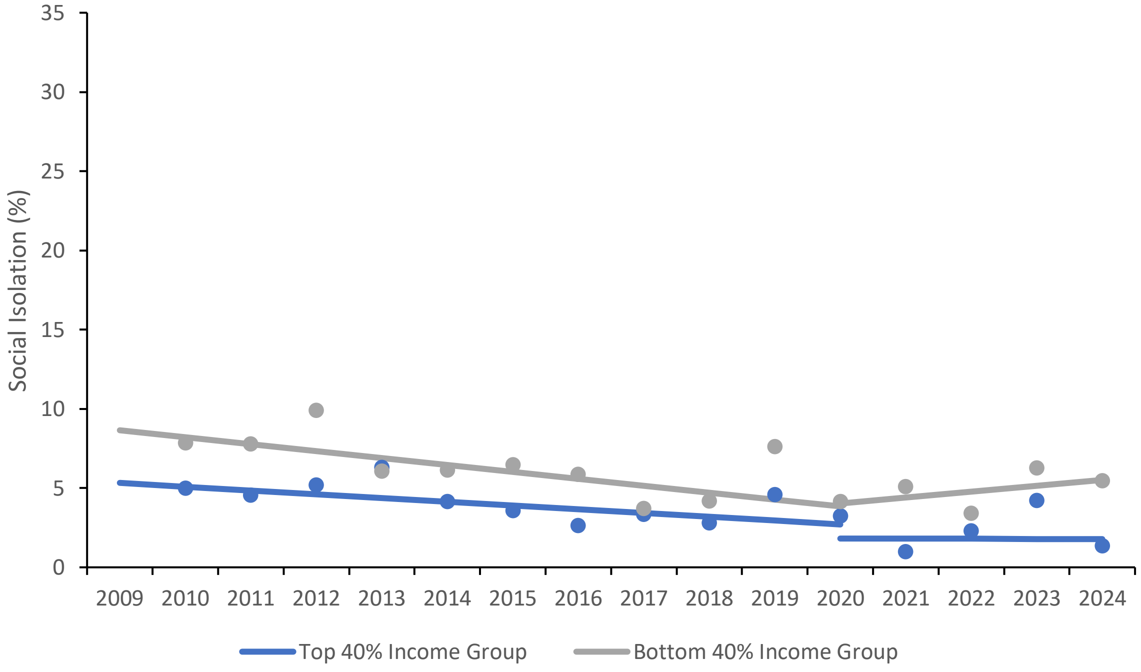

© 2025 Fuller-Rowell TE et al. *JAMA Network Open.*

eFigure 133. Trends in Social Isolation for Slovakia by Income Group. Fitted trajectories are derived from empirical Bayes estimates of the final best fitting model, with raw data overlaid.

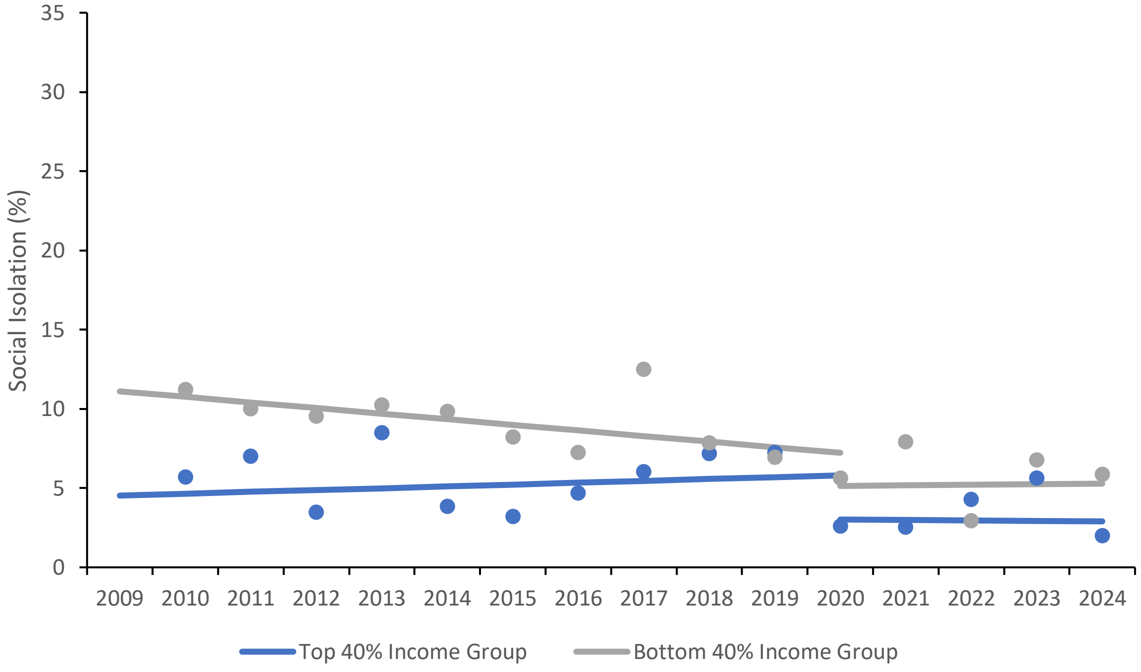

© 2025 Fuller-Rowell TE et al. *JAMA Network Open.*

eFigure 134. Trends in Social Isolation for Croatia by Income Group. Fitted trajectories are derived from empirical Bayes estimates of the final best fitting model, with raw data overlaid.

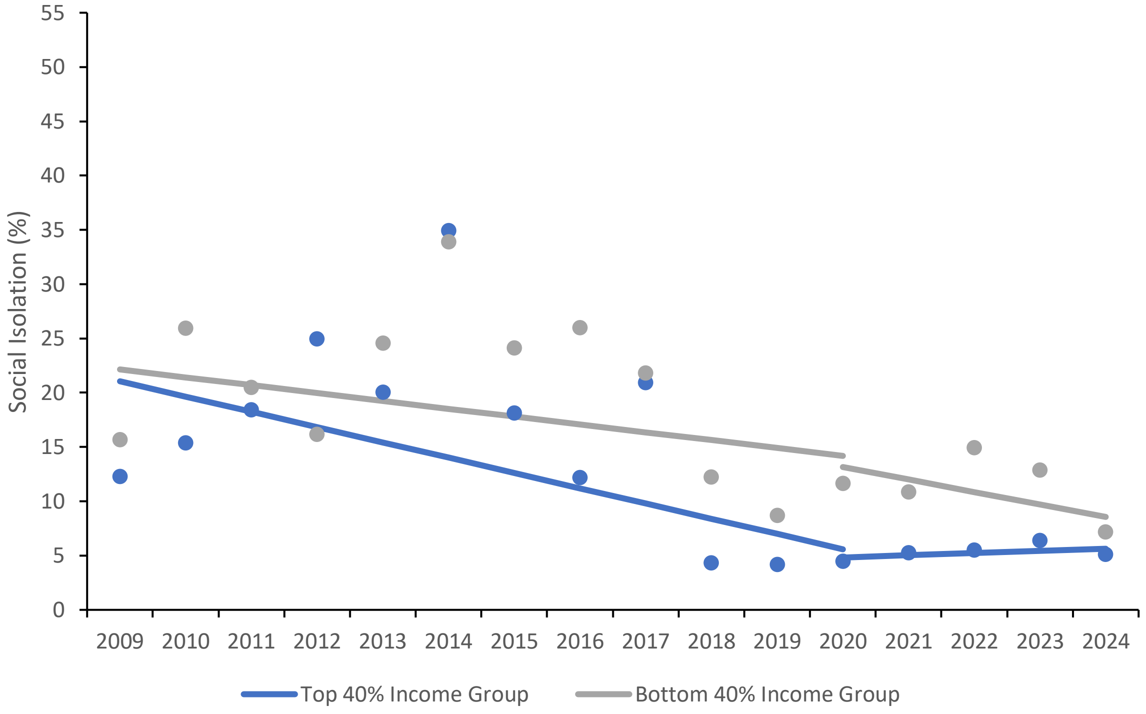

© 2025 Fuller-Rowell TE et al. *JAMA Network Open.*

eFigure 135. Trends in Social Isolation for Serbia by Income Group. Fitted trajectories are derived from empirical Bayes estimates of the final best fitting model, with raw data overlaid.

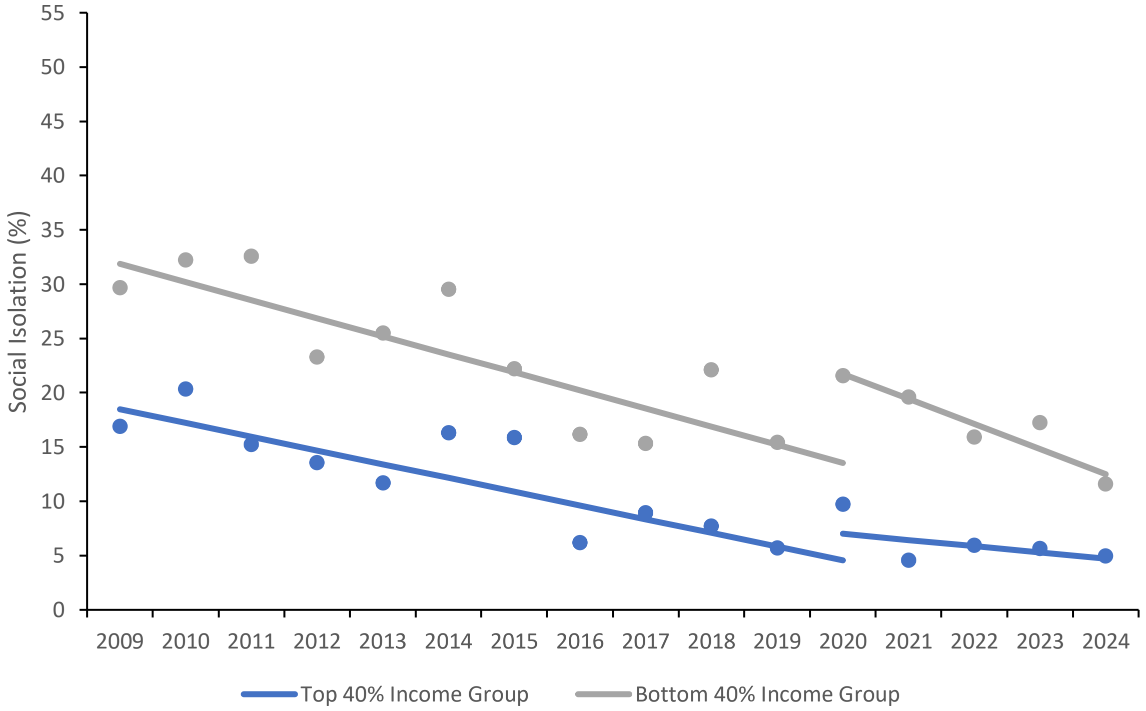

© 2025 Fuller-Rowell TE et al. *JAMA Network Open.*

eFigure 136. Trends in Social Isolation for Latvia by Income Group. Fitted trajectories are derived from empirical Bayes estimates of the final best fitting model, with raw data overlaid.

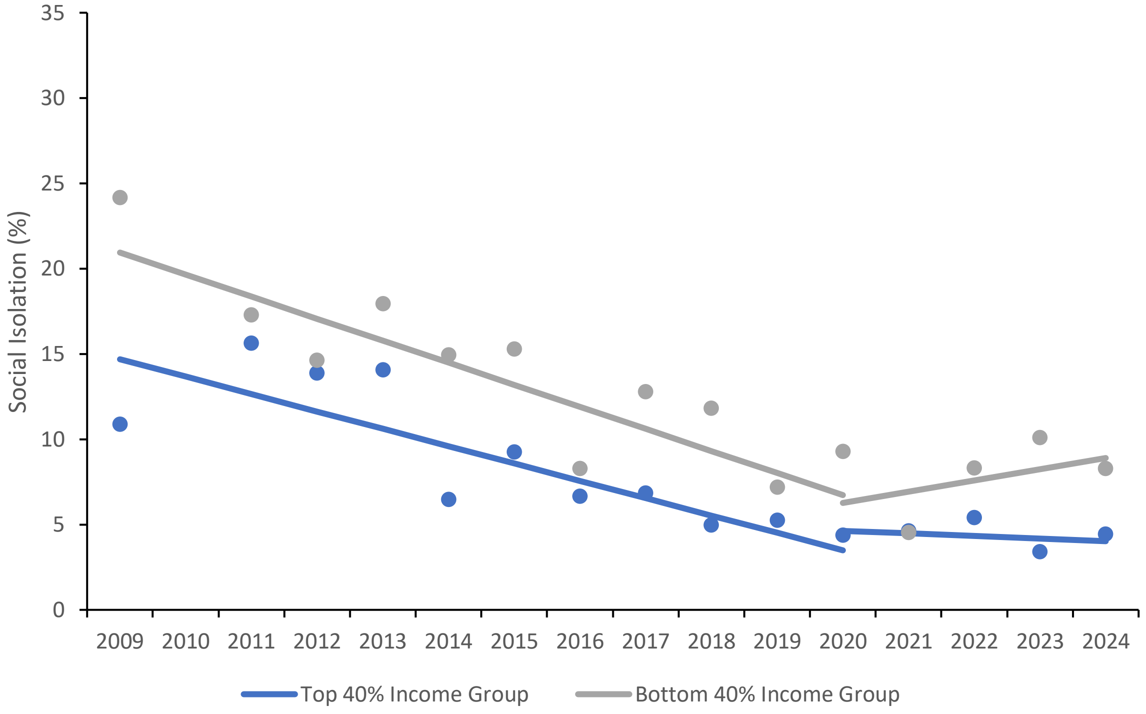

© 2025 Fuller-Rowell TE et al. *JAMA Network Open.*

eFigure 137. Trends in Social Isolation for Romania by Income Group. Fitted trajectories are derived from empirical Bayes estimates of the final best fitting model, with raw data overlaid.

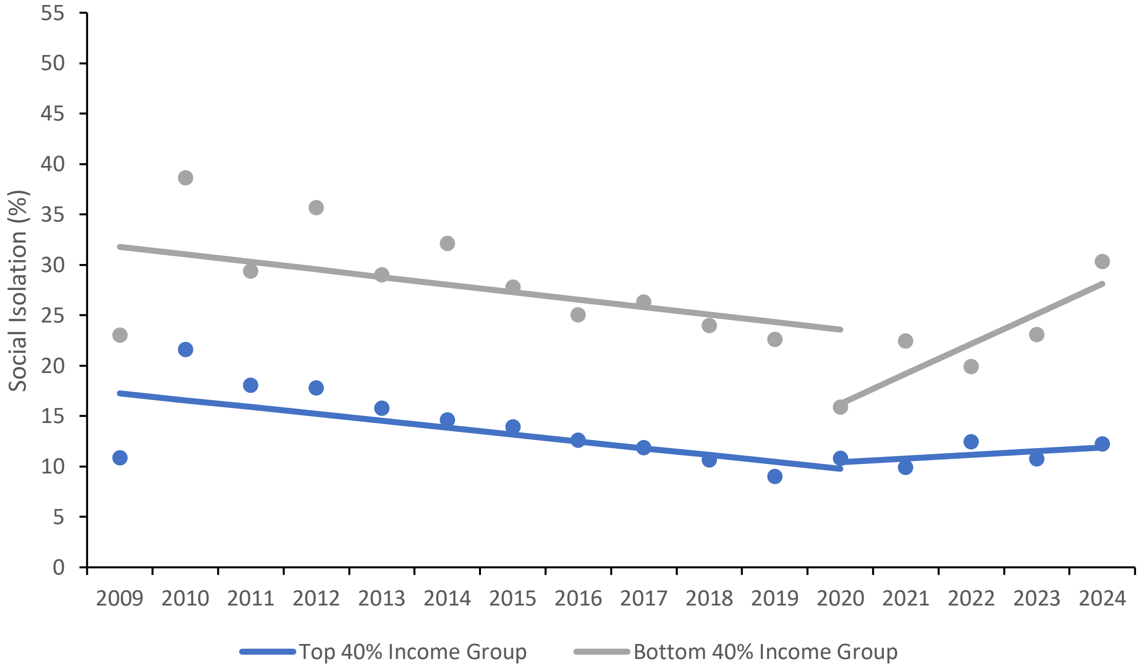

© 2025 Fuller-Rowell TE et al. *JAMA Network Open.*

eFigure 138. Trends in Social Isolation for Hungary by Income Group. Fitted trajectories are derived from empirical Bayes estimates of the final best fitting model, with raw data overlaid.

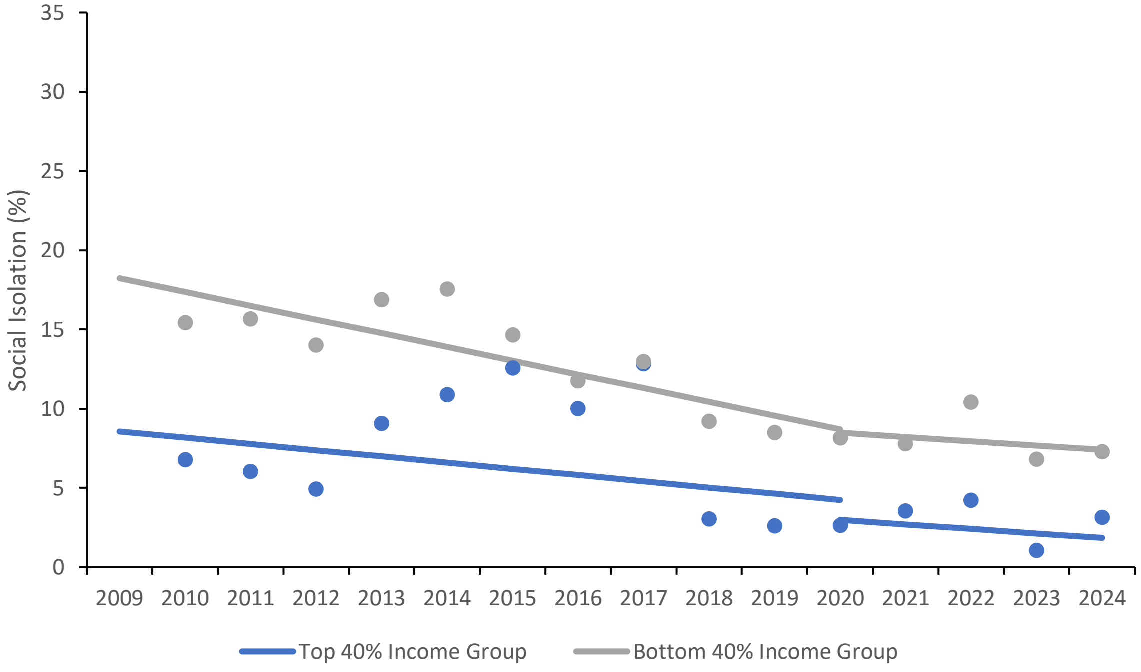

© 2025 Fuller-Rowell TE et al. *JAMA Network Open.*

eFigure 139. Trends in Social Isolation for Bulgaria by Income Group. Fitted trajectories are derived from empirical Bayes estimates of the final best fitting model, with raw data overlaid.

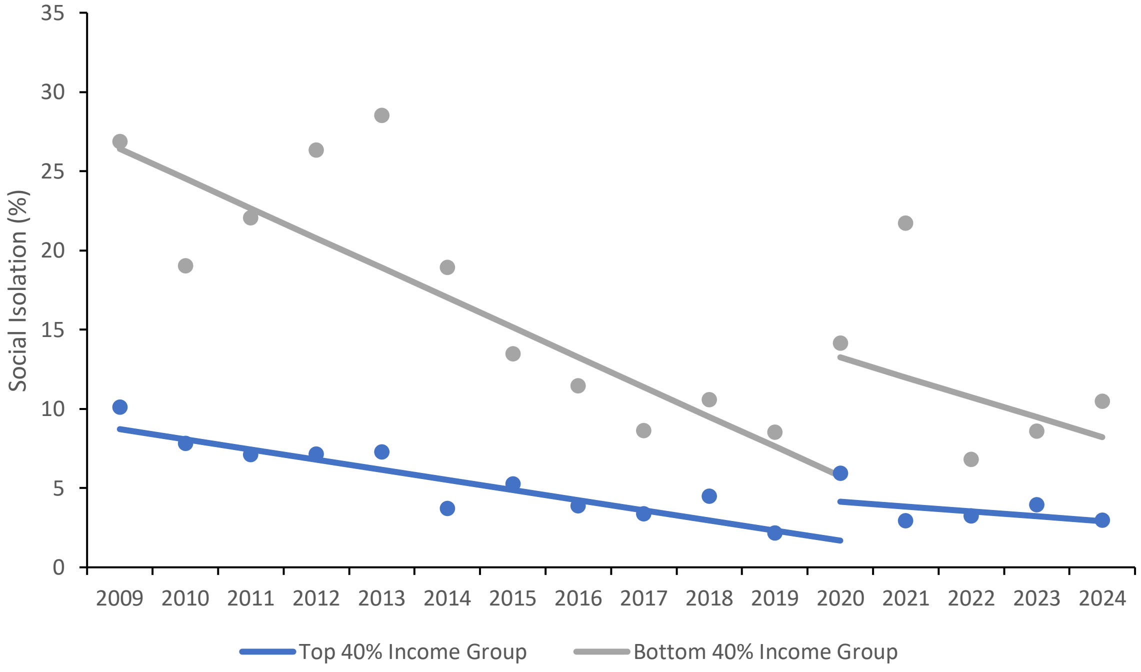

© 2025 Fuller-Rowell TE et al. *JAMA Network Open.*

eFigure 140. Trends in Social Isolation for Kosovo by Income Group. Fitted trajectories are derived from empirical Bayes estimates of the final best fitting model, with raw data overlaid.

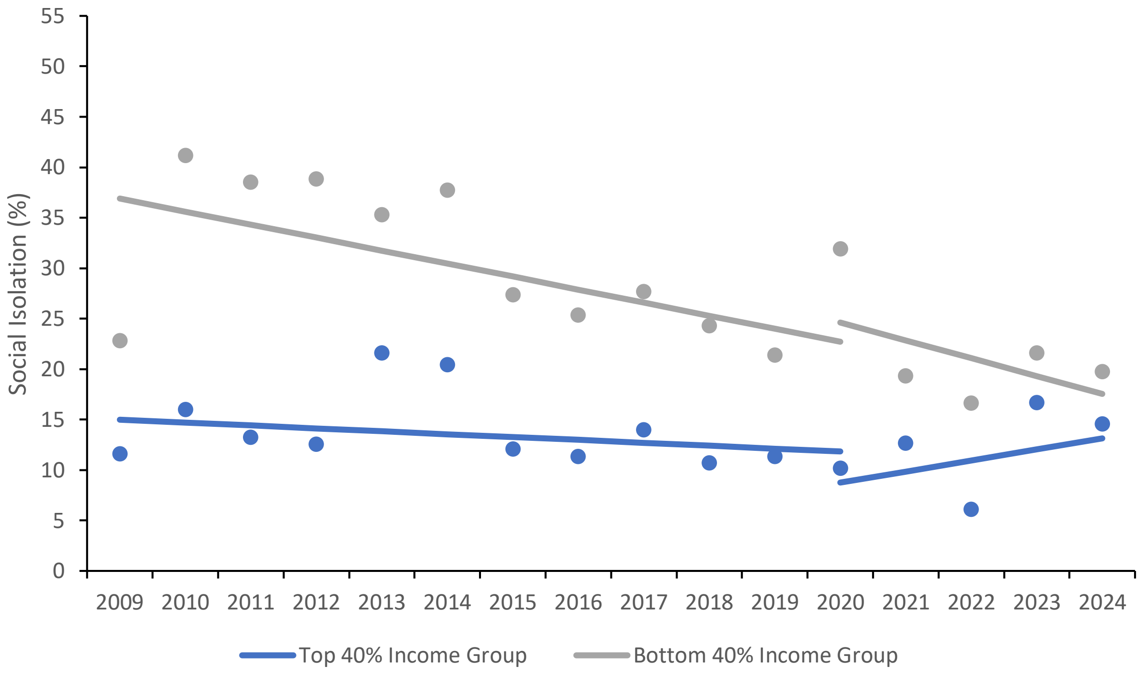

© 2025 Fuller-Rowell TE et al. *JAMA Network Open.*

eFigure 141. Trends in Social Isolation for Bosnia Herzegovina by Income Group. Fitted trajectories are derived from empirical Bayes estimates of the final best fitting model, with raw data overlaid.

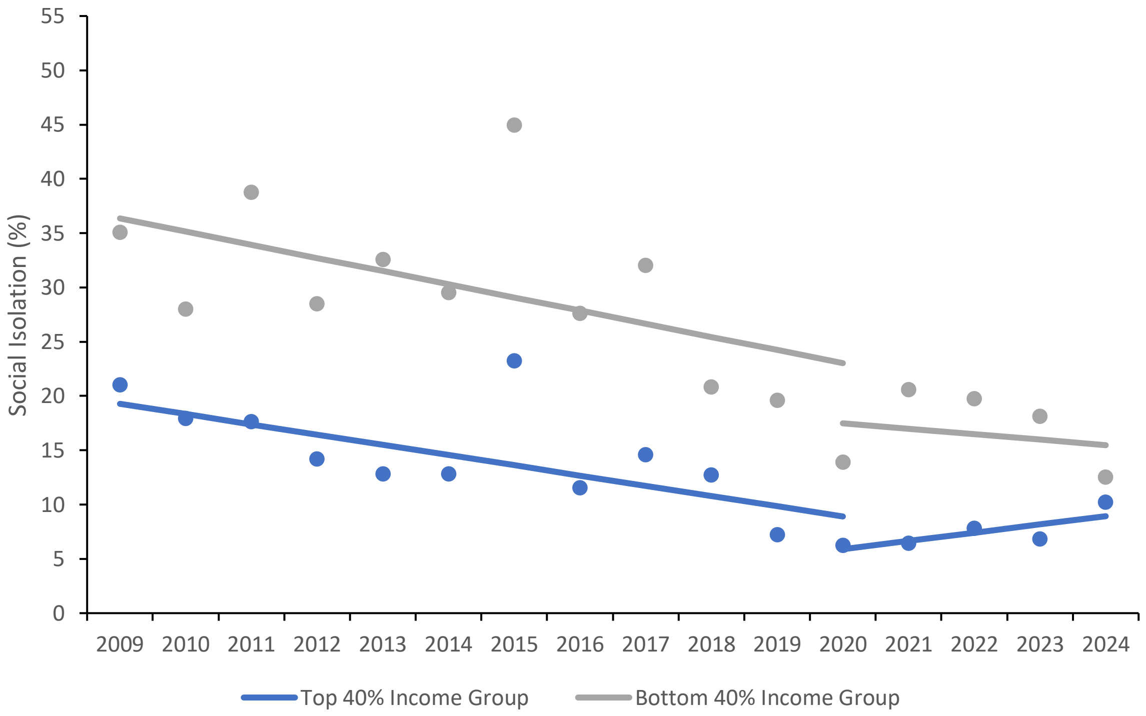

© 2025 Fuller-Rowell TE et al. *JAMA Network Open.*

eFigure 142. Trends in Social Isolation for Hong Kong by Income Group. Fitted trajectories are derived from empirical Bayes estimates of the final best fitting model, with raw data overlaid.

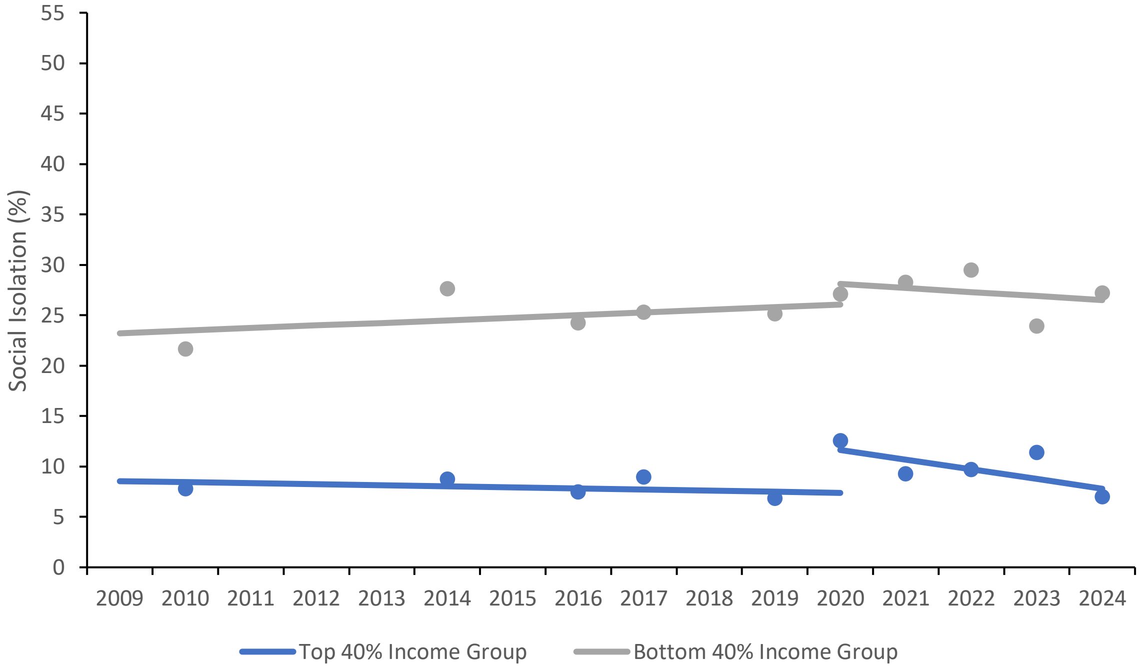

© 2025 Fuller-Rowell TE et al. *JAMA Network Open.*

eFigure 143. Trends in Social Isolation for Japan by Income Group. Fitted trajectories are derived from empirical Bayes estimates of the final best fitting model, with raw data overlaid.

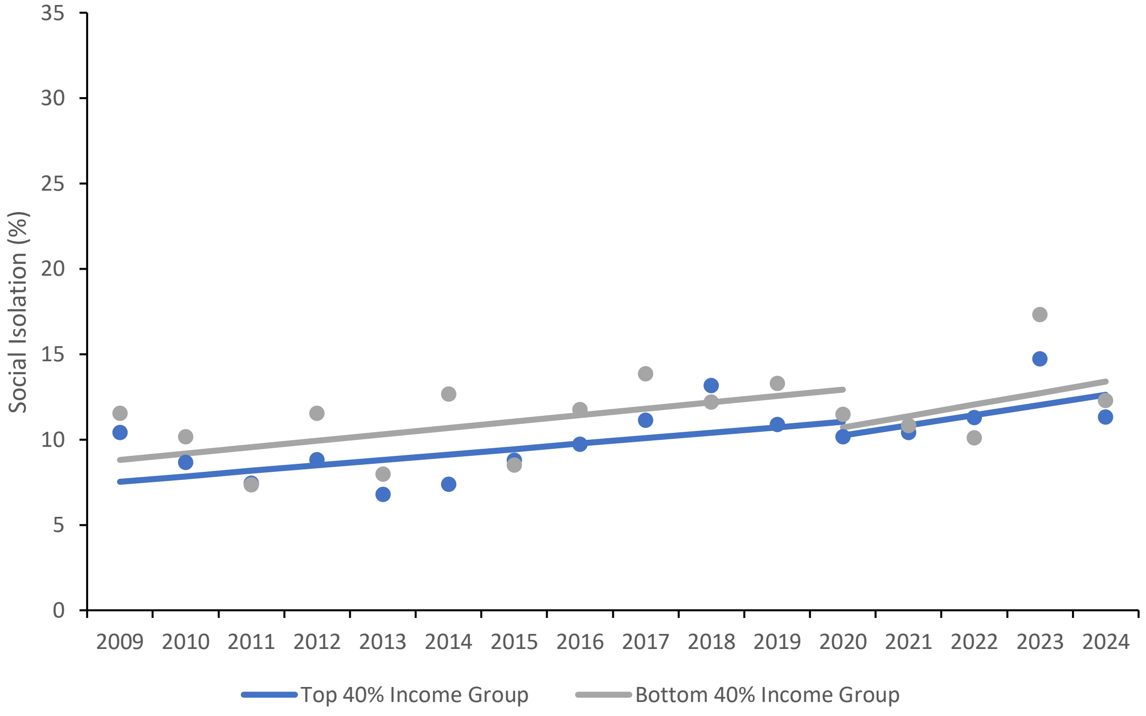

© 2025 Fuller-Rowell TE et al. *JAMA Network Open.*

eFigure 144. Trends in Social Isolation for South Korea by Income Group. Fitted trajectories are derived from empirical Bayes estimates of the final best fitting model, with raw data overlaid.

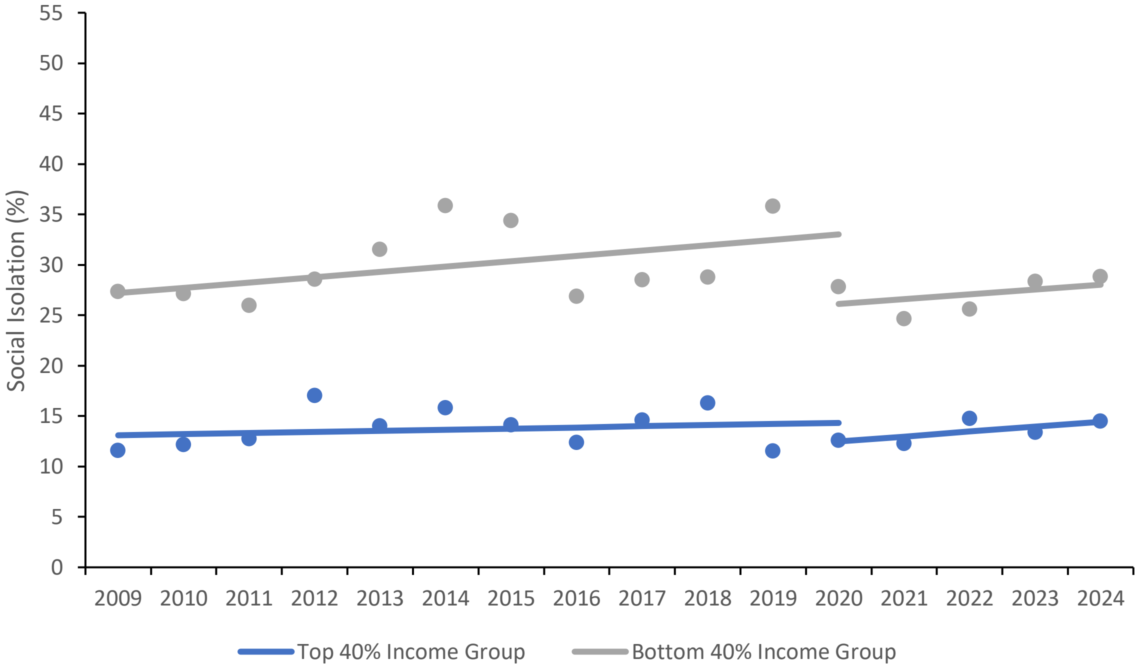

© 2025 Fuller-Rowell TE et al. *JAMA Network Open.*

eFigure 145. Trends in Social Isolation for Mongolia by Income Group. Fitted trajectories are derived from empirical Bayes estimates of the final best fitting model, with raw data overlaid.

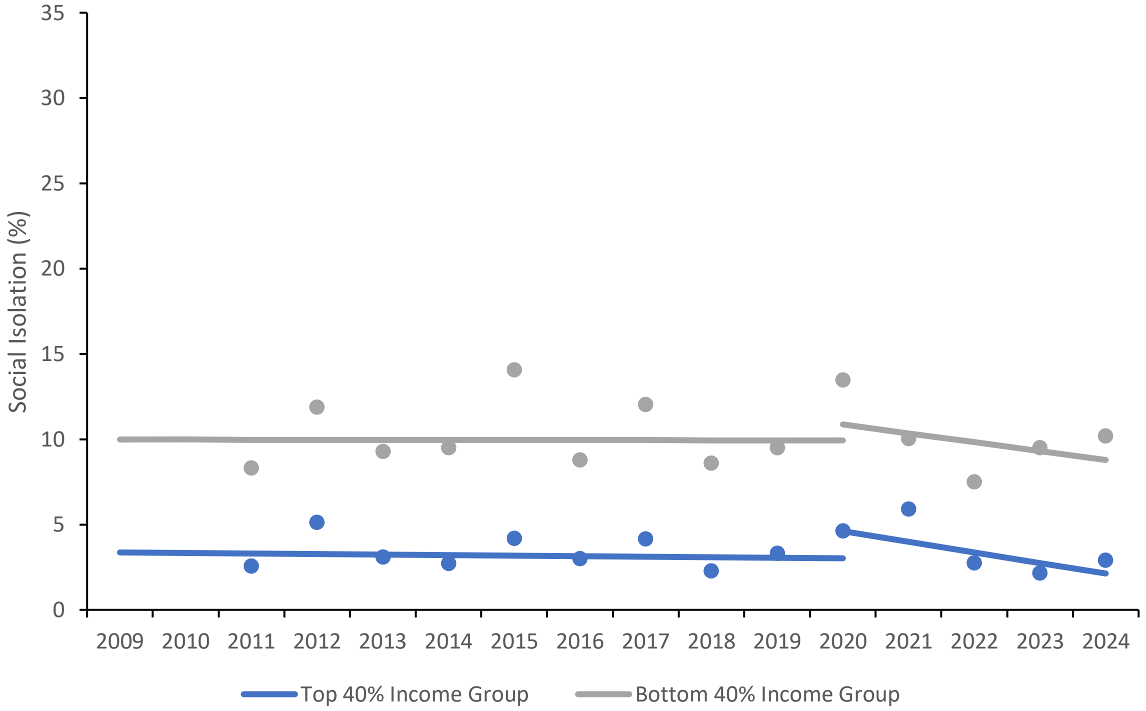

© 2025 Fuller-Rowell TE et al. *JAMA Network Open.*

eFigure 146. Trends in Social Isolation for Taiwan by Income Group. Fitted trajectories are derived from empirical Bayes estimates of the final best fitting model, with raw data overlaid.

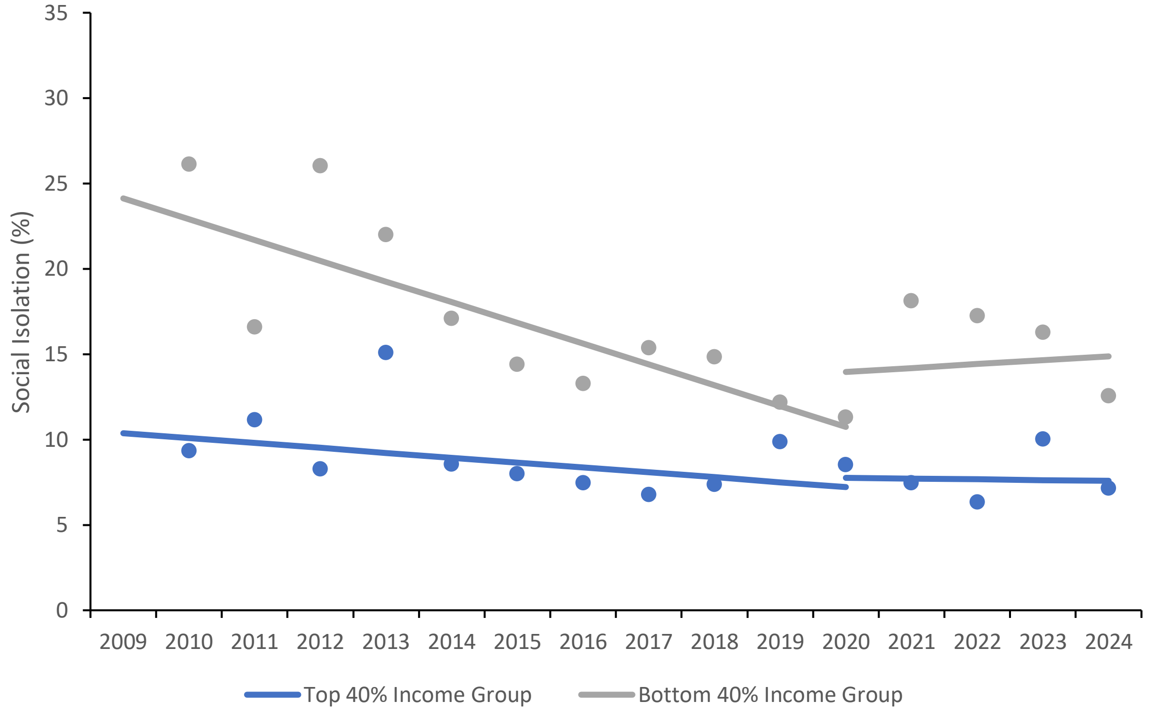

© 2025 Fuller-Rowell TE et al. *JAMA Network Open.*

eFigure 147. Trends in Social Isolation for China by Income Group. Fitted trajectories are derived from empirical Bayes estimates of the final best fitting model, with raw data overlaid.

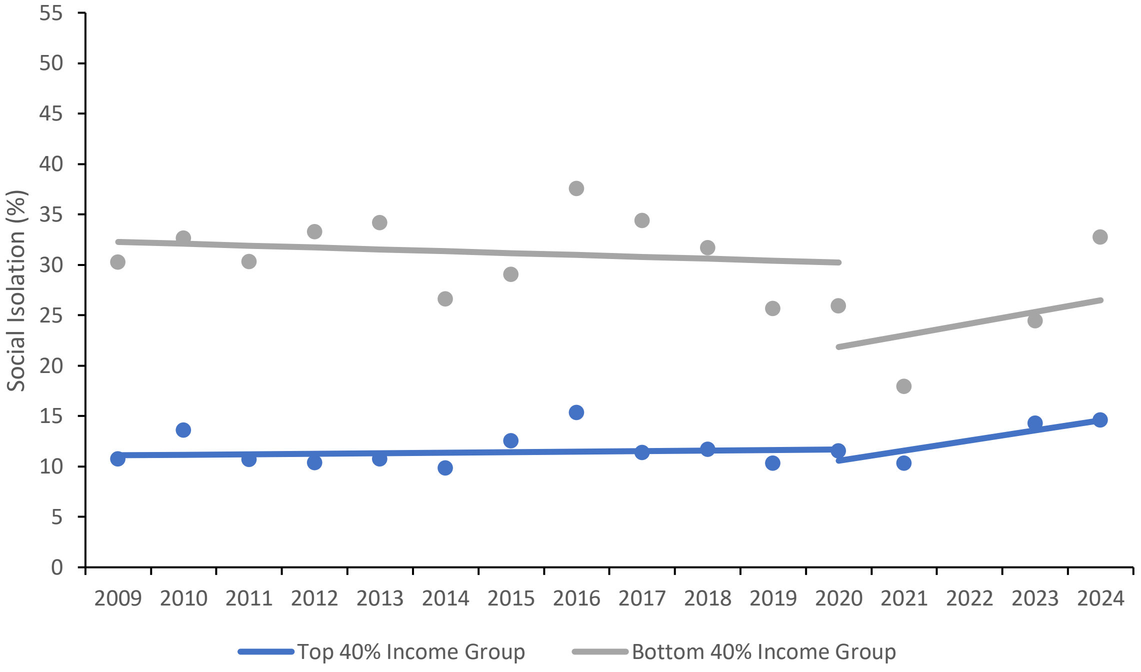

© 2025 Fuller-Rowell TE et al. *JAMA Network Open.*

eFigure 148. Trends in Social Isolation for Azerbizan by Income Group. Fitted trajectories are derived from empirical Bayes estimates of the final best fitting model, with raw data overlaid.

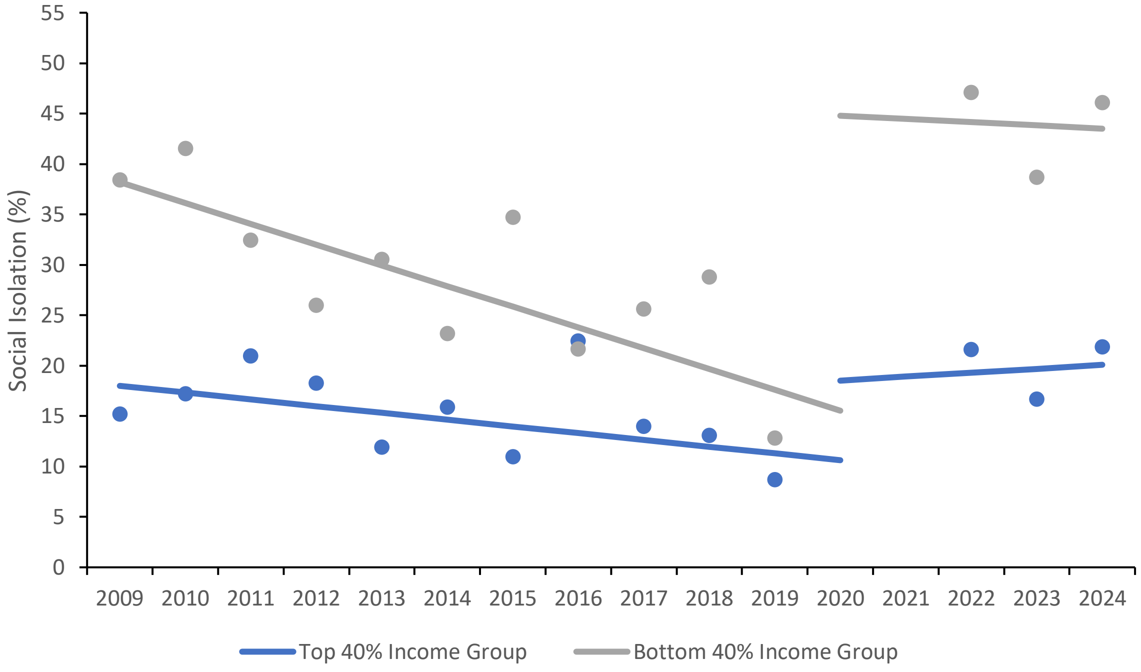

© 2025 Fuller-Rowell TE et al. *JAMA Network Open.*

eFigure 149. Trends in Social Isolation for Russia by Income Group. Fitted trajectories are derived from empirical Bayes estimates of the final best fitting model, with raw data overlaid.

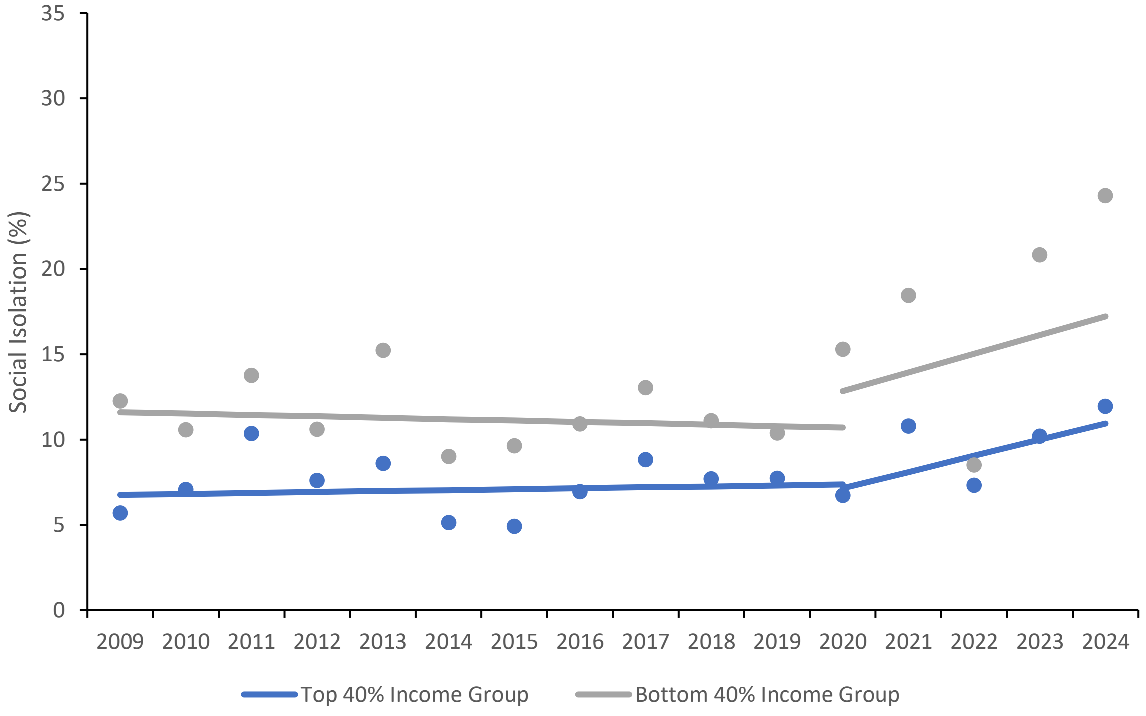

© 2025 Fuller-Rowell TE et al. *JAMA Network Open.*

eFigure 150. Trends in Social Isolation for Uzbekistan by Income Group. Fitted trajectories are derived from empirical Bayes estimates of the final best fitting model, with raw data overlaid.

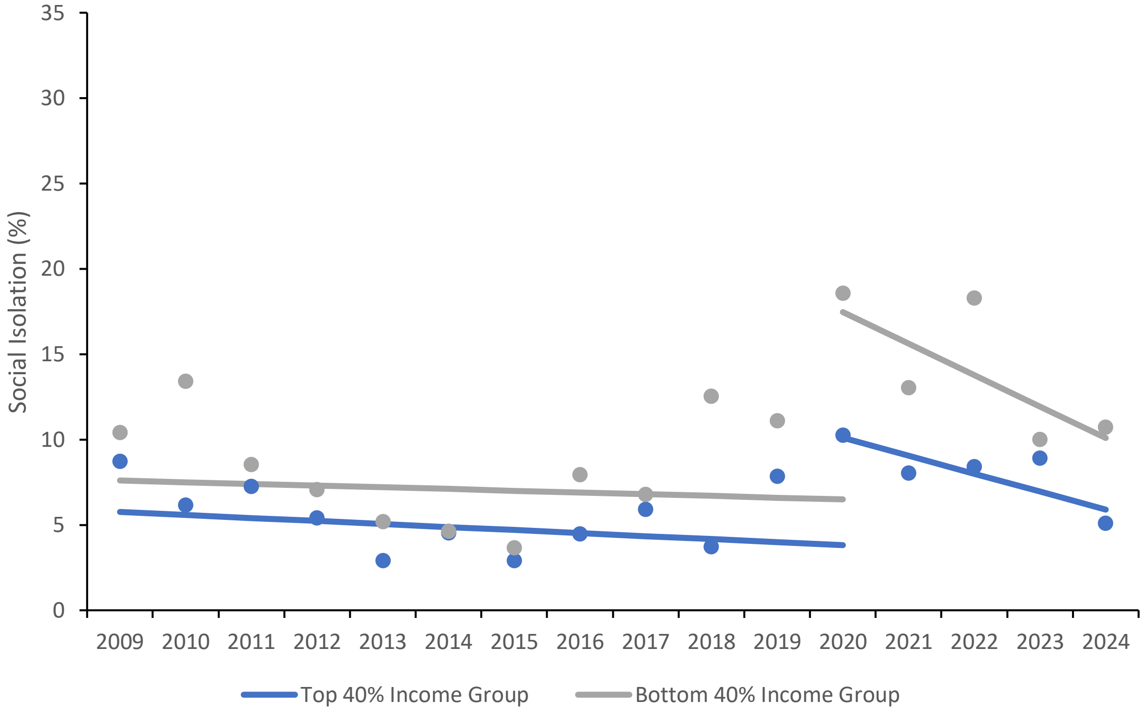

© 2025 Fuller-Rowell TE et al. *JAMA Network Open.*

eFigure 151. Trends in Social Isolation for Moldova by Income Group. Fitted trajectories are derived from empirical Bayes estimates of the final best fitting model, with raw data overlaid.

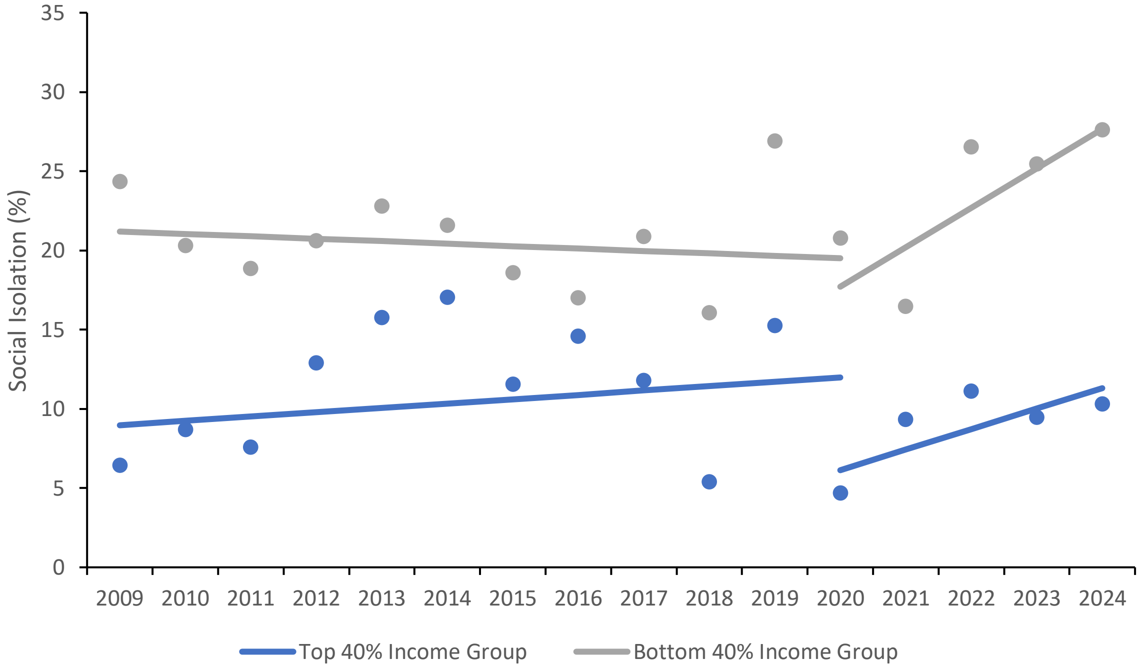

© 2025 Fuller-Rowell TE et al. *JAMA Network Open.*

eFigure 152. Trends in Social Isolation for Ukraine by Income Group. Fitted trajectories are derived from empirical Bayes estimates of the final best fitting model, with raw data overlaid.

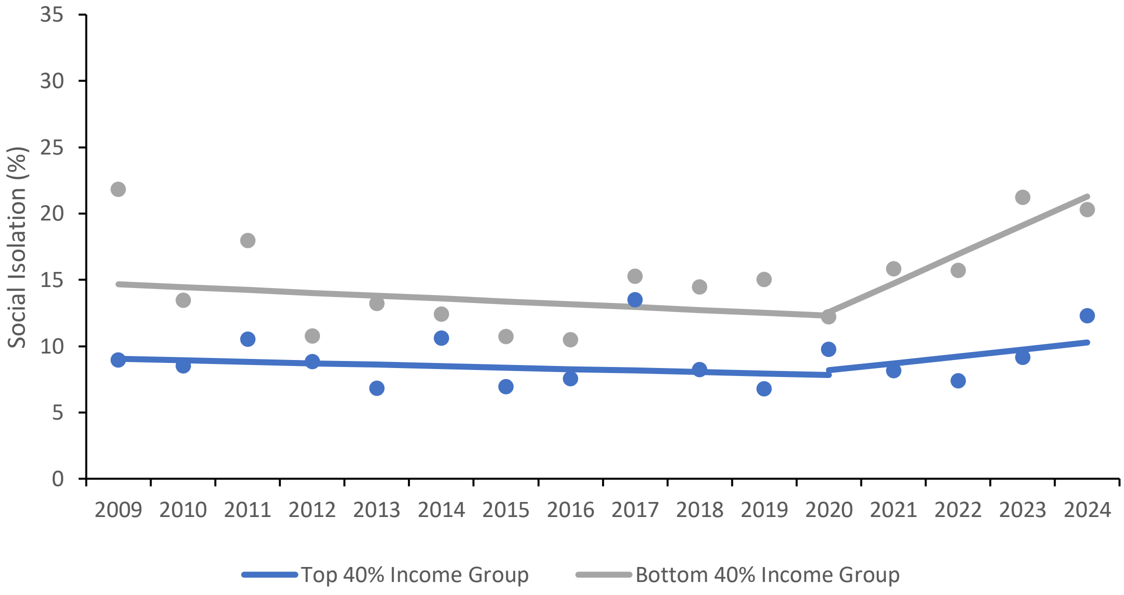

© 2025 Fuller-Rowell TE et al. *JAMA Network Open.*

eFigure 153. Trends in Social Isolation for Turkmenistan by Income Group. Fitted trajectories are derived from empirical Bayes estimates of the final best fitting model, with raw data overlaid.

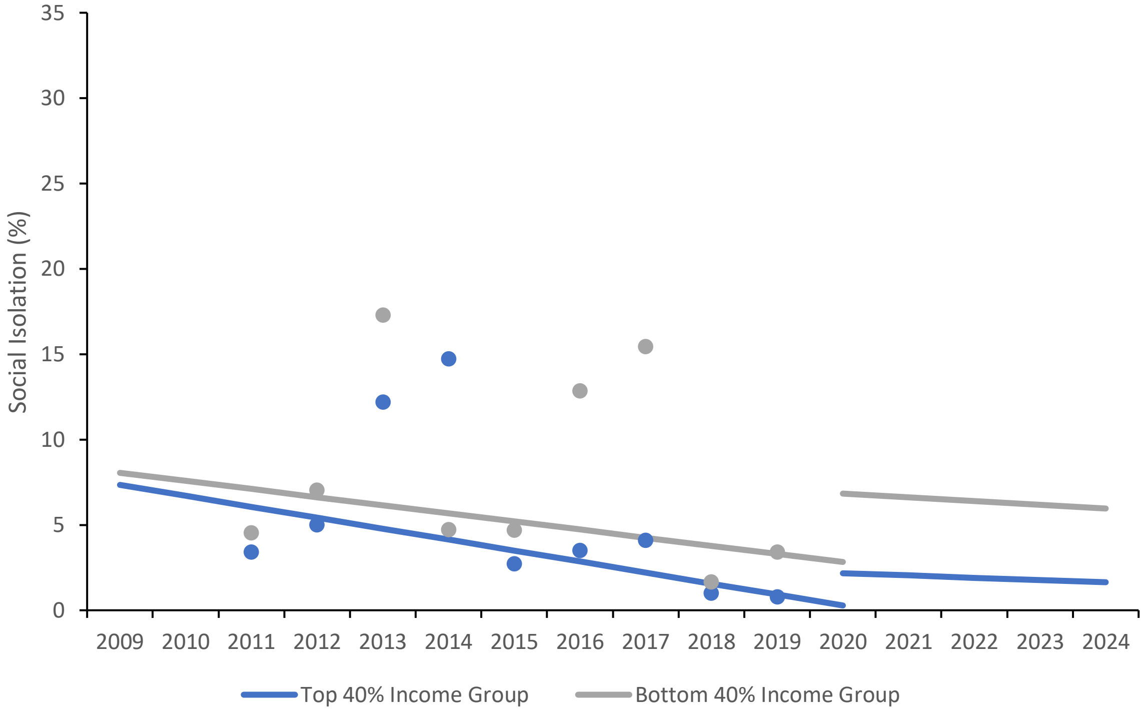

© 2025 Fuller-Rowell TE et al. *JAMA Network Open.*

eFigure 154. Trends in Social Isolation for Belarus by Income Group. Fitted trajectories are derived from empirical Bayes estimates of the final best fitting model, with raw data overlaid.

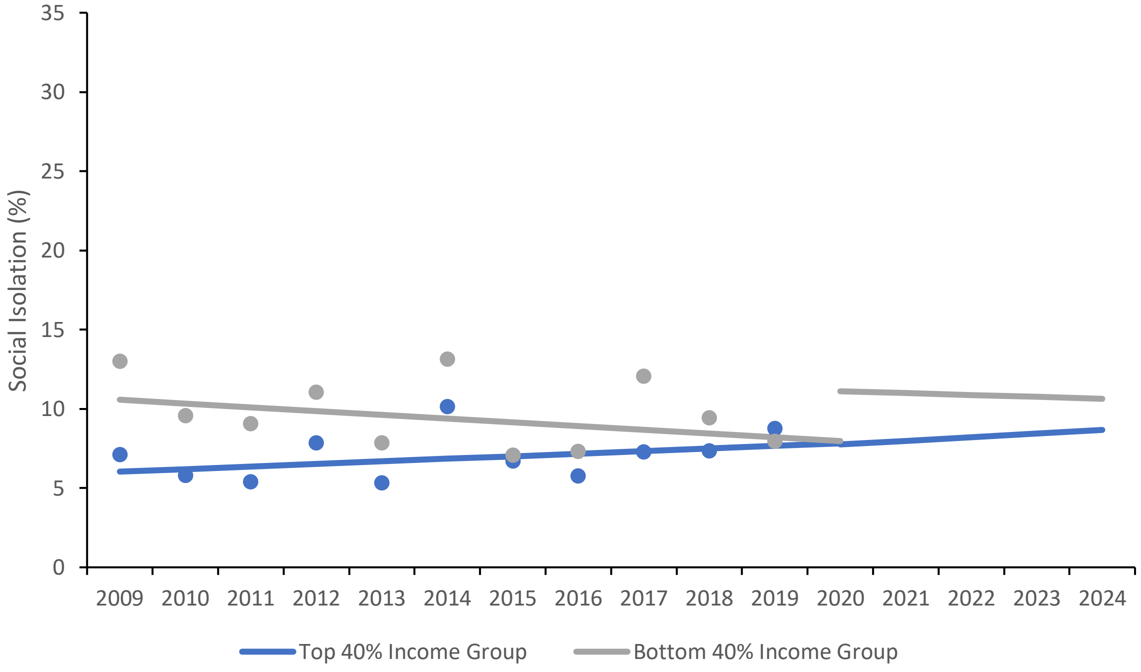

© 2025 Fuller-Rowell TE et al. *JAMA Network Open.*

eFigure 155. Trends in Social Isolation for Kyrgyzstan by Income Group. Fitted trajectories are derived from empirical Bayes estimates of the final best fitting model, with raw data overlaid.

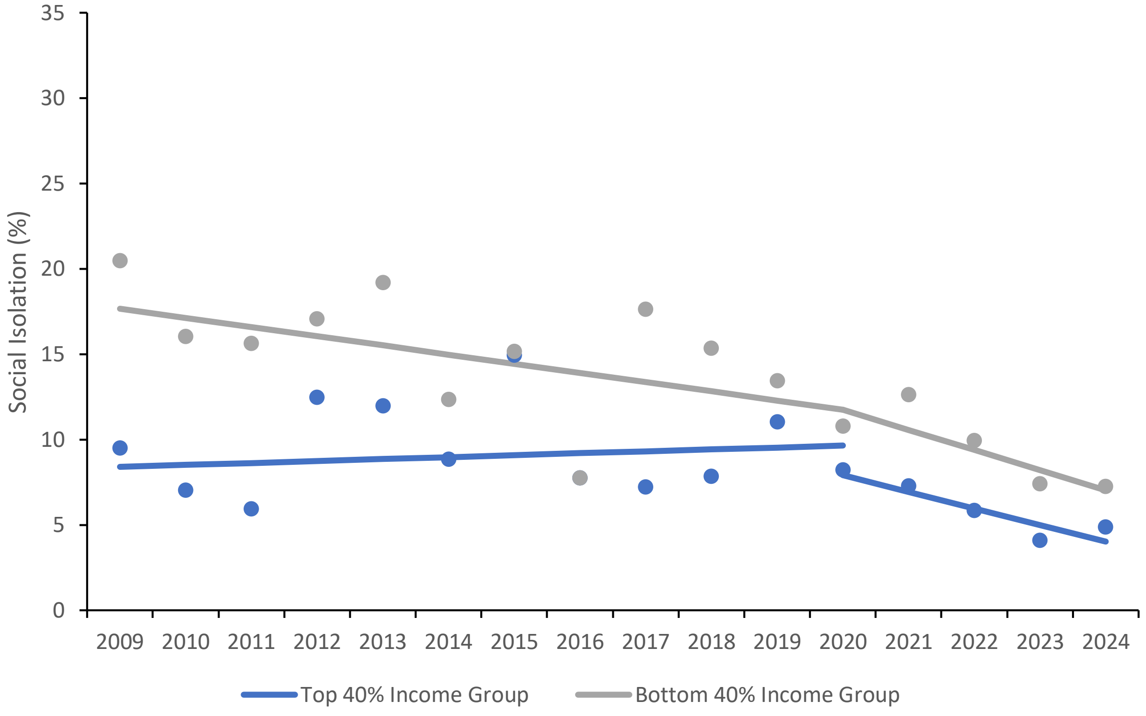

© 2025 Fuller-Rowell TE et al. *JAMA Network Open.*

eFigure 156. Trends in Social Isolation for Kazakhstan by Income Group. Fitted trajectories are derived from empirical Bayes estimates of the final best fitting model, with raw data overlaid.

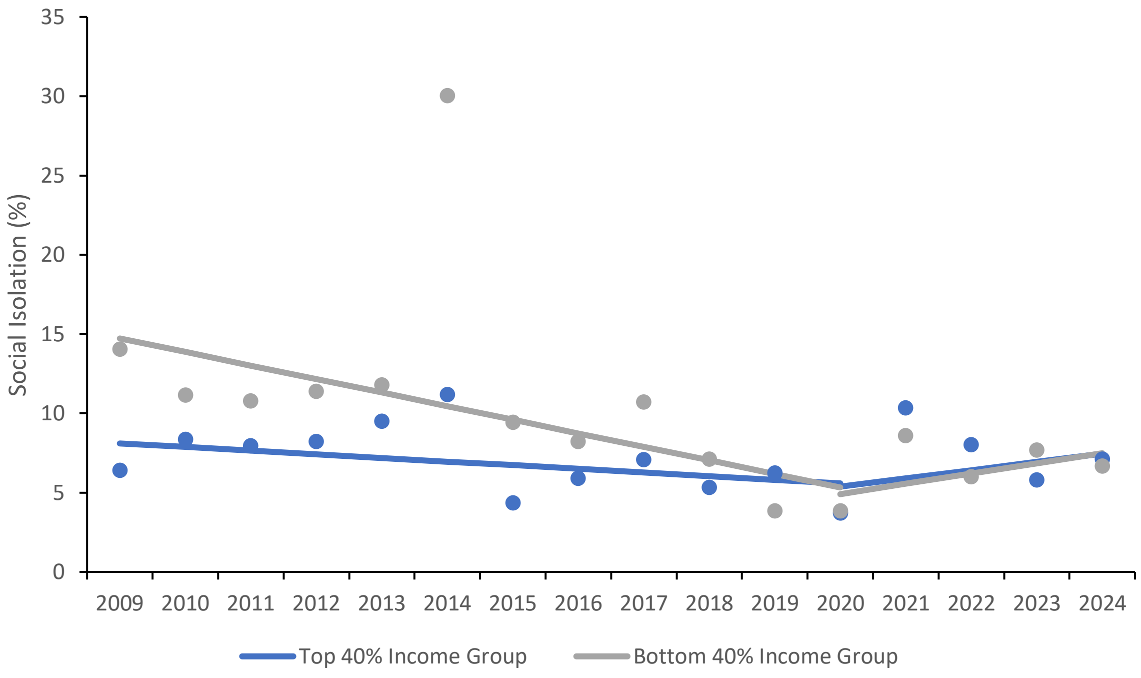

© 2025 Fuller-Rowell TE et al. *JAMA Network Open.*

eFigure 157. Trends in Social Isolation for Armenia by Income Group. Fitted trajectories are derived from empirical Bayes estimates of the final best fitting model, with raw data overlaid.

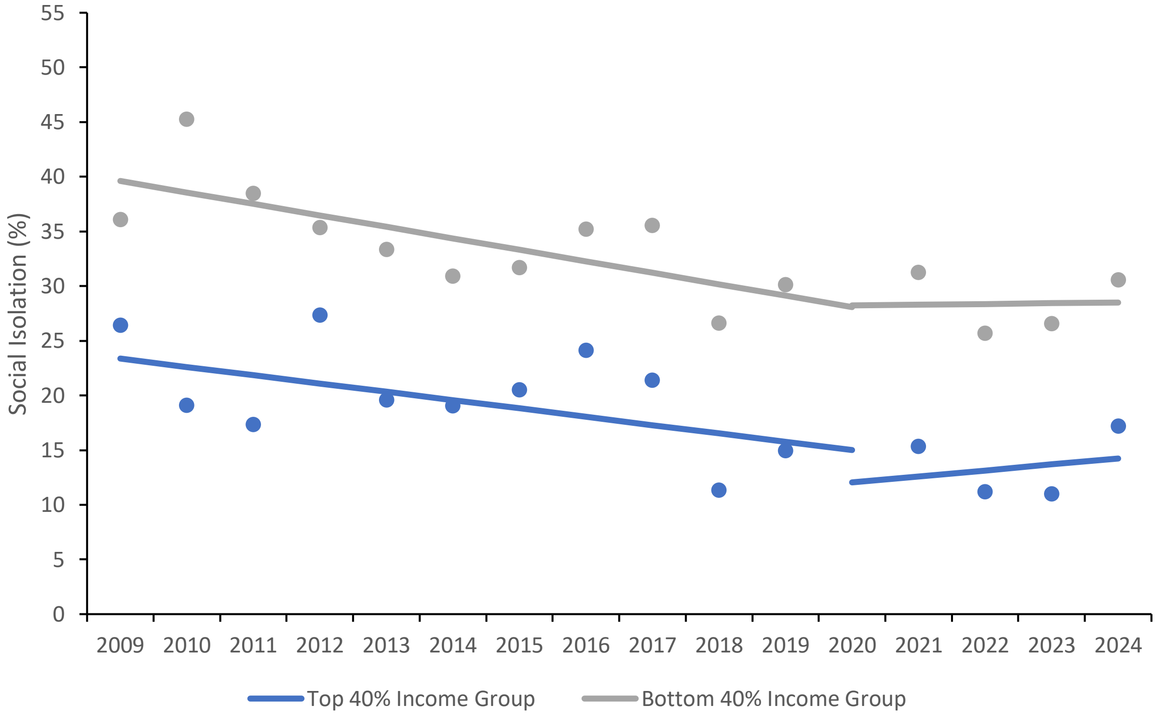

© 2025 Fuller-Rowell TE et al. *JAMA Network Open.*

eFigure 158. Trends in Social Isolation for Tajikistan by Income Group. Fitted trajectories are derived from empirical Bayes estimates of the final best fitting model, with raw data overlaid.

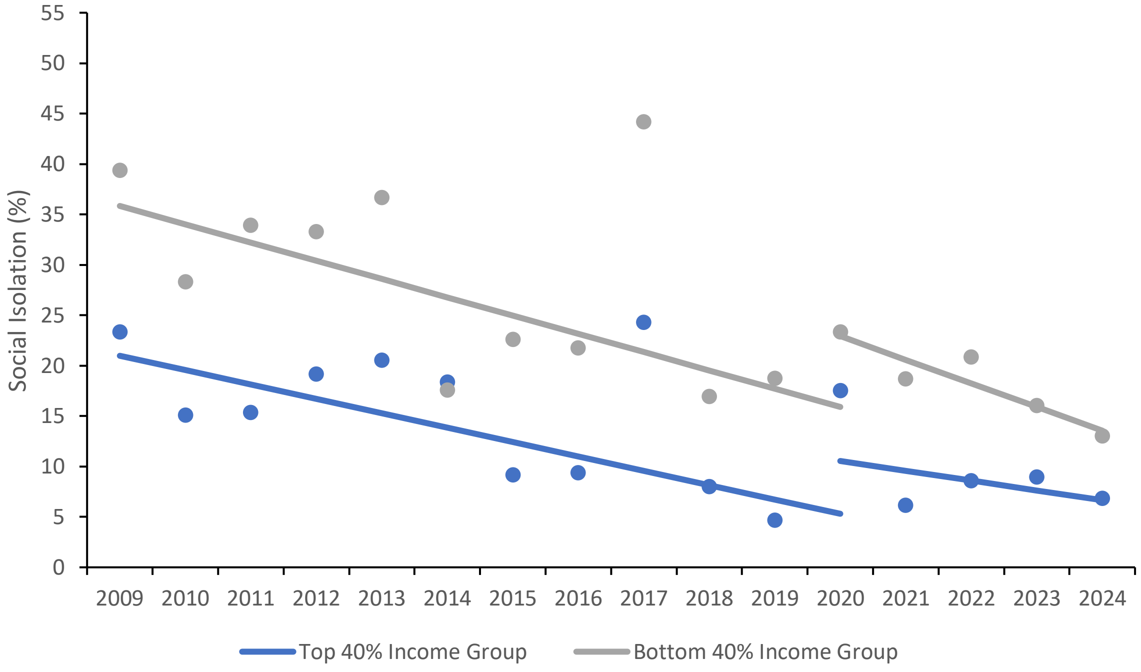

© 2025 Fuller-Rowell TE et al. *JAMA Network Open.*

eFigure 159. Trends in Social Isolation for Georgia by Income Group. Fitted trajectories are derived from empirical Bayes estimates of the final best fitting model, with raw data overlaid.

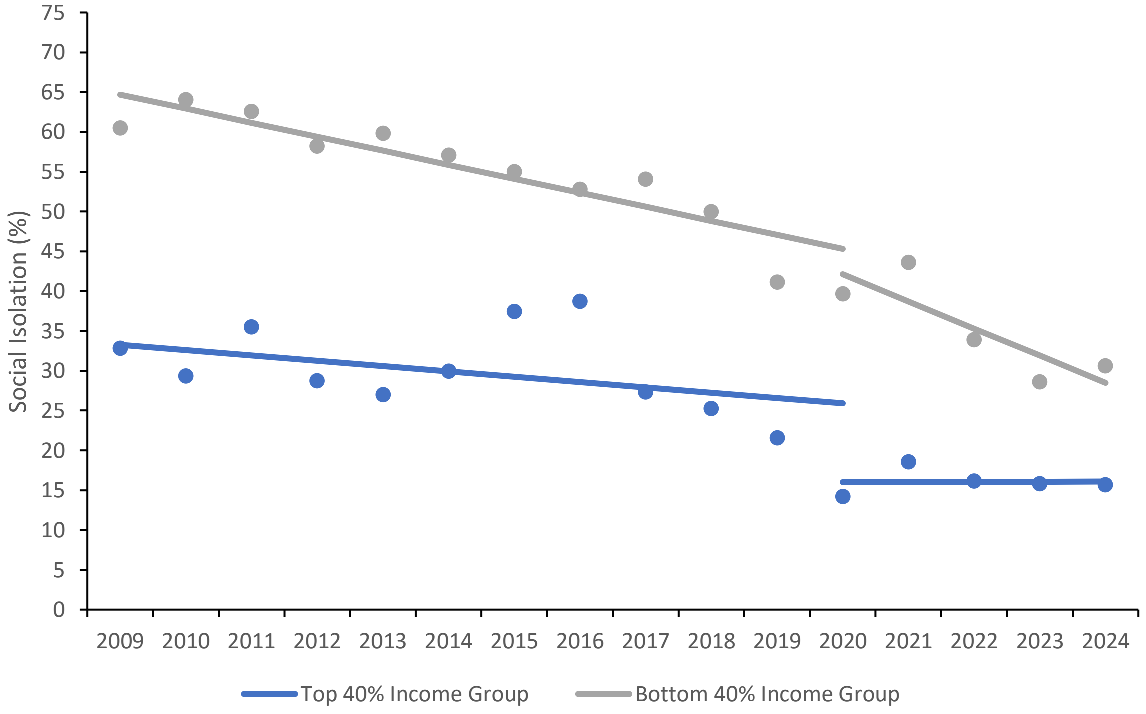

© 2025 Fuller-Rowell TE et al. *JAMA Network Open.*

eFigure 160. Trends in Social Isolation for Sub-Saharan Africa by Income Group. Fitted trajectories are derived from empirical empirical Bayes estimates of the final best fitting model, with raw data overlaid.

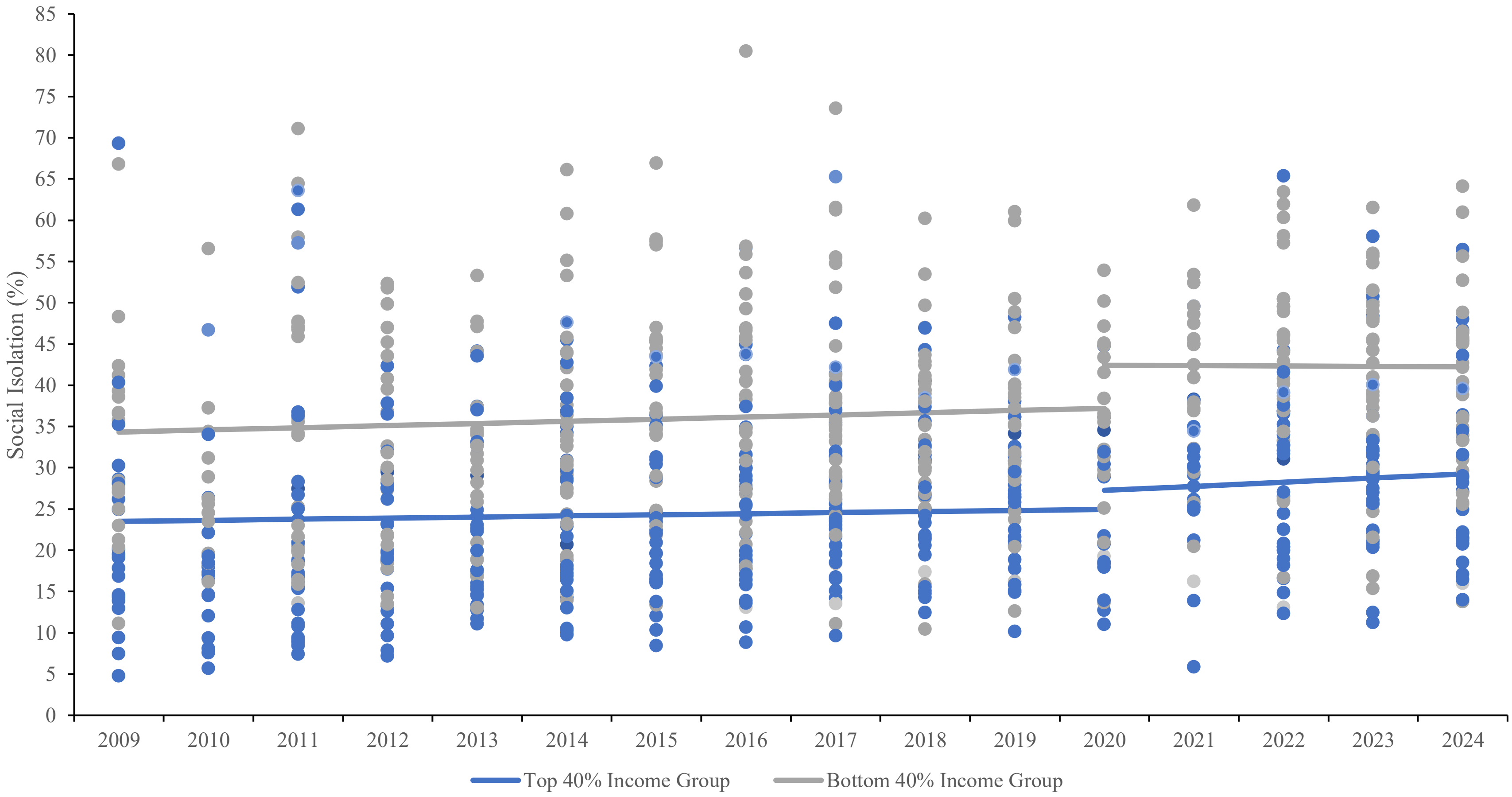

© 2025 Fuller-Rowell TE et al. *JAMA Network Open.*

eFigure 161. Trends in Social Isolation for South Asia by Income Group. Fitted trajectories are derived from empirical Bayes estimates of the final best fitting model, with raw data overlaid.

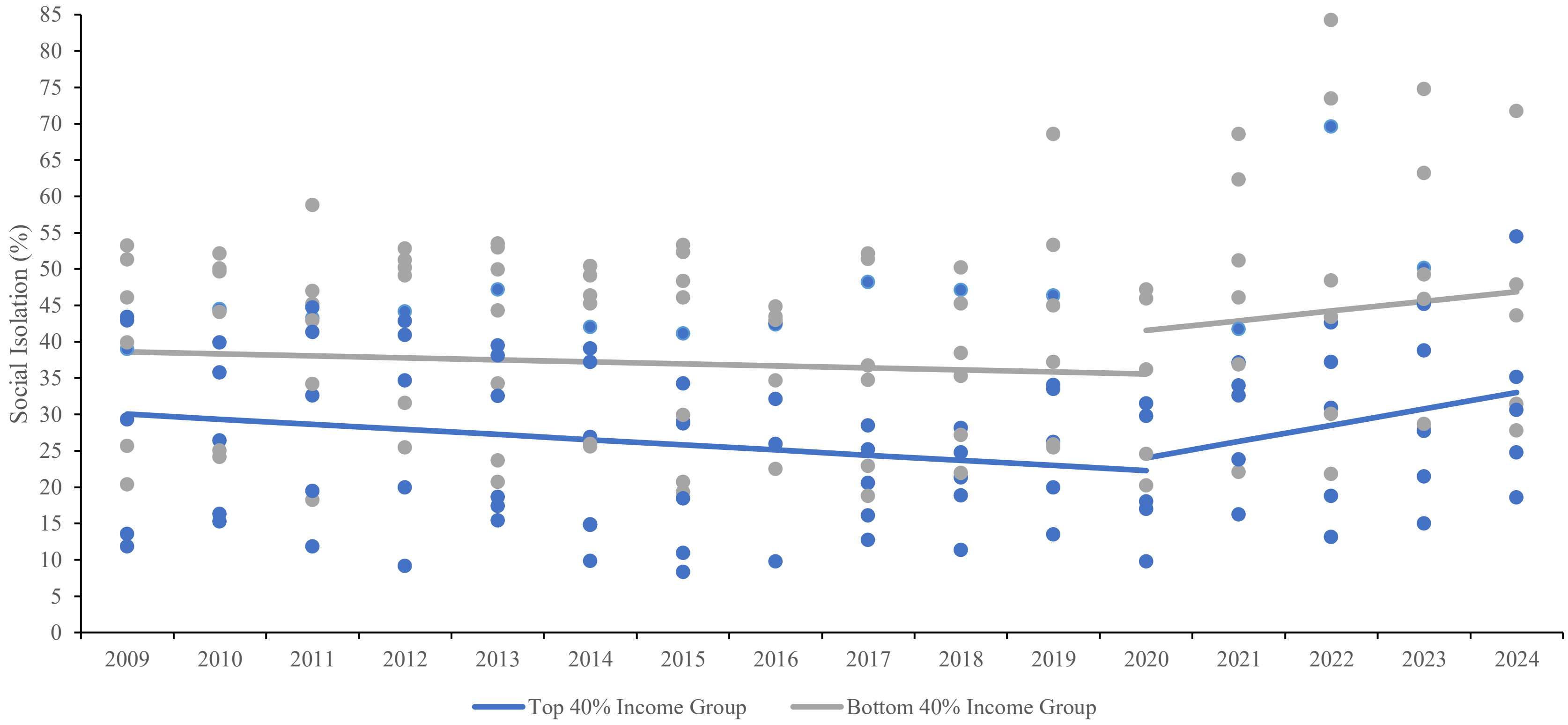

© 2025 Fuller-Rowell TE et al. *JAMA Network Open.*

eFigure 162. Trends in Social Isolation for Latin America and the Caribbean (LAC) by Income Group. Fitted trajectories are derived from empirical Bayes estimates of the final best fitting model, with raw data overlaid.

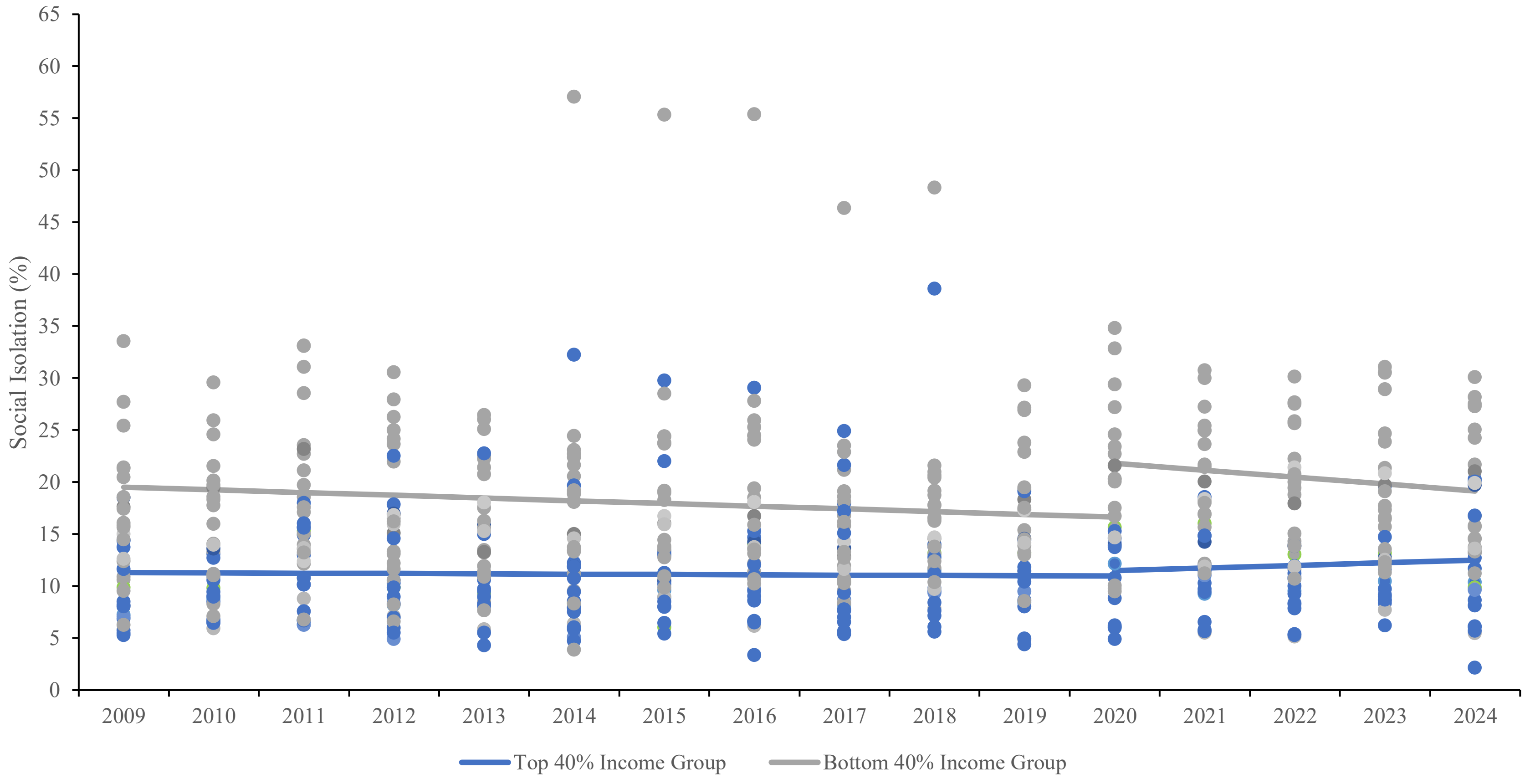

© 2025 Fuller-Rowell TE et al. *JAMA Network Open.*

eFigure 163. Trends in Social Isolation for Middle East and North Africa (MENA) by Income Group. Fitted trajectories are derived from empirical Bayes estimates of the final best fitting model, with raw data overlaid.

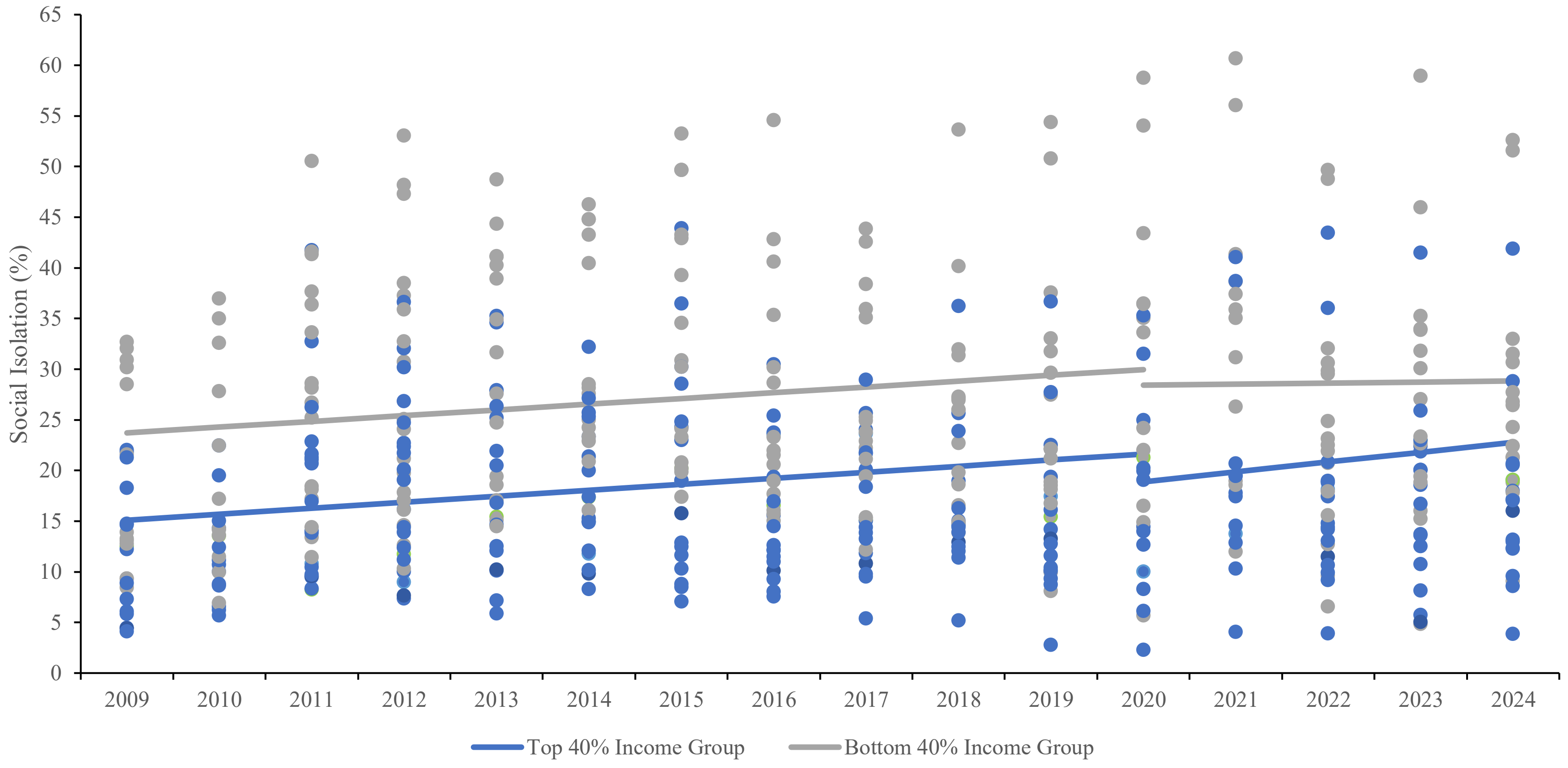

© 2025 Fuller-Rowell TE et al. *JAMA Network Open.*

eFigure 164. Trends in Social Isolation for North America by Income Group. Fitted trajectories are derived from empirical Bayes estimates of the final best fitting model, with raw data overlaid.

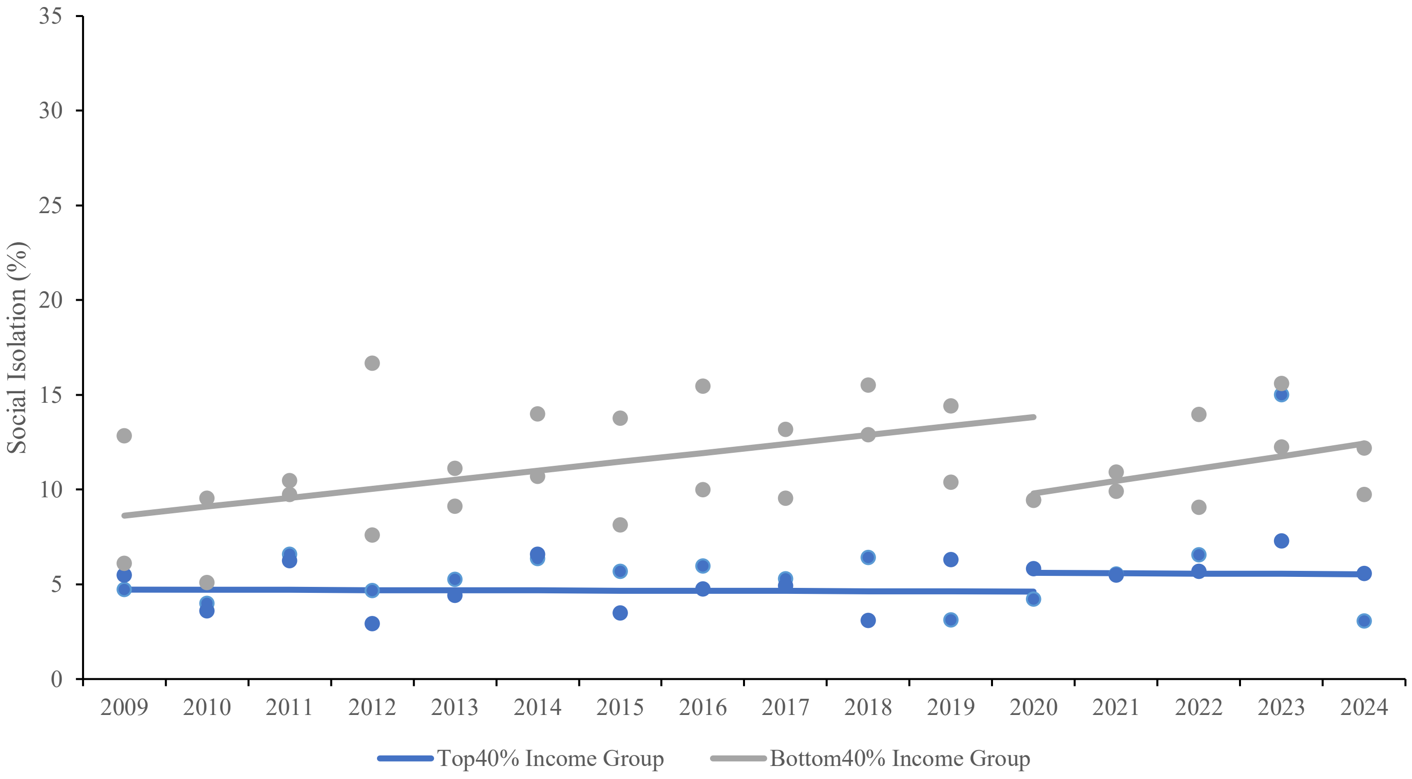

© 2025 Fuller-Rowell TE et al. *JAMA Network Open.*

eFigure 165. Trends in Social Isolation for ANZ (Australia and New Zealand) by Income Group. Fitted trajectories are derived from empirical Bayes estimates of the final best fitting model, with raw data overlaid.

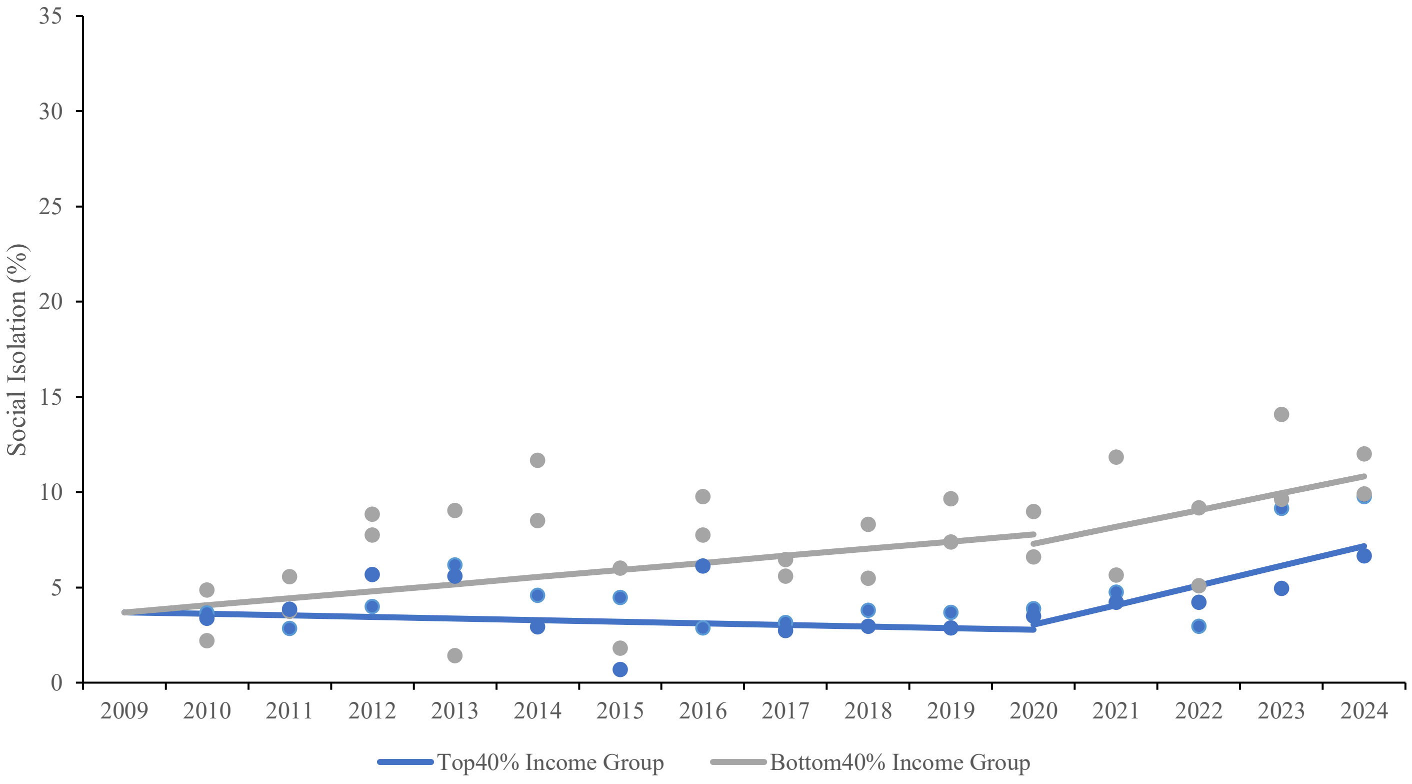

© 2025 Fuller-Rowell TE et al. *JAMA Network Open.*

eFigure 166. Trends in Social Isolation for Southeast Asia by Income Group. Fitted trajectories are derived from empirical Bayes estimates of the final best fitting model, with raw data overlaid.

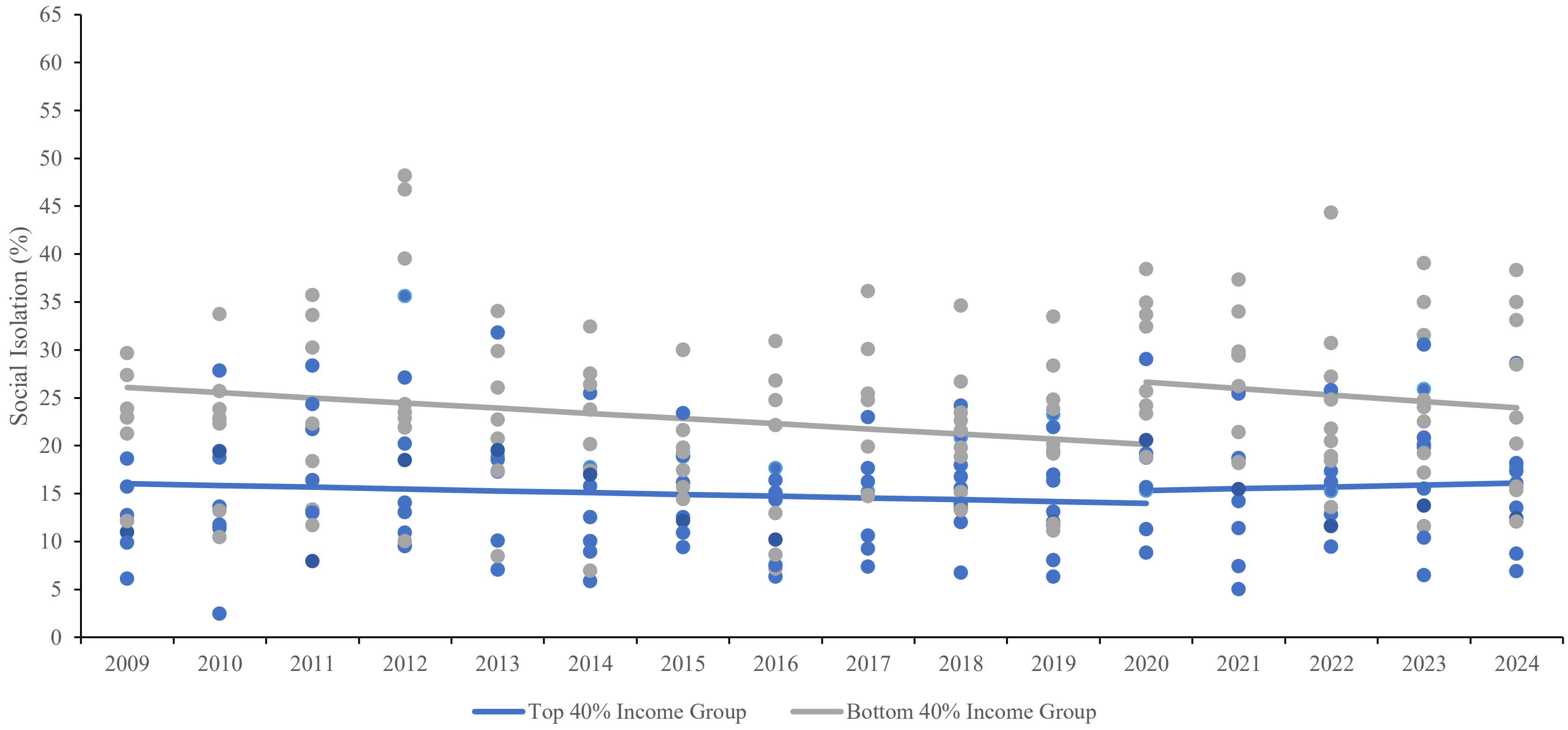

© 2025 Fuller-Rowell TE et al. *JAMA Network Open.*

eFigure 167. Trends in Social Isolation for Europe by Income Group. Fitted trajectories are derived from empirical Bayes estimates of the final best fitting model, with raw data overlaid.

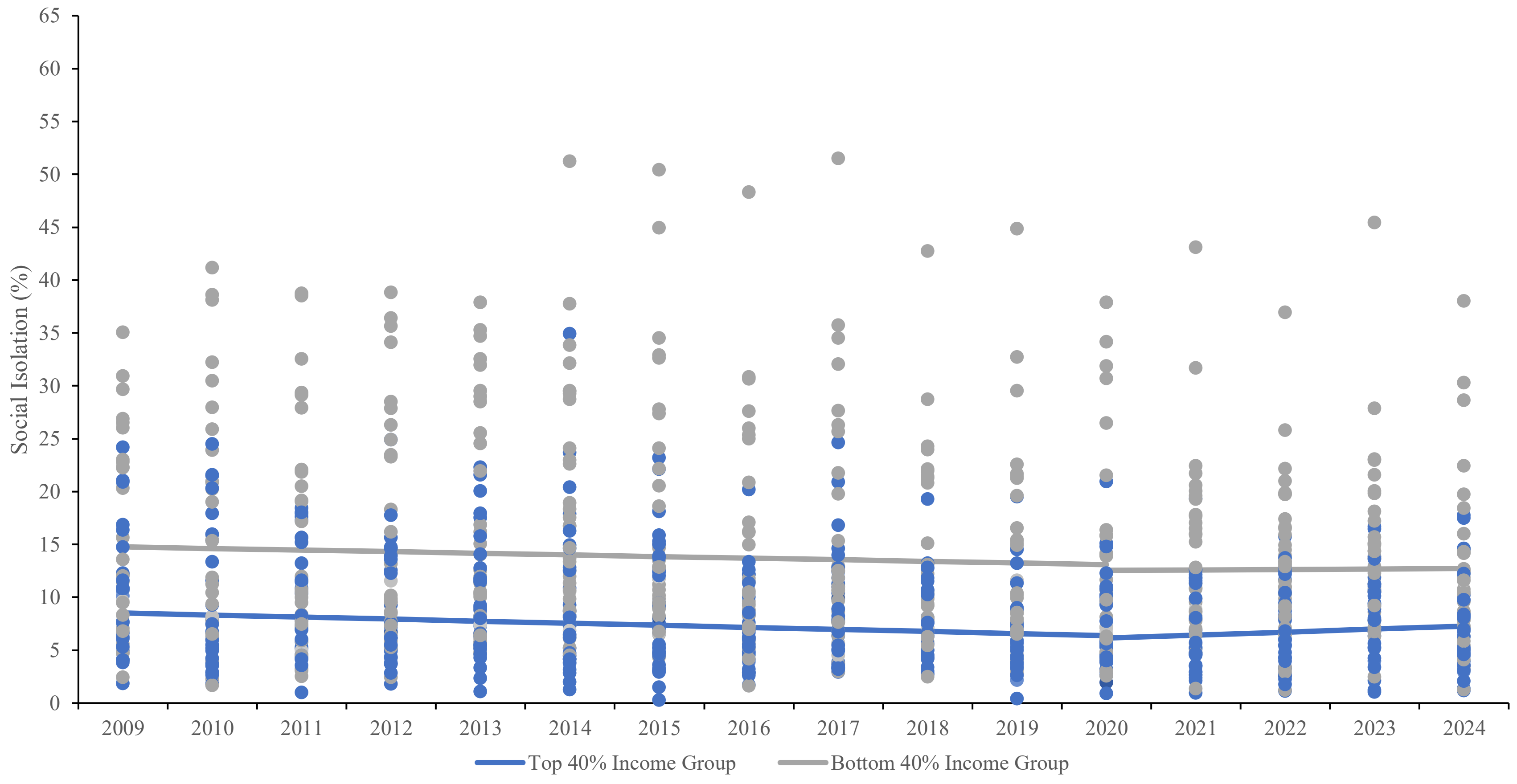

© 2025 Fuller-Rowell TE et al. *JAMA Network Open.*

eFigure 168. Trends in Social Isolation for East Asia by Income Group. Fitted trajectories are derived from empirical Bayes estimates of the final best fitting model, with raw data overlaid.

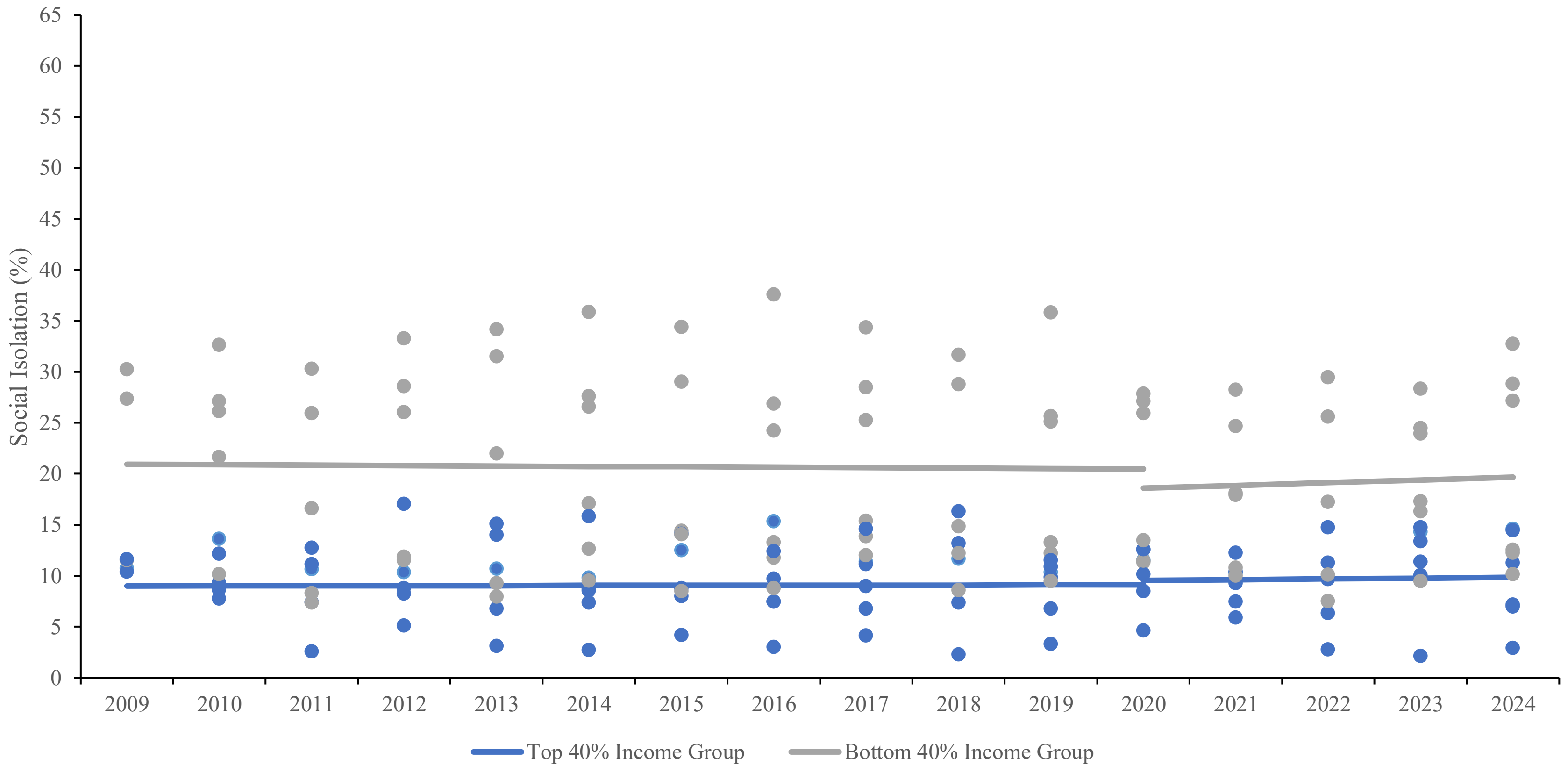

© 2025 Fuller-Rowell TE et al. *JAMA Network Open.*

eFigure 169. Trends in Social Isolation for RFSU (Russia and the Former Soviet Union) by Income Group. Fitted trajectories are derived from empirical Bayes estimates of the final best fitting model, with raw data overlaid.

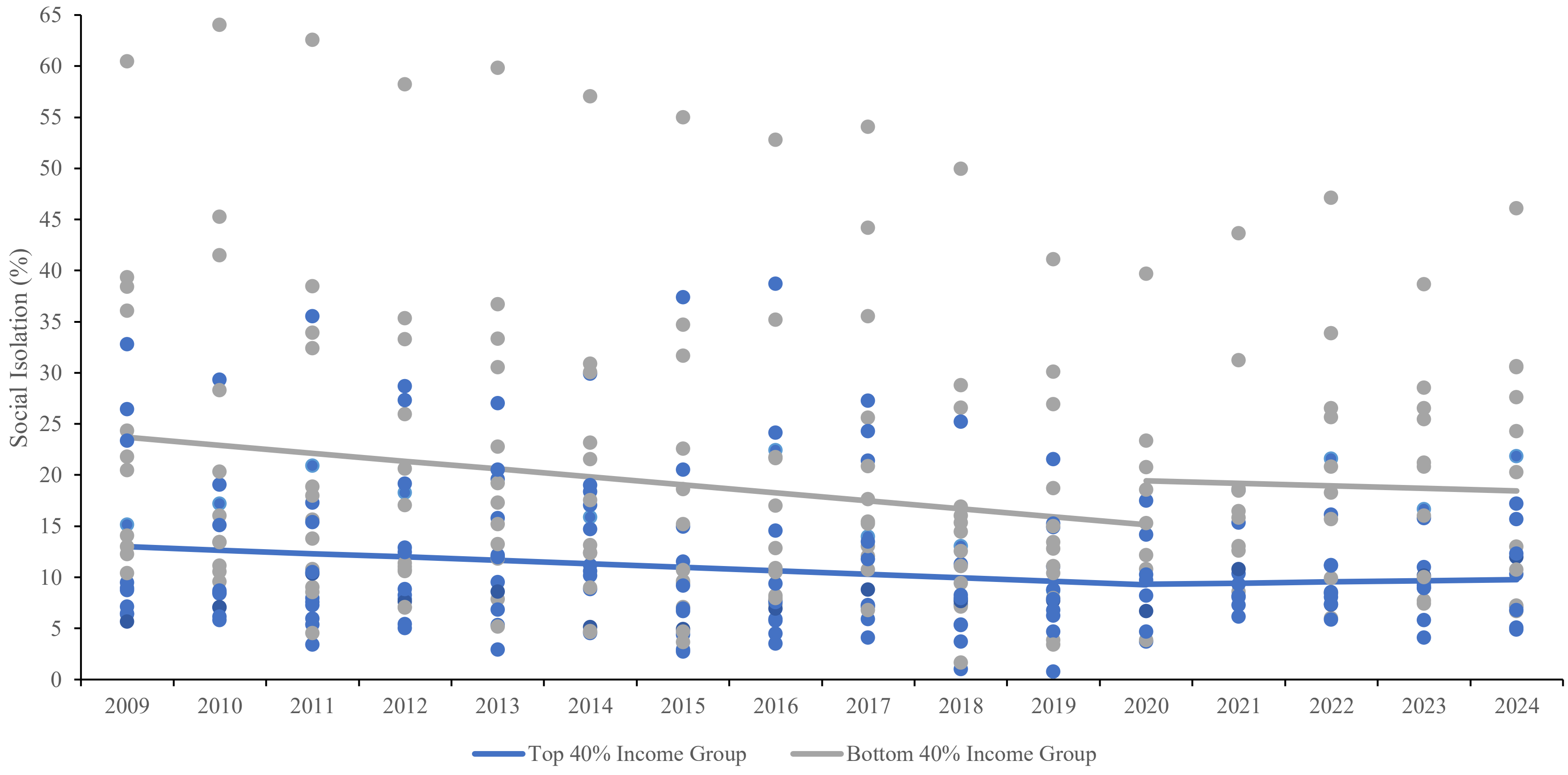

© 2025 Fuller-Rowell TE et al. *JAMA Network Open.*

eTable 1. Raw Global Descriptive Statistics for Social Isolation at Each Timepoint

Year	Global (M ± SD)	Top 40% (M ± SD)	Bottom 40% (M ± SD)
2009	18.01 ± 10.96	14.1 ± 10.44	22.49 ± 12.61
2010	16.54 ± 10.34	12.55 ± 8.66	20.56 ± 12.68
2011	19.39 ± 12.57	15.35 ± 11.78	23.29 ± 14.45
2012	18.9 ± 11.25	14.79 ± 9.79	22.92 ± 13.68
2013	19.24 ± 10.94	15.05 ± 9.58	22.99 ± 12.96
2014	19.2 ± 11.37	14.7 ± 9.93	23.89 ± 13.50
2015	19.97 ± 12.41	15.01 ± 10.18	24.67 ± 15.03
2016	18.65 ± 12.48	14.52 ± 10.77	23.01 ± 14.59
2017	19.3 ± 12.13	15.19 ± 10.59	23.66 ± 14.38
2018	18.59 ± 11.4	14.73 ± 10.19	22.54 ± 12.88
2019	18.25 ± 11.75	14.18 ± 10.08	22.66 ± 13.65
2020	17.53 ± 11.07	12.89 ± 8.96	22.20 ± 13.67
2021	19.49 ± 12.7	14.47 ± 10.65	24.60 ± 14.83
2022	20.03 ± 13.81	15.54 ± 12.13	24.94 ± 15.91
2023	20.91 ± 12.88	16.7 ± 11.31	25.50 ± 14.90
2024	20.21 ± 12.89	15.78 ± 11.36	24.87 ± 14.87

© 2025 Fuller-Rowell TE et al. *JAMA Network Open.*

eTable 2. Comparison of Fixed-Effect Estimates From the Weighted and Unweighted Models

	Weighted Model			Unweighted Model			(|Δβ|)
Parameters	Estimate	SE	*p*-value	Estimate	SE	*p*-value	
Full Sample							
1. Intercept (2009)	19.22	0.99	<.001	19.58	0.97	<.001	0.36
2. Linear trend	0.04	0.09	.681	-0.00	0.07	0.979	0.04
3. Step-change	1.47	0.63	.020	1.32	0.52	0.010	0.15
4. Slope-change	0.15	0.17	.395	0.19	0.15	0.195	0.04
Low Income							
1. Intercept (2009)	24.07	1.23	<.001	24.35	1.17	<.001	0.28
2. Linear trend	-0.03	0.11	.795	-0.05	0.08	0.593	0.02
3. Step-change	2.65	0.95	.005	2.38	0.71	<.001	0.27
4. Slope-change	-0.02	0.21	.908	0.06	0.18	0.758	0.08
High Income							
1. Intercept (2009)	15.29	0.93	<.001	15.62	0.89	<.001	0.33
2. Linear trend	-0.01	0.09	.886	-0.03	0.06	.658	0.02
3. Step-change	0.49	0.54	.360	0.42	0.44	.340	0.05
4. Slope-change	0.49	0.16	.002	0.43	0.14	.002	0.06

© 2025 Fuller-Rowell TE et al. *JAMA Network Open.*

eTable 3. Comparing Fixed-Effect Estimates From the Unrestricted and Diagonal G-Matrix Models

	Unrestricted G-Matrix Model			Diagonal G-matrix Model			(|Δβ|)
Parameters	Estimate	SE	*p*-value	Estimate	SE	*p*-value	
Full Sample							
1. Intercept (2009)	19.22	0.99	<.001	19.21	0.99	<.001	0.01
2. Linear trend	0.04	0.09	.681	0.04	0.09	0.654	0.00
3. Step-change	1.47	0.63	.020	1.62	0.61	0.008	0.15
4. Slope-change	0.15	0.17	.395	0.18	0.17	0.283	0.02
Low Income							
1. Intercept (2009)	24.07	1.23	<.001	24.01	1.24	<.001	0.05
2. Linear trend	-0.03	0.11	.795	-0.02	0.11	0.831	0.01
3. Step-change	2.65	0.95	.005	2.89	0.90	.001	0.24
4. Slope-change	-0.02	0.21	.908	0.02	0.21	0.930	0.04
High Income							
1. Intercept (2009)	15.29	0.93	<.001	15.23	0.92	<.001	0.06
2. Linear trend	-0.01	0.09	.886	-0.01	0.08	.946	0.00
3. Step-change	0.49	0.54	.360	0.59	0.51	.253	0.00
4. Slope-change	0.49	0.16	.002	0.49	0.16	.001	0.06

© 2025 Fuller-Rowell TE et al. *JAMA Network Open.*

eTable 4. Comparison of Model Fit Statistics for Alternative Trajectory Structures

Model	AIC	BIC	Parameters	Log-Likelihood	Deviance
Full Sample					
1. Linear trend	13240.7	13274.8	6	-6614.4	13228.7
2. Slope-change	13195.2	13252.0	10	-6587.6	13175.2
3. Step-change	13146.8	13203.6	10	-6563.4	13126.8
4. Slope-change + step-change	13119.7	13205.0	14	-6544.8	13089.7
Low Income					
1. Linear trend	13995.9	14029.8	6	-6991.9	13983.9
2. Slope-change	13967.0	14023.6	10	-6973.5	13947.0
3. Step-change	13868.4	13925.0	10	-6924.2	13848.4
4. Slope-change + step-change	13859.3	13944.2	14	-6914.6	13829.3
High Income					
1. Linear trend	12732.7	12766.7	6	-6360.3	12720.7
2. Slope-change	12688.8	12745.4	10	-6334.4	12668.8
3. Step-change	12704.1	12760.7	10	-6342.1	12684.1
4. Slope-change + step-change	12672.3	12757.3	14	-6321.2	12642.3

Note. Slope-change = linear slope parameter + parameter allowing the slope to change after 2020; Step-change = linear slope parameter + parameter allowing a discontinuity (upward or downward shift) between 2019 and 2020.

© 2025 Fuller-Rowell TE et al. *JAMA Network Open.*

eTable 5. Multilevel Discontinuous Growth Curve Parameter Estimates for Full Sample, Low-Income (Bottom 40%), and High-Income (Top 40%) Groups

	Estimate	95% CI	*p*-value	% Change
Full Sample				
1. Intercept (2009)	19.22	(17.28, 21.16)	<.001	--
2. Linear trend (2009-2019)	0.04	(-0.13, 0.20)	.681	0.21
3. Step-change (2019-2020)	1.50	(0.30, 2.70)	.01	7.66
4. Linear trend (2020-2024)	0.15	(-0.19, 0.48)	.395	.71
5. End point (2024)	21.80	(19.39, 24.21)	<.001	13.42
Low Income				
1. Intercept (2009)	24.07	(21.63, 26.51)	<.001	--
2. Linear trend (2009-2019)	-0.03	(-0.25, 0.19)	.795	0.12
3. Step-change (2019-2020)	2.62	(0.86, 4.38)	.003	11.01
4. Linear trend (2020-2024)	-0.02	(-0.44, 0.39)	.908	0.08
5. End point (2024)	26.19	(23.54, 28.83)	<.001	8.80
High Income				
1. Intercept (2009)	15.29	(13.46, 17.11)	<.001	--
2. Linear trend (2009-2019)	-0.01	(-0.18, 0.15)	.886	0.07
3. Step-change (2019-2020)	0.48	(-0.52, 1.49)	.347	3.17
4. Linear trend (2020-2024)	0.49	(0.18, 0.81)	.002	3.13
5. End point (2024)	17.58	(15.33, 19.83)	<.001	14.98

© 2025 Fuller-Rowell TE et al. *JAMA Network Open.*

eTable 6. Country Rankings on Changes in Social Isolation Trajectories (2009–2024)

No.	Country	Change in Disparity (Rank)	Change in Mean (Rank)	Combined Score (Rank)
1	Georgia	-19.02 (5)	-26.68 (2)	-45.69 (1)
2	Angola	-19.64 (4)	-22.29 (5)	-41.93 (2)
3	Mozambique	-14.74 (11)	-24.12 (4)	-38.85 (3)
4	Namibia	-17.21 (9)	-20.54 (6)	-37.74 (4)
5	Haiti	-29.39 (1)	-5.81 (33)	-35.19 (5)
6	Togo	-7.14 (28)	-27.79 (1)	-34.93 (6)
7	Yemen	-21.93 (3)	-11.51 (16)	-33.45 (7)
8	Iran	-6.29 (35)	-25.24 (3)	-31.52 (8)
9	Kosovo	-17.51 (7)	-10.60 (18)	-28.11 (9)
10	Liberia	-14.44 (13)	-13.48 (12)	-27.92 (10)
11	Tajikistan	-7.96 (26)	-18.30 (7)	-26.26 (11)
12	Bosnia and Herzegovina	-10.54 (21)	-15.62 (9)	-26.15 (12)
13	Bulgaria	-12.38 (16)	-12.00 (15)	-24.39 (13)
14	North Macedonia	-10.16 (22)	-14.22 (11)	-24.37 (14)
15	Serbia	-5.61 (43)	-16.57 (8)	-22.18 (15)
16	Ethiopia	-14.44 (12)	-6.30 (29)	-20.74 (16)
17	Gabon	-12.95 (15)	-6.41 (28)	-19.35 (17)
18	Belize	-6.02 (39)	-12.54 (14)	-18.56 (18)
19	Singapore	-11.85 (17)	-3.19 (48)	-15.04 (19)
20	Algeria	-19.01 (6)	4.04 (100)	-14.97 (20)
21	Tunisia	-13.65 (14)	-1.300 (57)	-14.95 (21)
22	Kyrgyzstan	-6.26 (36)	-7.51 (25)	-13.77 (22)
23	Philippines	-10.56 (19)	-2.74 (50)	-13.30 (23)
24	Hungary	-4.11 (47)	-8.77 (22)	-12.88 (24)
25	Latvia	-1.38 (62)	-11.35 (17)	-12.74 (25)

© 2025 Fuller-Rowell TE et al. *JAMA Network Open.*

26	Vietnam	-6.65 (31)	-6.08 (30)	-12.73 (26)
27	Jamaica	-10.55 (20)	-2.17 (55)	-12.72(27)
28	Croatia	1.84 (91)	-14.50 (10)	-12.67 (28)
29	Taiwan	-6.47 (33)	-6.02 (31)	-12.49 (29)
30	Armenia	-1.98 (56)	-10.13 (20)	-12.11 (30)
31	Gambia	-5.98 (40)	-5.90 (32)	-11.88 (31)
32	Chile	-6.41 (34)	-5.42 (35)	-11.83 (32)
33	Mauritius	-1.39 (61)	-10.20 (19)	-11.60 (33)
34	Kazakhstan	-6.59 (32)	-3.93 (40)	-10.53 (34)
35	China	-9.24 (24)	-1.17 (60)	-10.40 (35)
36	Slovakia	-4.20 (46)	-3.72 (43)	-7.92 (36)
37	Pakistan	-1.05 (67)	-6.64 (27)	-7.68 (37)
38	Myanmar	-1.96 (57)	-5.73 (34)	-7.68 (38)
39	Turkiye	0.064 (78)	-7.71 (24)	-7.64 (39)
40	Nepal	-9.95 (23)	3.47 (93)	-6.49 (40)
41	Israel	-2.09 (54)	-4.34 (38)	-6.43 (41)
42	Montenegro	3.25 (113)	-9.44 (21)	-6.19 (42)
43	Mexico	-0.84 (69)	-4.27 (39)	-5.10 (43)
44	State of Palestine	2.49 (98)	-7.15 (26)	-4.66 (44)
45	Greece	-1.06 (65)	-3.34 (46)	-4.40 (45)
46	Spain	-6.18 (37)	2.07 (82)	-4.11 (46)
47	Slovenia	-1.13 (64)	-2.79 (49)	-3.92 (47)
48	Portugal	-1.52 (60)	-2.28 (53)	-3.80 (48)
49	Peru	-1.06 (66)	-2.55 (51)	-3.61 (49)
50	Congo Brazzaville	-8.09 (25)	4.66 (104)	-3.43 (50)
51	Finland	0.42 (80)	-3.34 (45)	-2.93 (51)
52	Romania	1.71 (90)	-4.51 (37)	-2.81 (52)
53	Nicaragua	-1.79 (58)	-0.90 (61)	-2.69 (53)

© 2025 Fuller-Rowell TE et al. *JAMA Network Open.*

54	Laos	-3.61 (50)	1.07 (73)	-2.54 (54)
55	Trinidad and Tobago	-7.78 (27)	5.52 (111)	-2.25 (55)
56	Estonia	1.67 (89)	-3.75 (42)	-2.08 (56)
57	Iceland	-1.28 (63)	-0.80 (62)	-2.08 (57)
58	France	-6.83 (29)	5.47 (110)	-1.36 (58)
59	Belarus	-2.58 (52)	1.34 (78)	-1.24 (59)
60	Mongolia	0.036 (75)	-1.22 (59)	-1.18 (60)
61	Bolivia	-3.44 (51)	2.42 (83)	-1.02 (61)
62	El Salvador	-1.65 (59)	0.89 (71)	-0.76 (62)
63	Dominican Republic	0.63 (81)	-1.28 (58)	-0.65 (63)
64	Lithuania	1.21 (85)	-1.51 (56)	-0.30 (64)
65	Turkmenistan	3.61 (115)	-3.89 (41)	-0.28 (65)
66	Lebanon	4.66 (124)	-4.74 (36)	-0.08 (66)
67	Guatemala	-5.93 (41)	6.09 (113)	0.16 (67)
68	Denmark	-0.62 (70)	0.87 (70)	0.25 (68)
69	South Korea	-0.51 (71)	1.08 (74)	0.57 (69)
70	Norway	-0.44 (73)	1.24 (75)	0.80 (70)
71	Malta	-0.35 (74)	1.35 (79)	1.01 (71)
72	Cambodia	4.68 (127)	-3.45 (44)	1.22 (72)
73	Honduras	-3.83 (48)	5.23 (108)	1.40 (73)
74	Guinea	4.73 (128)	-3.33 (47)	1.40 (74)
75	Argentina	0.79 (82)	0.65 (68)	1.44 (75)
76	Czech Republic	1.53 (88)	0.20 (65)	1.73 (76)
77	Indonesia	-0.91 (68)	2.66 (87)	1.75 (77)
78	Saudi Arabia	2.24 (95)	-0.09 (64)	2.15 (78)
79	Uruguay	4.75 (129)	-2.47 (52)	2.29 (79)
80	Nigeria	-1.99 (55)	4.41 (102)	2.43 (80)
81	United States of America	1.85 (92)	0.77 (69)	2.62 (81)

© 2025 Fuller-Rowell TE et al. *JAMA Network Open.*

82	Ghana	3.20 (109)	-0.56 (63)	2.64 (82)
83	Panama	2.01 (94)	0.97 (72)	2.98 (83)
84	Colombia	0.04 (76)	3.01 (92)	3.05 (84)
85	Uzbekistan	2.36 (96)	1.31 (77)	3.67 (85)
86	Qatar	-25.94 (2)	29.84 (158)	3.90 (86)
87	Luxembourg	-3.65 (49)	7.72 (121)	4.07 (87)
88	Switzerland	0.20 (79)	3.99 (99)	4.19 (88)
89	Japan	-0.51 (72)	4.84 (105)	4.34 (89)
90	India	6.77 (137)	-2.20 (54)	4.57 (90)
91	Belgium	2.70 (100)	1.92 (81)	4.62 (91)
92	Italy	3.19 (108)	1.81 (80)	5.00 (92)
93	Costa Rica	4.66 (126)	0.50 (66)	5.16 (93)
94	Hong Kong	4.04 (120)	1.27 (76)	5.32 (94)
95	Republic of Cyprus	2.70 (101)	2.87 (90)	5.57 (95)
96	Austria	0.06 (77)	5.64 (112)	5.70 (96)
97	Paraguay	3.21 (110)	2.64 (86)	5.85 (97)
98	Morocco	-4.65 (45)	10.74 (133)	6.09 (98)
99	Ecuador	3.69 (117)	2.46 (85)	6.15 (99)
100	Russia	1.44 (86)	4.90 (106)	6.34 (100)
101	Azerbaijan	3.21 (111)	3.70 (95)	6.91 (101)
102	Brazil	3.24 (112)	3.85 (97)	7.09 (102)
103	Burundi	20.95 (157)	-13.27 (13)	7.68 (103)
104	Madagascar	-5.05 (44)	12.84 (134)	7.79 (104)
105	Canada	4.15 (123)	3.84 (96)	7.98 (105)
106	Sierra Leone	-16.16 (10)	24.17 (153)	8.02 (106)
107	Netherlands	5.38 (131)	2.97 (91)	8.35 (107)
108	Moldova	4.13 (122)	4.41 (101)	8.53 (108)
109	New Zealand	4.08 (121)	4.51 (103)	8.60 (109)

© 2025 Fuller-Rowell TE et al. *JAMA Network Open.*

110	Germany	3.66 (116)	4.95 (107)	8.60 (110)
111	Poland	1.45 (87)	7.53 (119)	8.98 (111)
112	Ukraine	5.39 (132)	3.92 (98)	9.31 (112)
113	Benin	-5.68 (42)	14.99 (141)	9.31 (113)
114	Congo (Kinshasa)	0.93 (84)	8.38 (123)	9.32 (114)
115	Australia	3.26 (114)	6.11 (114)	9.37 (115)
116	Thailand	6.55 (136)	2.86 (89)	9.41 (116)
117	Ireland	2.79 (102)	6.69 (115)	9.48 (117)
118	Sri Lanka	2.64 (99)	6.92 (116)	9.56 (118)
119	Malaysia	4.66 (125)	5.35 (109)	10.01 (119)
120	United Arab Emirates	-2.56 (53)	12.89 (135)	10.34 (120)
121	Mauritania	2.90 (103)	7.46 (118)	10.35 (121)
122	South Africa	3.07 (106)	7.36 (117)	10.42 (122)
123	Niger	-6.70 (30)	17.35 (145)	10.65 (123)
124	Comoros	-10.96 (18)	21.69 (151)	10.73 (124)
125	Uganda	2.38 (97)	8.48 (125)	10.87 (125)
126	Bahrain	1.91 (93)	9.15 (127)	11.06 (126)
127	United Kingdom of Great Britain and Northern Ireland	3.06 (105)	8.39 (124)	11.45 (127)
128	Bhutan	20.65 (156)	-7.97 (23)	12.68 (128)
129	Cameroon	3.88 (119)	9.20 (128)	13.07 (129)
130	Sweden	9.77 (140)	3.61 (94)	13.38 (130)
131	Senegal	6.05 (134)	7.78 (122)	13.84 (131)
132	Iraq	11.14 (143)	2.80 (88)	13.94 (132)
133	Malawi	-6.10 (38)	20.07 (149)	13.97 (133)
134	Cote dIvoire	4.99 (130)	10.15 (131)	15.14 (134)
135	Eswatini	3.09 (107)	13.00 (136)	16.08 (135)

© 2025 Fuller-Rowell TE et al. *JAMA Network Open.*

136	Venezuela	5.84 (133)	10.27 (132)	16.11 (136)
137	Puerto Rico	13.89 (152)	2.42 (84)	16.31 (137)
138	Rwanda	16.36 (154)	0.54 (67)	16.90 (138)
139	Mali	3.02 (104)	15.72 (142)	18.74 (139)
140	Egypt	11.54 (144)	7.56 (120)	19.103 (140)
141	Kuwait	10.82 (142)	10.11 (130)	20.93 (141)
142	Jordan	6.21 (135)	14.79 (140)	21.00 (142)
143	Bangladesh	3.75 (118)	17.61 (146)	21.36 (143)
144	Albania	13.15 (147)	8.80 (126)	21.95 (144)
145	Burkina Faso	10.04 (141)	14.72 (138)	24.76 (145)
146	Botswana	8.76 (138)	16.92 (144)	25.69 (146)
147	Libya	13.59 (151)	13.48 (137)	27.08 (147)
148	Central African Republic	0.83 (83)	26.85 (156)	27.69 (148)
149	Zambia	9.27 (139)	18.99 (147)	28.25 (149)
150	Lesotho	13.59 (150)	14.73 (139)	28.32 (150)
151	Kenya	12.80 (146)	15.96 (143)	28.75 (151)
152	Chad	13.47 (149)	21.57 (150)	35.03 (152)
153	Tanzania	12.43 (145)	24.98 (154)	37.40 (153)
154	Sudan	13.46 (148)	26.03 (155)	39.49 (154)
155	Afghanistan	14.32 (153)	28.14 (157)	42.46 (155)
156	Zimbabwe	19.81 (155)	23.51 (152)	43.32 (156)
157	South Sudan	29.07 (158)	19.09 (148)	48.15 (157)
158	Somalia	41.66 (159)	9.56 (129)	51.22 (158)
159	Syria	-17.25 (8)	68.47 (159)	51.22 (159)

© 2025 Fuller-Rowell TE et al. *JAMA Network Open.*

eTable 7. Regional Rankings from Best to Worst for Social Isolation Trajectories (2009-2024)

Region	Change in Disparity (Ranking)	Change in Mean (Ranking)	Combined score (Ranking)
RFSU	-2.02 (4)	-4.24 (1)	-6.26 (1)
Southeast Asia	-2.18 (2)	-1.03 (3)	-3.21 (2)
Europe	-0.79 (6)	-1.65 (2)	-2.44 (3)
East Asia	-2.11 (3)	-0.20 (4)	-2.31 (4)
LAC	-1.56 (5)	0.41 (5)	-1.15 (5)
MENA	-2.56 (1)	6.41 (9)	3.85 (6)
North America	3.00 (8)	2.31 (6)	5.30 (7)
ANZ	3.67 (9)	5.31 (7)	8.98 (8)
Sub-Saharan Africa	2.19 (7)	6.83 (10)	9.02 (9)
South Asia	5.30 (10)	5.62 (8)	10.92 (10)

Note. RFSU = Russia and Former Soviet Union Countries that are not part of the European Union; ANZ = Australia and New Zealand; MENA = Middle East and North Africa; LAC = Latin America and the Caribbean

.

eTable 8. Country Rankings Within Each Region on Changes in Social Isolation Trajectories (2009-2024)

Region (No of Country)	Country	Change in Disparity	Change in Mean	Combined Score
ANZ (2)	New Zealand	4.08 (2)	4.52 (1)	8.6 (1)
	Australia	3.26 (1)	6.11 (2)	9.37 (2)
East Asia (6)	Taiwan	-6.47 (2)	-6.02 (1)	-12.49 (1)
	China	-9.24 (1)	-1.17 (3)	-10.4 (2)
	Mongolia	0.04 (5)	-1.22 (2)	-1.18 (3)
	South Korea	-0.51 (3)	1.08 (4)	0.57 (4)
	Japan	-0.51 (4)	4.84 (6)	4.34 (5)
	Hong Kong	4.04 (6)	1.27 (5)	5.32 (6)
Europe (37)	Kosovo	-17.51 (1)	-10.6 (7)	-28.11 (1)
	Bosnia and Herzegovina	-10.54 (3)	-15.62 (2)	-26.15 (2)
	Bulgaria	-12.38 (2)	-12.0 (5)	-24.39 (3)
	North Macedonia	-10.16 (4)	-14.22 (4)	-24.37 (4)
	Serbia	-5.61 (7)	-16.57 (1)	-22.18 (5)
	Hungary	-4.11 (9)	-8.77 (9)	-12.88 (6)
	Latvia	-1.38 (12)	-11.35 (6)	-12.73 (7)
	Croatia	1.84 (27)	-14.5 (3)	-12.67 (8)
	Slovakia	-4.2 (8)	-3.72 (12)	-7.92 (9)
	Montenegro	3.25 (33)	-9.43 (8)	-6.18 (10)
	Greece	-1.06 (15)	-3.34 (14)	-4.4 (11)
	Spain	-6.18 (6)	2.07 (25)	-4.11 (12)
	Slovenia	-1.13 (14)	-2.79 (15)	-3.92 (13)
	Portugal	-1.52 (11)	-2.28 (16)	-3.8 (14)
	Finland	0.42 (21)	-3.34 (13)	-2.93 (15)
	Romania	1.71 (26)	-4.51 (10)	-2.81 (16)
	Estonia	1.67 (25)	-3.75 (11)	-2.08 (17)

© 2025 Fuller-Rowell TE et al. *JAMA Network Open.*

	Iceland	-1.28 (13)	-0.8 (18)	-2.08 (18)
	France	-6.83 (5)	5.47 (31)	-1.36 (19)
	Lithuania	1.21 (22)	-1.51 (17)	-0.3 (20)
	Denmark	-0.62 (16)	0.87 (20)	0.25 (21)
	Norway	-0.44 (17)	1.24 (21)	0.8 (22)
	Malta	-0.35 (18)	1.35 (22)	1.01 (23)
	Czech Republic	1.53 (24)	0.2 (19)	1.73 (24)
	Luxembourg	-3.65 (10)	7.72 (35)	4.07 (25)
	Switzerland	0.21 (20)	3.99 (29)	4.19 (26)
	Belgium	2.7 (28)	1.92 (24)	4.62 (27)
	Italy	3.19 (32)	1.81 (23)	5.0 (28)
	Republic of Cyprus	2.7 (29)	2.87 (26)	5.57 (29)
	Austria	0.06 (19)	5.64 (32)	5.7 (30)
	Netherlands	5.38 (35)	2.97 (27)	8.35 (31)
	Germany	3.66 (34)	4.95 (30)	8.6 (32)
	Poland	1.45 (23)	7.53 (34)	8.98 (33)
	Ireland	2.79 (30)	6.69 (33)	9.48 (34)
	United Kingdom of Great Britain and Northern Ireland	3.06 (31)	8.39 (36)	11.45 (35)
	Sweden	9.77 (36)	3.61 (28)	13.38 (36)
	Albania	13.15 (37)	8.8 (37)	21.95 (37)
LAC (23)	Haiti	-29.39 (1)	-5.81 (2)	-35.19 (1)
	Belize	-6.02 (5)	-12.54 (1)	-18.56 (2)
	Jamaica	-10.55 (2)	-2.17 (7)	-12.72 (3)
	Chile	-6.41 (4)	-5.42 (3)	-11.83 (4)
	Mexico	-0.84 (12)	-4.27 (4)	-5.1 (5)
	Peru	-1.06 (11)	-2.55 (5)	-3.61 (6)

© 2025 Fuller-Rowell TE et al. *JAMA Network Open.*

	Nicaragua	-1.79 (9)	-0.9 (9)	-2.69 (7)
	Trinidad and Tobago	-7.77 (3)	5.52 (21)	-2.25 (8)
	Bolivia	-3.44 (8)	2.42 (14)	-1.02 (9)
	El Salvador	-1.65 (10)	0.89 (12)	-0.76 (10)
	Dominican Republic	0.63 (14)	-1.28 (8)	-0.65 (11)
	Guatemala	-5.93 (6)	6.09 (22)	0.16 (12)
	Honduras	-3.83 (7)	5.23 (20)	1.4 (13)
	Argentina	0.79 (15)	0.65 (11)	1.44 (14)
	Uruguay	4.75 (21)	-2.47 (6)	2.29 (15)
	Panama	2.01 (16)	0.97 (13)	2.98 (16)
	Colombia	0.04 (13)	3.01 (18)	3.05 (17)
	Costa Rica	4.66 (20)	0.5 (10)	5.16 (18)
	Paraguay	3.21 (17)	2.64 (17)	5.85 (19)
	Ecuador	3.69 (19)	2.46 (16)	6.15 (20)
	Brazil	3.24 (18)	3.85 (19)	7.09 (21)
	Venezuela	5.84 (22)	10.27 (23)	16.11 (22)
	Puerto Rico	13.89 (23)	2.42 (15)	16.31 (23)
MENA (19)	Yemen	-21.93 (2)	-11.51 (2)	-33.45 (1)
	Iran	-6.29 (6)	-25.24 (1)	-31.52 (2)
	Algeria	-19.01 (3)	4.04 (10)	-14.97 (3)
	Tunisia	-13.65 (5)	-1.3 (7)	-14.95 (4)
	Turkiye	0.06 (10)	-7.71 (3)	-7.64 (5)
	Israel	-2.09 (9)	-4.34 (6)	-6.43 (6)
	State of Palestine	2.49 (13)	-7.15 (4)	-4.66 (7)
	Lebanon	4.66 (14)	-4.74 (5)	-0.08 (8)
	Saudi Arabia	2.24 (12)	-0.09 (8)	2.15 (9)
	Qatar	-25.94 (1)	29.84 (18)	3.9 (10)
	Morocco	-4.65 (7)	10.74 (14)	6.09 (11)

	United Arab Emirates	-2.56 (8)	12.89 (15)	10.34 (12)
	Bahrain	1.91 (11)	9.15 (12)	11.06 (13)
	Iraq	11.14 (17)	2.8 (9)	13.94 (14)
	Egypt	11.54 (18)	7.56 (11)	19.1 (15)
	Kuwait	10.82 (16)	10.11 (13)	20.93 (16)
	Jordan	6.21 (15)	14.79 (17)	21.0 (17)
	Libya	13.59 (19)	13.48 (16)	27.08 (18)
	Syria	-17.25 (4)	68.47 (19)	51.22 (19)
NAM (2)	United States of America	1.85 (1)	0.77 (1)	2.62 (1)
	Canada	4.15 (2)	3.84 (2)	7.98 (2)
RFSU (12)	Georgia	-19.01 (1)	-26.68 (1)	-45.69 (1)
	Tajikistan	-7.96 (2)	-18.3 (2)	-26.26 (2)
	Kyrgyzstan	-6.26 (4)	-7.51 (4)	-13.77 (3)
	Armenia	-1.98 (6)	-10.13 (3)	-12.11 (4)
	Kazakhstan	-6.59 (3)	-3.94 (5)	-10.53 (5)
	Belarus	-2.58 (5)	1.34 (8)	-1.24 (6)
	Turkmenistan	3.61 (10)	-3.89 (6)	-0.28 (7)
	Uzbekistan	2.36 (8)	1.31 (7)	3.67 (8)
	Russia	1.44 (7)	4.9 (12)	6.34 (9)
	Azerbaijan	3.21 (9)	3.7 (9)	6.91 (10)
	Moldova	4.13 (11)	4.41 (11)	8.53 (11)
	Ukraine	5.39 (12)	3.92 (10)	9.31 (12)
South Asia (7)	Pakistan	-1.04 (2)	-6.64 (2)	-7.68 (1)
	Nepal	-9.95 (1)	3.47 (4)	-6.49 (2)
	India	6.77 (5)	-2.2 (3)	4.57 (3)
	Sri Lanka	2.64 (3)	6.92 (5)	9.56 (4)
	Bhutan	20.65 (7)	-7.97 (1)	12.68 (5)
	Bangladesh	3.75 (4)	17.61 (6)	21.36 (6)

© 2025 Fuller-Rowell TE et al. *JAMA Network Open.*

	Afghanistan	14.32 (6)	28.14 (7)	42.46 (7)
Southeast Asia (9)	Singapore	-11.85 (1)	-3.19 (4)	-15.04 (1)
	Philippines	-10.56 (2)	-2.74 (5)	-13.3 (2)
	Vietnam	-6.65 (3)	-6.08 (1)	-12.73 (3)
	Myanmar	-1.96 (5)	-5.72 (2)	-7.68 (4)
	Laos	-3.61 (4)	1.07 (6)	-2.54 (5)
	Cambodia	4.68 (8)	-3.45 (3)	1.22 (6)
	Indonesia	-0.91 (6)	2.66 (7)	1.75 (7)
	Thailand	6.55 (9)	2.86 (8)	9.41 (8)
	Malaysia	4.66 (7)	5.35 (9)	10.01 (9)
Sub-Saharan Africa (42)	Angola	-19.64 (1)	-22.29 (3)	-41.93 (1)
	Mozambique	-14.74 (4)	-24.12 (2)	-38.85 (2)
	Namibia	-17.21 (2)	-20.54 (4)	-37.74 (3)
	Togo	-7.14 (10)	-27.79 (1)	-34.93 (4)
	Liberia	-14.44 (6)	-13.48 (5)	-27.92 (5)
	Ethiopia	-14.44 (5)	-6.3 (9)	-20.74 (6)
	Gabon	-12.95 (7)	-6.41 (8)	-19.35 (7)
	Gambia	-5.98 (13)	-5.9 (10)	-11.88 (8)
	Mauritius	-1.39 (17)	-10.21 (7)	-11.6 (9)
	Congo Brazzaville	-8.09 (9)	4.66 (15)	-3.43 (10)
	Guinea	4.73 (27)	-3.33 (11)	1.4 (11)
	Nigeria	-1.98 (16)	4.41 (14)	2.43 (12)
	Ghana	3.2 (25)	-0.56 (12)	2.64 (13)
	Burundi	20.95 (40)	-13.27 (6)	7.68 (14)
	Madagascar	-5.05 (15)	12.84 (24)	7.79 (15)
	Sierra Leone	-16.16 (3)	24.17 (39)	8.02 (16)
	Benin	-5.68 (14)	14.99 (28)	9.31 (17)
	Congo (Kinshasa)	0.93 (19)	8.38 (19)	9.32 (18)

© 2025 Fuller-Rowell TE et al. *JAMA Network Open.*

Mauritania	2.9 (21)	7.46 (17)	10.35 (19)
South Africa	3.07 (23)	7.36 (16)	10.42 (20)
Niger	-6.7 (11)	17.35 (32)	10.65 (21)
Comoros	-10.97 (8)	21.7 (37)	10.73 (22)
Uganda	2.38 (20)	8.48 (20)	10.87 (23)
Cameroon	3.88 (26)	9.2 (21)	13.07 (24)
Senegal	6.05 (29)	7.78 (18)	13.83 (25)
Malawi	-6.1 (12)	20.07 (35)	13.97 (26)
Cote dIvoire	4.99 (28)	10.15 (23)	15.14 (27)
Eswatini	3.09 (24)	13.0 (25)	16.08 (28)
Rwanda	16.36 (38)	0.54 (13)	16.9 (29)
Mali	3.02 (22)	15.72 (29)	18.74 (30)
Burkina Faso	10.04 (32)	14.72 (26)	24.76 (31)
Botswana	8.76 (30)	16.92 (31)	25.68 (32)
Central African Republic	0.84 (18)	26.85 (42)	27.69 (33)
Zambia	9.27 (31)	18.99 (33)	28.25 (34)
Lesotho	13.59 (37)	14.73 (27)	28.32 (35)
Kenya	12.8 (34)	15.96 (30)	28.75 (36)
Chad	13.46 (36)	21.57 (36)	35.04 (37)
Tanzania	12.43 (33)	24.98 (40)	37.4 (38)
Sudan	13.46 (35)	26.03 (41)	39.49 (39)
Zimbabwe	19.81 (39)	23.51 (38)	43.32 (40)
South Sudan	29.06 (41)	19.09 (34)	48.15 (41)
Somalia	41.66 (42)	9.56 (22)	51.22 (42)

© 2025 Fuller-Rowell TE et al. *JAMA Network Open.*

eTable 9. Global Summary of Social Isolation Trends by Trend Type

Trend Type	Number of Countries	List of Countries (Grouped by Region)	Δ Mean	Δ Disparity
Improving (decrease in mean levels and disparities)	41	11 in Europe (Bosnia and Herzegovina, Bulgaria, Greece, Hungary, Kosovo, Latvia, North Macedonia, Portugal, Serbia, Slovakia, Slovenia); 9 Sub-Saharan Africa (Angola, Ethiopia, Gabon, Gambia, Liberia, Mauritius, Mozambique, Namibia, Togo); 5 RFSU (Armenia, Georgia, Kazakhstan, Kyrgyzstan, Tajikistan); 5 LAC (Belize, Chile, Haiti, Jamaica, Peru); 4 MENA (Iran, Israel, Tunisia, Yemen); 4 South-East Asia (Myanmar, Philippines, Singapore, Vietnam);2 East Asia (China, Taiwan);1 South Asia (Pakistan)	-9.93	-8.89
Partly Improving (decrease in one; no change in the other)	8	2 in Europe (Finland, Iceland); 4 LAC (Dominican Republic, El Salvador, Mexico, Nicaragua); 1 MENA (Turkiye); 1 East Asia (Mongolia)	-2.33	-.55
Consistent (no change in mean levels or disparities)	2	1 Europe (Denmark); 1 LAC (Argentina)	0.76	0.09
Opposite (increase in mean levels; decrease in disparities)	23	8 in Sub-Saharan Africa (Benin, Comoros, Congo Brazzaville, Madagascar, Malawi, Niger, Nigeria, Sierra Leone); 3 Europe (France, Luxembourg, Spain); 5 MENA (Algeria, Morocco, Qatar, Syria, United Arab Emirates); 4 LAC (Bolivia, Guatemala, Hondurus,Trinidad and Tobago); 1 RFSU (Belarus);1 South Asia (Nepal); 1 South East Asia (Laos)	12.46	-8.00

© 2025 Fuller-Rowell TE et al. *JAMA Network Open.*

Opposite (increase in disparities; decrease in mean levels)	14	5 in Europe (Croatia, Estonia. Lithuania, Montenegro, Romania); 2 MENA (Lebanon, Palestine); 2 Sub-Saharan Africa (Burundi, Guinea); 2 South Asia (Bhutan, India); 1 South East Asia (Cambodia); 1 RFSU (Turkmenistan); 1 LAC (Uruguay)	-5.87	5.93
Partly Problematic (increase in one; no change in the other)	17	5 Europe (Austria, Czech Republic, Malta, Norway, Switzerland); 4 Sub-Saharan Africa (Central African Republic, Congo Kinshasa, Ghana, Rwanda); 3 in LAC (Colombia, Costa Rica, Panama); 2 East Asia (Japan, South Korea); 1 MENA (Saudi Arabia);1 North America (United States of America);1 South East Asia (Indonesia)	3.61	1.84
Problematic (increase in mean levels and disparities)	54	19 in Sub-Saharan Africa (Botswana, Burkina Faso, Cameroon, Chad, Cote dIvoire, Eswatini, Kenya, Lesotho, Mali, Mauritania, Senegal, Somalia, South Africa, South Sudan, Sudan, Tanzania, Uganda, Zambia, Zimbabwe); 10 Europe (Albania, Belgium, Germany, Ireland, Italy, Netherlands, Poland, Republic of Cyprus, Sweden, United Kingdom of Great Britain and Northern Ireland); 6 MENA (Bahrain, Egypt, Iraq, Jordan, Kuwait, Libya); 5 LAC (Brazil, Ecuador, Paraguay, Puerto Rico, Venezuela) ; 5 RFSU (Azerbaijan, Moldova, Russia, Ukraine, Uzbekistan); 3 South Asia (Afghanistan, Bangladesh, Sri Lanka); 2 South East Asia (Malaysia, Thailand); 2 ANZ (Australia , New Zealand); 1 North America (Canada); 1 East Asia (Hong Kong)	9.43	7.60

Note. RFSU = Russia and Former Soviet Union Countries that are not part of the European Union; LAC = Latin America and Caribbean, ANZ = Australia and New Zealand; MENA = Middle East and North Africa; Δ Mean = Mean change in Mean levels of social isolation between 2009 and 2024; Δ Disparity = Mean change in disparity between 2009 and 2024.

© 2025 Fuller-Rowell TE et al. *JAMA Network Open.*

eTable 10. Country Rankings for Social Isolation Levels and Disparities in 2024 (Best to Worst)

No.	Country	Disparity (Rank)	Mean Levels (Rank)	Combined score (Rank)
1	Iceland	0.16 (13)	1.3 (1)	1.46 (1)
2	Denmark	-0.83 (8)	4.4 (5)	3.57 (2)
3	Slovakia	2.38 (25)	4.09 (4)	6.47 (3)
4	Slovenia	1.78 (21)	5.22 (7)	7.0 (4)
5	Spain	-0.58 (10)	7.97 (22)	7.38 (5)
6	Finland	3.73 (33)	3.65 (2)	7.38 (6)
7	Kazakhstan	0.03 (12)	7.47 (19)	7.5 (7)
8	Israel	2.06 (23)	5.85 (11)	7.91 (8)
9	Turkmenistan	4.32 (38)	3.8 (3)	8.12 (9)
10	Kyrgyzstan	3.0 (29)	5.52 (9)	8.52 (10)
11	Jamaica	-2.27 (5)	11.2 (44)	8.93 (11)
12	Croatia	2.93 (27)	7.09 (17)	10.03 (12)
13	Hungary	5.56 (56)	4.63 (6)	10.19 (13)
14	Austria	-1.04 (7)	11.28 (46)	10.24 (14)
15	Switzerland	0.89 (17)	9.5 (33)	10.39 (15)
16	New Zealand	2.98 (28)	7.49 (20)	10.47 (16)
17	Bulgaria	5.3 (51)	5.56 (10)	10.87 (17)
18	Latvia	4.87 (47)	6.46 (12)	11.34 (18)
19	Norway	4.85 (46)	6.49 (13)	11.34 (19)
20	Belarus	1.96 (22)	9.65 (34)	11.61 (20)
21	Luxembourg	-1.53 (6)	13.18 (63)	11.65 (21)
22	Belgium	3.59 (32)	8.21 (24)	11.81 (22)
23	Argentina	3.12 (30)	8.96 (29)	12.08 (23)
24	Mongolia	6.65 (68)	5.46 (8)	12.11 (24)
25	Uzbekistan	4.2 (36)	7.99 (23)	12.19 (25)

© 2025 Fuller-Rowell TE et al. *JAMA Network Open.*

26	Angola	-4.53 (3)	16.75 (75)	12.22 (26)
27	Czech Republic	5.34 (52)	6.96 (16)	12.3 (27)
28	Poland	1.28 (19)	11.42 (48)	12.7 (28)
29	Vietnam	0.96 (18)	11.88 (50)	12.83 (29)
30	France	-0.16 (11)	13.59 (64)	13.43 (30)
31	Lithuania	6.9 (72)	6.61 (14)	13.51 (31)
32	Japan	0.77 (16)	13.01 (61)	13.78 (32)
33	Malta	5.59 (57)	8.64 (26)	14.23 (33)
34	Estonia	7.53 (82)	6.76 (15)	14.29 (34)
35	Ireland	5.49 (54)	8.9 (27)	14.38 (35)
36	Panama	3.75 (34)	10.81 (43)	14.56 (36)
37	Trinidad and Tobago	0.51 (15)	14.15 (66)	14.66 (37)
38	Australia	4.35 (39)	10.5 (40)	14.85 (38)
39	Germany	4.73 (45)	10.13 (37)	14.86 (39)
40	North Macedonia	4.46 (42)	10.46 (39)	14.92 (40)
41	Canada	5.95 (58)	9.02 (30)	14.98 (41)
42	Chile	3.54 (31)	11.48 (49)	15.03 (42)
43	Sweden	8.05 (91)	7.43 (18)	15.48 (43)
44	Netherlands	7.2 (78)	9.17 (31)	16.37 (44)
45	Serbia	7.78 (85)	8.61 (25)	16.39 (45)
46	Namibia	5.27 (50)	11.34 (47)	16.61 (46)
47	Mexico	4.24 (37)	12.43 (55)	16.67 (47)
48	United States of America	7.85 (88)	8.93 (28)	16.78 (48)
49	Uruguay	9.38 (96)	7.5 (21)	16.88 (49)
50	Tajikistan	6.9 (71)	10.11 (36)	17.0 (50)
51	Singapore	6.5 (64)	10.74 (42)	17.24 (51)
52	Yemen	-4.52 (4)	21.96 (97)	17.44 (52)
53	Dominican Republic	6.76 (70)	10.73 (41)	17.49 (53)

© 2025 Fuller-Rowell TE et al. *JAMA Network Open.*

54	United Kingdom of Great Britain and Northern Ireland	5.11 (49)	12.67 (57)	17.78 (54)
55	Paraguay	7.83 (87)	10.35 (38)	18.18 (55)
56	Costa Rica	8.62 (93)	9.76 (35)	18.38 (56)
57	Taiwan	7.29 (80)	11.23 (45)	18.51 (57)
58	Nicaragua	4.38 (40)	14.23 (67)	18.61 (58)
59	Bosnia and Herzegovina	6.54 (66)	12.2 (52)	18.74 (59)
60	Colombia	6.54 (65)	12.46 (56)	19.0 (60)
61	Thailand	6.46 (63)	12.72 (59)	19.18 (61)
62	Brazil	6.28 (60)	13.05 (62)	19.33 (62)
63	Algeria	-0.77 (9)	20.12 (89)	19.35 (63)
64	Saudi Arabia	6.97 (74)	12.72 (58)	19.7 (64)
65	Kosovo	4.41 (41)	15.34 (71)	19.74 (65)
66	Italy	7.13 (75)	12.78 (60)	19.91 (66)
67	Russia	6.28 (61)	14.07 (65)	20.35 (67)
68	Puerto Rico	11.54 (109)	9.47 (32)	21.01 (68)
69	Belize	6.63 (67)	14.59 (68)	21.22 (69)
70	Philippines	4.1 (35)	17.31 (77)	21.4 (70)
71	Qatar	-16.88 (1)	38.82 (138)	21.94 (71)
72	Portugal	10.11 (101)	12.07 (51)	22.18 (72)
73	Venezuela	6.27 (59)	16.26 (73)	22.53 (73)
74	Greece	7.28 (79)	15.33 (70)	22.61 (74)
75	Bahrain	4.5 (43)	18.17 (81)	22.67 (75)
76	Indonesia	5.51 (55)	19.0 (83)	24.51 (76)
77	Nigeria	0.24 (14)	24.38 (104)	24.62 (77)
78	Montenegro	12.55 (118)	12.37 (54)	24.93 (78)
79	South Africa	7.57 (83)	17.47 (78)	25.04 (79)
80	Bolivia	4.6 (44)	20.75 (91)	25.35 (80)

© 2025 Fuller-Rowell TE et al. *JAMA Network Open.*

81	United Arab Emirates	2.28 (24)	23.95 (102)	26.23 (81)
82	Ukraine	11.0 (108)	15.78 (72)	26.78 (82)
83	Mauritius	12.65 (119)	14.85 (69)	27.51 (83)
84	State of Palestine	9.8 (99)	17.93 (80)	27.73 (84)
85	Iran	10.39 (103)	17.92 (79)	28.31 (85)
86	Tunisia	6.43 (62)	22.57 (99)	29.0 (86)
87	Turkiye	12.69 (120)	16.47 (74)	29.16 (87)
88	Bhutan	16.85 (138)	12.34 (53)	29.19 (88)
89	Guatemala	6.96 (73)	22.72 (100)	29.68 (89)
90	Malaysia	8.42 (92)	21.9 (96)	30.31 (90)
91	Iraq	10.51 (104)	19.87 (87)	30.38 (91)
92	Uganda	10.94 (107)	19.72 (86)	30.66 (92)
93	Peru	12.37 (115)	18.71 (82)	31.08 (93)
94	Nepal	4.92 (48)	26.2 (113)	31.12 (94)
95	Ecuador	10.33 (102)	21.52 (95)	31.85 (95)
96	China	11.91 (111)	20.53 (90)	32.44 (96)
97	Honduras	7.9 (89)	24.78 (107)	32.68 (97)
98	Mozambique	7.52 (81)	25.66 (110)	33.18 (98)
99	Libya	9.53 (98)	23.76 (101)	33.29 (99)
100	Gambia	7.76 (84)	26.11 (112)	33.87 (100)
101	El Salvador	9.46 (97)	24.48 (106)	33.94 (101)
102	Republic of Cyprus	12.78 (121)	21.48 (94)	34.25 (102)
103	Georgia	12.39 (116)	22.29 (98)	34.68 (103)
104	South Korea	13.6 (124)	21.22 (92)	34.82 (104)
105	Armenia	14.26 (128)	21.36 (93)	35.63 (105)
106	Kuwait	16.59 (137)	19.04 (84)	35.63 (106)
107	Moldova	16.35 (135)	19.48 (85)	35.84 (107)
108	Hong Kong	18.71 (145)	17.15 (76)	35.85 (108)

© 2025 Fuller-Rowell TE et al. *JAMA Network Open.*

109	Romania	16.23 (134)	20.0 (88)	36.23 (109)
110	Sri Lanka	12.53 (117)	24.02 (103)	36.55 (110)
111	Eswatini	10.64 (106)	26.32 (114)	36.96 (111)
112	Jordan	12.25 (114)	24.91 (108)	37.16 (112)
113	Cambodia	14.03 (126)	25.19 (109)	39.21 (113)
114	Gabon	15.2 (131)	24.39 (105)	39.6 (114)
115	Ghana	11.99 (112)	30.07 (118)	42.06 (115)
116	Laos	9.31 (95)	33.29 (124)	42.6 (116)
117	Niger	1.73 (20)	41.41 (143)	43.14 (117)
118	Mali	7.8 (86)	35.43 (130)	43.23 (118)
119	Myanmar	15.44 (132)	28.22 (117)	43.66 (119)
120	Botswana	16.87 (139)	27.82 (115)	44.69 (120)
121	Madagascar	7.91 (90)	36.98 (135)	44.88 (121)
122	Ethiopia	9.93 (100)	35.66 (131)	45.59 (122)
123	Mauritania	10.59 (105)	35.13 (129)	45.72 (123)
124	Lebanon	20.15 (151)	25.96 (111)	46.1 (124)
125	Congo (Kinshasa)	14.46 (129)	31.67 (122)	46.14 (125)
126	Sierra Leone	7.15 (76)	39.68 (142)	46.83 (126)
127	Liberia	13.11 (122)	33.85 (127)	46.96 (127)
128	Lesotho	18.35 (144)	30.31 (119)	48.66 (128)
129	Pakistan	7.2 (77)	41.47 (144)	48.67 (129)
130	Senegal	18.87 (146)	30.82 (120)	49.69 (130)
131	Egypt	17.23 (140)	33.37 (125)	50.6 (131)
132	Togo	6.73 (69)	44.19 (148)	50.92 (132)
133	Albania	23.82 (156)	28.06 (116)	51.89 (133)
134	Kenya	21.06 (153)	30.94 (121)	52.0 (134)
135	Congo Brazzaville	13.44 (123)	39.33 (141)	52.77 (135)
136	Tanzania	16.37 (136)	37.95 (136)	54.31 (136)

© 2025 Fuller-Rowell TE et al. *JAMA Network Open.*

137	Cameroon	20.44 (152)	34.22 (128)	54.66 (137)
138	Comoros	5.48 (53)	49.38 (151)	54.86 (138)
139	Azerbaijan	23.41 (155)	31.79 (123)	55.2 (139)
140	Zambia	19.64 (149)	35.7 (132)	55.34 (140)
141	Guinea	13.79 (125)	42.76 (146)	56.55 (141)
142	Cote dIvoire	15.0 (130)	41.73 (145)	56.73 (142)
143	India	18.04 (141)	38.96 (139)	57.0 (143)
144	Haiti	14.24 (127)	43.52 (147)	57.76 (144)
145	Burkina Faso	19.15 (147)	39.3 (140)	58.45 (145)
146	Sudan	21.99 (154)	36.67 (133)	58.66 (146)
147	Rwanda	25.1 (157)	33.74 (126)	58.84 (147)
148	Morocco	11.64 (110)	47.66 (150)	59.3 (148)
149	Syria	-15.69 (2)	79.29 (158)	63.6 (149)
150	Chad	19.72 (150)	45.22 (149)	64.95 (150)
151	Benin	2.63 (26)	62.42 (155)	65.04 (151)
152	Zimbabwe	28.31 (158)	36.8 (134)	65.12 (152)
153	South Sudan	15.76 (133)	51.39 (153)	67.15 (153)
154	Malawi	12.03 (113)	55.57 (154)	67.6 (154)
155	Somalia	30.62 (159)	38.02 (137)	68.64 (155)
156	Burundi	18.05 (142)	50.6 (152)	68.66 (156)
157	Bangladesh	18.25 (143)	64.8 (156)	83.05 (157)
158	Central African Republic	8.84 (94)	79.53 (159)	88.37 (158)
159	Afghanistan	19.22 (148)	71.87 (157)	91.09 (159)

© 2025 Fuller-Rowell TE et al. *JAMA Network Open.*

eTable 11. Regional Rankings for Social Isolation Levels and Disparities in 2024 (Best to Worst)

No	Region	Disparity	Mean Isolation	Combined Score
1	ANZ	3.66 (1)	9.00 (2)	12.66 (1)
2	Europe	5.47 (2)	10.00 (3)	15.47 (2)
3	NAM	6.90 (5)	8.98 (1)	15.88 (3)
4	LAC	6.65 (4)	15.82 (6)	22.47 (4)
5	RFSU	8.67 (7)	14.11 (4)	22.79 (5)
6	East Asia	9.82 (8)	14.77 (5)	24.59 (6)
7	Southeast Asia	7.86 (6)	20.03 (7)	27.88 (7)
8	MENA	6.06 (3)	25.81 (8)	31.87 (8)
9	Sub Saharan Africa	13.00 (9)	35.74 (9)	48.75 (9)
10	South Asia	13.86 (10)	39.95 (10)	53.81 (10)

Note. RFSU = Russia and Former Soviet Union Countries that are not part of the European Union; ANZ = Australia and New Zealand; MENA = Middle East and North Africa; LAC = Latin America and the Caribbean.

© 2025 Fuller-Rowell TE et al. *JAMA Network Open.*

eTable 12. Country Ranking Within Each Region on Social Isolation Trajectories in 2024

Region	Country	Disparity	Mean Levels	Combined Score
ANZ (2)	Australia	4.35 (1)	10.50 (1)	14.85 (1)
	New Zealand	2.98 (2)	7.49 (2)	10.47 (2)
East Asia (6)	Hong Kong	18.71 (1)	17.15 (3)	35.85 (1)
	South Korea	13.60 (2)	21.22 (1)	34.82 (2)
	China	11.91 (3)	20.53 (2)	32.44 (3)
	Taiwan	7.29 (4)	11.23 (5)	18.51 (4)
	Japan	0.77 (6)	13.01 (4)	13.78 (5)
	Mongolia	6.65 (5)	5.46 (6)	12.11 (6)
Europe (37)	Albania	23.82 (1)	28.06 (1)	51.89 (1)
	Romania	16.23 (2)	20.00 (3)	36.23 (2)
	Republic of Cyprus	12.78 (3)	21.48 (2)	34.25 (3)
	Montenegro	12.55 (4)	12.37 (10)	24.93 (4)
	Greece	7.28 (9)	15.33 (5)	22.61 (5)
	Portugal	10.11 (5)	12.07 (12)	22.18 (6)
	Italy	7.13 (11)	12.78 (8)	19.91 (7)
	Kosovo	4.41 (24)	15.34 (4)	19.74 (8)
	Bosnia and Herzegovina	6.54 (13)	12.20 (11)	18.74 (9)
	United Kingdom of Great Britain and Northern Ireland	5.11 (19)	12.67 (9)	17.78 (10)
	Serbia	7.78 (7)	8.61 (21)	16.39 (11)
	Netherlands	7.20 (10)	9.17 (18)	16.37 (12)
	Sweden	8.05 (6)	7.43 (24)	15.48 (13)

© 2025 Fuller-Rowell TE et al. *JAMA Network Open.*

	North Macedonia	4.46 (23)	10.46 (15)	14.92 (14)
	Germany	4.73 (22)	10.13 (16)	14.86 (15)
	Ireland	5.49 (16)	8.90 (19)	14.38 (16)
	Estonia	7.53 (8)	6.76 (27)	14.29 (17)
	Malta	5.59 (14)	8.64 (20)	14.23 (18)
	Lithuania	6.90 (12)	6.61 (28)	13.51 (19)
	France	-0.16 (33)	13.59 (6)	13.43 (20)
	Poland	1.28 (30)	11.42 (13)	12.70 (21)
	Czech Republic	5.34 (17)	6.96 (26)	12.30 (22)
	Belgium	3.59 (26)	8.21 (22)	11.81 (23)
	Luxembourg	-1.53 (37)	13.18 (7)	11.65 (24)
	Latvia	4.87 (20)	6.46 (30)	11.34 (25)
	Norway	4.85 (21)	6.49 (29)	11.34 (25)
	Bulgaria	5.30 (18)	5.56 (31)	10.87 (27)
	Switzerland	0.89 (31)	9.50 (17)	10.39 (28)
	Austria	-1.04 (36)	11.28 (14)	10.24 (29)
	Hungary	5.56 (15)	4.63 (33)	10.19 (30)
	Croatia	2.93 (27)	7.09 (25)	10.03 (31)
	Spain	-0.58 (34)	7.97 (23)	7.38 (32)
	Finland	3.73 (25)	3.65 (36)	7.38 (32)
	Slovenia	1.78 (29)	5.22 (32)	7.00 (34)
	Slovakia	2.38 (28)	4.09 (35)	6.47 (35)
	Denmark	-0.83 (35)	4.40 (34)	3.57 (36)
	Iceland	0.16 (32)	1.30 (37)	1.46 (37)
RFSU (12)	Azerbaijan	23.41 (1)	31.79 (1)	55.20 (1)
	Moldova	16.35 (2)	19.48 (4)	35.84 (2)
	Armenia	14.26 (3)	21.36 (3)	35.63 (3)
	Georgia	12.39 (4)	22.29 (2)	34.68 (4)

© 2025 Fuller-Rowell TE et al. *JAMA Network Open.*

	Ukraine	11.00 (5)	15.78 (5)	26.78 (5)
	Russia	6.28 (7)	14.07 (6)	20.35 (6)
	Tajikistan	6.90 (6)	10.11 (7)	17.00 (7)
	Uzbekistan	4.20 (9)	7.99 (9)	12.19 (8)
	Belarus	1.96 (11)	9.65 (8)	11.61 (9)
	Kyrgyzstan	3.00 (10)	5.52 (11)	8.52 (10)
	Turkmenistan	4.32 (8)	3.80 (12)	8.12 (11)
	Kazakhstan	0.03 (12)	7.47 (10)	7.50 (12)
LAC (23)	Haiti	14.24 (1)	43.52 (1)	57.76 (1)
	El Salvador	9.46 (5)	24.48 (3)	33.94 (2)
	Honduras	7.90 (8)	24.78 (2)	32.68 (3)
	Ecuador	10.33 (4)	21.52 (5)	31.85 (4)
	Peru	12.37 (2)	18.71 (7)	31.08 (5)
	Guatemala	6.96 (10)	22.72 (4)	29.68 (6)
	Bolivia	4.60 (16)	20.75 (6)	25.35 (7)
	Venezuela	6.27 (15)	16.26 (8)	22.53 (8)
	Belize	6.63 (12)	14.59 (9)	21.22 (9)
	Puerto Rico	11.54 (3)	9.47 (21)	21.01 (10)
	Brazil	6.28 (14)	13.05 (12)	19.33 (11)
	Colombia	6.54 (13)	12.46 (13)	19.00 (12)
	Nicaragua	4.38 (17)	14.23 (10)	18.61 (13)
	Costa Rica	8.62 (7)	9.76 (20)	18.38 (14)
	Paraguay	7.83 (9)	10.35 (19)	18.18 (15)
	Dominican Republic	6.76 (11)	10.73 (18)	17.49 (16)
	Uruguay	9.38 (6)	7.50 (23)	16.88 (17)
	Mexico	4.24 (18)	12.43 (14)	16.67 (18)
	Chile	3.54 (20)	11.48 (15)	15.03 (19)

© 2025 Fuller-Rowell TE et al. *JAMA Network Open.*

	Trinidad and Tobago	0.51 (22)	14.15 (11)	14.66 (20)
	Panama	3.75 (19)	10.81 (17)	14.56 (21)
	Argentina	3.12 (21)	8.96 (22)	12.08 (22)
	Jamaica	-2.27 (23)	11.20 (16)	8.93 (23)
MENA (19)	Syria	-15.69 (18)	79.29 (1)	63.60 (1)
	Morocco	11.64 (6)	47.66 (2)	59.30 (2)
	Egypt	17.23 (2)	33.37 (4)	50.60 (3)
	Lebanon	20.15 (1)	25.96 (5)	46.10 (4)
	Jordan	12.25 (5)	24.91 (6)	37.16 (5)
	Kuwait	16.59 (3)	19.04 (13)	35.63 (6)
	Libya	9.53 (10)	23.76 (8)	33.29 (7)
	Iraq	10.51 (7)	19.87 (12)	30.38 (8)
	Turkiye	12.69 (4)	16.47 (17)	29.16 (9)
	Tunisia	6.43 (12)	22.57 (9)	29.00 (10)
	Iran	10.39 (8)	17.92 (16)	28.31 (11)
	State of Palestine	9.80 (9)	17.93 (15)	27.73 (12)
	United Arab Emirates	2.28 (14)	23.95 (7)	26.23 (13)
	Bahrain	4.50 (13)	18.17 (14)	22.67 (14)
	Qatar	-16.88 (19)	38.82 (3)	21.94 (15)
	Saudi Arabia	6.97 (11)	12.72 (18)	19.70 (16)
	Algeria	-0.77 (16)	20.12 (11)	19.35 (17)
	Yemen	-4.52 (17)	21.96 (10)	17.44 (18)
	Israel	2.06 (15)	5.85 (19)	7.91 (19)
North America (2)	United States of America	7.85 (1)	8.93 (2)	16.78 (1)
	Canada	5.95 (2)	9.02 (1)	14.98 (2)

© 2025 Fuller-Rowell TE et al. *JAMA Network Open.*

South Asia (7)	Afghanistan	19.22 (1)	71.87 (1)	91.09 (1)
	Bangladesh	18.25 (2)	64.80 (2)	83.05 (2)
	India	18.04 (3)	38.96 (4)	57.00 (3)
	Pakistan	7.20 (6)	41.47 (3)	48.67 (4)
	Sri Lanka	12.53 (5)	24.02 (6)	36.55 (5)
	Nepal	4.92 (7)	26.20 (5)	31.12 (6)
	Bhutan	16.85 (4)	12.34 (7)	29.19 (7)
Southeast Asia (9)	Myanmar	15.44 (1)	28.22 (2)	43.66 (1)
	Laos	9.31 (3)	33.29 (1)	42.60 (2)
	Cambodia	14.03 (2)	25.19 (3)	39.21 (3)
	Malaysia	8.42 (4)	21.90 (4)	30.31 (4)
	Indonesia	5.51 (7)	19.00 (5)	24.51 (5)
	Philippines	4.10 (8)	17.31 (6)	21.40 (6)
	Thailand	6.46 (6)	12.72 (7)	19.18 (7)
	Singapore	6.50 (5)	10.74 (9)	17.24 (8)
	Vietnam	0.96 (9)	11.88 (8)	12.83 (9)
Sub-Saharan Africa (42)	Central African Republic	8.84 (29)	79.53 (1)	88.37 (1)
	Burundi	18.05 (12)	50.60 (5)	68.66 (2)
	Somalia	30.62 (1)	38.02 (15)	68.64 (3)
	Malawi	12.03 (23)	55.57 (3)	67.60 (4)
	South Sudan	15.76 (15)	51.39 (4)	67.15 (5)
	Zimbabwe	28.31 (2)	36.80 (18)	65.12 (6)
	Benin	2.63 (39)	62.42 (2)	65.04 (7)
	Chad	19.72 (7)	45.22 (7)	64.95 (8)
	Rwanda	25.10 (3)	33.74 (26)	58.84 (9)
	Sudan	21.99 (4)	36.67 (19)	58.66 (10)
	Burkina Faso	19.15 (9)	39.30 (14)	58.45 (11)

© 2025 Fuller-Rowell TE et al. *JAMA Network Open.*

Cote dIvoire	15.00 (17)	41.73 (10)	56.73 (12)
Guinea	13.79 (19)	42.76 (9)	56.55 (13)
Zambia	19.64 (8)	35.70 (20)	55.34 (14)
Comoros	5.48 (37)	49.38 (6)	54.86 (15)
Cameroon	20.44 (6)	34.22 (24)	54.66 (16)
Tanzania	16.37 (14)	37.95 (16)	54.31 (17)
Congo Brazzaville	13.44 (20)	39.33 (13)	52.77 (18)
Kenya	21.06 (5)	30.94 (28)	52.00 (19)
Togo	6.73 (36)	44.19 (8)	50.92 (20)
Senegal	18.87 (10)	30.82 (29)	49.69 (21)
Lesotho	18.35 (11)	30.31 (30)	48.66 (22)
Liberia	13.11 (21)	33.85 (25)	46.96 (23)
Sierra Leone	7.15 (35)	39.68 (12)	46.83 (24)
Congo (Kinshasa)	14.46 (18)	31.67 (27)	46.14 (25)
Mauritania	10.59 (27)	35.13 (23)	45.72 (26)
Ethiopia	9.93 (28)	35.66 (21)	45.59 (27)
Madagascar	7.91 (30)	36.98 (17)	44.88 (28)
Botswana	16.87 (13)	27.82 (32)	44.69 (29)
Mali	7.80 (31)	35.43 (22)	43.23 (30)
Niger	1.73 (40)	41.41 (11)	43.14 (31)
Ghana	11.99 (24)	30.07 (31)	42.06 (32)
Gabon	15.20 (16)	24.39 (36)	39.60 (33)
Eswatini	10.64 (26)	26.32 (33)	36.96 (34)
Gambia	7.76 (32)	26.11 (34)	33.87 (35)
Mozambique	7.52 (34)	25.66 (35)	33.18 (36)
Uganda	10.94 (25)	19.72 (38)	30.66 (37)
Mauritius	12.65 (22)	14.85 (41)	27.51 (38)
South Africa	7.57 (33)	17.47 (39)	25.04 (39)

© 2025 Fuller-Rowell TE et al. *JAMA Network Open.*

	Nigeria	0.24 (41)	24.38 (37)	24.62 (40)
	Namibia	5.27 (38)	11.34 (42)	16.61 (41)
	Angola	-4.53 (42)	16.75 (40)	12.22 (42)

Note. RFSU = Russia and Former Soviet Union Countries that are not part of the European Union; ANZ = Australia and New Zealand; MENA = Middle East and North Africa; LAC = Latin America and the Caribbean.

© 2025 Fuller-Rowell TE et al. *JAMA Network Open.*
