# Supplementary material for: Global Trends and Disparities in Social Isolation
Source: JAMA Netw Open. 2025 Sep 15;8(9):e2532008. doi: 10.1001/jamanetworkopen.2025.32008 (PMC12439063; doi:10.1001/jamanetworkopen.2025.32008)
Supplement: Supplement 2. — Data Sharing Statement [file jamanetwopen-e2532008-s002.pdf]

Data Sharing Statement

Fuller-Rowell. Global Trends and Disparities in Social Isolation. *JAMA Netw Open*. Published September 15, 2025. doi:10.1001/jamanetworkopen.2025.32008

Data
Data available: No

Additional Information
Explanation for why data not available: Data for the Gallup World Poll is available from Gallup Analytics (https://www.gallup.com/analytics). A subscription is required to access the data and can be obtained by contacting Gallup Analytics. A list of university libraries with access to the Gallup World Poll data, and information about obtaining access, is available here: https://www.gallup.com/analytics/214565/universities-colleges-using-gallup-analytics.aspx.
